# Supplementary material for: HIVprotI: an integrated web based platform for prediction and design of HIV proteins inhibitors
Source: J Cheminform. 2018 Mar 9;10:12. doi: 10.1186/s13321-018-0266-y (PMC5845081; doi:10.1186/s13321-018-0266-y)
Supplement: Supplementary file 2 — Additional file 2. Source code of HIVProtI web server. [file 13321_2018_266_MOESM2_ESM.zip › HIVprotI_Source-code/hivprotI/weka-3-6-6/WekaManual.pdf]

THE UNIVERSITY OF
WAIKATO
Te Whare Wānanga o Waikato

WEKA Manual
for Version 3-6-6

Remco R. Bouckaert
Eibe Frank
Mark Hall
Richard Kirkby
Peter Reutemann
Alex Seewald
David Scuse

October 28, 2011

©2002-2011

University of Waikato, Hamilton, New Zealand
Alex Seewald (original Commnd-line primer)
David Scuse (original Experimenter tutorial)

This manual is licensed under the GNU General Public License version 2. More information about this license can be found at http://www.gnu.org/copyleft/gpl.html

Contents

I The Command-line 11

1 A command-line primer 13
1.1 Introduction . . . 13
1.2 Basic concepts . . . 14
1.2.1 Dataset . . . 14
1.2.2 Classifier . . . 16
1.2.3 weka.filters . . . 17
1.2.4 weka.classifiers . . . 19
1.3 Examples . . . 23

II The Graphical User Interface 25

2 Launching WEKA 27

3 Simple CLI 31
3.1 Commands . . . 31
3.2 Invocation . . . 32
3.3 Command redirection . . . 32
3.4 Command completion . . . 33

4 Explorer 35
4.1 The user interface . . . 35
4.1.1 Section Tabs . . . 35
4.1.2 Status Box . . . 35
4.1.3 Log Button . . . 36
4.1.4 WEKA Status Icon . . . 36
4.1.5 Graphical output . . . 36
4.2 Preprocessing . . . 37
4.2.1 Loading Data . . . 37
4.2.2 The Current Relation . . . 37
4.2.3 Working With Attributes . . . 38
4.2.4 Working With Filters . . . 39
4.3 Classification . . . 41
4.3.1 Selecting a Classifier . . . 41
4.3.2 Test Options . . . 41
4.3.3 The Class Attribute . . . 42
4.3.4 Training a Classifier . . . 43

4.3.5 The Classifier Output Text . . . 43
4.3.6 The Result List . . . 43
4.4 Clustering . . . 45
4.4.1 Selecting a Clusterer . . . 45
4.4.2 Cluster Modes . . . 45
4.4.3 Ignoring Attributes . . . 45
4.4.4 Working with Filters . . . 46
4.4.5 Learning Clusters . . . 46
4.5 Associating . . . 47
4.5.1 Setting Up . . . 47
4.5.2 Learning Associations . . . 47
4.6 Selecting Attributes . . . 48
4.6.1 Searching and Evaluating . . . 48
4.6.2 Options . . . 48
4.6.3 Performing Selection . . . 48
4.7 Visualizing . . . 50
4.7.1 The scatter plot matrix . . . 50
4.7.2 Selecting an individual 2D scatter plot . . . 50
4.7.3 Selecting Instances . . . 51

5 Experimenter 53
5.1 Introduction . . . 53
5.2 Standard Experiments . . . 54
5.2.1 Simple . . . 54
5.2.1.1 New experiment . . . 54
5.2.1.2 Results destination . . . 54
5.2.1.3 Experiment type . . . 56
5.2.1.4 Datasets . . . 58
5.2.1.5 Iteration control . . . 59
5.2.1.6 Algorithms . . . 59
5.2.1.7 Saving the setup . . . 61
5.2.1.8 Running an Experiment . . . 62
5.2.2 Advanced . . . 63
5.2.2.1 Defining an Experiment . . . 63
5.2.2.2 Running an Experiment . . . 66
5.2.2.3 Changing the Experiment Parameters . . . 68
5.2.2.4 Other Result Producers . . . 75
5.3 Cluster Experiments . . . 80
5.4 Remote Experiments . . . 83
5.4.1 Preparation . . . 83
5.4.2 Database Server Setup . . . 83
5.4.3 Remote Engine Setup . . . 84
5.4.4 Configuring the Experimenter . . . 85
5.4.5 Multi-core support . . . 86
5.4.6 Troubleshooting . . . 86
5.5 Analysing Results . . . 88
5.5.1 Setup . . . 88
5.5.2 Saving the Results . . . 91
5.5.3 Changing the Baseline Scheme . . . 91
5.5.4 Statistical Significance . . . 92

5.5.5 Summary Test 92
5.5.6 Ranking Test 93

6 KnowledgeFlow 95
6.1 Introduction 95
6.2 Features 97
6.3 Components 98
6.3.1 DataSources 98
6.3.2 DataSinks 98
6.3.3 Filters 98
6.3.4 Classifiers 98
6.3.5 Clusterers 98
6.3.6 Evaluation 99
6.3.7 Visualization 100
6.4 Examples 101
6.4.1 Cross-validated J48 101
6.4.2 Plotting multiple ROC curves 103
6.4.3 Processing data incrementally 105
6.5 Plugin Facility 107

7 ArffViewer 109
7.1 Menus 110
7.2 Editing 112

8 Bayesian Network Classifiers 115
8.1 Introduction 115
8.2 Local score based structure learning 119
8.2.1 Local score metrics 119
8.2.2 Search algorithms 120
8.3 Conditional independence test based structure learning 123
8.4 Global score metric based structure learning 125
8.5 Fixed structure 'learning' 126
8.6 Distribution learning 126
8.7 Running from the command line 128
8.8 Inspecting Bayesian networks 138
8.9 Bayes Network GUI 141
8.10 Bayesian nets in the experimenter 153
8.11 Adding your own Bayesian network learners 153
8.12 FAQ 155
8.13 Future development 156

III Data 159

9 ARFF 161
9.1 Overview 161
9.2 Examples 162
9.2.1 The ARFF Header Section 162
9.2.2 The ARFF Data Section 164
9.3 Sparse ARFF files 165

9.4 Instance weights in ARFF files 166

10 XRFF 167
10.1 File extensions 167
10.2 Comparison 167
10.2.1 ARFF 167
10.2.2 XRFF 168
10.3 Sparse format 169
10.4 Compression 170
10.5 Useful features 170
10.5.1 Class attribute specification 170
10.5.2 Attribute weights 170
10.5.3 Instance weights 171

11 Converters 173
11.1 Introduction 173
11.2 Usage 174
11.2.1 File converters 174
11.2.2 Database converters 174

12 Stemmers 177
12.1 Introduction 177
12.2 Snowball stemmers 177
12.3 Using stemmers 178
12.3.1 Commandline 178
12.3.2 StringToWordVector 178
12.4 Adding new stemmers 178

13 Databases 179
13.1 Configuration files 179
13.2 Setup 180
13.3 Missing Datatypes 181
13.4 Stored Procedures 182
13.5 Troubleshooting 183

14 Windows databases 185

IV Appendix 189

15 Research 191
15.1 Citing Weka 191
15.2 Paper references 191

16 Using the API 195
16.1 Option handling 196
16.2 Loading data 198
16.2.1 Loading data from files 198
16.2.2 Loading data from databases 199
16.3 Creating datasets in memory 202
16.3.1 Defining the format 202

16.3.2 Adding data . . . 203
16.4 Randomizing data . . . 205
16.5 Filtering . . . 206
16.5.1 Batch filtering . . . 207
16.5.2 Filtering on-the-fly . . . 208
16.6 Classification . . . 209
16.6.1 Building a classifier . . . 209
16.6.2 Evaluating a classifier . . . 211
16.6.3 Classifying instances . . . 214
16.7 Clustering . . . 216
16.7.1 Building a clusterer . . . 216
16.7.2 Evaluating a clusterer . . . 218
16.7.3 Clustering instances . . . 220
16.8 Selecting attributes . . . 221
16.8.1 Using the meta-classifier . . . 222
16.8.2 Using the filter . . . 223
16.8.3 Using the API directly . . . 224
16.9 Saving data . . . 225
16.9.1 Saving data to files . . . 225
16.9.2 Saving data to databases . . . 225
16.10 Visualization . . . 227
16.10.1 ROC curves . . . 227
16.10.2 Graphs . . . 228
16.10.2.1 Tree . . . 228
16.10.2.2 BayesNet . . . 229
16.11 Serialization . . . 230

17 Extending WEKA 233
17.1 Writing a new Classifier . . . 235
17.1.1 Choosing the base class . . . 235
17.1.2 Additional interfaces . . . 235
17.1.3 Packages . . . 236
17.1.4 Implementation . . . 237
17.1.4.1 Methods . . . 237
17.1.4.2 Guidelines . . . 241
17.2 Writing a new Filter . . . 247
17.2.1 Default approach . . . 247
17.2.1.1 Implementation . . . 247
17.2.1.2 Examples . . . 250
17.2.2 Simple approach . . . 254
17.2.2.1 SimpleBatchFilter . . . 254
17.2.2.2 SimpleStreamFilter . . . 256
17.2.2.3 Internals . . . 258
17.2.3 Capabilities . . . 258
17.2.4 Packages . . . 258
17.2.5 Revisions . . . 258
17.2.6 Testing . . . 259
17.2.6.1 Option handling . . . 259
17.2.6.2 GenericObjectEditor . . . 259
17.2.6.3 Source code . . . 259

17.2.6.4 Unit tests 259
17.3 Writing other algorithms 260
17.3.1 Clusterers 260
17.3.2 Attribute selection 262
17.3.3 Associators 264
17.4 Extending the Explorer 266
17.4.1 Adding tabs 266
17.4.1.1 Requirements 266
17.4.1.2 Examples 266
17.4.2 Adding visualization plugins 274

18 Technical documentation 277
18.1 ANT 277
18.1.1 Basics 277
18.1.2 Weka and ANT 277
18.2 CLASSPATH 278
18.2.1 Setting the CLASSPATH 278
18.2.2 RunWeka.bat 279
18.2.3 java -jar 280
18.3 Subversion 280
18.3.1 General 280
18.3.2 Source code 280
18.3.3 JUnit 281
18.3.4 Specific version 281
18.3.5 Clients 281
18.4 GenericObjectEditor 282
18.4.1 Introduction 282
18.4.2 File Structure 283
18.4.3 Exclusion 284
18.4.4 Class Discovery 285
18.4.5 Multiple Class Hierarchies 285
18.4.6 Capabilities 287
18.5 Properties 288
18.5.1 Precedence 288
18.5.2 Examples 288
18.6 XML 289
18.6.1 Command Line 289
18.6.2 Serialization of Experiments 292
18.6.3 Serialization of Classifiers 293
18.6.4 Bayesian Networks 294
18.6.5 XRFF files 294

19 Other resources 295
19.1 Mailing list 295
19.2 Troubleshooting 295
19.2.1 Weka download problems 295
19.2.2 OutOfMemoryException 295
19.2.2.1 Windows 296
19.2.3 Mac OSX 296
19.2.4 StackOverflowError 296

19.2.5 just-in-time (JIT) compiler 297
19.2.6 CSV file conversion 297
19.2.7 ARFF file doesn't load 297
19.2.8 Spaces in labels of ARFF files 297
19.2.9 CLASSPATH problems 297
19.2.10 Instance ID 298
19.2.10.1 Adding the ID 298
19.2.10.2 Removing the ID 298
19.2.11 Visualization 299
19.2.12 Memory consumption and Garbage collector 299
19.2.13 GUIChooser starts but not Experimenter or Explorer . . 299
19.2.14 KnowledgeFlow toolbars are empty 300
19.2.15 Links 300

Bibliography **302**

Part I

The Command-line

Chapter 1

A command-line primer

1.1 Introduction

While for initial experiments the included graphical user interface is quite sufficient, for in-depth usage the command line interface is recommended, because it offers some functionality which is not available via the GUI - and uses far less memory. Should you get *Out of Memory* errors, increase the maximum heap size for your java engine, usually via `-Xmx1024M` or `-Xmx1024m` for 1GB - the default setting of 16 to 64MB is usually too small. If you get errors that classes are not found, check your `CLASSPATH`: does it include `weka.jar`? You can explicitly set `CLASSPATH` via the `-cp` command line option as well.

We will begin by describing basic concepts and ideas. Then, we will describe the `weka.filters` package, which is used to transform input data, e.g. for preprocessing, transformation, feature generation and so on.

Then we will focus on the machine learning algorithms themselves. These are called Classifiers in WEKA. We will restrict ourselves to common settings for all classifiers and shortly note representatives for all main approaches in machine learning.

Afterwards, practical examples are given.

Finally, in the `doc` directory of WEKA you find a documentation of all java classes within WEKA. Prepare to use it since this overview is not intended to be complete. If you want to know exactly what is going on, take a look at the mostly well-documented source code, which can be found in `weka-src.jar` and can be extracted via the `jar` utility from the Java Development Kit (or any archive program that can handle ZIP files).

1.2 Basic concepts

1.2.1 Dataset

A set of data items, the dataset, is a very basic concept of machine learning. A dataset is roughly equivalent to a two-dimensional spreadsheet or database table. In WEKA, it is implemented by the `weka.core.Instances` class. A dataset is a collection of examples, each one of class `weka.core.Instance`. Each Instance consists of a number of attributes, any of which can be *nominal* (= one of a predefined list of values), *numeric* (= a real or integer number) or a *string* (= an arbitrary long list of characters, enclosed in "double quotes"). Additional types are *date* and *relational*, which are not covered here but in the ARFF chapter. The external representation of an Instances class is an ARFF file, which consists of a header describing the attribute types and the data as comma-separated list. Here is a short, commented example. A complete description of the ARFF file format can be found here.

```
% This is a toy example, the UCI weather dataset.
% Any relation to real weather is purely coincidental.
```

Comment lines at the beginning of the dataset should give an indication of its source, context and meaning.

```
@relation golfWeatherMichigan_1988/02/10_14days
```

Here we state the internal name of the dataset. Try to be as comprehensive as possible.

```
@attribute outlook {sunny, overcast, rainy}
@attribute windy {TRUE, FALSE}
```

Here we define two nominal attributes, *outlook* and *windy*. The former has three values: *sunny*, *overcast* and *rainy*; the latter two: *TRUE* and *FALSE*. Nominal values with special characters, commas or spaces are enclosed in 'single quotes'.

```
@attribute temperature real
@attribute humidity real
```

These lines define two numeric attributes. Instead of real, integer or numeric can also be used. While double floating point values are stored internally, only seven decimal digits are usually processed.

```
@attribute play {yes, no}
```

The last attribute is the default target or class variable used for prediction. In our case it is a nominal attribute with two values, making this a binary classification problem.

```
@data
sunny,FALSE,85,85,no
sunny,TRUE,80,90,no
overcast,FALSE,83,86,yes
rainy,FALSE,70,96,yes
rainy,FALSE,68,80,yes
```

The rest of the dataset consists of the token @data, followed by comma-separated values for the attributes – one line per example. In our case there are five examples.

In our example, we have not mentioned the attribute type string, which defines "double quoted" string attributes for text mining. In recent WEKA versions, date/time attribute types are also supported.

By default, the last attribute is considered the class/target variable, i.e. the attribute which should be predicted as a function of all other attributes. If this is not the case, specify the target variable via `-c`. The attribute numbers are one-based indices, i.e. `-c 1` specifies the first attribute.

Some basic statistics and validation of given ARFF files can be obtained via the main() routine of `weka.core.Instances`:

```
java weka.core.Instances data/soybean.arff
```

`weka.core` offers some other useful routines, e.g. `converters.C45Loader` and `converters.CSVLoader`, which can be used to import C45 datasets and comma/tab-separated datasets respectively, e.g.:

```
java weka.core.converters.CSVLoader data.csv > data.arff
java weka.core.converters.C45Loader c45_filestem > data.arff
```

1.2.2 Classifier

Any learning algorithm in WEKA is derived from the abstract `weka.classifiers.Classifier` class. Surprisingly little is needed for a basic classifier: a routine which generates a classifier model from a training dataset (= `buildClassifier`) and another routine which evaluates the generated model on an unseen test dataset (= `classifyInstance`), or generates a probability distribution for all classes (= `distributionForInstance`).

A classifier model is an arbitrary complex mapping from all-but-one dataset attributes to the class attribute. The specific form and creation of this mapping, or model, differs from classifier to classifier. For example, ZeroR's (= `weka.classifiers.rules.ZeroR`) model just consists of a single value: the most common class, or the median of all numeric values in case of predicting a numeric value (= regression learning). ZeroR is a trivial classifier, but it gives a lower bound on the performance of a given dataset which should be significantly improved by more complex classifiers. As such it is a reasonable test on how well the class can be predicted without considering the other attributes.

Later, we will explain how to interpret the output from classifiers in detail – for now just focus on the *Correctly Classified Instances* in the section *Stratified cross-validation* and notice how it improves from ZeroR to J48:

```
java weka.classifiers.rules.ZeroR -t weather.arff
java weka.classifiers.trees.J48 -t weather.arff
```

There are various approaches to determine the performance of classifiers. The performance can most simply be measured by counting the proportion of correctly predicted examples in an unseen test dataset. This value is the *accuracy*, which is also *1-ErrorRate*. Both terms are used in literature.

The simplest case is using a training set and a test set which are mutually independent. This is referred to as hold-out estimate. To estimate variance in these performance estimates, hold-out estimates may be computed by repeatedly resampling the same dataset – i.e. randomly reordering it and then splitting it into training and test sets with a specific proportion of the examples, collecting all estimates on test data and computing average and standard deviation of accuracy.

A more elaborate method is cross-validation. Here, a number of folds n is specified. The dataset is randomly reordered and then split into n folds of equal size. In each iteration, one fold is used for testing and the other n-1 folds are used for training the classifier. The test results are collected and averaged over all folds. This gives the cross-validation estimate of the accuracy. The folds can be purely random or slightly modified to create the same class distributions in each fold as in the complete dataset. In the latter case the cross-validation is called *stratified*. Leave-one-out (= loo) cross-validation signifies that n is equal to the number of examples. Out of necessity, loo cv has to be non-stratified, i.e. the class distributions in the test set are not related to those in the training data. Therefore loo cv tends to give less reliable results. However it is still quite useful in dealing with small datasets since it utilizes the greatest amount of training data from the dataset.

1.2.3 weka.filters

The `weka.filters` package is concerned with classes that transform datasets – by removing or adding attributes, resampling the dataset, removing examples and so on. This package offers useful support for data preprocessing, which is an important step in machine learning.

All filters offer the options `-i` for specifying the input dataset, and `-o` for specifying the output dataset. If any of these parameters is not given, standard input and/or standard output will be read from/written to. Other parameters are specific to each filter and can be found out via *-h*, as with any other class. The `weka.filters` package is organized into `supervised` and `unsupervised` filtering, both of which are again subdivided into instance and attribute filtering. We will discuss each of the four subsections separately.

weka.filters.supervised

Classes below `weka.filters.supervised` in the class hierarchy are for supervised filtering, i.e., taking advantage of the class information. A class must be assigned via `-c`, for WEKA default behaviour use `-c last`.

weka.filters.supervised.attribute

`Discretize` is used to discretize numeric attributes into nominal ones, based on the class information, via Fayyad & Irani's MDL method, or optionally with Kononeko's MDL method. At least some learning schemes or classifiers can only process nominal data, e.g. `weka.classifiers.rules.Prism`; in some cases discretization may also reduce learning time.

```
java weka.filters.supervised.attribute.Discretize -i data/iris.arff \
  -o iris-nom.arff -c last
java weka.filters.supervised.attribute.Discretize -i data/cpu.arff \
  -o cpu-classvendor-nom.arff -c first
```

`NominalToBinary` encodes all nominal attributes into binary (two-valued) attributes, which can be used to transform the dataset into a purely numeric representation, e.g. for visualization via multi-dimensional scaling.

```
java weka.filters.supervised.attribute.NominalToBinary \
  -i data/contact-lenses.arff -o contact-lenses-bin.arff -c last
```

Keep in mind that most classifiers in WEKA utilize transformation filters internally, e.g. Logistic and SMO, so you will usually not have to use these filters explicity. However, if you plan to run a lot of experiments, pre-applying the filters yourself may improve runtime performance.

weka.filters.supervised.instance

`Resample` creates a stratified subsample of the given dataset. This means that overall class distributions are approximately retained within the sample. A bias towards uniform class distribution can be specified via `-B`.

```
java weka.filters.supervised.instance.Resample -i data/soybean.arff \
  -o soybean-5%.arff -c last -Z 5
java weka.filters.supervised.instance.Resample -i data/soybean.arff \
  -o soybean-uniform-5%.arff -c last -Z 5 -B 1
```

`StratifiedRemoveFolds` creates stratified cross-validation folds of the given dataset. This means that by default the class distributions are approximately retained within each fold. The following example splits soybean.arff into stratified training and test datasets, the latter consisting of 25% (= 1/4) of the data.

```
java weka.filters.supervised.instance.StratifiedRemoveFolds \
  -i data/soybean.arff -o soybean-train.arff \
  -c last -N 4 -F 1 -V
java weka.filters.supervised.instance.StratifiedRemoveFolds \
  -i data/soybean.arff -o soybean-test.arff \
  -c last -N 4 -F 1
```

weka.filters.unsupervised

Classes below `weka.filters.unsupervised` in the class hierarchy are for unsupervised filtering, e.g. the non-stratified version of Resample. A class attribute should not be assigned here.

weka.filters.unsupervised.attribute

`StringToWordVector` transforms string attributes into word vectors, i.e. creating one attribute for each word which either encodes presence or word count (= `-C`) within the string. `-W` can be used to set an approximate limit on the number of words. When a class is assigned, the limit applies to each class separately. This filter is useful for text mining.
`Obfuscate` renames the dataset name, all attribute names and nominal attribute values. This is intended for exchanging sensitive datasets without giving away restricted information.
`Remove` is intended for explicit deletion of attributes from a dataset, e.g. for removing attributes of the iris dataset:

```
java weka.filters.unsupervised.attribute.Remove -R 1-2 \
  -i data/iris.arff -o iris-simplified.arff
java weka.filters.unsupervised.attribute.Remove -V -R 3-last \
  -i data/iris.arff -o iris-simplified.arff
```

weka.filters.unsupervised.instance

`Resample` creates a non-stratified subsample of the given dataset, i.e. random sampling without regard to the class information. Otherwise it is equivalent to its supervised variant.

```
java weka.filters.unsupervised.instance.Resample -i data/soybean.arff \
  -o soybean-5%.arff -Z 5
```

`RemoveFolds`creates cross-validation folds of the given dataset. The class distributions are not retained. The following example splits soybean.arff into training and test datasets, the latter consisting of 25% (= 1/4) of the data.

```
java weka.filters.unsupervised.instance.RemoveFolds -i data/soybean.arff \
  -o soybean-train.arff -c last -N 4 -F 1 -V
java weka.filters.unsupervised.instance.RemoveFolds -i data/soybean.arff \
  -o soybean-test.arff -c last -N 4 -F 1
```

`RemoveWithValues` filters instances according to the value of an attribute.

```
java weka.filters.unsupervised.instance.RemoveWithValues -i data/soybean.arff \
  -o soybean-without_herbicide_injury.arff -V -C last -L 19
```

1.2.4 weka.classifiers

Classifiers are at the core of WEKA. There are a lot of common options for classifiers, most of which are related to evaluation purposes. We will focus on the most important ones. All others including classifier-specific parameters can be found via `-h`, as usual.

-t	specifies the training file (ARFF format)
-T	specifies the test file in (ARFF format). If this parameter is missing, a crossvalidation will be performed (default: ten-fold cv)
-x	This parameter determines the number of folds for the cross-validation. A cv will only be performed if -T is missing.
-c	As we already know from the weka.filters section, this parameter sets the class variable with a one-based index.
-d	The model after training can be saved via this parameter. Each classifier has a different binary format for the model, so it can only be read back by the exact same classifier on a compatible dataset. Only the model on the training set is saved, not the multiple models generated via cross-validation.
-l	Loads a previously saved model, usually for testing on new, previously unseen data. In that case, a compatible test file should be specified, i.e. the same attributes in the same order.
-p #	If a test file is specified, this parameter shows you the predictions and one attribute (0 for none) for all test instances.
-i	A more detailed performance description via precision, recall, true- and false positive rate is additionally output with this parameter. All these values can also be computed from the confusion matrix.
-o	This parameter switches the human-readable output of the model description off. In case of support vector machines or NaiveBayes, this makes some sense unless you want to parse and visualize a lot of information.

We now give a short list of selected classifiers in WEKA. Other classifiers below weka.classifiers may also be used. This is more easy to see in the Explorer GUI.

- `trees.J48` A clone of the C4.5 decision tree learner
- `bayes.NaiveBayes` A Naive Bayesian learner. `-K` switches on kernel density estimation for numerical attributes which often improves performance.
- `meta.ClassificationViaRegression -W functions.LinearRegression` Multi-response linear regression.
- `functions.Logistic` Logistic Regression.

- `functions.SMO` Support Vector Machine (linear, polynomial and RBF kernel) with Sequential Minimal Optimization Algorithm due to [4]. Defaults to SVM with linear kernel, `-E 5 -C 10` gives an SVM with polynomial kernel of degree *5* and lambda of *10*.

- `lazy.KStar` Instance-Based learner. `-E` sets the blend entropy automatically, which is usually preferable.

- `lazy.IBk` Instance-Based learner with fixed neighborhood. `-K` sets the number of neighbors to use. `IB1` is equivalent to `IBk -K 1`

- `rules.JRip` A clone of the RIPPER rule learner.

Based on a simple example, we will now explain the output of a typical classifier, `weka.classifiers.trees.J48`. Consider the following call from the command line, or start the WEKA explorer and train J48 on *weather.arff*:

```
java weka.classifiers.trees.J48 -t data/weather.arff -i
```

```
J48 pruned tree
------------------

outlook = sunny
|   humidity <= 75: yes (2.0)
|   humidity > 75: no (3.0)
outlook = overcast: yes (4.0)
outlook = rainy
|   windy = TRUE: no (2.0)
|   windy = FALSE: yes (3.0)

Number of Leaves  :  5

Size of the tree :  8
```

The first part, unless you specify `-o`, is a human-readable form of the training set model. In this case, it is a decision tree. *outlook* is at the root of the tree and determines the first decision. In case it is overcast, we'll always play golf. The numbers in (parentheses) at the end of each leaf tell us the number of examples in this leaf. If one or more leaves were not pure (= all of the same class), the number of misclassified examples would also be given, after a /slash/

```
Time taken to build model: 0.05 seconds
Time taken to test model on training data: 0 seconds
```

As you can see, a decision tree learns quite fast and is evaluated even faster. E.g. for a lazy learner, testing would take far longer than training.

```
== Error on training data ===

Correctly Classified Instance      14      100 %
Incorrectly Classified Instances    0      0 %
Kappa statistic                     1
Mean absolute error                 0
Root mean squared error             0
Relative absolute error             0      %
Root relative squared error         0      %
Total Number of Instances          14

=== Detailed Accuracy By Class ===

TP Rate  FP Rate  Precision  Recall  F-Measure  Class
1        0        1          1       1          yes
1        0        1          1       1          no

=== Confusion Matrix ===

a b   <-- classified as
9 0 | a = yes
0 5 | b = no
```

This is quite boring: our classifier is perfect, at least on the training data – all instances were classified correctly and all errors are zero. As is usually the case, the training set accuracy is too optimistic. The detailed accuracy by class, which is output via `-i`, and the confusion matrix is similarily trivial.

```
=== Stratified cross-validation ===

Correctly Classified Instances      9      64.2857 %
Incorrectly Classified Instances    5      35.7143 %
Kappa statistic                     0.186
Mean absolute error                 0.2857
Root mean squared error             0.4818
Relative absolute error            60      %
Root relative squared error        97.6586 %
Total Number of Instances          14


=== Detailed Accuracy By Class ===

TP Rate  FP Rate  Precision  Recall  F-Measure  Class
0.778    0.6      0.7        0.778   0.737      yes
0.4      0.222    0.5        0.4     0.444      no


=== Confusion Matrix ===

a b   <-- classified as
7 2 | a = yes
3 2 | b = no
```

The stratified cv paints a more realistic picture. The accuracy is around 64%. The kappa statistic measures the agreement of prediction with the true class – 1.0 signifies complete agreement. The following error values are not very meaningful for classification tasks, however for regression tasks e.g. the root of the mean squared error per example would be a reasonable criterion. We will discuss the relation between confusion matrix and other measures in the text.

The confusion matrix is more commonly named *contingency table*. In our case we have two classes, and therefore a 2x2 confusion matrix, the matrix could be arbitrarily large. The number of correctly classified instances is the sum of diagonals in the matrix; all others are incorrectly classified (class "a" gets misclassified as "b" exactly twice, and class "b" gets misclassified as "a" three times).

The *True Positive (TP)* rate is the proportion of examples which were classified as class x, among all examples which truly have class x, i.e. how much part of the class was captured. It is equivalent to Recall. In the confusion matrix, this is the diagonal element divided by the sum over the relevant row, i.e. 7/(7+2)=0.778 for class yes and 2/(3+2)=0.4 for class no in our example.

The *False Positive (FP)* rate is the proportion of examples which were classified as class x, but belong to a different class, among all examples which are not of class x. In the matrix, this is the column sum of class x minus the diagonal element, divided by the rows sums of all other classes; i.e. 3/5=0.6 for class yes and 2/9=0.222 for class no.

The *Precision* is the proportion of the examples which truly have class x

among all those which were classified as class x. In the matrix, this is the diagonal element divided by the sum over the relevant column, i.e. 7/(7+3)=0.7 for class yes and 2/(2+2)=0.5 for class no.

The *F-Measure* is simply 2*Precision*Recall/(Precision+Recall), a combined measure for precision and recall.

These measures are useful for comparing classifiers. However, if more detailed information about the classifier's predictions are necessary, `-p #` outputs just the predictions for each test instance, along with a range of one-based attribute ids (0 for none). Let's look at the following example. We shall assume soybean-train.arff and soybean-test.arff have been constructed via weka.filters.supervised.instance.StratifiedRemoveFolds as in a previous example.

```
java weka.classifiers.bayes.NaiveBayes -K -t soybean-train.arff \
  -T soybean-test.arff -p 0
```

```
0 diaporthe-stem-canker 0.9999672587892333 diaporthe-stem-canker
1 diaporthe-stem-canker 0.9999992614503429 diaporthe-stem-canker
2 diaporthe-stem-canker 0.999998948559035 diaporthe-stem-canker
3 diaporthe-stem-canker 0.9999998441238833 diaporthe-stem-canker
4 diaporthe-stem-canker 0.9999989997681132 diaporthe-stem-canker
5 rhizoctonia-root-rot 0.9999999395928124 rhizoctonia-root-rot
6 rhizoctonia-root-rot 0.999998912860593 rhizoctonia-root-rot
7 rhizoctonia-root-rot 0.9999994386283236 rhizoctonia-root-rot
...
```

The values in each line are separated by a single space. The fields are the zero-based test instance id, followed by the predicted class value, the confidence for the prediction (estimated probability of predicted class), and the true class. All these are correctly classified, so let's look at a few erroneous ones.

```
32 phyllosticta-leaf-spot 0.7789710144361445 brown-spot
...
39 alternarialeaf-spot 0.6403333824349896 brown-spot
...
44 phyllosticta-leaf-spot 0.893568420641914 brown-spot
...
46 alternarialeaf-spot 0.5788190397739439 brown-spot
...
73 brown-spot 0.4943768155314637 alternarialeaf-spot
...
```

In each of these cases, a misclassification occurred, mostly between classes *alternarialeaf-spot* and *brown-spot*. The confidences seem to be lower than for correct classification, so for a real-life application it may make sense to output *don't know* below a certain threshold. WEKA also outputs a trailing newline.

If we had chosen a range of attributes via `-p`, e.g. `-p first-last`, the mentioned attributes would have been output afterwards as comma-separated values, in (parentheses). However, the zero-based instance id in the first column offers a safer way to determine the test instances.

If we had saved the output of `-p` in *soybean-test.preds*, the following call would compute the number of correctly classified instances:

```
cat soybean-test.preds | awk '$2==$4&&$0!=""' | wc -l
```

Dividing by the number of instances in the test set, i.e. `wc -l < soybean-test.preds` minus one (= trailing newline), we get the training set accuracy.

1.3 Examples

Usually, if you evaluate a classifier for a longer experiment, you will do something like this (for `csh`):

```
java -Xmx1024m weka.classifiers.trees.J48 -t data.arff -i -k \
  -d J48-data.model >&! J48-data.out &
```

The `-Xmx1024m` parameter for maximum heap size ensures your task will get enough memory. There is no overhead involved, it just leaves more room for the heap to grow. `-i` and `-k` gives you some additional information, which may be useful, e.g. precision and recall for all classes. In case your model performs well, it makes sense to save it via `-d` - you can always delete it later! The implicit cross-validation gives a more reasonable estimate of the expected accuracy on unseen data than the training set accuracy. The output both of standard error and output should be redirected, so you get both errors and the normal output of your classifier. The last `&` starts the task in the background. Keep an eye on your task via top and if you notice the hard disk works hard all the time (for linux), this probably means your task needs too much memory and will not finish in time for the exam. In that case, switch to a faster classifier or use filters, e.g. for `Resample` to reduce the size of your dataset or `StratifiedRemoveFolds` to create training and test sets - for most classifiers, training takes more time than testing.

So, now you have run a lot of experiments – which classifier is best? Try

```
cat *.out | grep -A 3 "Stratified" | grep "^Correctly"
```

...this should give you all cross-validated accuracies. If the cross-validated accuracy is roughly the same as the training set accuracy, this indicates that your classifiers is presumably not overfitting the training set.

Now you have found the best classifier. To apply it on a new dataset, use e.g.

```
java weka.classifiers.trees.J48 -l J48-data.model -T new-data.arff
```

You will have to use the same classifier to load the model, but you need not set any options. Just add the new test file via `-T`. If you want, `-p first-last` will output all test instances with classifications and confidence, followed by all attribute values, so you can look at each error separately.

The following more complex csh script creates datasets for learning curves, i.e. creating a 75% training set and 25% test set from a given dataset, then successively reducing the test set by factor 1.2 (83%), until it is also 25% in size. All this is repeated thirty times, with different random reorderings (-S) and the results are written to different directories. The Experimenter GUI in WEKA can be used to design and run similar experiments.

```
#!/bin/csh
foreach f ($*)
  set run=1
  while ( $run <= 30 )
    mkdir $run >&! /dev/null
    java weka.filters.supervised.instance.StratifiedRemoveFolds -N 4 -F 1 -S $run -c last -i ../$f -o $run/t_$f
    java weka.filters.supervised.instance.StratifiedRemoveFolds -N 4 -F 1 -S $run -V -c last -i ../$f -o $run/t0$f
    foreach nr (0 1 2 3 4 5)
      set nrp1=$nr
      @ nrp1++
```

```
    java weka.filters.supervised.instance.Resample -S 0 -Z 83 -c last -i $run/t$nr$f -o $run/t$nrp1$f
  end

  echo Run $run of $f done.
  @ run++
 end
end
```

If meta classifiers are used, i.e. classifiers whose options include classifier specifications - for example, `StackingC` or `ClassificationViaRegression`, care must be taken not to mix the parameters. E.g.:

```
java weka.classifiers.meta.ClassificationViaRegression \
  -W weka.classifiers.functions.LinearRegression -S 1 \
  -t data/iris.arff -x 2
```

gives us an illegal options exception for `-S 1`. This parameter is meant for `LinearRegression`, not for `ClassificationViaRegression`, but WEKA does not know this by itself. One way to clarify this situation is to enclose the classifier specification, including all parameters, in "double" quotes, like this:

```
java weka.classifiers.meta.ClassificationViaRegression \
  -W "weka.classifiers.functions.LinearRegression -S 1" \
  -t data/iris.arff -x 2
```

However this does not always work, depending on how the option handling was implemented in the top-level classifier. While for `Stacking` this approach would work quite well, for `ClassificationViaRegression` it does not. We get the dubious error message that the class *weka.classifiers.functions.LinearRegression -S 1* cannot be found. Fortunately, there is another approach: All parameters given after `--` are processed by the first sub-classifier; another `--` lets us specify parameters for the second sub-classifier and so on.

```
java weka.classifiers.meta.ClassificationViaRegression \
  -W weka.classifiers.functions.LinearRegression \
  -t data/iris.arff -x 2 -- -S 1
```

In some cases, both approaches have to be mixed, for example:

```
java weka.classifiers.meta.Stacking -B "weka.classifiers.lazy.IBk -K 10" \
  -M "weka.classifiers.meta.ClassificationViaRegression -W weka.classifiers.functions.LinearRegression -- -S 1" \
  -t data/iris.arff -x 2
```

Notice that while `ClassificationViaRegression` honors the `--` parameter, `Stacking` itself does not. Sadly the option handling for sub-classifier specifications is not yet completely unified within WEKA, but hopefully one or the other approach mentioned here will work.

Part II

The Graphical User Interface

Chapter 2

Launching WEKA

The Weka GUI Chooser (class `weka.gui.GUIChooser`) provides a starting point for launching Weka's main GUI applications and supporting tools. If one prefers a MDI ("multiple document interface") appearance, then this is provided by an alternative launcher called "Main" (class `weka.gui.Main`).

The GUI Chooser consists of four buttons—one for each of the four major Weka applications—and four menus.

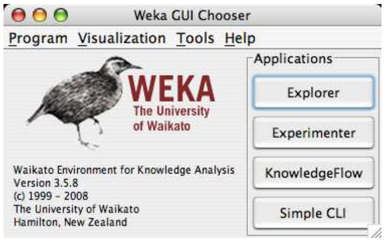

The buttons can be used to start the following applications:

- **Explorer** An environment for exploring data with WEKA (the rest of this documentation deals with this application in more detail).
- **Experimenter** An environment for performing experiments and conducting statistical tests between learning schemes.
- **KnowledgeFlow** This environment supports essentially the same functions as the Explorer but with a drag-and-drop interface. One advantage is that it supports incremental learning.
- **SimpleCLI** Provides a simple command-line interface that allows direct execution of WEKA commands for operating systems that do not provide their own command line interface.

The menu consists of four sections:

1. **Program**

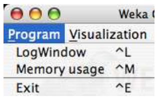

- **LogWindow** Opens a log window that captures all that is printed to *stdout* or *stderr*. Useful for environments like MS Windows, where WEKA is normally not started from a terminal.
- **Exit** Closes WEKA.

2. **Tools** Other useful applications.

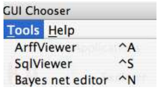

- **ArffViewer** An MDI application for viewing ARFF files in spreadsheet format.
- **SqlViewer** Represents an SQL worksheet, for querying databases via JDBC.
- **Bayes net editor** An application for editing, visualizing and learning Bayes nets.

3. **Visualization** Ways of visualizing data with WEKA.

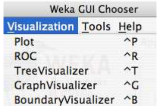

- **Plot** For plotting a 2D plot of a dataset.
- **ROC** Displays a previously saved ROC curve.
- **TreeVisualizer** For displaying directed graphs, e.g., a decision tree.
- **GraphVisualizer** Visualizes XML BIF or DOT format graphs, e.g., for Bayesian networks.
- **BoundaryVisualizer** Allows the visualization of classifier decision boundaries in two dimensions.

4. **Help** Online resources for WEKA can be found here.

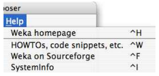

- **Weka homepage** Opens a browser window with WEKA's homepage.
- **HOWTOs, code snippets, etc.** The general WekaWiki [2], containing lots of examples and HOWTOs around the development and use of WEKA.
- **Weka on Sourceforge** WEKA's project homepage on Sourceforge.net.
- **SystemInfo** Lists some internals about the Java/WEKA environment, e.g., the `CLASSPATH`.

To make it easy for the user to add new functionality to the menu without having to modify the code of WEKA itself, the GUI now offers a plugin mechanism for such add-ons. Due to the inherent dynamic class discovery, plugins only need to implement the `weka.gui.MainMenuExtension` interface and

WEKA notified of the package they reside in to be displayed in the menu under "Extensions" (this extra menu appears automatically as soon as extensions are discovered). More details can be found in the Wiki article "Extensions for Weka's main GUI" [6].

If you launch WEKA from a terminal window, some text begins scrolling in the terminal. Ignore this text unless something goes wrong, in which case it can help in tracking down the cause (the *LogWindow* from the *Program* menu displays that information as well).

This User Manual focuses on using the Explorer but does not explain the individual data preprocessing tools and learning algorithms in WEKA. For more information on the various filters and learning methods in WEKA, see the book *Data Mining* [1].

Chapter 3

Simple CLI

The Simple CLI provides full access to all Weka classes, i.e., classifiers, filters, clusterers, etc., but without the hassle of the CLASSPATH (it facilitates the one, with which Weka was started).

It offers a simple *Weka shell* with separated commandline and output.

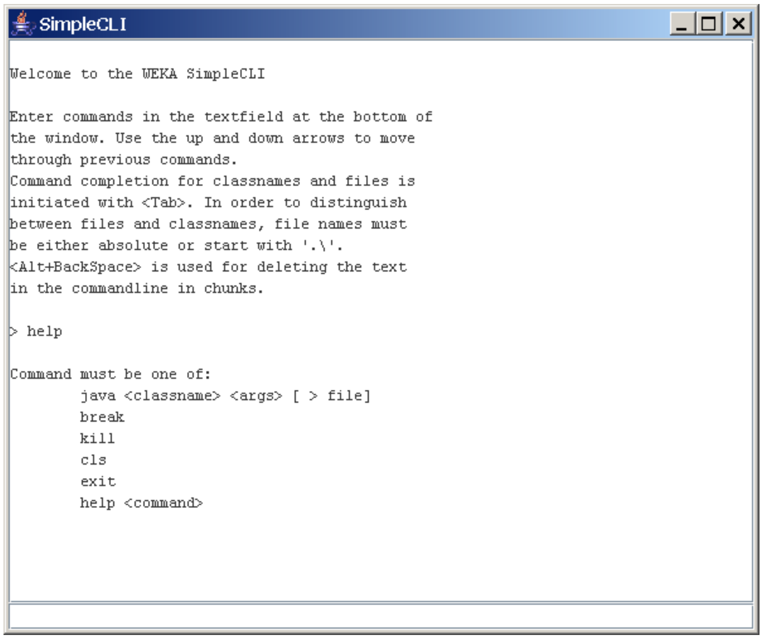

3.1 Commands

The following commands are available in the Simple CLI:

- `java <classname> [<args>]`
  invokes a java class with the given arguments (if any)

- `break`
  stops the current thread, e.g., a running classifier, in a friendly manner

- `kill`
  stops the current thread in an unfriendly fashion

- `cls`
  clears the output area

- `exit`
  exits the Simple CLI

- `help [<command>]`
  provides an overview of the available commands if without a command name as argument, otherwise more help on the specified command

3.2 Invocation

In order to invoke a Weka class, one has only to prefix the class with "java". This command tells the Simple CLI to load a class and execute it with any given parameters. E.g., the J48 classifier can be invoked on the iris dataset with the following command:

```
java weka.classifiers.trees.J48 -t c:/temp/iris.arff
```

This results in the following output:

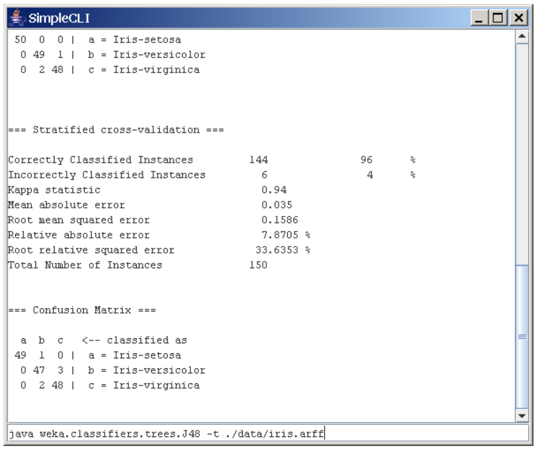

3.3 Command redirection

Starting with this version of Weka one can perform a basic redirection:

```
java weka.classifiers.trees.J48 -t test.arff > j48.txt
```

Note: the `>` must be preceded and followed by a space, otherwise it is not recognized as redirection, but part of another parameter.

3.4 Command completion

Commands starting with java support completion for classnames and filenames via Tab (`Alt+BackSpace` deletes parts of the command again). In case that there are several matches, Weka lists all possible matches.

- **package name completion**

  ```
  java weka.cl<Tab>
  ```

  results in the following output of possible matches of package names:

  ```
  Possible matches:
    weka.classifiers
    weka.clusterers
  ```

- **classname completion**

  ```
  java weka.classifiers.meta.A<Tab>
  ```

  lists the following classes

  ```
  Possible matches:
    weka.classifiers.meta.AdaBoostM1
    weka.classifiers.meta.AdditiveRegression
    weka.classifiers.meta.AttributeSelectedClassifier
  ```

- **filename completion**
  In order for Weka to determine whether a the string under the cursor is a classname or a filename, filenames need to be absolute (Unix/Linx: `/some/path/file`; Windows: C:\Some\Path\file) or relative and starting with a dot (Unix/Linux: `./some/other/path/file`; Windows: .\Some\Other\Path\file).

Chapter 4

Explorer

4.1 The user interface

4.1.1 Section Tabs

At the very top of the window, just below the title bar, is a row of tabs. When the Explorer is first started only the first tab is active; the others are greyed out. This is because it is necessary to open (and potentially pre-process) a data set before starting to explore the data.

The tabs are as follows:

1. **Preprocess**. Choose and modify the data being acted on.
2. **Classify**. Train and test learning schemes that classify or perform regression.
3. **Cluster**. Learn clusters for the data.
4. **Associate**. Learn association rules for the data.
5. **Select attributes**. Select the most relevant attributes in the data.
6. **Visualize**. View an interactive 2D plot of the data.

Once the tabs are active, clicking on them flicks between different screens, on which the respective actions can be performed. The bottom area of the window (including the status box, the log button, and the Weka bird) stays visible regardless of which section you are in.

The Explorer can be easily extended with custom tabs. The Wiki article "Adding tabs in the Explorer" [7] explains this in detail.

4.1.2 Status Box

The status box appears at the very bottom of the window. It displays messages that keep you informed about what's going on. For example, if the Explorer is busy loading a file, the status box will say that.

TIP—right-clicking the mouse anywhere inside the status box brings up a little menu. The menu gives two options:

1. **Memory information**. Display in the log box the amount of memory available to WEKA.

2. **Run garbage collector**. Force the Java garbage collector to search for memory that is no longer needed and free it up, allowing more memory for new tasks. Note that the garbage collector is constantly running as a background task anyway.

4.1.3 Log Button

Clicking on this button brings up a separate window containing a scrollable text field. Each line of text is stamped with the time it was entered into the log. As you perform actions in WEKA, the log keeps a record of what has happened. For people using the command line or the SimpleCLI, the log now also contains the full setup strings for classification, clustering, attribute selection, etc., so that it is possible to copy/paste them elsewhere. Options for dataset(s) and, if applicable, the class attribute still have to be provided by the user (e.g., `-t` for classifiers or `-i` and `-o` for filters).

4.1.4 WEKA Status Icon

To the right of the status box is the WEKA status icon. When no processes are running, the bird sits down and takes a nap. The number beside the × symbol gives the number of concurrent processes running. When the system is idle it is zero, but it increases as the number of processes increases. When any process is started, the bird gets up and starts moving around. If it's standing but stops moving for a long time, it's sick: something has gone wrong! In that case you should restart the WEKA Explorer.

4.1.5 Graphical output

Most graphical displays in WEKA, e.g., the GraphVisualizer or the TreeVisualizer, support saving the output to a file. A dialog for saving the output can be brought up with *Alt+Shift+left-click*. Supported formats are currently Windows Bitmap, JPEG, PNG and EPS (encapsulated Postscript). The dialog also allows you to specify the dimensions of the generated image.

4.2 Preprocessing

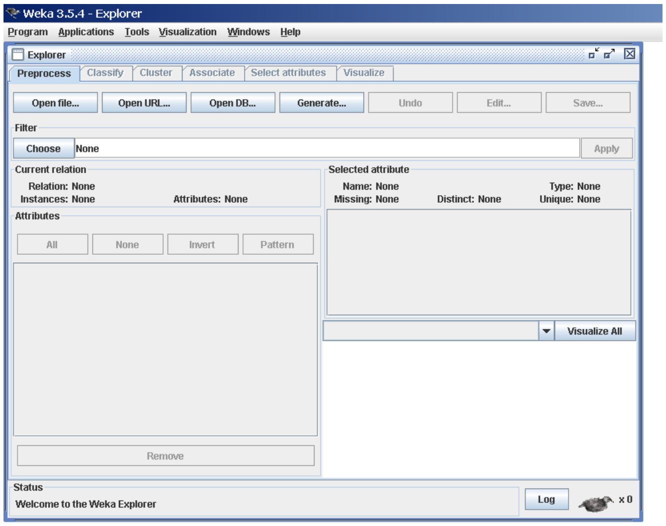

4.2.1 Loading Data

The first four buttons at the top of the preprocess section enable you to load data into WEKA:

1. **Open file....** Brings up a dialog box allowing you to browse for the data file on the local file system.

2. **Open URL....** Asks for a Uniform Resource Locator address for where the data is stored.

3. **Open DB....** Reads data from a database. (Note that to make this work you might have to edit the file in weka/experiment/DatabaseUtils.props.)

4. **Generate....** Enables you to generate artificial data from a variety of DataGenerators.

Using the **Open file...** button you can read files in a variety of formats: WEKA's ARFF format, CSV format, C4.5 format, or serialized Instances format. ARFF files typically have a *.arff* extension, CSV files a *.csv* extension, C4.5 files a *.data* and *.names* extension, and serialized Instances objects a *.bsi* extension.

NB: This list of formats can be extended by adding custom file converters to the `weka.core.converters` package.

4.2.2 The Current Relation

Once some data has been loaded, the Preprocess panel shows a variety of information. The **Current relation** box (the "current relation" is the currently loaded data, which can be interpreted as a single relational table in database terminology) has three entries:

1. **Relation**. The name of the relation, as given in the file it was loaded from. Filters (described below) modify the name of a relation.

2. **Instances**. The number of instances (data points/records) in the data.

3. **Attributes**. The number of attributes (features) in the data.

4.2.3 Working With Attributes

Below the **Current relation** box is a box titled **Attributes**. There are four buttons, and beneath them is a list of the attributes in the current relation. The list has three columns:

1. **No.**. A number that identifies the attribute in the order they are specified in the data file.

2. **Selection tick boxes**. These allow you select which attributes are present in the relation.

3. **Name**. The name of the attribute, as it was declared in the data file.

When you click on different rows in the list of attributes, the fields change in the box to the right titled **Selected attribute**. This box displays the characteristics of the currently highlighted attribute in the list:

1. **Name**. The name of the attribute, the same as that given in the attribute list.

2. **Type**. The type of attribute, most commonly Nominal or Numeric.

3. **Missing**. The number (and percentage) of instances in the data for which this attribute is missing (unspecified).

4. **Distinct**. The number of different values that the data contains for this attribute.

5. **Unique**. The number (and percentage) of instances in the data having a value for this attribute that no other instances have.

Below these statistics is a list showing more information about the values stored in this attribute, which differ depending on its type. If the attribute is nominal, the list consists of each possible value for the attribute along with the number of instances that have that value. If the attribute is numeric, the list gives four statistics describing the distribution of values in the data—the minimum, maximum, mean and standard deviation. And below these statistics there is a coloured histogram, colour-coded according to the attribute chosen as the *Class* using the box above the histogram. (This box will bring up a drop-down list of available selections when clicked.) Note that only nominal *Class* attributes will result in a colour-coding. Finally, after pressing the **Visualize All** button, histograms for all the attributes in the data are shown in a separate window.

Returning to the attribute list, to begin with all the tick boxes are unticked. They can be toggled on/off by clicking on them individually. The four buttons above can also be used to change the selection:

1. **All**. All boxes are ticked.

2. **None**. All boxes are cleared (unticked).

3. **Invert**. Boxes that are ticked become unticked and *vice versa*.

4. **Pattern**. Enables the user to select attributes based on a Perl 5 Regular Expression. E.g., `.*_id` selects all attributes which name ends with `_id`.

Once the desired attributes have been selected, they can be removed by clicking the **Remove** button below the list of attributes. Note that this can be undone by clicking the **Undo** button, which is located next to the **Edit** button in the top-right corner of the Preprocess panel.

4.2.4 Working With Filters

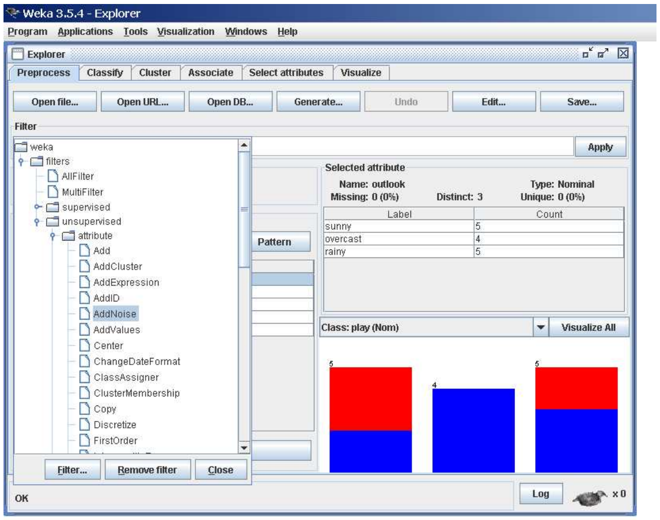

The preprocess section allows filters to be defined that transform the data in various ways. The **Filter** box is used to set up the filters that are required. At the left of the **Filter** box is a **Choose** button. By clicking this button it is possible to select one of the filters in WEKA. Once a filter has been selected, its name and options are shown in the field next to the **Choose** button. Clicking on this box with the *left* mouse button brings up a GenericObjectEditor dialog box. A click with the *right* mouse button (or *Alt+Shift+left click*) brings up a menu where you can choose, either to display the properties in a GenericObjectEditor dialog box, or to copy the current setup string to the clipboard.

The GenericObjectEditor Dialog Box

The GenericObjectEditor dialog box lets you configure a filter. The same kind of dialog box is used to configure other objects, such as classifiers and clusterers (see below). The fields in the window reflect the available options.

Right-clicking (or *Alt+Shift+Left-Click*) on such a field will bring up a popup menu, listing the following options:

1. **Show properties...** has the same effect as left-clicking on the field, i.e., a dialog appears allowing you to alter the settings.

2. **Copy configuration to clipboard** copies the currently displayed configuration string to the system's clipboard and therefore can be used anywhere else in WEKA or in the console. This is rather handy if you have to setup complicated, nested schemes.

3. **Enter configuration...** is the "receiving" end for configurations that got copied to the clipboard earlier on. In this dialog you can enter a classname followed by options (if the class supports these). This also allows you to transfer a filter setting from the Preprocess panel to a `FilteredClassifier` used in the Classify panel.

Left-Clicking on any of these gives an opportunity to alter the filters settings. For example, the setting may take a text string, in which case you type the string into the text field provided. Or it may give a drop-down box listing several states to choose from. Or it may do something else, depending on the information required. Information on the options is provided in a tool tip if you let the mouse pointer hover of the corresponding field. More information on the filter and its options can be obtained by clicking on the **More** button in the **About** panel at the top of the GenericObjectEditor window.

Some objects display a brief description of what they do in an **About** box, along with a **More** button. Clicking on the **More** button brings up a window describing what the different options do. Others have an additional button, *Capabilities*, which lists the types of attributes and classes the object can handle.

At the bottom of the GenericObjectEditor dialog are four buttons. The first two, **Open...** and **Save...** allow object configurations to be stored for future use. The **Cancel** button backs out without remembering any changes that have been made. Once you are happy with the object and settings you have chosen, click **OK** to return to the main Explorer window.

Applying Filters

Once you have selected and configured a filter, you can apply it to the data by pressing the **Apply** button at the right end of the **Filter** panel in the Preprocess panel. The Preprocess panel will then show the transformed data. The change can be undone by pressing the **Undo** button. You can also use the **Edit...** button to modify your data manually in a dataset editor. Finally, the **Save...** button at the top right of the Preprocess panel saves the current version of the relation in file formats that can represent the relation, allowing it to be kept for future use.

Note: Some of the filters behave differently depending on whether a class attribute has been set or not (using the box above the histogram, which will bring up a drop-down list of possible selections when clicked). In particular, the "supervised filters" require a class attribute to be set, and some of the "unsupervised attribute filters" will skip the class attribute if one is set. Note that it is also possible to set *Class* to *None*, in which case no class is set.

4.3 Classification

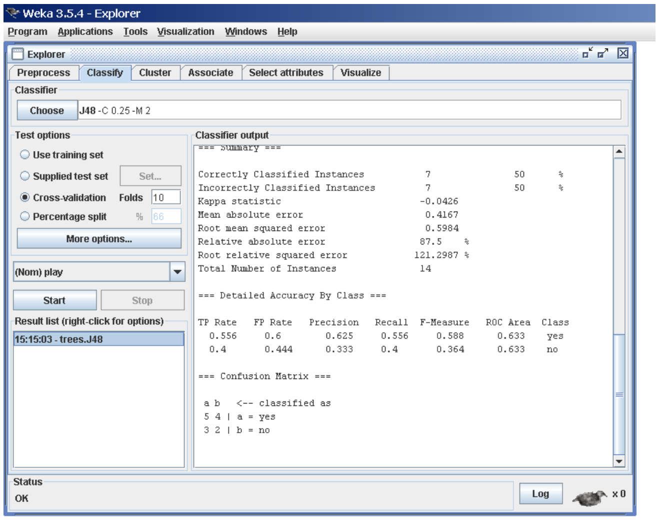

4.3.1 Selecting a Classifier

At the top of the classify section is the **Classifier** box. This box has a text field that gives the name of the currently selected classifier, and its options. Clicking on the text box with the left mouse button brings up a GenericObjectEditor dialog box, just the same as for filters, that you can use to configure the options of the current classifier. With a *right click* (or *Alt+Shift+left click*) you can once again copy the setup string to the clipboard or display the properties in a GenericObjectEditor dialog box. The **Choose** button allows you to choose one of the classifiers that are available in WEKA.

4.3.2 Test Options

The result of applying the chosen classifier will be tested according to the options that are set by clicking in the **Test options** box. There are four test modes:

1. **Use training set**. The classifier is evaluated on how well it predicts the class of the instances it was trained on.

2. **Supplied test set**. The classifier is evaluated on how well it predicts the class of a set of instances loaded from a file. Clicking the **Set...** button brings up a dialog allowing you to choose the file to test on.

3. **Cross-validation**. The classifier is evaluated by cross-validation, using the number of folds that are entered in the **Folds** text field.

4. **Percentage split**. The classifier is evaluated on how well it predicts a certain percentage of the data which is held out for testing. The amount of data held out depends on the value entered in the **%** field.

Note: No matter which evaluation method is used, the model that is output is always the one build from ***all*** the training data. Further testing options can be set by clicking on the **More options...** button:

1. **Output model**. The classification model on the full training set is output so that it can be viewed, visualized, etc. This option is selected by default.

2. **Output per-class stats**. The precision/recall and true/false statistics for each class are output. This option is also selected by default.

3. **Output entropy evaluation measures**. Entropy evaluation measures are included in the output. This option is not selected by default.

4. **Output confusion matrix**. The confusion matrix of the classifier's predictions is included in the output. This option is selected by default.

5. **Store predictions for visualization**. The classifier's predictions are remembered so that they can be visualized. This option is selected by default.

6. **Output predictions**. The predictions on the evaluation data are output. Note that in the case of a cross-validation the instance numbers do not correspond to the location in the data!

7. **Output additional attributes**. If additional attributes need to be output alongside the predictions, e.g., an ID attribute for tracking misclassifications, then the index of this attribute can be specified here. The usual Weka ranges are supported,"first" and "last" are therefore valid indices as well (example: "first-3,6,8,12-last").

8. **Cost-sensitive evaluation**. The errors is evaluated with respect to a cost matrix. The **Set...** button allows you to specify the cost matrix used.

9. **Random seed for xval / % Split**. This specifies the random seed used when randomizing the data before it is divided up for evaluation purposes.

10. **Preserve order for % Split**. This suppresses the randomization of the data before splitting into train and test set.

11. **Output source code**. If the classifier can output the built model as Java source code, you can specify the class name here. The code will be printed in the "Classifier output" area.

4.3.3 The Class Attribute

The classifiers in WEKA are designed to be trained to predict a single 'class' attribute, which is the target for prediction. Some classifiers can only learn nominal classes; others can only learn numeric classes (regression problems); still others can learn both.

By default, the class is taken to be the last attribute in the data. If you want to train a classifier to predict a different attribute, click on the box below the **Test options** box to bring up a drop-down list of attributes to choose from.

4.3.4 Training a Classifier

Once the classifier, test options and class have all been set, the learning process is started by clicking on the **Start** button. While the classifier is busy being trained, the little bird moves around. You can stop the training process at any time by clicking on the **Stop** button.

When training is complete, several things happen. The **Classifier output** area to the right of the display is filled with text describing the results of training and testing. A new entry appears in the **Result list** box. We look at the result list below; but first we investigate the text that has been output.

4.3.5 The Classifier Output Text

The text in the **Classifier output** area has scroll bars allowing you to browse the results. Clicking with the left mouse button into the text area, while holding `Alt` and `Shift`, brings up a dialog that enables you to save the displayed output in a variety of formats (currently, BMP, EPS, JPEG and PNG). Of course, you can also resize the Explorer window to get a larger display area. The output is split into several sections:

1. **Run information**. A list of information giving the learning scheme options, relation name, instances, attributes and test mode that were involved in the process.

2. **Classifier model (full training set)**. A textual representation of the classification model that was produced on the full training data.

3. The results of the chosen test mode are broken down thus:

4. **Summary**. A list of statistics summarizing how accurately the classifier was able to predict the true class of the instances under the chosen test mode.

5. **Detailed Accuracy By Class**. A more detailed per-class break down of the classifier's prediction accuracy.

6. **Confusion Matrix**. Shows how many instances have been assigned to each class. Elements show the number of test examples whose actual class is the row and whose predicted class is the column.

7. **Source code** (optional). This section lists the Java source code if one chose "Output source code" in the "More options" dialog.

4.3.6 The Result List

After training several classifiers, the result list will contain several entries. Left-clicking the entries flicks back and forth between the various results that have been generated. Pressing `Delete` removes a selected entry from the results. Right-clicking an entry invokes a menu containing these items:

1. **View in main window**. Shows the output in the main window (just like left-clicking the entry).

2. **View in separate window**. Opens a new independent window for viewing the results.

3. **Save result buffer**. Brings up a dialog allowing you to save a text file containing the textual output.

4. **Load model**. Loads a pre-trained model object from a binary file.

5. **Save model**. Saves a model object to a binary file. Objects are saved in Java 'serialized object' form.

6. **Re-evaluate model on current test set**. Takes the model that has been built and tests its performance on the data set that has been specified with the **Set..** button under the **Supplied test set** option.

7. **Visualize classifier errors**. Brings up a visualization window that plots the results of classification. Correctly classified instances are represented by crosses, whereas incorrectly classified ones show up as squares.

8. **Visualize tree** or **Visualize graph**. Brings up a graphical representation of the structure of the classifier model, if possible (i.e. for decision trees or Bayesian networks). The graph visualization option only appears if a Bayesian network classifier has been built. In the tree visualizer, you can bring up a menu by right-clicking a blank area, pan around by dragging the mouse, and see the training instances at each node by clicking on it. CTRL-clicking zooms the view out, while SHIFT-dragging a box zooms the view in. The graph visualizer should be self-explanatory.

9. **Visualize margin curve**. Generates a plot illustrating the prediction margin. The margin is defined as the difference between the probability predicted for the actual class and the highest probability predicted for the other classes. For example, boosting algorithms may achieve better performance on test data by increasing the margins on the training data.

10. **Visualize threshold curve**. Generates a plot illustrating the trade-offs in prediction that are obtained by varying the threshold value between classes. For example, with the default threshold value of 0.5, the predicted probability of 'positive' must be greater than 0.5 for the instance to be predicted as 'positive'. The plot can be used to visualize the precision/recall trade-off, for ROC curve analysis (true positive rate *vs* false positive rate), and for other types of curves.

11. **Visualize cost curve**. Generates a plot that gives an explicit representation of the expected cost, as described by [5].

12. **Plugins**. This menu item only appears if there are visualization plugins available (by default: none). More about these plugins can be found in the *WekaWiki* article "Explorer visualization plugins" [8].

Options are greyed out if they do not apply to the specific set of results.

4.4 Clustering

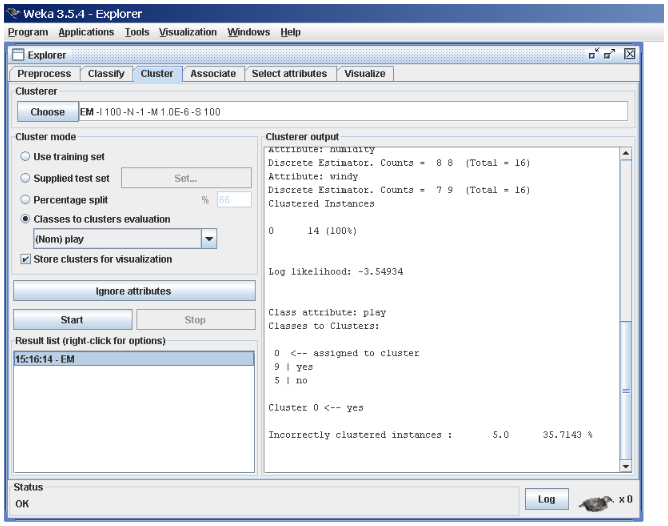

4.4.1 Selecting a Clusterer

By now you will be familiar with the process of selecting and configuring objects. Clicking on the clustering scheme listed in the **Clusterer** box at the top of the window brings up a GenericObjectEditor dialog with which to choose a new clustering scheme.

4.4.2 Cluster Modes

The **Cluster mode** box is used to choose what to cluster and how to evaluate the results. The first three options are the same as for classification: **Use training set**, **Supplied test set** and **Percentage split** (Section 4.3.1)—except that now the data is assigned to clusters instead of trying to predict a specific class. The fourth mode, **Classes to clusters evaluation**, compares how well the chosen clusters match up with a pre-assigned class in the data. The drop-down box below this option selects the class, just as in the **Classify** panel.

An additional option in the **Cluster mode** box, the **Store clusters for visualization** tick box, determines whether or not it will be possible to visualize the clusters once training is complete. When dealing with datasets that are so large that memory becomes a problem it may be helpful to disable this option.

4.4.3 Ignoring Attributes

Often, some attributes in the data should be ignored when clustering. The **Ignore attributes** button brings up a small window that allows you to select which attributes are ignored. Clicking on an attribute in the window highlights it, holding down the SHIFT key selects a range of consecutive attributes, and holding down CTRL toggles individual attributes on and off. To cancel the selection, back out with the **Cancel** button. To activate it, click the **Select** button. The next time clustering is invoked, the selected attributes are ignored.

4.4.4 Working with Filters

The `FilteredClusterer` meta-clusterer offers the user the possibility to apply filters directly before the clusterer is learned. This approach eliminates the manual application of a filter in the **Preprocess** panel, since the data gets processed on the fly. Useful if one needs to try out different filter setups.

4.4.5 Learning Clusters

The **Cluster** section, like the **Classify** section, has **Start/Stop** buttons, a result text area and a result list. These all behave just like their classification counterparts. Right-clicking an entry in the result list brings up a similar menu, except that it shows only two visualization options: **Visualize cluster assignments** and **Visualize tree**. The latter is grayed out when it is not applicable.

4.5 Associating

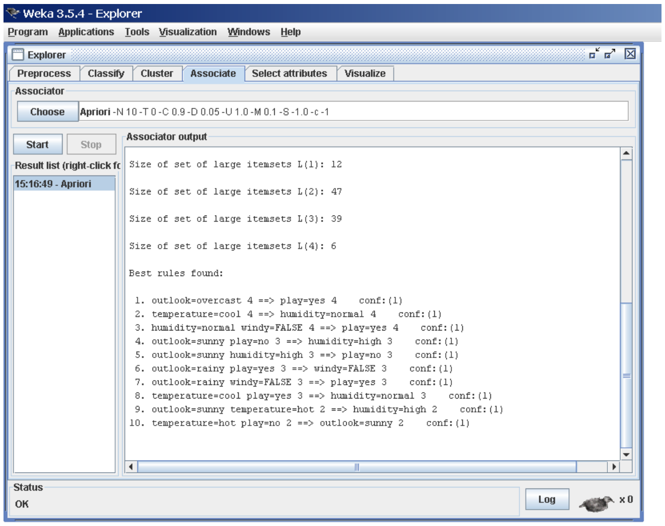

4.5.1 Setting Up

This panel contains schemes for learning association rules, and the learners are chosen and configured in the same way as the clusterers, filters, and classifiers in the other panels.

4.5.2 Learning Associations

Once appropriate parameters for the association rule learner bave been set, click the **Start** button. When complete, right-clicking on an entry in the result list allows the results to be viewed or saved.

4.6 Selecting Attributes

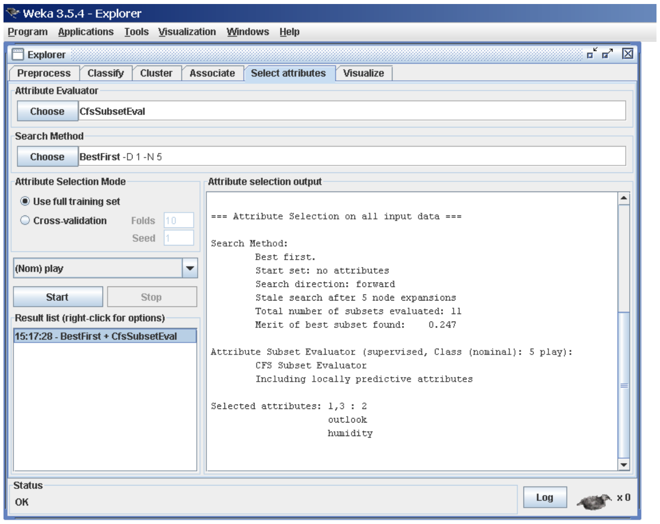

4.6.1 Searching and Evaluating

Attribute selection involves searching through all possible combinations of attributes in the data to find which subset of attributes works best for prediction. To do this, two objects must be set up: an attribute evaluator and a search method. The evaluator determines what method is used to assign a worth to each subset of attributes. The search method determines what style of search is performed.

4.6.2 Options

The **Attribute Selection Mode** box has two options:

1. **Use full training set**. The worth of the attribute subset is determined using the full set of training data.

2. **Cross-validation**. The worth of the attribute subset is determined by a process of cross-validation. The **Fold** and **Seed** fields set the number of folds to use and the random seed used when shuffling the data.

As with **Classify** (Section 4.3.1), there is a drop-down box that can be used to specify which attribute to treat as the class.

4.6.3 Performing Selection

Clicking **Start** starts running the attribute selection process. When it is finished, the results are output into the result area, and an entry is added to the result list. Right-clicking on the result list gives several options. The first three, (**View in main window**, **View in separate window** and **Save result buffer**), are the same as for the classify panel. It is also possible to **Visualize**

reduced data, or if you have used an attribute transformer such as PrincipalComponents, **Visualize transformed data**. The reduced/transformed data can be saved to a file with the **Save reduced data...** or **Save transformed data...** option.

In case one wants to reduce/transform a training and a test at the same time and not use the AttributeSelectedClassifier from the classifier panel, it is best to use the AttributeSelection filter (a supervised attribute filter) in batch mode ('`-b`') from the command line or in the SimpleCLI. The batch mode allows one to specify an additional input and output file pair (options `-r` and `-s`), that is processed with the filter setup that was determined based on the training data (specified by options `-i` and `-o`).

Here is an example for a Unix/Linux bash:

```
java weka.filters.supervised.attribute.AttributeSelection \
       -E "weka.attributeSelection.CfsSubsetEval " \
       -S "weka.attributeSelection.BestFirst -D 1 -N 5" \
       -b \
       -i <input1.arff> \
       -o <output1.arff> \
       -r <input2.arff> \
       -s <output2.arff>
```

Notes:

- The "backslashes" at the end of each line tell the bash that the command is not finished yet. Using the SimpleCLI one has to use this command in one line without the backslashes.
- It is assumed that WEKA is available in the `CLASSPATH`, otherwise one has to use the `-classpath` option.
- The full filter setup is output in the log, as well as the setup for running regular attribute selection.

4.7 Visualizing

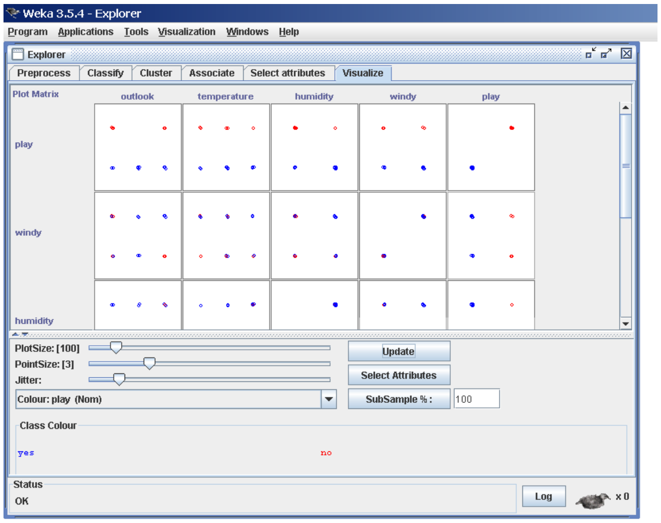

WEKA's visualization section allows you to visualize 2D plots of the current relation.

4.7.1 The scatter plot matrix

When you select the *Visualize* panel, it shows a scatter plot matrix for all the attributes, colour coded according to the currently selected class. It is possible to change the size of each individual 2D plot and the point size, and to randomly jitter the data (to uncover obscured points). It also possible to change the attribute used to colour the plots, to select only a subset of attributes for inclusion in the scatter plot matrix, and to sub sample the data. Note that changes will only come into effect once the **Update** button has been pressed.

4.7.2 Selecting an individual 2D scatter plot

When you click on a cell in the scatter plot matrix, this will bring up a separate window with a visualization of the scatter plot you selected. (We described above how to visualize particular results in a separate window—for example, classifier errors—the same visualization controls are used here.)

Data points are plotted in the main area of the window. At the top are two drop-down list buttons for selecting the axes to plot. The one on the left shows which attribute is used for the x-axis; the one on the right shows which is used for the y-axis.

Beneath the x-axis selector is a drop-down list for choosing the colour scheme. This allows you to colour the points based on the attribute selected. Below the plot area, a legend describes what values the colours correspond to. If the values are discrete, you can modify the colour used for each one by clicking on them and making an appropriate selection in the window that pops up.

To the right of the plot area is a series of horizontal strips. Each strip represents an attribute, and the dots within it show the distribution of values

of the attribute. These values are randomly scattered vertically to help you see concentrations of points. You can choose what axes are used in the main graph by clicking on these strips. Left-clicking an attribute strip changes the x-axis to that attribute, whereas right-clicking changes the y-axis. The ‘X’ and ‘Y’ written beside the strips shows what the current axes are (‘B’ is used for ‘both X and Y’).

Above the attribute strips is a slider labelled **Jitter**, which is a random displacement given to all points in the plot. Dragging it to the right increases the amount of jitter, which is useful for spotting concentrations of points. Without jitter, a million instances at the same point would look no different to just a single lonely instance.

4.7.3 Selecting Instances

There may be situations where it is helpful to select a subset of the data using the visualization tool. (A special case of this is the UserClassifier in the *Classify* panel, which lets you build your own classifier by interactively selecting instances.)

Below the y-axis selector button is a drop-down list button for choosing a selection method. A group of data points can be selected in four ways:

1. **Select Instance**. Clicking on an individual data point brings up a window listing its attributes. If more than one point appears at the same location, more than one set of attributes is shown.

2. **Rectangle**. You can create a rectangle, by dragging, that selects the points inside it.

3. **Polygon**. You can build a free-form polygon that selects the points inside it. Left-click to add vertices to the polygon, right-click to complete it. The polygon will always be closed off by connecting the first point to the last.

4. **Polyline**. You can build a polyline that distinguishes the points on one side from those on the other. Left-click to add vertices to the polyline, right-click to finish. The resulting shape is open (as opposed to a polygon, which is always closed).

Once an area of the plot has been selected using **Rectangle**, **Polygon** or **Polyline**, it turns grey. At this point, clicking the **Submit** button removes all instances from the plot except those within the grey selection area. Clicking on the **Clear** button erases the selected area without affecting the graph.

Once any points have been removed from the graph, the **Submit** button changes to a **Reset** button. This button undoes all previous removals and returns you to the original graph with all points included. Finally, clicking the **Save** button allows you to save the currently visible instances to a new ARFF file.

Chapter 5

Experimenter

5.1 Introduction

The Weka Experiment Environment enables the user to create, run, modify, and analyse experiments in a more convenient manner than is possible when processing the schemes individually. For example, the user can create an experiment that runs several schemes against a series of datasets and then analyse the results to determine if one of the schemes is (statistically) better than the other schemes.

The Experiment Environment can be run from the command line using the Simple CLI. For example, the following commands could be typed into the CLI to run the `OneR` scheme on the Iris dataset using a basic train and test process. (Note that the commands would be typed on one line into the CLI.)

```
java weka.experiment.Experiment -r -T data/iris.arff
  -D weka.experiment.InstancesResultListener
  -P weka.experiment.RandomSplitResultProducer --
  -W weka.experiment.ClassifierSplitEvaluator --
  -W weka.classifiers.rules.OneR
```

While commands can be typed directly into the CLI, this technique is not particularly convenient and the experiments are not easy to modify.

The Experimenter comes in two flavours, either with a simple interface that provides most of the functionality one needs for experiments, or with an interface with full access to the Experimenter's capabilities. You can choose between those two with the *Experiment Configuration Mode* radio buttons:

- Simple
- Advanced

Both setups allow you to setup *standard* experiments, that are run locally on a single machine, or remote experiments, which are distributed between several hosts. The distribution of experiments cuts down the time the experiments will take until completion, but on the other hand the setup takes more time.

The next section covers the *standard* experiments (both, simple and advanced), followed by the *remote* experiments and finally the *analysing* of the results.

5.2 Standard Experiments

5.2.1 Simple

5.2.1.1 New experiment

After clicking *New* default parameters for an Experiment are defined.

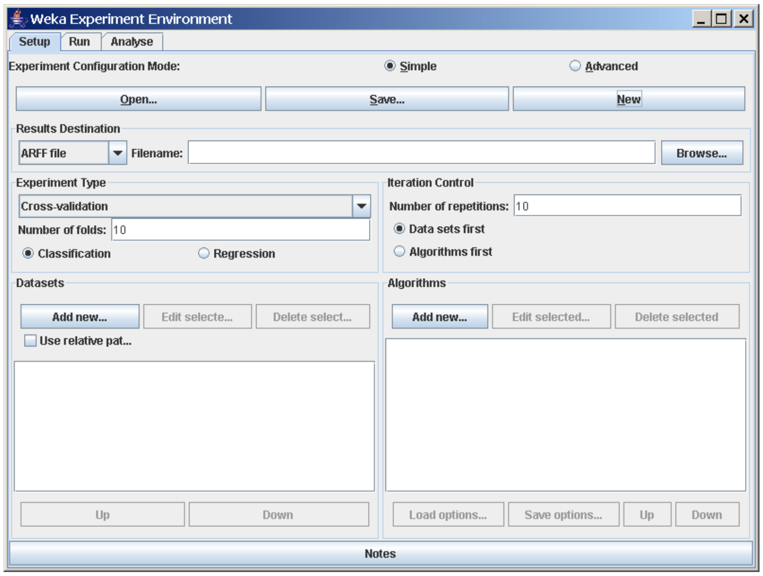

5.2.1.2 Results destination

By default, an ARFF file is the destination for the results output. But you can choose between

- ARFF file
- CSV file
- JDBC database

ARFF file and JDBC database are discussed in detail in the following sections. CSV is similar to ARFF, but it can be used to be loaded in an external spreadsheet application.

ARFF file

If the file name is left empty a temporary file will be created in the TEMP directory of the system. If one wants to specify an explicit results file, click on *Browse* and choose a filename, e.g., *Experiment1.arff*.

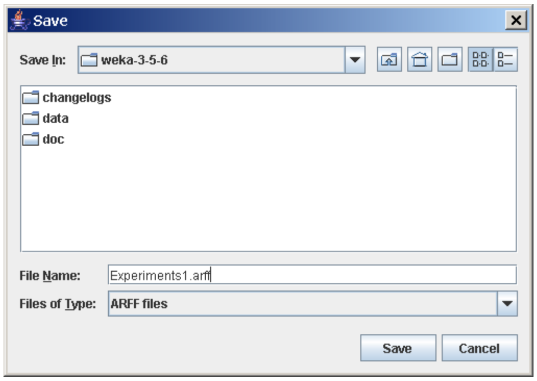

Click on *Save* and the name will appear in the edit field next to *ARFF file*.

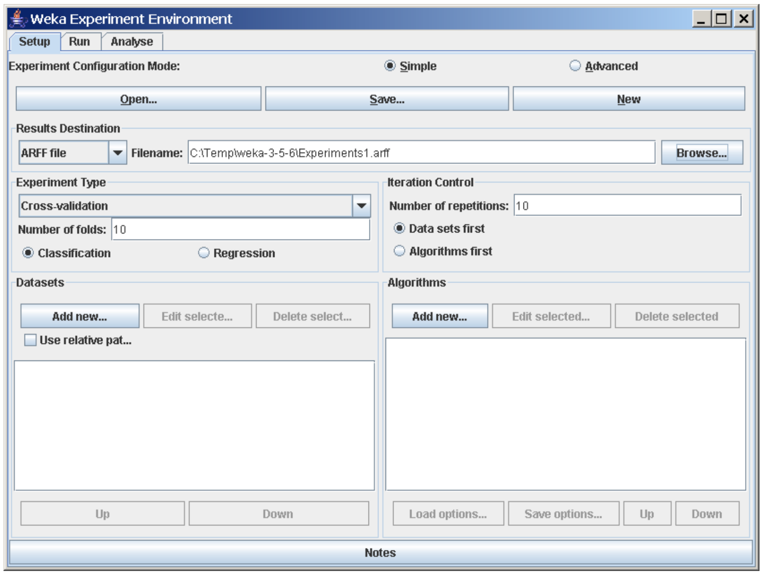

The advantage of ARFF or CSV files is that they can be created without any additional classes besides the ones from Weka. The drawback is the lack of the ability to resume an experiment that was interrupted, e.g., due to an error or the addition of dataset or algorithms. Especially with time-consuming experiments, this behavior can be annoying.

JDBC database

With JDBC it is easy to store the results in a database. The necessary jar archives have to be in the CLASSPATH to make the JDBC functionality of a particular database available.

After changing *ARFF file* to *JDBC database* click on *User...* to specify JDBC URL and user credentials for accessing the database.

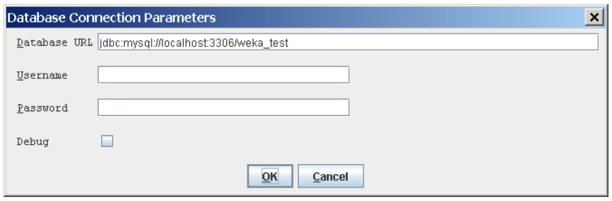

After supplying the necessary data and clicking on *OK*, the URL in the main window will be updated.

Note: at this point, the database connection is not tested; this is done when the experiment is started.

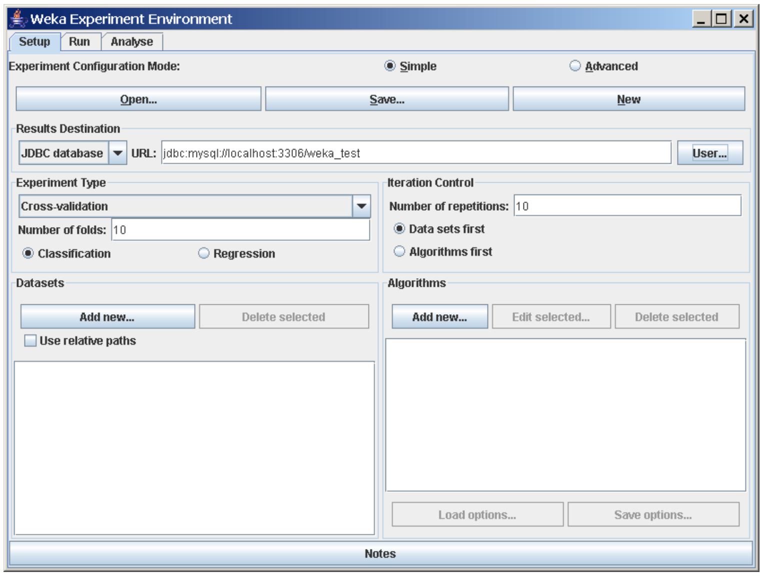

The advantage of a JDBC database is the possibility to resume an interrupted or extended experiment. Instead of re-running all the other algorithm/dataset combinations again, only the missing ones are computed.

5.2.1.3 Experiment type

The user can choose between the following three different types

- **Cross-validation (default)**
  performs stratified cross-validation with the given number of folds

- **Train/Test Percentage Split (data randomized)**
  splits a dataset according to the given percentage into a train and a test file (one cannot specify explicit training and test files in the Experimenter), after the order of the data has been randomized and stratified

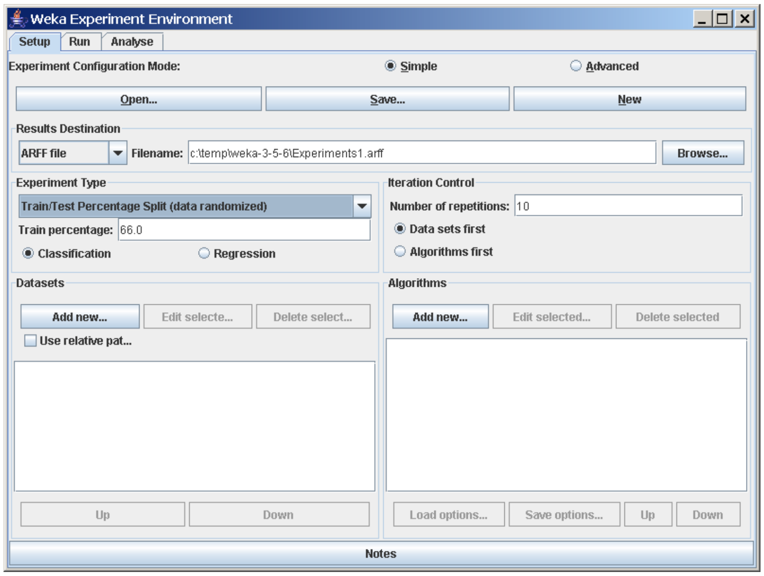

- **Train/Test Percentage Split (order preserved)**
  because it is impossible to specify an explicit train/test files pair, one can *abuse* this type to *un-merge* previously merged train and test file into the two original files (one only needs to find out the correct percentage)

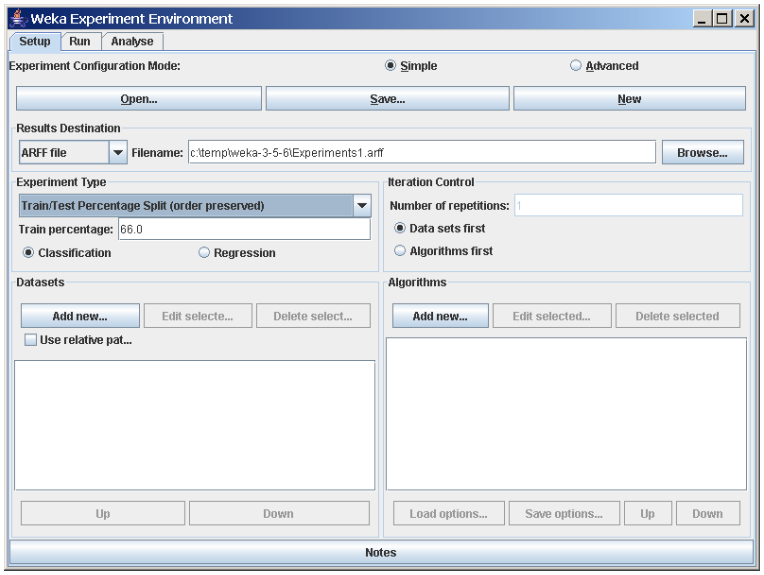

Additionally, one can choose between *Classification* and *Regression*, depending on the datasets and classifiers one uses. For decision trees like `J48` (Weka's implementation of Quinlan's C4.5 [10]) and the iris dataset, *Classification* is necessary, for a numeric classifier like `M5P`, on the other hand, *Regression*. *Classification* is selected by default.

Note: if the percentage splits are used, one has to make sure that the corrected paired *T-Tester* still produces sensible results with the given ratio [9].

5.2.1.4 Datasets

One can add dataset files either with an absolute path or with a relative one. The latter makes it often easier to run experiments on different machines, hence one should check *Use relative paths*, before clicking on *Add new...*.

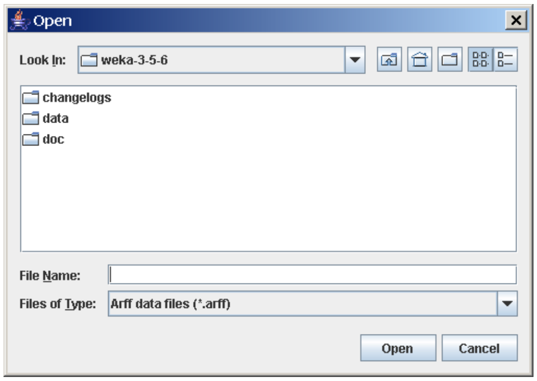

In this example, open the *data* directory and choose the *iris.arff* dataset.

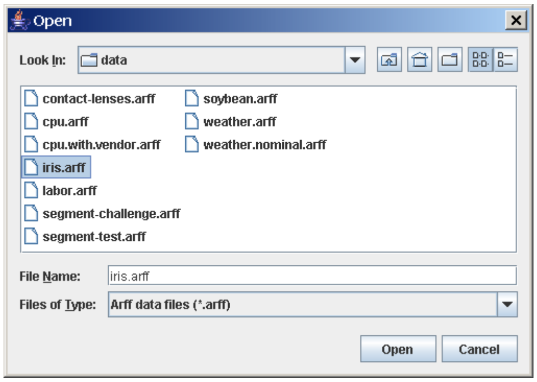

After clicking *Open* the file will be displayed in the datasets list. If one selects a directory and hits *Open*, then all ARFF files will be added recursively. Files can be deleted from the list by selecting them and then clicking on *Delete selected*.

ARFF files are not the only format one can load, but *all* files that can be converted with Weka's *"core converters"*. The following formats are currently supported:

- ARFF (+ compressed)
- C4.5
- CSV
- libsvm
- binary serialized instances
- XRFF (+ compressed)

By default, the class attribute is assumed to be the last attribute. But if a data format contains information about the class attribute, like XRFF or C4.5, this attribute will be used instead.

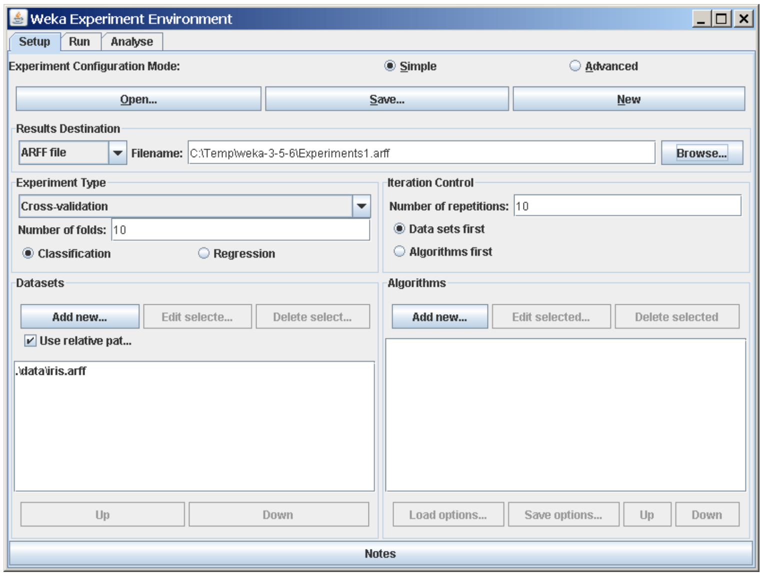

5.2.1.5 Iteration control

- **Number of repetitions**
  In order to get statistically meaningful results, the default number of iterations is 10. In case of 10-fold cross-validation this means 100 calls of one classifier with training data and tested against test data.

- **Data sets first/Algorithms first**
  As soon as one has more than one dataset and algorithm, it can be useful to switch from datasets being iterated over first to algorithms. This is the case if one stores the results in a database and wants to complete the results for all the datasets for one algorithm as early as possible.

5.2.1.6 Algorithms

New algorithms can be added via the *Add new...* button. Opening this dialog for the first time, `ZeroR` is presented, otherwise the one that was selected last.

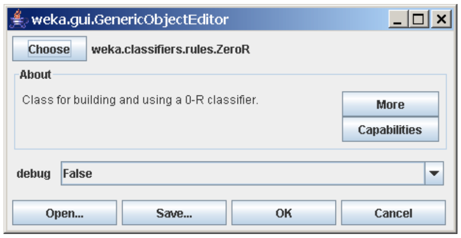

With the *Choose* button one can open the *GenericObjectEditor* and choose another classifier.

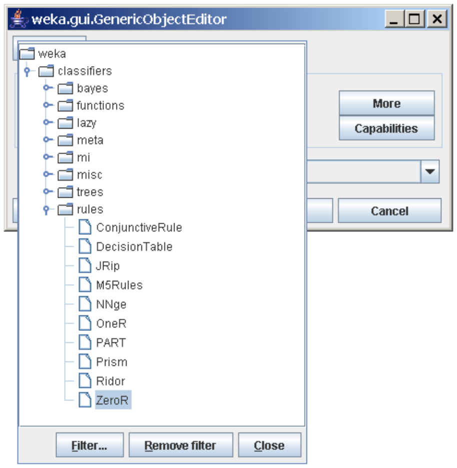

The *Filter...* button enables one to highlight classifiers that can handle certain attribute and class types. With the *Remove filter* button all the selected capabilities will get cleared and the highlighting removed again.

Additional algorithms can be added again with the *Add new...* button, e.g., the `J48` decision tree.

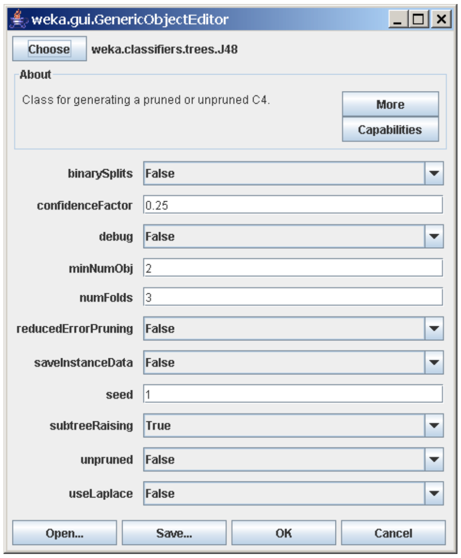

After setting the classifier parameters, one clicks on *OK* to add it to the list of algorithms.

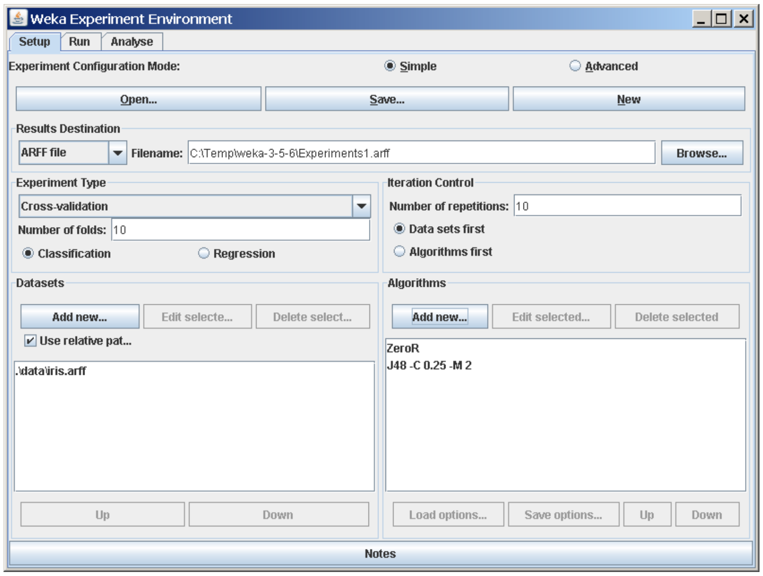

With the *Load options...* and *Save options...* buttons one can load and save the setup of a selected classifier from and to XML. This is especially useful for highly configured classifiers (e.g., nested meta-classifiers), where the manual setup takes quite some time, and which are used often.

One can also paste classifier settings here by right-clicking (or *Alt-Shift-left-clicking*) and selecting the appropriate menu point from the popup menu, to either add a new classifier or replace the selected one with a new setup. This is rather useful for transferring a classifier setup from the Weka Explorer over to the Experimenter without having to setup the classifier from scratch.

5.2.1.7 Saving the setup

For future re-use, one can save the current setup of the experiment to a file by clicking on *Save...* at the top of the window.

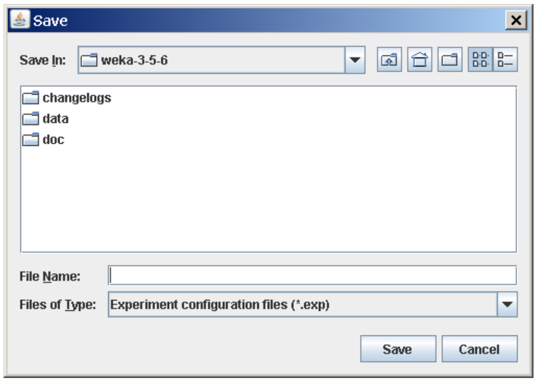

By default, the format of the experiment files is the binary format that Java serialization offers. The drawback of this format is the possible incompatibility between different versions of Weka. A more robust alternative to the binary format is the XML format.

Previously saved experiments can be loaded again via the *Open...* button.

5.2.1.8 Running an Experiment

To run the current experiment, click the *Run* tab at the top of the Experiment Environment window. The current experiment performs 10 runs of 10-fold stratified cross-validation on the Iris dataset using the `ZeroR` and `J48` scheme.

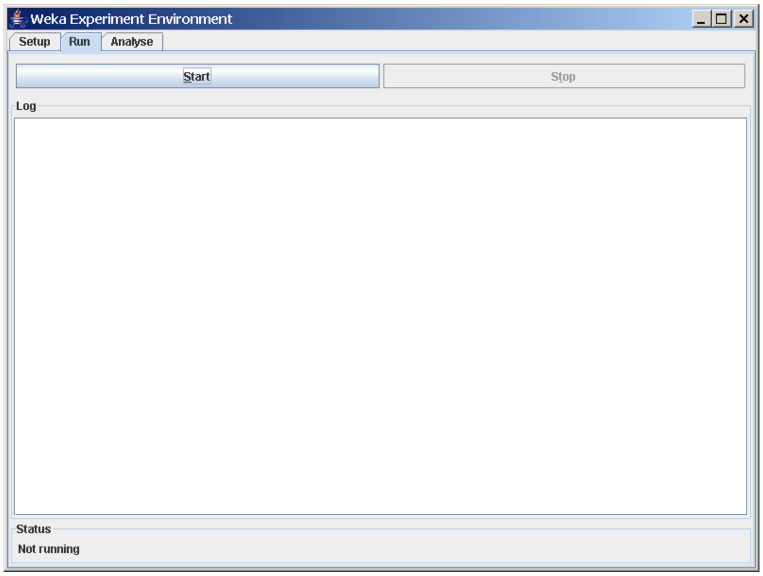

Click *Start* to run the experiment.

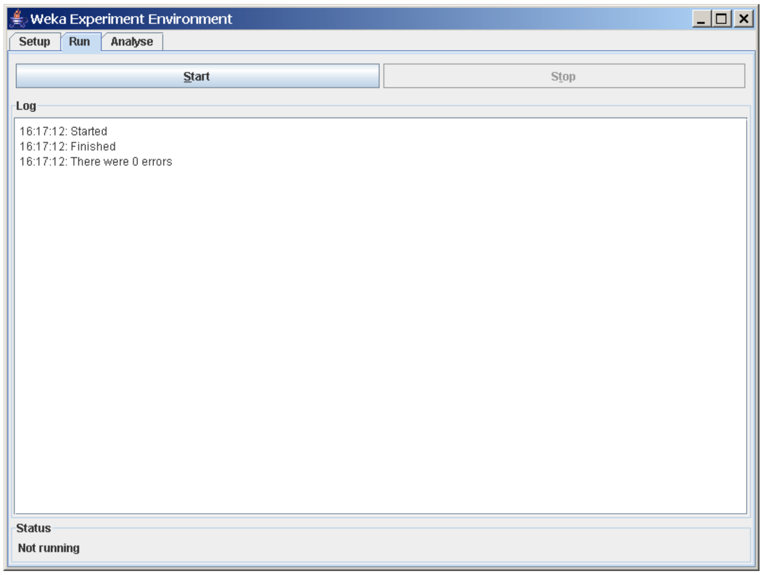

If the experiment was defined correctly, the 3 messages shown above will be displayed in the *Log* panel. The results of the experiment are saved to the dataset *Experiment1.arff*.

5.2.2 Advanced

5.2.2.1 Defining an Experiment

When the Experimenter is started in *Advanced* mode, the *Setup* tab is displayed. Click *New* to initialize an experiment. This causes default parameters to be defined for the experiment.

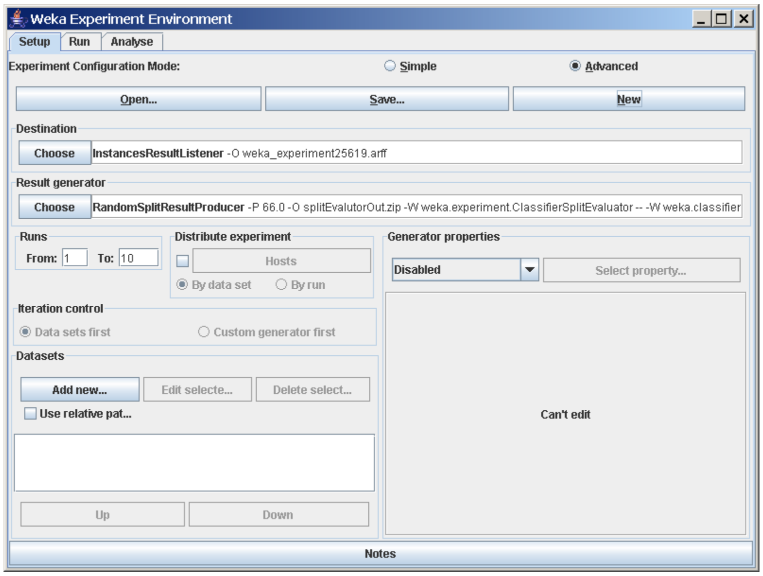

To define the dataset to be processed by a scheme, first select *Use relative paths* in the *Datasets* panel of the *Setup* tab and then click on *Add new...* to open a dialog window.

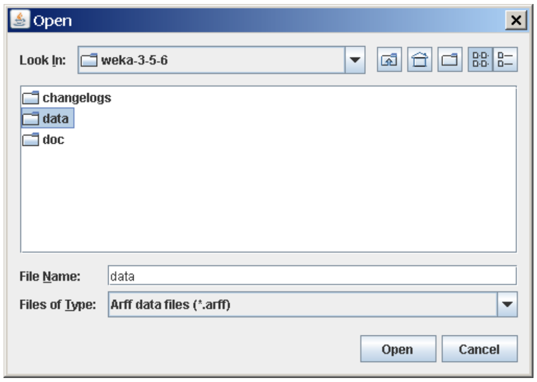

Double click on the *data* folder to view the available datasets or navigate to an alternate location. Select *iris.arff* and click *Open* to select the Iris dataset.

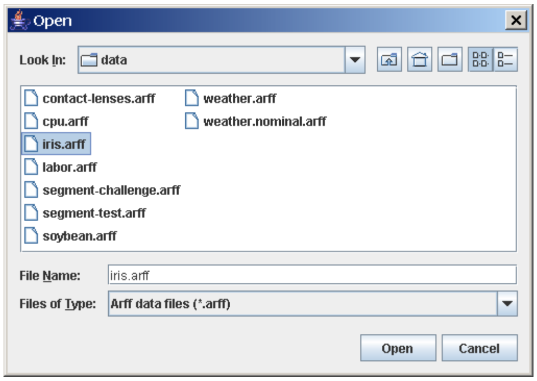

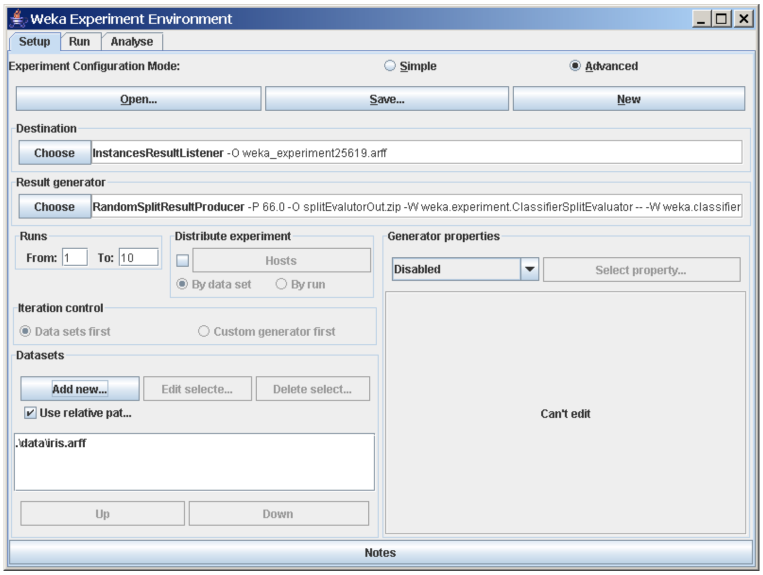

The dataset name is now displayed in the *Datasets* panel of the *Setup* tab.

Saving the Results of the Experiment

To identify a dataset to which the results are to be sent, click on the *Instances-ResultListener* entry in the *Destination* panel. The output file parameter is near the bottom of the window, beside the text *outputFile*. Click on this parameter to display a file selection window.

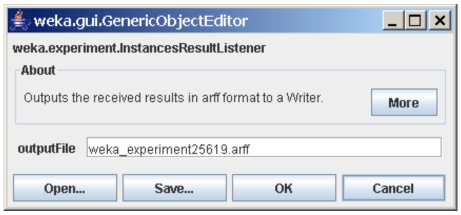

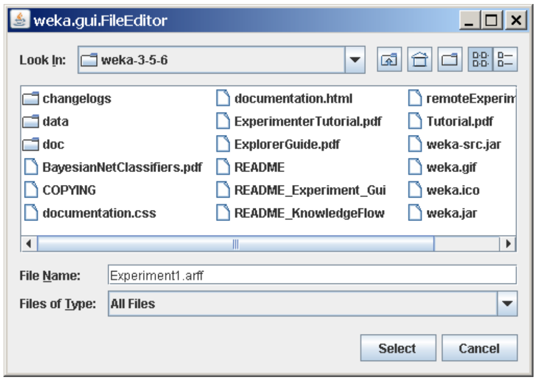

Type the name of the output file and click *Select*. The file name is displayed in the *outputFile* panel. Click on *OK* to close the window.

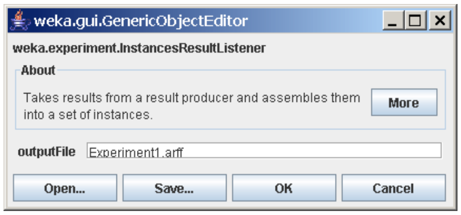

The dataset name is displayed in the *Destination* panel of the *Setup* tab.

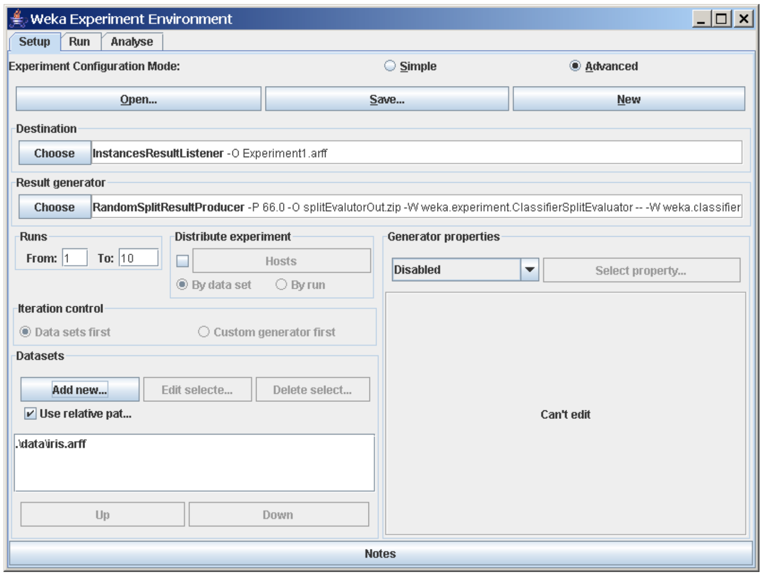

Saving the Experiment Definition

The experiment definition can be saved at any time. Select *Save…* at the top of the *Setup* tab. Type the dataset name with the extension *exp* (or select the dataset name if the experiment definition dataset already exists) for binary files or choose *Experiment configuration files (*.xml)* from the file types combobox (the XML files are robust with respect to version changes).

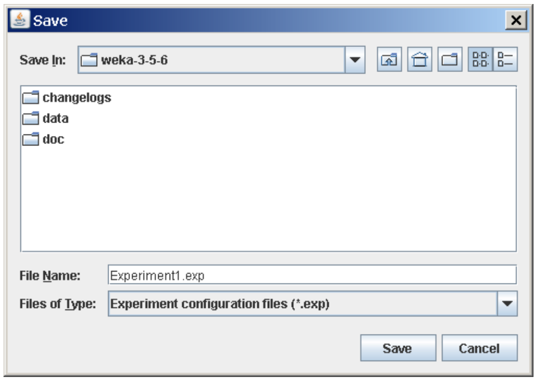

The experiment can be restored by selecting *Open* in the *Setup* tab and then selecting *Experiment1.exp* in the dialog window.

5.2.2.2 Running an Experiment

To run the current experiment, click the *Run* tab at the top of the Experiment Environment window. The current experiment performs 10 randomized train and test runs on the Iris dataset, using 66% of the patterns for training and 34% for testing, and using the `ZeroR` scheme.

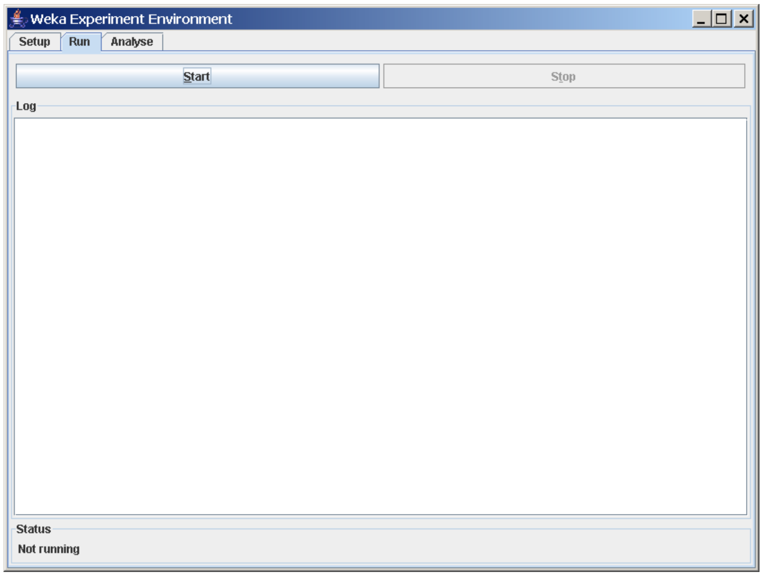

Click *Start* to run the experiment.

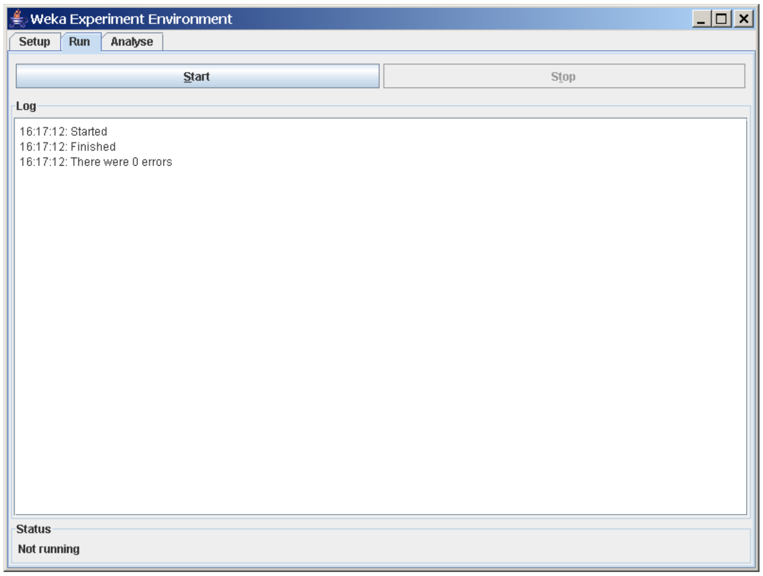

If the experiment was defined correctly, the 3 messages shown above will be displayed in the *Log* panel. The results of the experiment are saved to the dataset *Experiment1.arff*. The first few lines in this dataset are shown below.

```
@relation InstanceResultListener

@attribute Key_Dataset {iris}
@attribute Key_Run {1,2,3,4,5,6,7,8,9,10}
@attribute Key_Scheme {weka.classifiers.rules.ZeroR,weka.classifiers.trees.J48}
@attribute Key_Scheme_options {,'-C 0.25 -M 2'}
@attribute Key_Scheme_version_ID {48055541465867954,-217733168393644444}
@attribute Date_time numeric
@attribute Number_of_training_instances numeric
@attribute Number_of_testing_instances numeric
@attribute Number_correct numeric
@attribute Number_incorrect numeric
@attribute Number_unclassified numeric
@attribute Percent_correct numeric
@attribute Percent_incorrect numeric
@attribute Percent_unclassified numeric
@attribute Kappa_statistic numeric
@attribute Mean_absolute_error numeric
@attribute Root_mean_squared_error numeric
@attribute Relative_absolute_error numeric
@attribute Root_relative_squared_error numeric
@attribute SF_prior_entropy numeric
@attribute SF_scheme_entropy numeric
@attribute SF_entropy_gain numeric
@attribute SF_mean_prior_entropy numeric
@attribute SF_mean_scheme_entropy numeric
@attribute SF_mean_entropy_gain numeric
@attribute KB_information numeric
```

```
@attribute KB_mean_information numeric
@attribute KB_relative_information numeric
@attribute True_positive_rate numeric
@attribute Num_true_positives numeric
@attribute False_positive_rate numeric
@attribute Num_false_positives numeric
@attribute True_negative_rate numeric
@attribute Num_true_negatives numeric
@attribute False_negative_rate numeric
@attribute Num_false_negatives numeric
@attribute IR_precision numeric
@attribute IR_recall numeric
@attribute F_measure numeric
@attribute Area_under_ROC numeric
@attribute Time_training numeric
@attribute Time_testing numeric
@attribute Summary {'Number of leaves: 3\nSize of the tree: 5\n',
   'Number of leaves: 5\nSize of the tree: 9\n',
   'Number of leaves: 4\nSize of the tree: 7\n'}
@attribute measureTreeSize numeric
@attribute measureNumLeaves numeric
@attribute measureNumRules numeric

@data

iris,1,weka.classifiers.rules.ZeroR,,48055541465867954,20051221.033,99,51,
17,34,0,33.333333,66.666667,0,0,0.444444,0.471405,100,100,80.833088,80.833088,
0,1.584963,1.584963,0,0,0,0,1,17,1,34,0,0,0,0,0.333333,1,0.5,0.5,0,0,?,?,?,?
```

5.2.2.3 Changing the Experiment Parameters

Changing the Classifier

The parameters of an experiment can be changed by clicking on the *Result generator* panel.

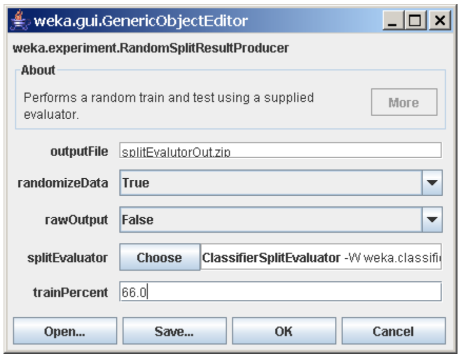

The *RandomSplitResultProducer* performs repeated train/test runs. The number of instances (expressed as a percentage) used for training is given in the

trainPercent box. (The number of runs is specified in the *Runs* panel in the *Setup* tab.)

A small help file can be displayed by clicking *More* in the *About* panel.

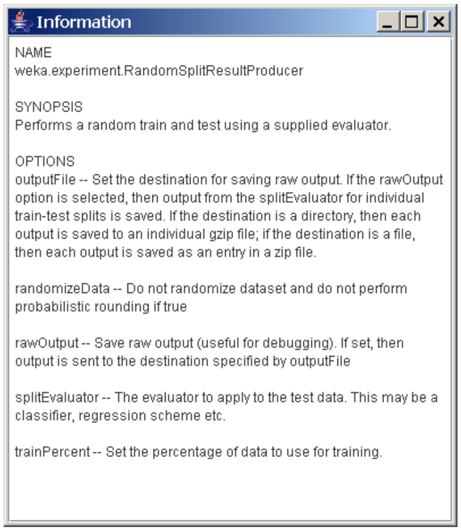

Click on the *splitEvaluator* entry to display the *SplitEvaluator* properties.

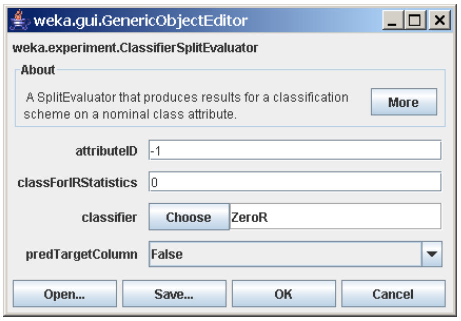

Click on the classifier entry (`ZeroR`) to display the scheme properties.

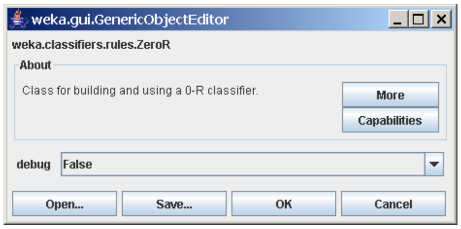

This scheme has no modifiable properties (besides *debug* mode on/off) but most other schemes do have properties that can be modified by the user. The *Capabilities* button opens a small dialog listing all the attribute and class types this classifier can handle. Click on the *Choose* button to select a different scheme. The window below shows the parameters available for the `J48` decision-tree scheme. If desired, modify the parameters and then click *OK* to close the window.

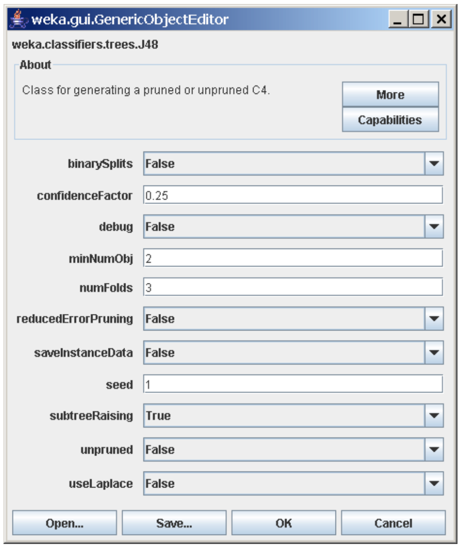

The name of the new scheme is displayed in the *Result generator* panel.

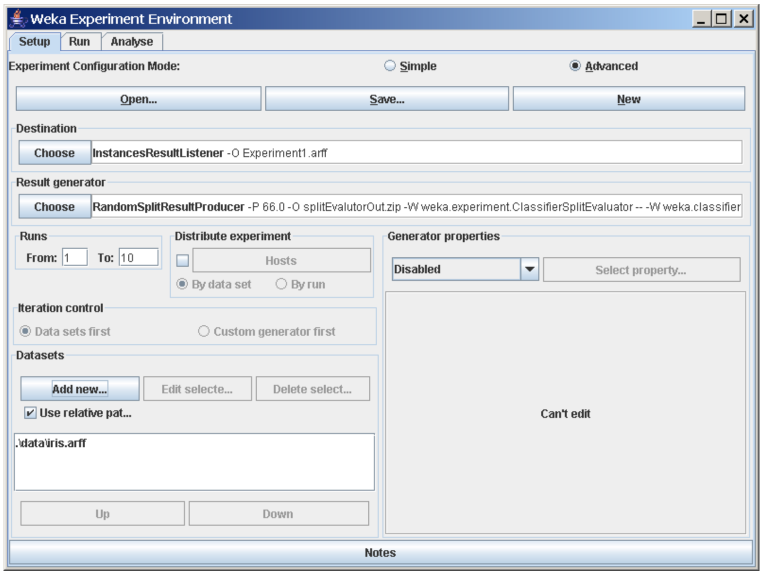

Adding Additional Schemes

Additional schemes can be added in the *Generator properties* panel. To begin, change the drop-down list entry from *Disabled* to *Enabled* in the *Generator properties* panel.

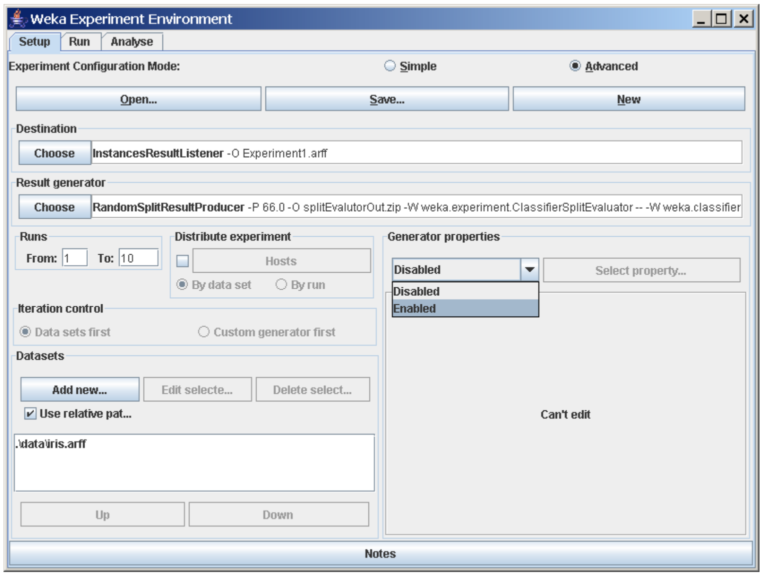

Click *Select property* and expand *splitEvaluator* so that the *classifier* entry is visible in the property list; click *Select*.

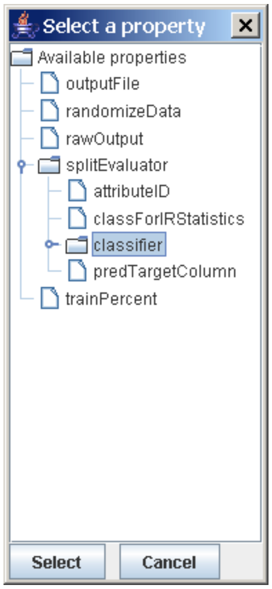

The scheme name is displayed in the *Generator properties* panel.

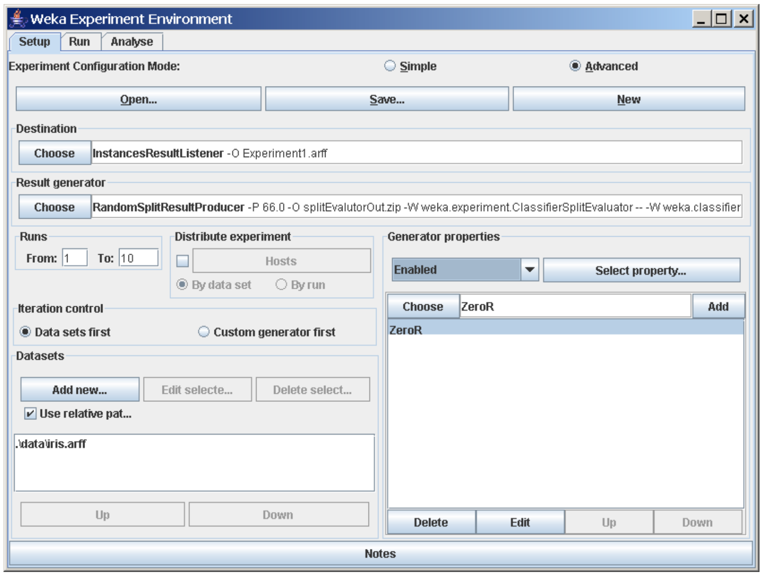

To add another scheme, click on the *Choose* button to display the *GenericObjectEditor* window.

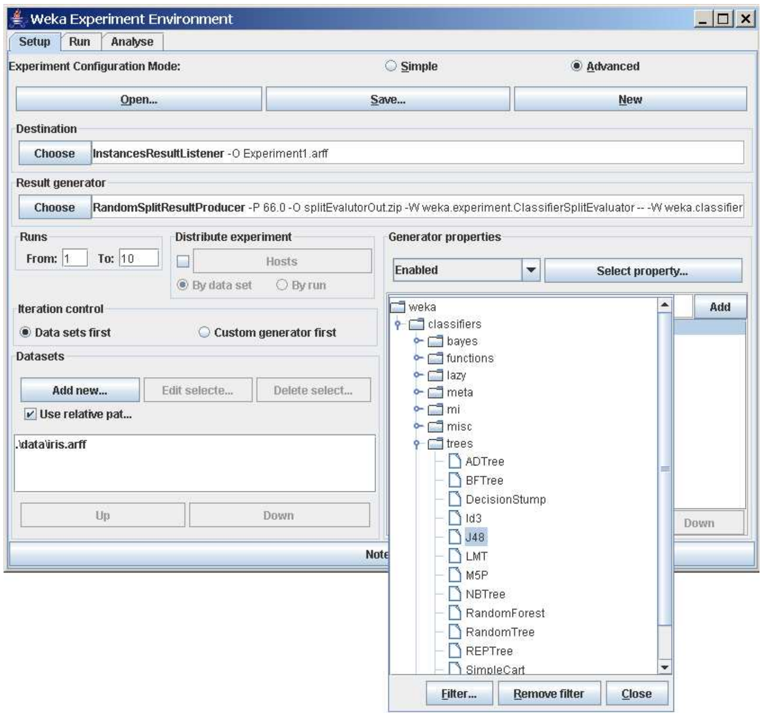

The *Filter...* button enables one to highlight classifiers that can handle certain attribute and class types. With the *Remove filter* button all the selected capabilities will get cleared and the highlighting removed again.

To change to a decision-tree scheme, select `J48` (in subgroup *trees*).

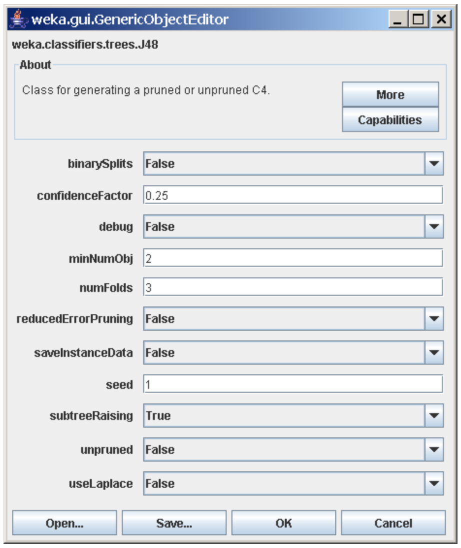

The new scheme is added to the *Generator properties* panel. Click *Add* to add the new scheme.

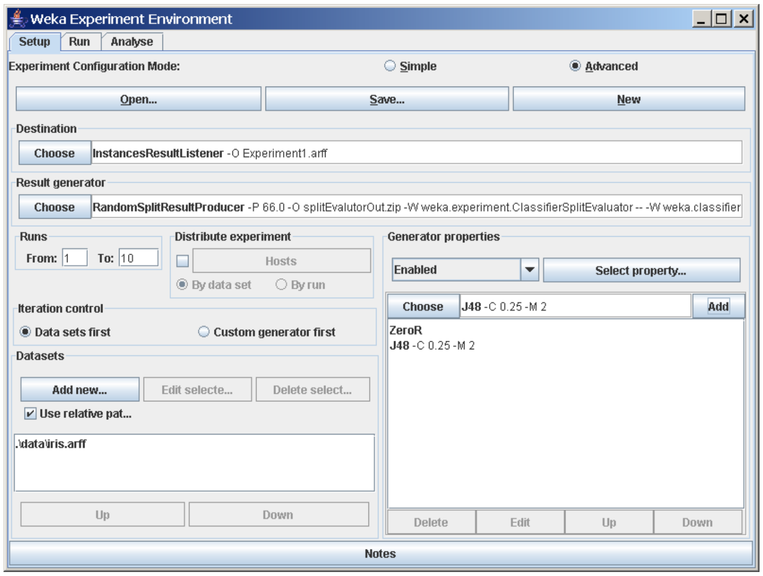

Now when the experiment is run, results are generated for both schemes.

To add additional schemes, repeat this process. To remove a scheme, select the scheme by clicking on it and then click *Delete*.

Adding Additional Datasets

The scheme(s) may be run on any number of datasets at a time. Additional datasets are added by clicking *Add new...* in the *Datasets* panel. Datasets are deleted from the experiment by selecting the dataset and then clicking *Delete Selected*.

Raw Output

The raw output generated by a scheme during an experiment can be saved to a file and then examined at a later time. Open the *ResultProducer* window by clicking on the *Result generator* panel in the *Setup* tab.

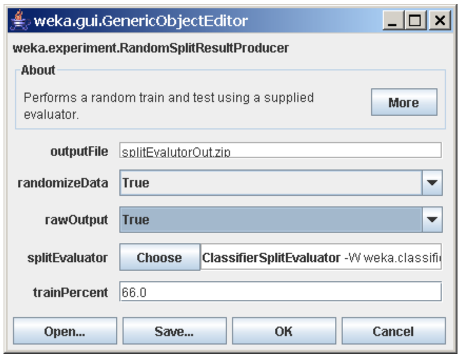

Click on *rawOutput* and select the *True* entry from the drop-down list. By default, the output is sent to the zip file *splitEvaluatorOut.zip*. The output file can be changed by clicking on the *outputFile* panel in the window. Now when the experiment is run, the result of each processing run is archived, as shown below.

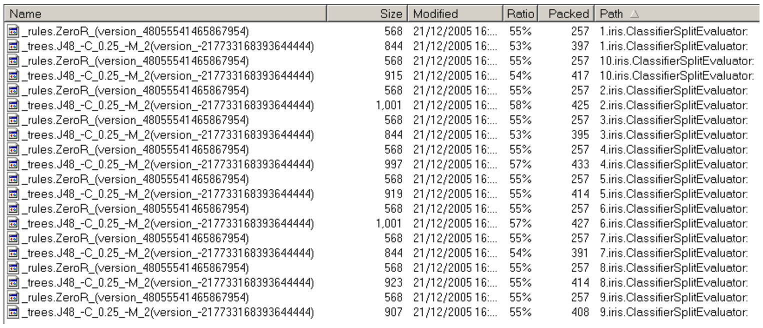

Name	Size	Modified	Ratio	Packed	Path
rules.ZeroR(version_48055541465867954)	568	21/12/2005 16:...	55%	257	1.iris.ClassifierSplitEvaluator:
trees.J48-C_0.25_-M_2(version_-217733168393644444)	844	21/12/2005 16:...	53%	397	1.iris.ClassifierSplitEvaluator:
rules.ZeroR(version_48055541465867954)	568	21/12/2005 16:...	55%	257	10.iris.ClassifierSplitEvaluator:
trees.J48-C_0.25_-M_2(version_-217733168393644444)	915	21/12/2005 16:...	54%	417	10.iris.ClassifierSplitEvaluator:
rules.ZeroR(version_48055541465867954)	568	21/12/2005 16:...	55%	257	2.iris.ClassifierSplitEvaluator:
trees.J48-C_0.25_-M_2(version_-217733168393644444)	1,001	21/12/2005 16:...	58%	425	2.iris.ClassifierSplitEvaluator:
rules.ZeroR(version_48055541465867954)	568	21/12/2005 16:...	55%	257	3.iris.ClassifierSplitEvaluator:
trees.J48-C_0.25_-M_2(version_-217733168393644444)	844	21/12/2005 16:...	53%	395	3.iris.ClassifierSplitEvaluator:
rules.ZeroR(version_48055541465867954)	568	21/12/2005 16:...	55%	257	4.iris.ClassifierSplitEvaluator:
trees.J48-C_0.25_-M_2(version_-217733168393644444)	997	21/12/2005 16:...	57%	433	4.iris.ClassifierSplitEvaluator:
rules.ZeroR(version_48055541465867954)	568	21/12/2005 16:...	55%	257	5.iris.ClassifierSplitEvaluator:
trees.J48-C_0.25_-M_2(version_-217733168393644444)	919	21/12/2005 16:...	55%	414	5.iris.ClassifierSplitEvaluator:
rules.ZeroR(version_48055541465867954)	568	21/12/2005 16:...	55%	257	6.iris.ClassifierSplitEvaluator:
trees.J48-C_0.25_-M_2(version_-217733168393644444)	1,001	21/12/2005 16:...	57%	427	6.iris.ClassifierSplitEvaluator:
rules.ZeroR(version_48055541465867954)	568	21/12/2005 16:...	55%	257	7.iris.ClassifierSplitEvaluator:
trees.J48-C_0.25_-M_2(version_-217733168393644444)	844	21/12/2005 16:...	54%	391	7.iris.ClassifierSplitEvaluator:
rules.ZeroR(version_48055541465867954)	568	21/12/2005 16:...	55%	257	8.iris.ClassifierSplitEvaluator:
trees.J48-C_0.25_-M_2(version_-217733168393644444)	923	21/12/2005 16:...	55%	414	8.iris.ClassifierSplitEvaluator:
rules.ZeroR(version_48055541465867954)	568	21/12/2005 16:...	55%	257	9.iris.ClassifierSplitEvaluator:
trees.J48-C_0.25_-M_2(version_-217733168393644444)	907	21/12/2005 16:...	55%	408	9.iris.ClassifierSplitEvaluator:

The contents of the first run are:

```
ClassifierSplitEvaluator: weka.classifiers.trees.J48 -C 0.25 -M 2(version
     -217733168393644444)Classifier model:
J48 pruned tree
------------------

petalwidth <= 0.6: Iris-setosa (33.0)
petalwidth > 0.6
|   petalwidth <= 1.5: Iris-versicolor (31.0/1.0)
|   petalwidth > 1.5: Iris-virginica (35.0/3.0)

Number of Leaves  :   3

Size of the tree :   5
```

```
Correctly Classified Instances          47               92.1569 %
Incorrectly Classified Instances         4                7.8431 %
Kappa statistic                          0.8824
Mean absolute error                      0.0723
Root mean squared error                  0.2191
Relative absolute error                 16.2754 %
Root relative squared error             46.4676 %
Total Number of Instances               51
measureTreeSize : 5.0
measureNumLeaves : 3.0
measureNumRules : 3.0
```

5.2.2.4 Other Result Producers

Cross-Validation Result Producer

To change from random train and test experiments to cross-validation experiments, click on the *Result generator* entry. At the top of the window, click on the drop-down list and select *CrossValidationResultProducer*. The window now contains parameters specific to cross-validation such as the number of partitions/folds. The experiment performs 10-fold cross-validation instead of train and test in the given example.

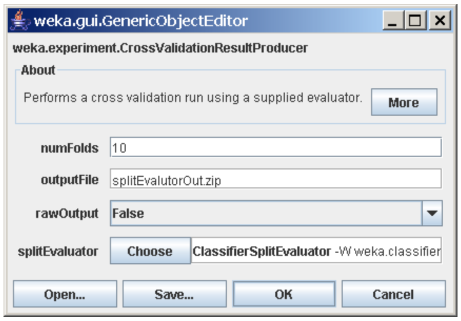

The *Result generator* panel now indicates that cross-validation will be performed. Click on *More* to generate a brief description of the *CrossValidationResultProducer*.

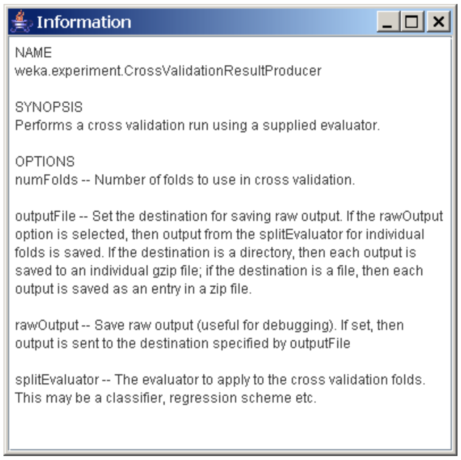

As with the *RandomSplitResultProducer*, multiple schemes can be run during cross-validation by adding them to the *Generator properties* panel.

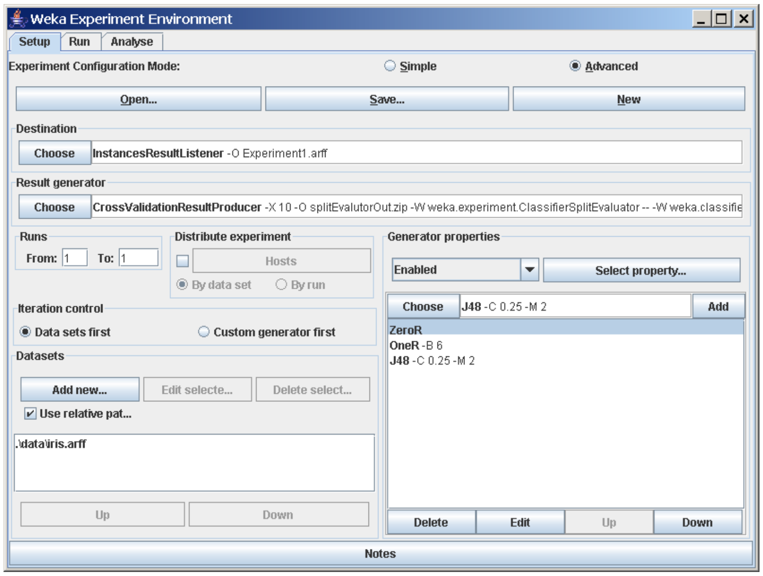

The number of runs is set to 1 in the *Setup* tab in this example, so that only one run of cross-validation for each scheme and dataset is executed.

When this experiment is analysed, the following results are generated. Note that there are 30 (1 run times 10 folds times 3 schemes) result lines processed.

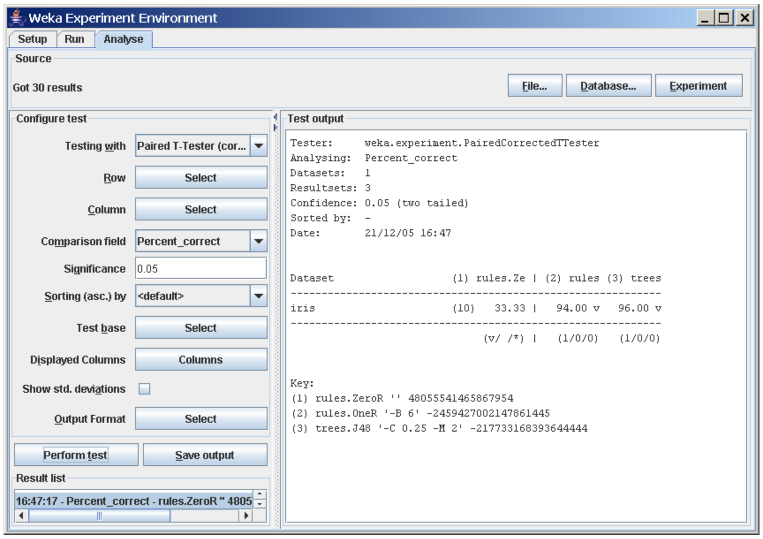

Averaging Result Producer

An alternative to the *CrossValidationResultProducer* is the *AveragingResultProducer*. This result producer takes the average of a set of runs (which are typically cross-validation runs). This result producer is identified by clicking the *Result generator* panel and then choosing the *AveragingResultProducer* from the *GenericObjectEditor*.

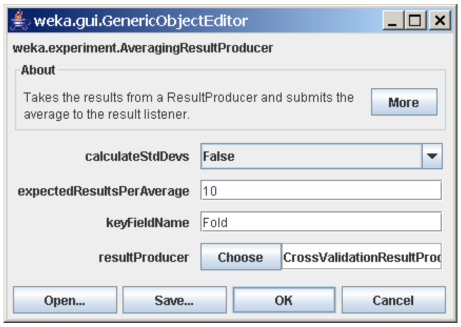

The associated help file is shown below.

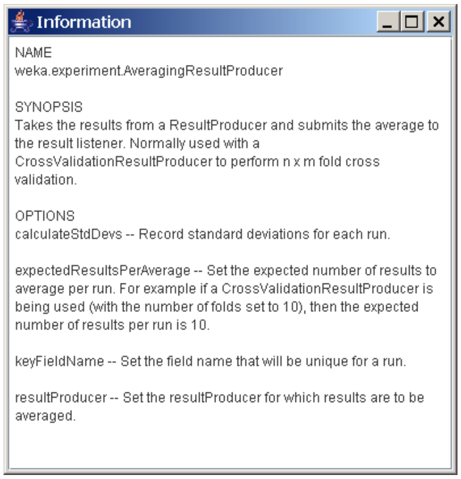

Clicking the *resultProducer* panel brings up the following window.

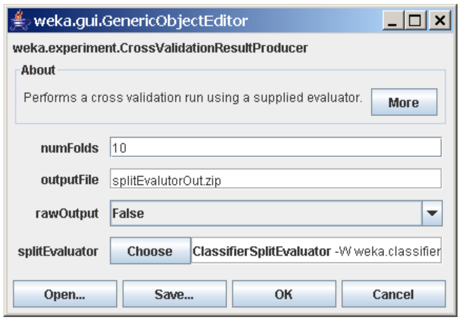

As with the other ResultProducers, additional schemes can be defined. When the *AveragingResultProducer* is used, the classifier property is located deeper in the *Generator properties* hierarchy.

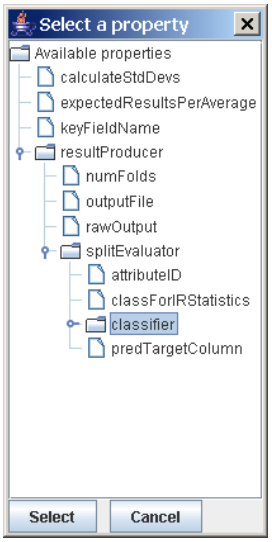

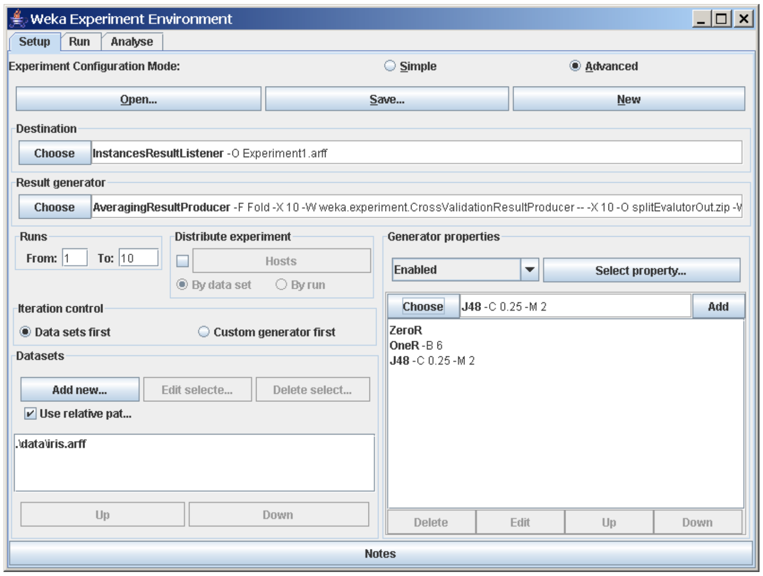

In this experiment, the `ZeroR`, `OneR`, and `J48` schemes are run 10 times with 10-fold cross-validation. Each set of 10 cross-validation folds is then averaged, producing one result line for each run (instead of one result line for each fold as in the previous example using the *CrossValidationResultProducer*) for a total of 30 result lines. If the raw output is saved, all 300 results are sent to the archive.

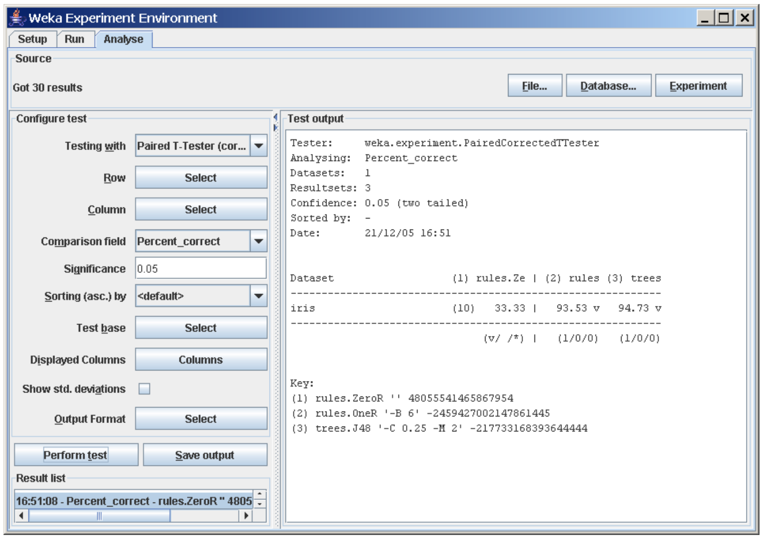
Weka Experiment Environment
Setup
Run
Analyse
Source
Got 30 results
File...
Database...
Experiment
Configure test
Testing with
Paired T-Tester (cor...
Row
Select
Column
Select
Comparison field
Percent_correct
Significance
0.05
Sorting (asc.) by
<default>
Test base
Select
Displayed Columns
Columns
Show std. deviations
Output Format
Select
Perform test
Save output
Result list
16:51:08 - Percent_correct - rules.ZeroR '' 4805
Test output
Tester: weka.experiment.PairedCorrectedTTester
Analysing: Percent_correct
Datasets: 1
Resultsets: 3
Confidence: 0.05 (two tailed)
Sorted by: -
Date: 21/12/05 16:51
Dataset (1) rules.Ze | (2) rules (3) trees
iris (10) 33.33 | 93.53 v 94.73 v
(v/ /*) | (1/0/0) (1/0/0)
Key:
(1) rules.ZeroR '' 48055541465867954
(2) rules.OneR '-B 6' -2459427002147861445
(3) trees.J48 '-C 0.25 -M 2' -217733168393644444

5.3 Cluster Experiments

Using the advanced mode of the Experimenter you can now run experiments on clustering algorithms as well as classifiers (Note: this is a new feature available with Weka 3.6.0). The main evaluation metric for this type of experiment is the log likelihood of the clusters found by each clusterer. Here is an example of setting up a cross-validation experiment using clusterers.

Choose *CrossValidationResultProducer* from the Result generator panel.

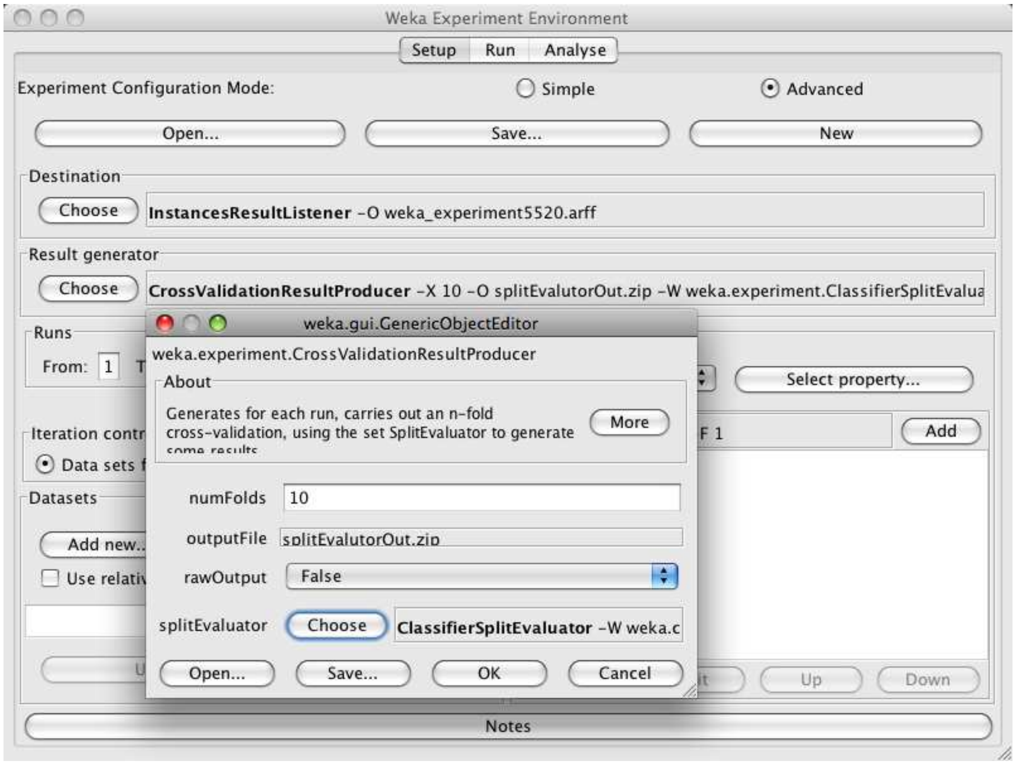

Next, choose *DensityBasedClustererSplitEvaluator* as the split evaluator to use.

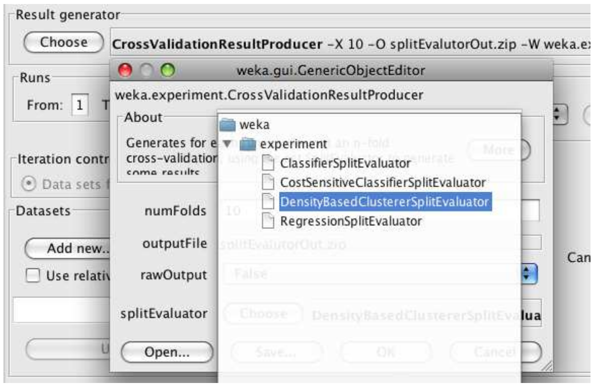

If you click on *DensityBasedClustererSplitEvaluator* you will see its options. **Note** that there is an option for removing the class column from the data. In the Experimenter, the class column is set to be the last column by default. Turn this off if you want to keep this column in the data.

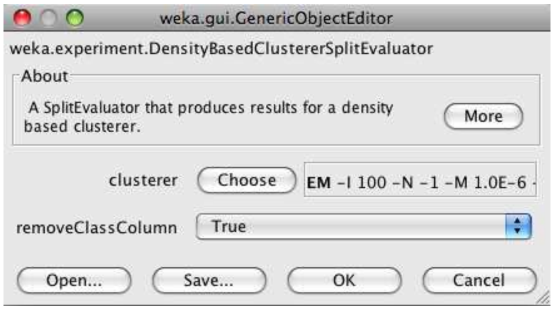

Once *DensityBasedClustererSplitEvaluator* has been selected, you will notice that the *Generator properties* have become disabled. Enable them again and expand *splitEvaluator*. Select the *clusterer* node.

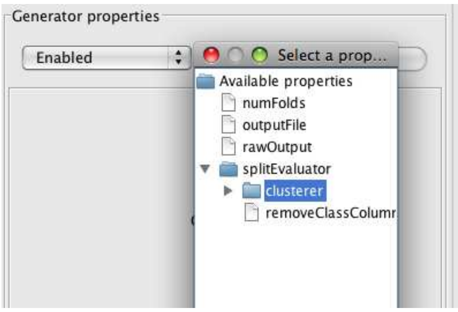

Now you will see that *EM* becomes the default clusterer and gets added to the list of schemes. You can now add/delete other clusterers.
IMPORTANT: in order to any clusterer that does not produce density estimates (i.e. most other clusterers in Weka), they will have to wrapped in the *MakeDensityBasedClusterer*.

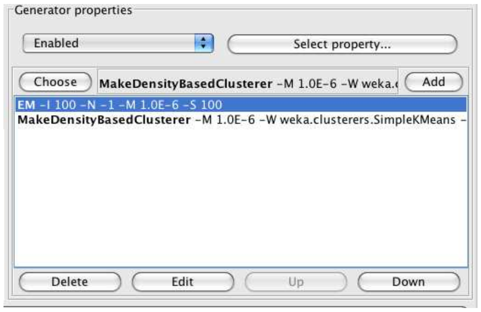

Once and experiment has been run, you can analyze results in the *Analyse* panel. In the *Comparison* field you will need to scroll down and select "Log_likelihood".

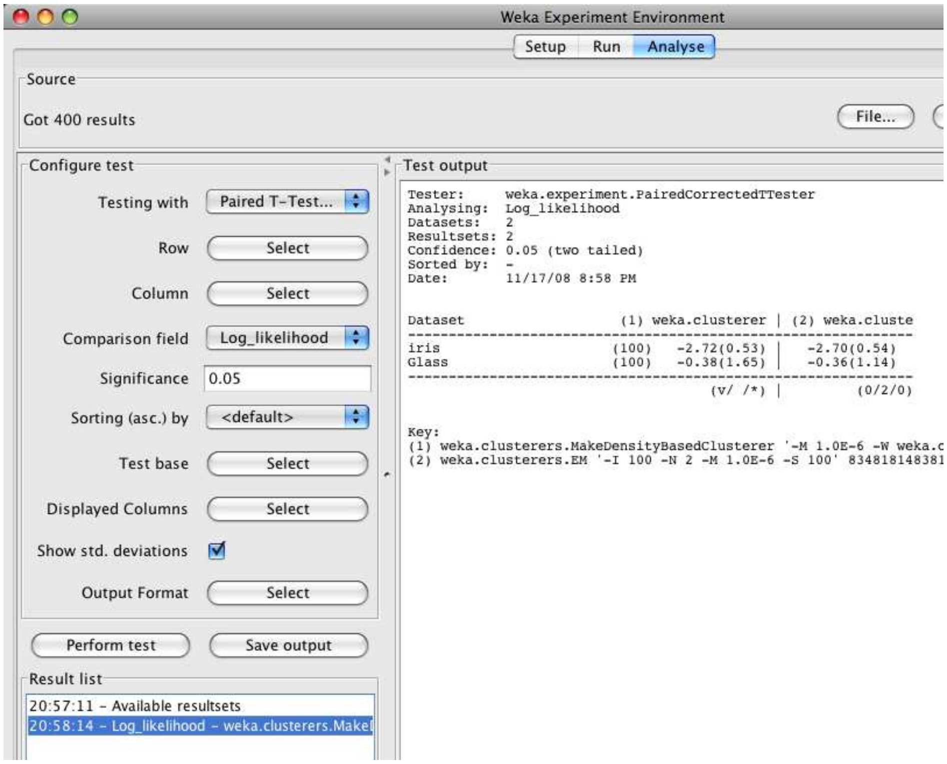

5.4 Remote Experiments

Remote experiments enable you to distribute the computing load across multiple computers. In the following we will discuss the setup and operation for HSQLDB [12] and MySQL [13].

5.4.1 Preparation

To run a remote experiment you will need:

- A database server.
- A number of computers to run remote engines on.
- To edit the remote engine policy file included in the Weka distribution to allow Java class and dataset loading from your home directory.
- An invocation of the Experimenter on a machine somewhere (any will do).

For the following examples, we assume a user called *johndoe* with this setup:

- Access to a set of computers running a flavour of Unix (pathnames need to be changed for Windows).
- The home directory is located at `/home/johndoe`.
- Weka is found in `/home/johndoe/weka`.
- Additional jar archives, i.e., JDBC drivers, are stored in `/home/johndoe/jars`.
- The directory for the datasets is `/home/johndoe/datasets`.

Note: The example policy file `remote.policy.example` is using this setup (available in `weka/experiment`[1]).

5.4.2 Database Server Setup

- HSQLDB
  - Download the JDBC driver for HSQLDB, extract the `hsqldb.jar` and place it in the directory `/home/johndoe/jars`.
  - To set up the database server, choose or create a directory to run the database server from, and start the server with:

    ```
    java -classpath /home/johndoe/jars/hsqldb.jar \
      org.hsqldb.Server \
      -database.0 experiment -dbname.0 experiment
    ```

    Note: This will start up a database with the alias "experiment" (`-dbname.0 <alias>`) and create a properties and a log file at the current location prefixed with "experiment" (`-database.0 <file>`).

[1] Weka's source code can be found in the `weka-src.jar` archive or obtained from Subversion [11].

- MySQL

  We won't go into the details of setting up a MySQL server, but this is rather straightforward and includes the following steps:

  – Download a suitable version of MySQL for your server machine.
  – Install and start the MySQL server.
  – Create a database - for our example we will use `experiment` as database name.
  – Download the appropriate JDBC driver, extract the JDBC jar and place it as `mysql.jar` in `/home/johndoe/jars`.

5.4.3 Remote Engine Setup

- First, set up a directory for scripts and policy files:

  ```
  /home/johndoe/remote_engine
  ```

- Unzip the `remoteExperimentServer.jar` (from the Weka distribution; or build it from the sources[2] with `ant remotejar`) into a temporary directory.

- Next, copy `remoteEngine.jar` and `remote.policy.example` to the `/home/johndoe/remote_engine` directory.

- Create a script, called `/home/johndoe/remote_engine/startRemoteEngine`, with the following content (don't forget to make it executable with `chmod a+x startRemoteEngine` when you are on Linux/Unix):

  – HSQLDB

  ```
  java -Xmx256m \
    -classpath /home/johndoe/jars/hsqldb.jar:remoteEngine.jar \
    -Djava.security.policy=remote.policy \
    weka.experiment.RemoteEngine &
  ```

  – MySQL

  ```
  java -Xmx256m \
    -classpath /home/johndoe/jars/mysql.jar:remoteEngine.jar \
    -Djava.security.policy=remote.policy \
    weka.experiment.RemoteEngine &
  ```

- Now we will start the remote engines that run the experiments on the remote computers (note that the same version of Java must be used for the Experimenter and remote engines):

  – Rename the `remote.policy.example` file to `remote.policy`.
  – For each machine you want to run a remote engine on:
    * `ssh` to the machine.

[2] Weka's source code can be found in the `weka-src.jar` archive or obtained from Subversion [11].

  * cd to /home/johndoe/remote_engine.
  * Run /home/johndoe/startRemoteEngine (to enable the remote engines to use more memory, modify the -Xmx option in the startRemoteEngine script) .

5.4.4 Configuring the Experimenter

Now we will run the Experimenter:

- HSQLDB
  - Copy the *DatabaseUtils.props.hsql* file from weka/experiment in the weka.jar archive to the /home/johndoe/remote_engine directory and rename it to *DatabaseUtils.props*.
  - Edit this file and change the "jdbcURL=jdbc:hsqldb:hsql://server_name/database_name" entry to include the name of the machine that is running your database server (e.g., jdbcURL=jdbc:hsqldb:hsql://dodo.company.com/experiment).
  - Now start the Experimenter (inside this directory):

```
java \
  -cp /home/johndoe/jars/hsqldb.jar:remoteEngine.jar:/home/johndoe/weka/weka.jar \
  -Djava.rmi.server.codebase=file:/home/johndoe/weka/weka.jar \
  weka.gui.experiment.Experimenter
```

- MySQL
  - Copy the *DatabaseUtils.props.mysql* file from weka/experiment in the weka.jar archive to the /home/johndoe/remote_engine directory and rename it to *DatabaseUtils.props*.
  - Edit this file and change the "jdbcURL=jdbc:mysql://server_name:3306/database_name" entry to include the name of the machine that is running your database server and the name of the database the result will be stored in (e.g., jdbcURL=jdbc:mysql://dodo.company.com:3306/experiment).
  - Now start the Experimenter (inside this directory):

```
java \
  -cp /home/johndoe/jars/mysql.jar:remoteEngine.jar:/home/johndoe/weka/weka.jar \
  -Djava.rmi.server.codebase=file:/home/johndoe/weka/weka.jar \
  weka.gui.experiment.Experimenter
```

Note: the database name *experiment* can still be modified in the Experimenter, this is just the default setup.

Now we will configure the experiment:

- First of all select the *Advanced* mode in the *Setup* tab
- Now choose the *DatabaseResultListener* in the *Destination* panel. Configure this result producer:
  - HSQLDB
    Supply the value **sa** for the username and leave the password empty.

  - MySQL
    Provide the username and password that you need for connecting to the database.

- From the *Result generator* panel choose either the *CrossValidationResultProducer* or the *RandomSplitResultProducer* (these are the most commonly used ones) and then configure the remaining experiment details (e.g., datasets and classifiers).

- Now enable the *Distribute Experiment* panel by checking the tick box.

- Click on the *Hosts* button and enter the names of the machines that you started remote engines on (`<Enter>` adds the host to the list).

- You can choose to distribute by run or dataset.

- Save your experiment configuration.

- Now start your experiment as you would do normally.

- Check your results in the *Analyse* tab by clicking either the *Database* or *Experiment* buttons.

5.4.5 Multi-core support

If you want to utilize all the cores on a multi-core machine, then you can do so with Weka. All you have to do, is define the port alongside the hostname in the Experimenter (format: `hostname:port`) and then start the RemoteEngine with the `-p` option, specifying the port to listen on.

5.4.6 Troubleshooting

- If you get an error at the start of an experiment that looks a bit like this:

  ```
  01:13:19: RemoteExperiment (//blabla.company.com/RemoteEngine)
  (sub)experiment (datataset vineyard.arff) failed :
  java.sql.SQLException: Table already exists: EXPERIMENT_INDEX
  in statement [CREATE TABLE Experiment_index ( Experiment_type
  LONGVARCHAR, Experiment_setup LONGVARCHAR, Result_table INT )]
  ```

  ```
  01:13:19: dataset :vineyard.arff RemoteExperiment
  (//blabla.company.com/RemoteEngine) (sub)experiment (datataset
  vineyard.arff) failed : java.sql.SQLException: Table already
  exists: EXPERIMENT_INDEX in statement [CREATE TABLE
  Experiment_index ( Experiment_type LONGVARCHAR, Experiment_setup
  LONGVARCHAR, Result_table INT )]. Scheduling for execution on
  another host.
  ```

  then do not panic - this happens because multiple remote machines are trying to create the same table and are temporarily locked out - this will resolve itself so just leave your experiment running - in fact, it is a sign that the experiment is working!

- If you serialized an experiment and then modify your *DatabaseUtils.props* file due to an error (e.g., a missing type-mapping), the Experimenter will use the *DatabaseUtils.props* you had *at the time you serialized the experiment*. Keep in mind that the serialization process also serializes the *DatabaseUtils* class and therefore stored your props-file! This is another reason for storing your experiments as XML and not in the properietary binary format the Java serialization produces.

- Using a corrupt or incomplete *DatabaseUtils.props* file can cause peculiar interface errors, for example disabling the use of the "User" button alongside the database URL. If in doubt copy a clean *DatabaseUtils.props* from Subversion [11].

- If you get `NullPointerException at java.util.Hashtable.get()` in the Remote Engine do not be alarmed. This will have no effect on the results of your experiment.

5.5 Analysing Results

5.5.1 Setup

Weka includes an experiment analyser that can be used to analyse the results of experiments (in this example, the results were sent to an *InstancesResultListener*). The experiment shown below uses 3 schemes, `ZeroR`, `OneR`, and `J48`, to classify the Iris data in an experiment using 10 train and test runs, with 66% of the data used for training and 34% used for testing.

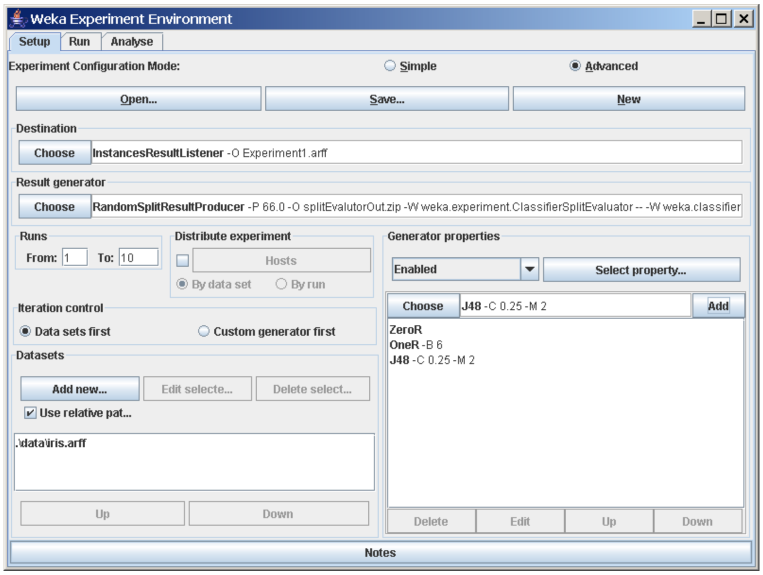

After the experiment setup is complete, run the experiment. Then, to analyse the results, select the *Analyse* tab at the top of the Experiment Environment window.

Click on *Experiment* to analyse the results of the current experiment.

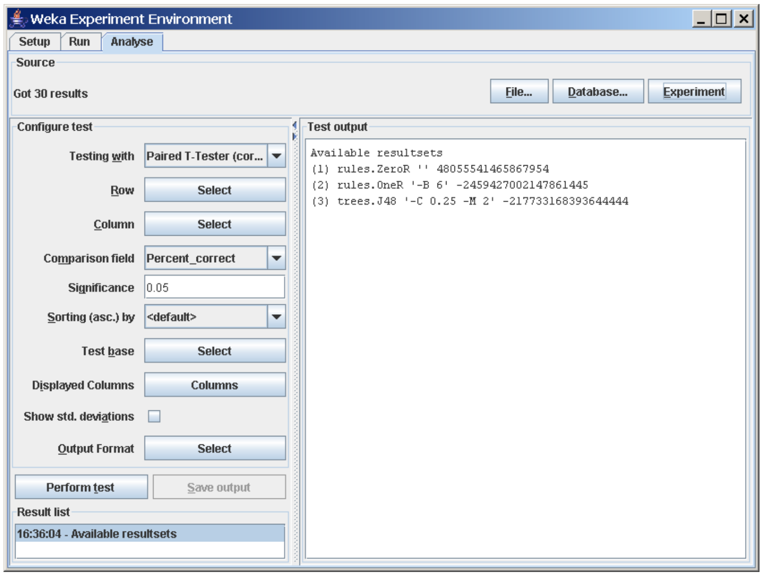

The number of result lines available (*Got 30 results*) is shown in the *Source* panel. This experiment consisted of 10 runs, for 3 schemes, for 1 dataset, for a total of 30 result lines. Results can also be loaded from an earlier experiment file by clicking *File* and loading the appropriate *.arff* results file. Similarly, results sent to a database (using the *DatabaseResultListener*) can be loaded from the database.

Select the *Percent_correct* attribute from the *Comparison field* and click *Perform* test to generate a comparison of the 3 schemes.

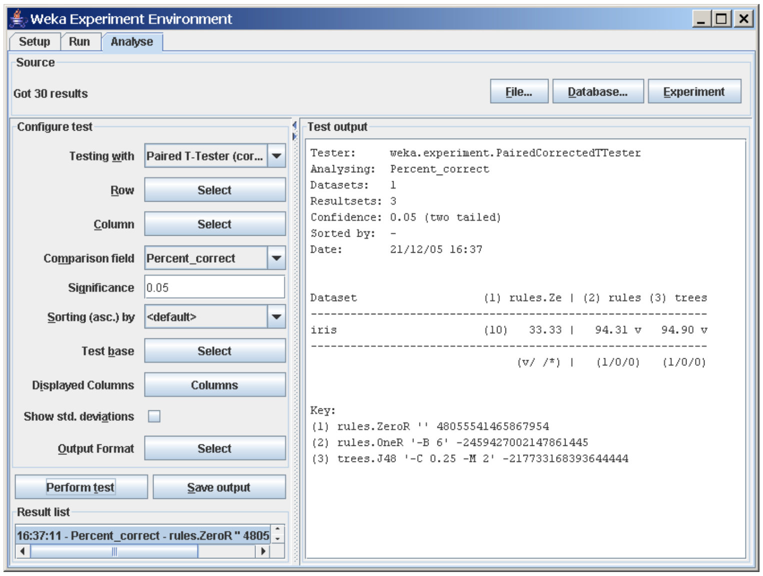

The schemes used in the experiment are shown in the columns and the datasets used are shown in the rows.

The percentage correct for each of the 3 schemes is shown in each dataset row: 33.33% for `ZeroR`, 94.31% for `OneR`, and 94.90% for `J48`. The annotation v or * indicates that a specific result is statistically better (v) or worse (*) than the baseline scheme (in this case, `ZeroR`) at the significance level specified (currently 0.05). The results of both `OneR` and `J48` are statistically better than the baseline established by `ZeroR`. At the bottom of each column after the first column is a count (xx/ yy/ zz) of the number of times that the scheme was better than (xx), the same as (yy), or worse than (zz), the baseline scheme on the datasets used in the experiment. In this example, there was only one dataset and `OneR` was better than `ZeroR` once and never equivalent to or worse than `ZeroR` (1/0/0); `J48` was also better than `ZeroR` on the dataset.

The standard deviation of the attribute being evaluated can be generated by selecting the *Show std. deviations* check box and hitting *Perform test* again. The value *(10)* at the beginning of the *iris* row represents the number of estimates that are used to calculate the standard deviation (the number of runs in this case).

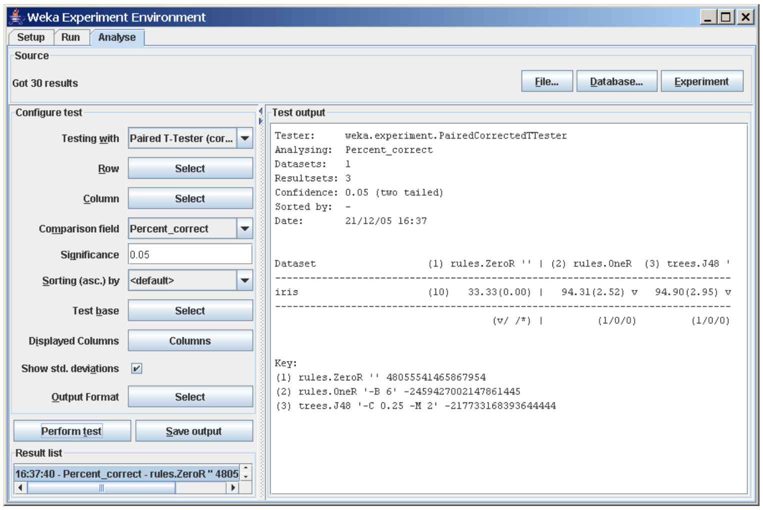

Selecting *Number_correct* as the comparison field and clicking *Perform test* generates the average number correct (out of 50 test patterns - 33% of 150 patterns in the Iris dataset).

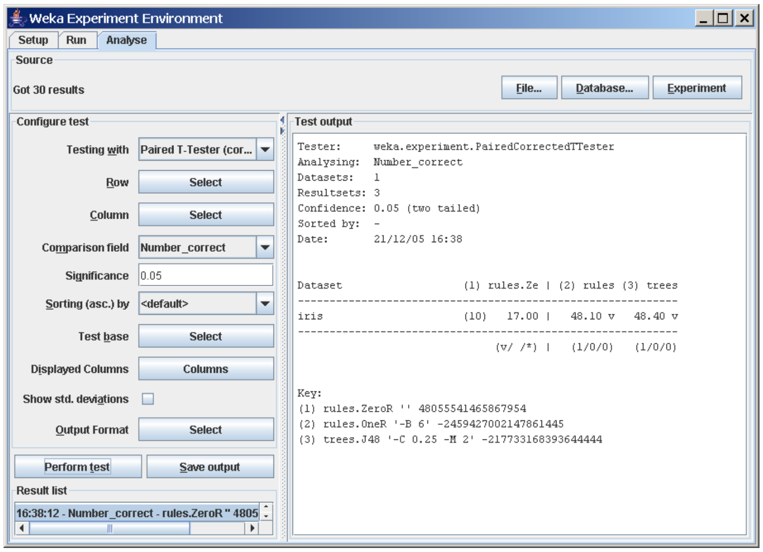

Clicking on the button for the *Output format* leads to a dialog that lets you choose the precision for the *mean* and the *std. deviations*, as well as the format of the output. Checking the *Show Average* checkbox adds an additional line to the output listing the average of each column. With the *Remove filter classnames* checkbox one can remove the filter name and options from processed datasets (filter names in Weka can be quite lengthy).

The following formats are supported:

- CSV
- GNUPlot
- HTML

- LaTeX
- Plain text (default)
- Significance only

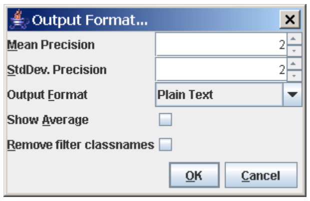

5.5.2 Saving the Results

The information displayed in the *Test output* panel is controlled by the currently-selected entry in the *Result list* panel. Clicking on an entry causes the results corresponding to that entry to be displayed.

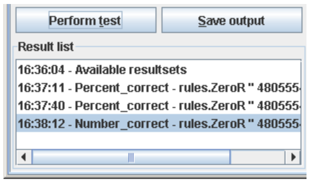

The results shown in the *Test output* panel can be saved to a file by clicking *Save output*. Only one set of results can be saved at a time but Weka permits the user to save all results to the same file by saving them one at a time and using the *Append* option instead of the *Overwrite* option for the second and subsequent saves.

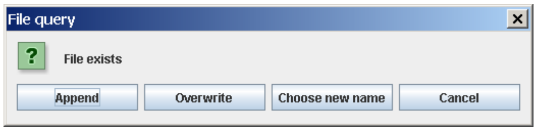

5.5.3 Changing the Baseline Scheme

The baseline scheme can be changed by clicking *Select base...* and then selecting the desired scheme. Selecting the `OneR` scheme causes the other schemes to be compared individually with the `OneR` scheme.

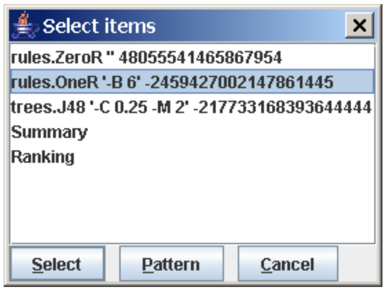

If the test is performed on the *Percent_correct* field with `OneR` as the base scheme, the system indicates that there is no statistical difference between the results for `OneR` and `J48`. There is however a statistically significant difference between `OneR` and `ZeroR`.

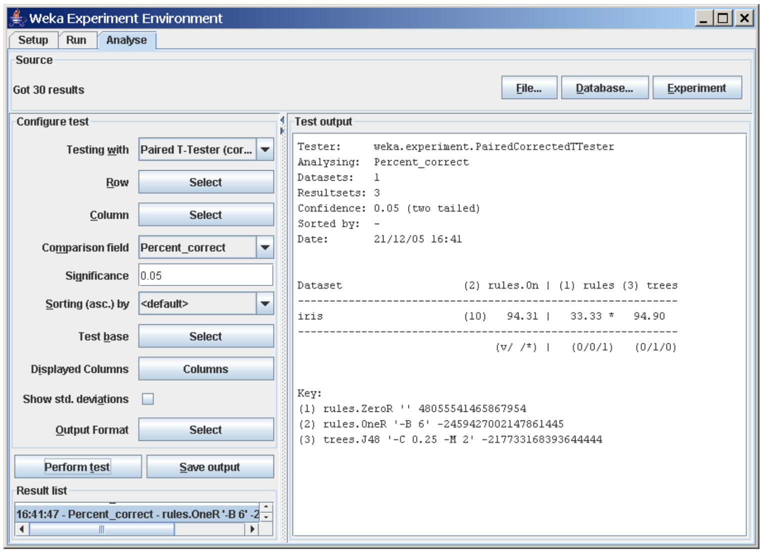

5.5.4 Statistical Significance

The term *statistical significance* used in the previous section refers to the result of a pair-wise comparison of schemes using either a standard *T-Test* or the corrected resampled *T-Test* [9]. The latter test is the default, because the standard *T-Test* can generate too many significant differences due to dependencies in the estimates (in particular when anything other than one run of an x-fold cross-validation is used). For more information on the *T-Test*, consult the Weka book [1] or an introductory statistics text. As the significance level is decreased, the confidence in the conclusion increases.

In the current experiment, there is not a statistically significant difference between the `OneR` and `J48` schemes.

5.5.5 Summary Test

Selecting *Summary* from *Test base* and performing a test causes the following information to be generated.

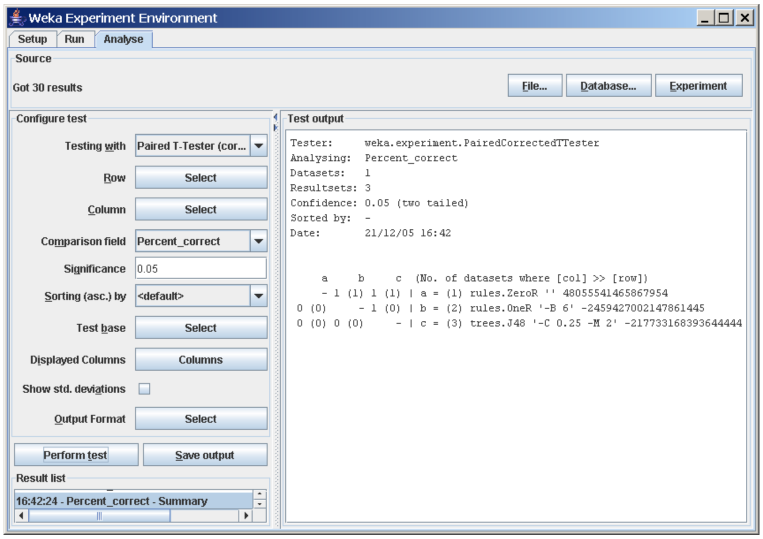

In this experiment, the first row (- 1 1) indicates that column *b* (`OneR`) is better than row *a* (`ZeroR`) and that column *c* (`J48`) is also better than row *a*. The number in brackets represents the number of significant wins for the column with regard to the row. A 0 means that the scheme in the corresponding column did not score a single (significant) win with regard to the scheme in the row.

5.5.6 Ranking Test

Selecting *Ranking* from *Test base* causes the following information to be generated.

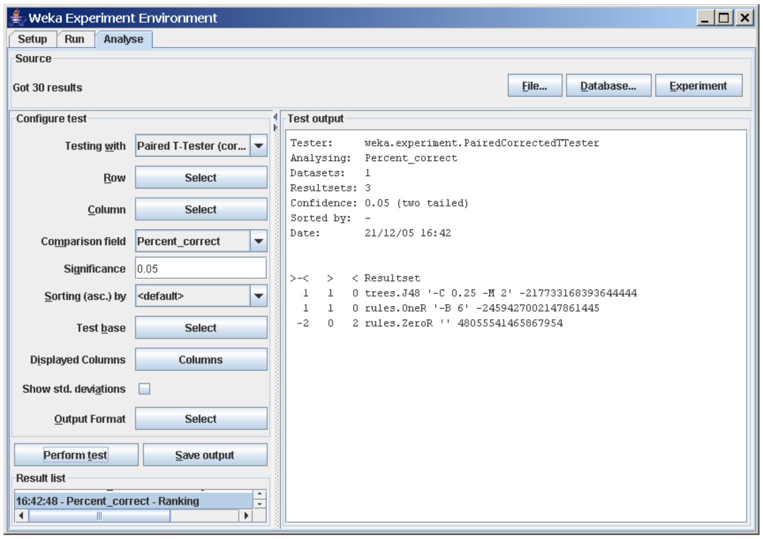

The ranking test ranks the schemes according to the total number of significant wins (>) and losses (<) against the other schemes. The first column ($> - <$) is the difference between the number of wins and the number of losses. This difference is used to generate the ranking.

Chapter 6

KnowledgeFlow

6.1 Introduction

The KnowledgeFlow provides an alternative to the Explorer as a graphical front end to WEKA's core algorithms. The KnowledgeFlow is a work in progress so some of the functionality from the Explorer is not yet available. On the other hand, there are things that can be done in the KnowledgeFlow but not in the Explorer.

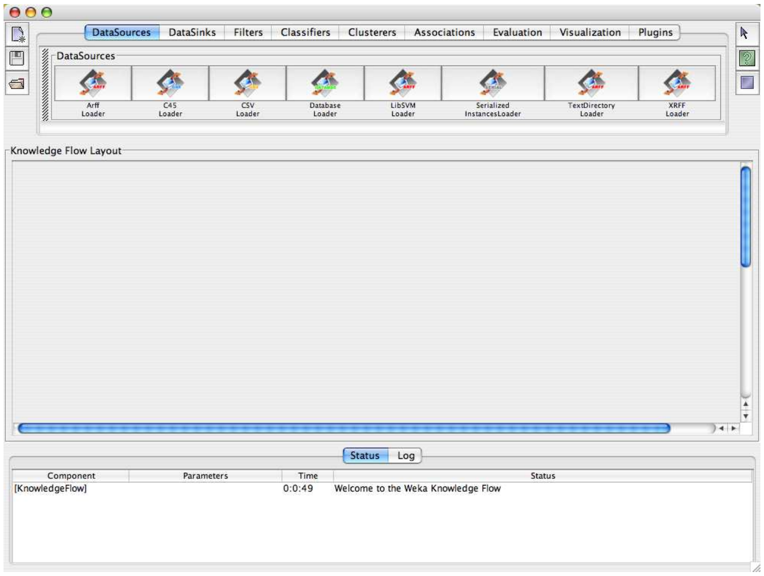

The KnowledgeFlow presents a *data-flow* inspired interface to WEKA. The user can select WEKA components from a tool bar, place them on a layout canvas and connect them together in order to form a *knowledge flow* for processing and analyzing data. At present, all of WEKA's classifiers, filters, clusterers, loaders and savers are available in the KnowledgeFlow along with some extra tools.

The KnowledgeFlow can handle data either incrementally or in batches (the Explorer handles batch data only). Of course learning from data incremen-

tally requires a classifier that can be updated on an instance by instance basis. Currently in WEKA there are ten classifiers that can handle data incrementally:

- AODE
- IB1
- IBk
- KStar
- NaiveBayesMultinomialUpdateable
- NaiveBayesUpdateable
- NNge
- Winnow

And two of them are meta classifiers:

- *RacedIncrementalLogitBoost* - that can use of any regression base learner to learn from discrete class data incrementally.
- *LWL* - locally weighted learning.

6.2 Features

The KnowledgeFlow offers the following features:

- intuitive data flow style layout
- process data in batches or incrementally
- process multiple batches or streams in parallel (each separate flow executes in its own thread)
- chain filters together
- view models produced by classifiers for each fold in a cross validation
- visualize performance of incremental classifiers during processing (scrolling plots of classification accuracy, RMS error, predictions etc.)
- plugin facility for allowing easy addition of new components to the KnowledgeFlow

6.3 Components

Components available in the KnowledgeFlow:

6.3.1 DataSources

All of WEKA's loaders are available.

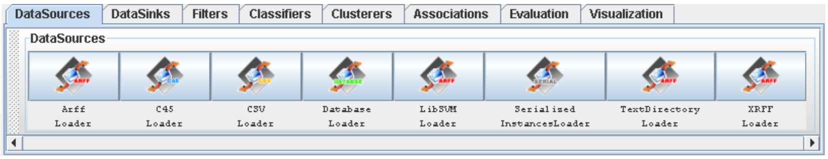

6.3.2 DataSinks

All of WEKA's savers are available.

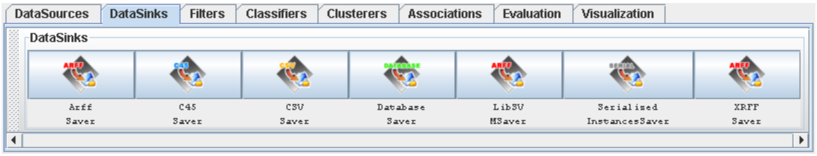

6.3.3 Filters

All of WEKA's filters are available.

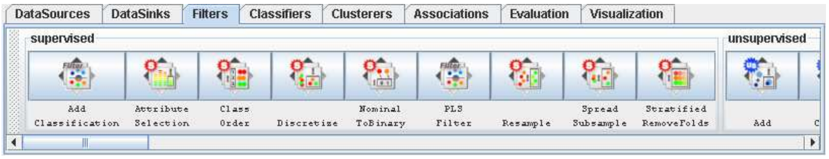

6.3.4 Classifiers

All of WEKA's classifiers are available.

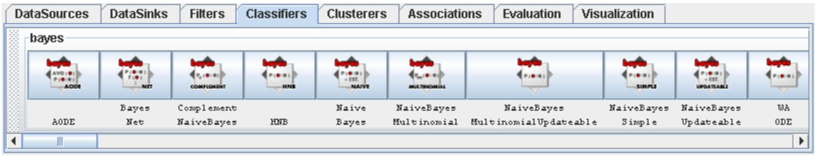

6.3.5 Clusterers

All of WEKA's clusterers are available.

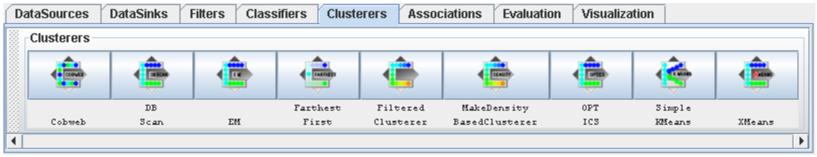

6.3.6 Evaluation

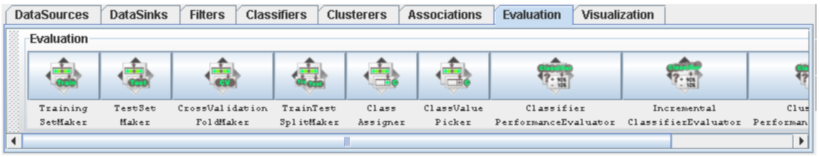

- *TrainingSetMaker* - make a data set into a training set.
- *TestSetMaker* - make a data set into a test set.
- *CrossValidationFoldMaker* - split any data set, training set or test set into folds.
- *TrainTestSplitMaker* - split any data set, training set or test set into a training set and a test set.
- *ClassAssigner* - assign a column to be the class for any data set, training set or test set.
- *ClassValuePicker* - choose a class value to be considered as the "positive" class. This is useful when generating data for ROC style curves (see *ModelPerformanceChart* below and example 6.4.2).
- *ClassifierPerformanceEvaluator* - evaluate the performance of batch trained/tested classifiers.
- *IncrementalClassifierEvaluator* - evaluate the performance of incrementally trained classifiers.
- *ClustererPerformanceEvaluator* - evaluate the performance of batch trained/tested clusterers.
- *PredictionAppender* - append classifier predictions to a test set. For discrete class problems, can either append predicted class labels or probability distributions.

6.3.7 Visualization

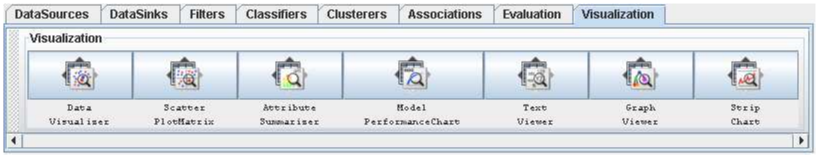

- *DataVisualizer* - component that can pop up a panel for visualizing data in a single large 2D scatter plot.
- *ScatterPlotMatrix* - component that can pop up a panel containing a matrix of small scatter plots (clicking on a small plot pops up a large scatter plot).
- *AttributeSummarizer* - component that can pop up a panel containing a matrix of histogram plots - one for each of the attributes in the input data.
- *ModelPerformanceChart* - component that can pop up a panel for visualizing threshold (i.e. ROC style) curves.
- *TextViewer* - component for showing textual data. Can show data sets, classification performance statistics etc.
- *GraphViewer* - component that can pop up a panel for visualizing tree based models.
- *StripChart* - component that can pop up a panel that displays a scrolling plot of data (used for viewing the online performance of incremental classifiers).

6.4 Examples

6.4.1 Cross-validated J48

Setting up a flow to load an ARFF file (batch mode) and perform a cross-validation using J48 (WEKA's C4.5 implementation).

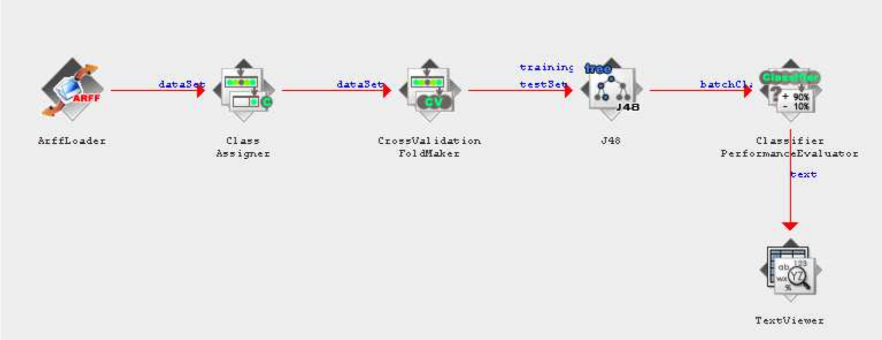

- Click on the DataSources tab and choose *ArffLoader* from the toolbar (the mouse pointer will change to a *cross hairs*).

- Next place the ArffLoader component on the layout area by clicking somewhere on the layout (a copy of the ArffLoader icon will appear on the layout area).

- Next specify an ARFF file to load by first right clicking the mouse over the ArffLoader icon on the layout. A pop-up menu will appear. Select *Configure* under *Edit* in the list from this menu and browse to the location of your ARFF file.

- Next click the *Evaluation* tab at the top of the window and choose the *ClassAssigner* (allows you to choose which column to be the class) component from the toolbar. Place this on the layout.

- Now connect the ArffLoader to the ClassAssigner: first right click over the ArffLoader and select the *dataSet* under *Connections* in the menu. A *rubber band* line will appear. Move the mouse over the ClassAssigner component and left click - a red line labeled *dataSet* will connect the two components.

- Next right click over the ClassAssigner and choose *Configure* from the menu. This will pop up a window from which you can specify which column is the class in your data (last is the default).

- Next grab a *CrossValidationFoldMaker* component from the Evaluation toolbar and place it on the layout. Connect the ClassAssigner to the CrossValidationFoldMaker by right clicking over *ClassAssigner* and selecting *dataSet* from under *Connections* in the menu.

- Next click on the *Classifiers* tab at the top of the window and scroll along the toolbar until you reach the *J48* component in the *trees* section. Place a J48 component on the layout.

- Connect the CrossValidationFoldMaker to J48 TWICE by first choosing *trainingSet* and then *testSet* from the pop-up menu for the CrossValidationFoldMaker.

- Next go back to the *Evaluation* tab and place a *ClassifierPerformanceEvaluator* component on the layout. Connect J48 to this component by selecting the *batchClassifier* entry from the pop-up menu for J48.

- Next go to the *Visualization* toolbar and place a *TextViewer* component on the layout. Connect the ClassifierPerformanceEvaluator to the TextViewer by selecting the *text* entry from the pop-up menu for ClassifierPerformanceEvaluator.

- Now start the flow executing by selecting *Start loading* from the pop-up menu for ArffLoader. Depending on how big the data set is and how long cross-validation takes you will see some animation from some of the icons in the layout (J48's tree will *grow* in the icon and the ticks will animate on the ClassifierPerformanceEvaluator). You will also see some progress information in the *Status* bar and *Log* at the bottom of the window.

When finished you can view the results by choosing *Show results* from the pop-up menu for the *TextViewer* component.

Other cool things to add to this flow: connect a *TextViewer* and/or a *GraphViewer* to J48 in order to view the textual or graphical representations of the trees produced for each fold of the cross validation (this is something that is not possible in the Explorer).

6.4.2 Plotting multiple ROC curves

The KnowledgeFlow can draw multiple ROC curves in the same plot window, something that the Explorer cannot do. In this example we use *J48* and *RandomForest* as classifiers. This example can be found on the *WekaWiki* as well [14].

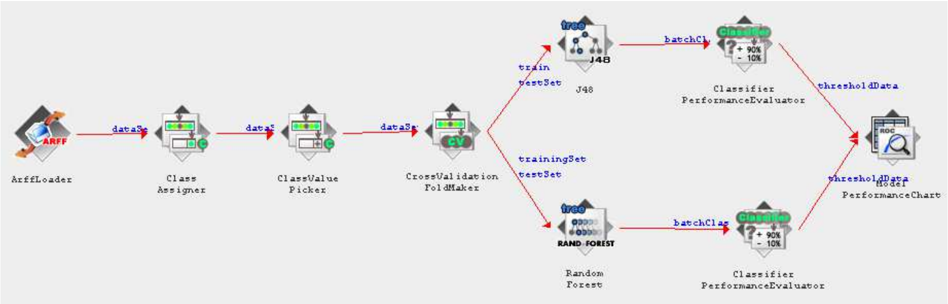

- Click on the DataSources tab and choose *ArffLoader* from the toolbar (the mouse pointer will change to a *cross hairs*).

- Next place the ArffLoader component on the layout area by clicking somewhere on the layout (a copy of the ArffLoader icon will appear on the layout area).

- Next specify an ARFF file to load by first right clicking the mouse over the ArffLoader icon on the layout. A pop-up menu will appear. Select *Configure* under *Edit* in the list from this menu and browse to the location of your ARFF file.

- Next click the *Evaluation* tab at the top of the window and choose the *ClassAssigner* (allows you to choose which column to be the class) component from the toolbar. Place this on the layout.

- Now connect the ArffLoader to the ClassAssigner: first right click over the ArffLoader and select the *dataSet* under *Connections* in the menu. A *rubber band* line will appear. Move the mouse over the ClassAssigner component and left click - a red line labeled *dataSet* will connect the two components.

- Next right click over the ClassAssigner and choose *Configure* from the menu. This will pop up a window from which you can specify which column is the class in your data (last is the default).

- Next choose the *ClassValuePicker* (allows you to choose which class label to be evaluated in the ROC) component from the toolbar. Place this on the layout and right click over *ClassAssigner* and select *dataSet* from under *Connections* in the menu and connect it with the *ClassValuePicker*.

- Next grab a *CrossValidationFoldMaker* component from the Evaluation toolbar and place it on the layout. Connect the ClassAssigner to the CrossValidationFoldMaker by right clicking over *ClassAssigner* and selecting *dataSet* from under *Connections* in the menu.

- Next click on the *Classifiers* tab at the top of the window and scroll along the toolbar until you reach the *J48* component in the *trees* section. Place a J48 component on the layout.
- Connect the CrossValidationFoldMaker to J48 TWICE by first choosing *trainingSet* and then *testSet* from the pop-up menu for the CrossValidationFoldMaker.
- Repeat these two steps with the RandomForest classifier.
- Next go back to the *Evaluation* tab and place a *ClassifierPerformanceEvaluator* component on the layout. Connect J48 to this component by selecting the *batchClassifier* entry from the pop-up menu for J48. Add another *ClassifierPerformanceEvaluator* for RandomForest and connect them via *batchClassifier* as well.
- Next go to the *Visualization* toolbar and place a *ModelPerformanceChart* component on the layout. Connect both ClassifierPerformanceEvaluators to the ModelPerformanceChart by selecting the *thresholdData* entry from the pop-up menu for ClassifierPerformanceEvaluator.
- Now start the flow executing by selecting *Start loading* from the pop-up menu for ArffLoader. Depending on how big the data set is and how long cross validation takes you will see some animation from some of the icons in the layout. You will also see some progress information in the *Status* bar and *Log* at the bottom of the window.
- Select *Show plot* from the popup-menu of the *ModelPerformanceChart* under the *Actions* section.

Here are the two ROC curves generated from the UCI dataset *credit-g*, evaluated on the class label *good*:

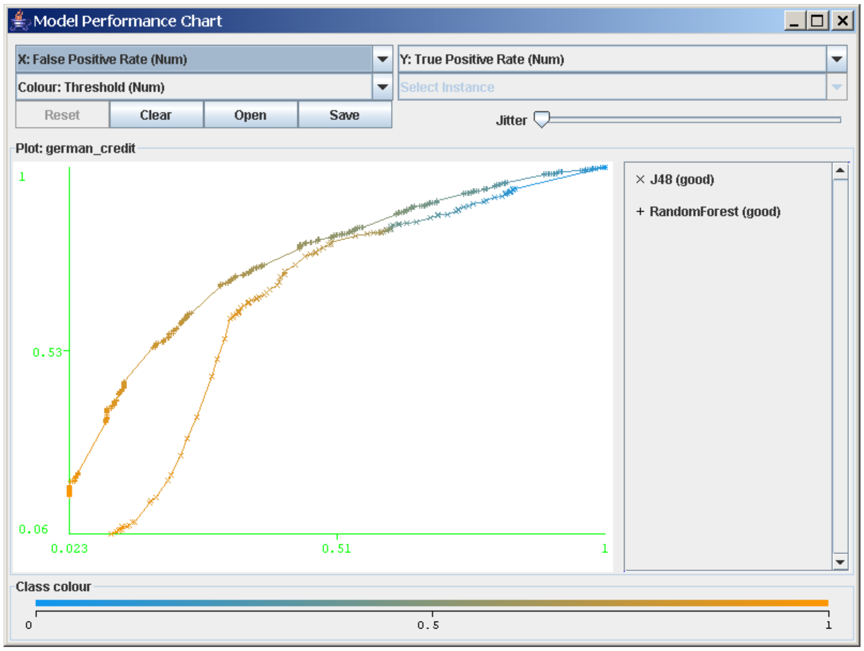

6.4.3 Processing data incrementally

Some classifiers, clusterers and filters in Weka can handle data incrementally in a streaming fashion. Here is an example of training and testing *naive Bayes* incrementally. The results are sent to a *TextViewer* and predictions are plotted by a *StripChart* component.

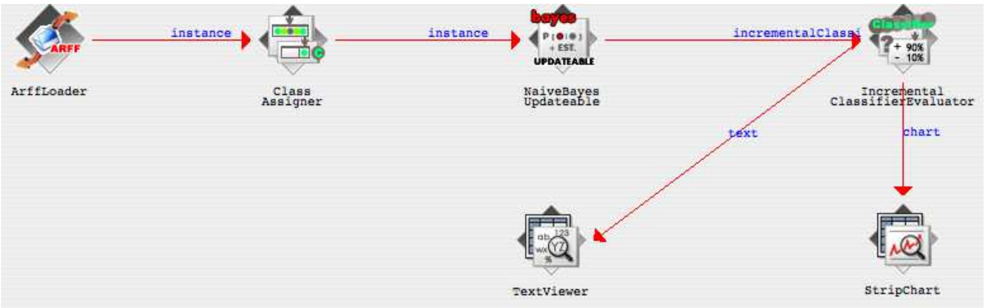

- Click on the DataSources tab and choose *ArffLoader* from the toolbar (the mouse pointer will change to a *cross hairs*).
- Next place the ArffLoader component on the layout area by clicking somewhere on the layout (a copy of the ArffLoader icon will appear on the layout area).
- Next specify an ARFF file to load by first right clicking the mouse over the ArffLoader icon on the layout. A pop-up menu will appear. Select *Configure* under *Edit* in the list from this menu and browse to the location of your ARFF file.
- Next click the *Evaluation* tab at the top of the window and choose the *ClassAssigner* (allows you to choose which column to be the class) component from the toolbar. Place this on the layout.
- Now connect the ArffLoader to the ClassAssigner: first right click over the ArffLoader and select the *dataSet* under *Connections* in the menu. A *rubber band* line will appear. Move the mouse over the ClassAssigner component and left click - a red line labeled *dataSet* will connect the two components.
- Next right click over the *ClassAssigner* and choose *Configure* from the menu. This will pop up a window from which you can specify which column is the class in your data (last is the default).
- Now grab a *NaiveBayesUpdateable* component from the *bayes* section of the *Classifiers* panel and place it on the layout.
- Next connect the *ClassAssigner* to *NaiveBayesUpdateable* using a *instance* connection.
- Next place an *IncrementalClassiferEvaluator* from the *Evaluation* panel onto the layout and connect *NaiveBayesUpdateable* to it using a *incrementalClassifier* connection.

- Next place a *TextViewer* component from the *Visualization* panel on the Layout. Connect the *IncrementalClassifierEvaluator* to it using a *text* connection.

- Next place a *StripChart* component from the *Visualization* panel on the layout and connect *IncrementalClassifierEvaluator* to it using a *chart* connection.

- Display the *StripChart's* chart by right-clicking over it and choosing *Show chart* from the pop-up menu. Note: the *StripChart* can be configured with options that control how often data points and labels are displayed.

- Finally, start the flow by right-clicking over the *ArffLoader* and selecting *Start loading* from the pop-up menu.

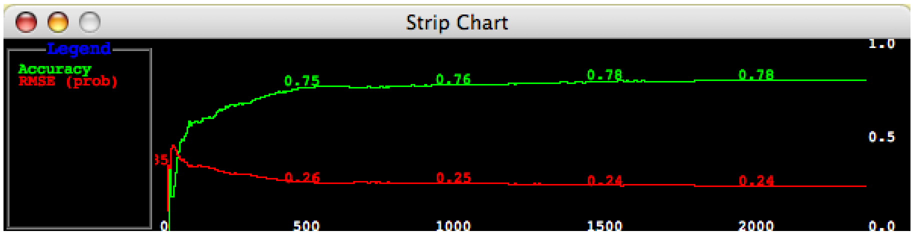

Note that, in this example, a prediction is obtained from naive Bayes for each incoming instance **before** the classifier is trained (updated) with the instance. If you have a pre-trained classifier, you can specify that the classifier **not** be updated on incoming instances by unselecting the check box in the configuration dialog for the classifier. If the pre-trained classifier is a **batch** classifier (i.e. it is not capable of incremental training) then you will only be able to test it in an incremental fashion.

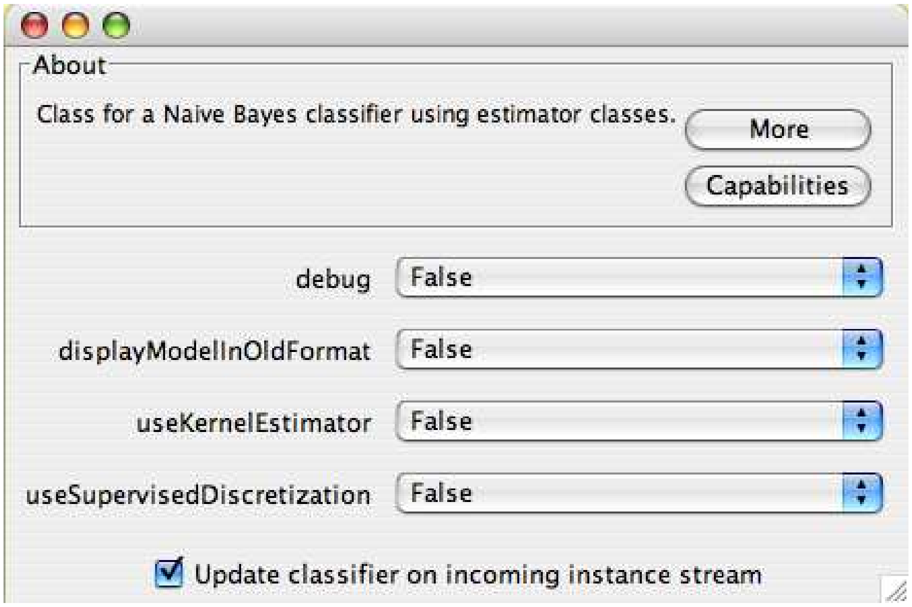

6.5 Plugin Facility

The KnowledgeFlow offers the ability to easily add new components via a plugin mechanism. Plugins are installed in a directory called `.knowledgeFlow/plugins` in the user's home directory. If this directory does not exist you must create it in order to install plugins. Plugins are installed in subdirectories of the `.knowledgeFlow/plugins` directory. More than one plugin component may reside in the same subdirectory. Each subdirectory should contain jar file(s) that contain and support the plugin components. The KnowledgeFlow will dynamically load jar files and add them to the classpath. In order to tell the KnowledgeFlow which classes in the jar files to instantiate as components, a second file called `Beans.props` needs to be created and placed into each plugin subdirectory. This file contains a list of fully qualified class names to be instantiated. Successfully instantiated components will appear in a "Plugins" tab in the KnowledgeFlow user interface.Below is an example plugin directory listing, the listing of the contents of the jar file and the contents of the associated Beans.props file:

```
cygnus:~ mhall$ ls -l $HOME/.knowledgeFlow/plugins/kettle/
total 24
-rw-r--r--   1 mhall  mhall   117 20 Feb 10:56 Beans.props
-rw-r--r--   1 mhall  mhall  8047 20 Feb 14:01 kettleKF.jar

cygnus:~ mhall$ jar tvf /Users/mhall/.knowledgeFlow/plugins/kettle/kettleKF.jar
     0 Wed Feb 20 14:01:34 NZDT 2008 META-INF/
    70 Wed Feb 20 14:01:34 NZDT 2008 META-INF/MANIFEST.MF
     0 Tue Feb 19 14:59:08 NZDT 2008 weka/
     0 Tue Feb 19 14:59:08 NZDT 2008 weka/gui/
     0 Wed Feb 20 13:55:52 NZDT 2008 weka/gui/beans/
     0 Wed Feb 20 13:56:36 NZDT 2008 weka/gui/beans/icons/
  2812 Wed Feb 20 14:01:20 NZDT 2008 weka/gui/beans/icons/KettleInput.gif
  2812 Wed Feb 20 14:01:18 NZDT 2008 weka/gui/beans/icons/KettleInput_animated.gif
  1839 Wed Feb 20 13:59:08 NZDT 2008 weka/gui/beans/KettleInput.class
   174 Tue Feb 19 15:27:24 NZDT 2008 weka/gui/beans/KettleInputBeanInfo.class

cygnus:~ mhall$ more /Users/mhall/.knowledgeFlow/plugins/kettle/Beans.props
# Specifies the tools to go into the Plugins toolbar
weka.gui.beans.KnowledgeFlow.Plugins=weka.gui.beans.KettleInput
```

Chapter 7

ArffViewer

The ArffViewer is a little tool for viewing ARFF files in a tabular format. The advantage of this kind of display over the file representation is, that attribute name, type and data are directly associated in columns and not separated in defintion and data part. But the viewer is not only limited to viewing multiple files at once, but also provides simple editing functionality, like sorting and deleting.

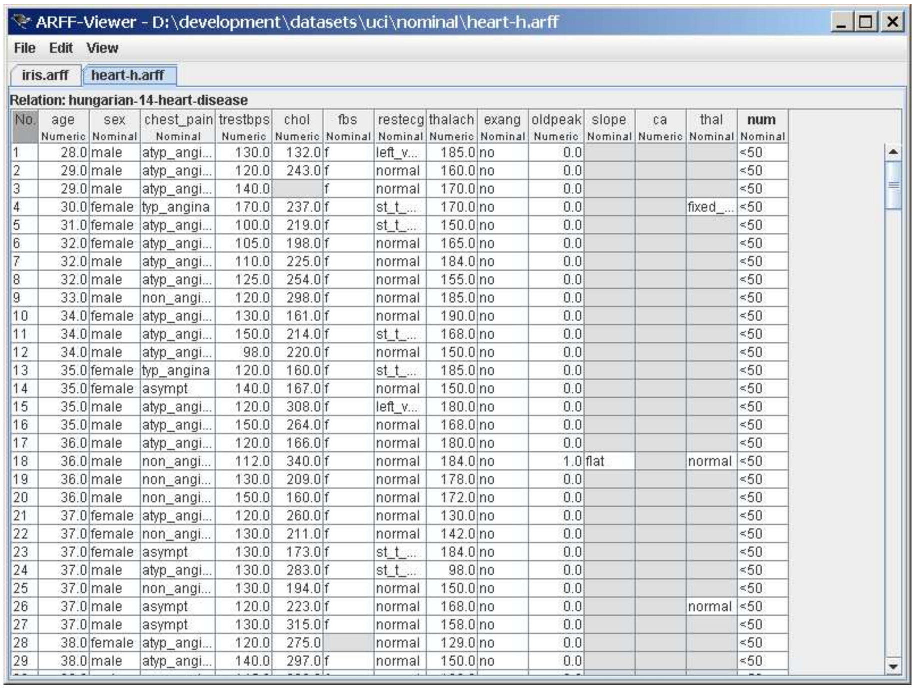

7.1 Menus

The ArffViewer offers most of its functionality either through the main menu or via popups (table header and table cells).

Short description of the available menus:

- **File**

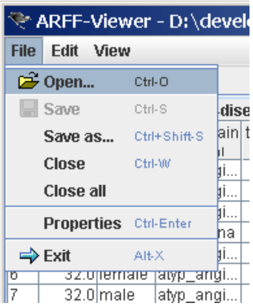

  contains options for opening and closing files, as well as viewing properties about the current file.

- **Edit**

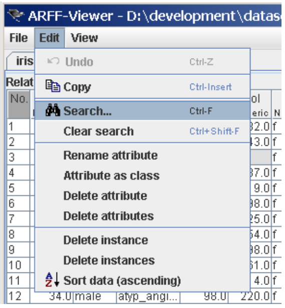

  allows one to delete attributes/instances, rename attributes, choose a new class attribute, search for certain values in the data and of course undo the modifications.

- **View**

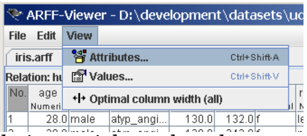

  brings either the chosen attribute into view or displays all the values of an attribute.

After opening a file, by default, the column widths are optimized based on the attribute name and not the content. This is to ensure that overlong cells do not force an enormously wide table, which the user has to reduce with quite some effort.

In the following, screenshots of the table popups:

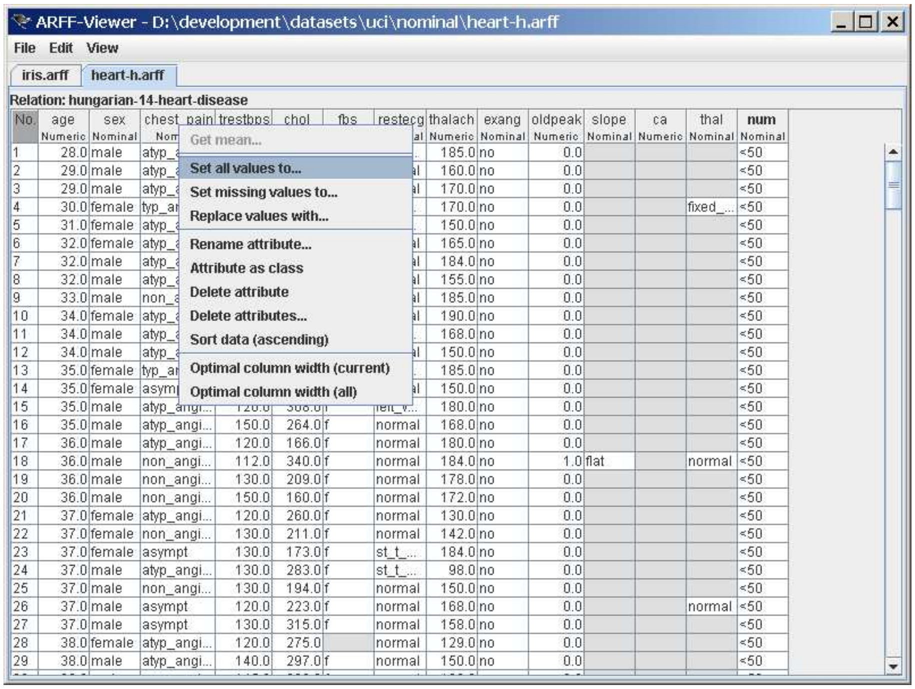

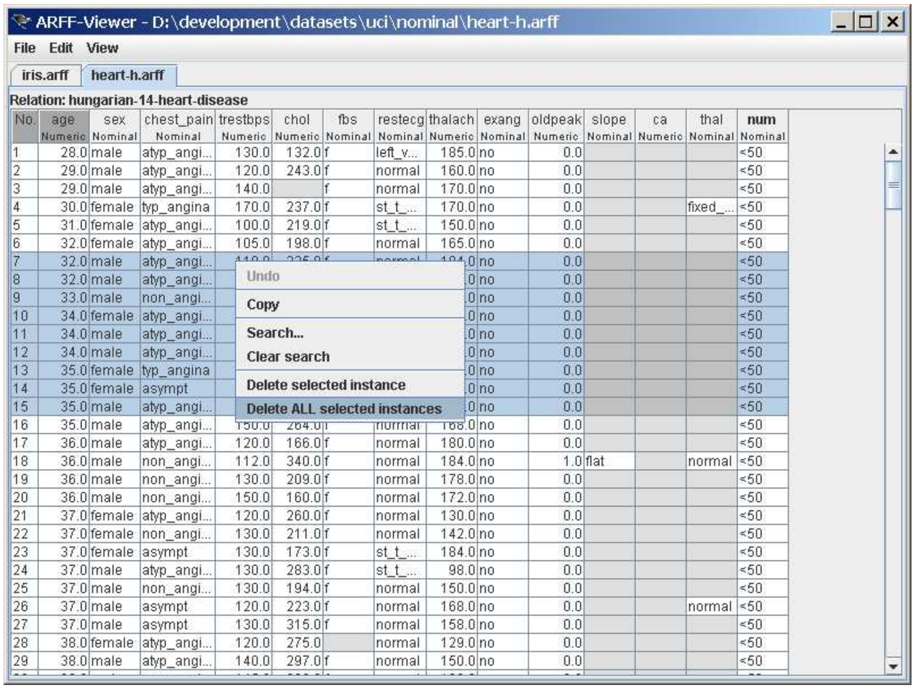

7.2 Editing

Besides the first column, which is the instance index, all cells in the table are editable. Nominal values can be easily modified via dropdown lists, numeric values are edited directly.

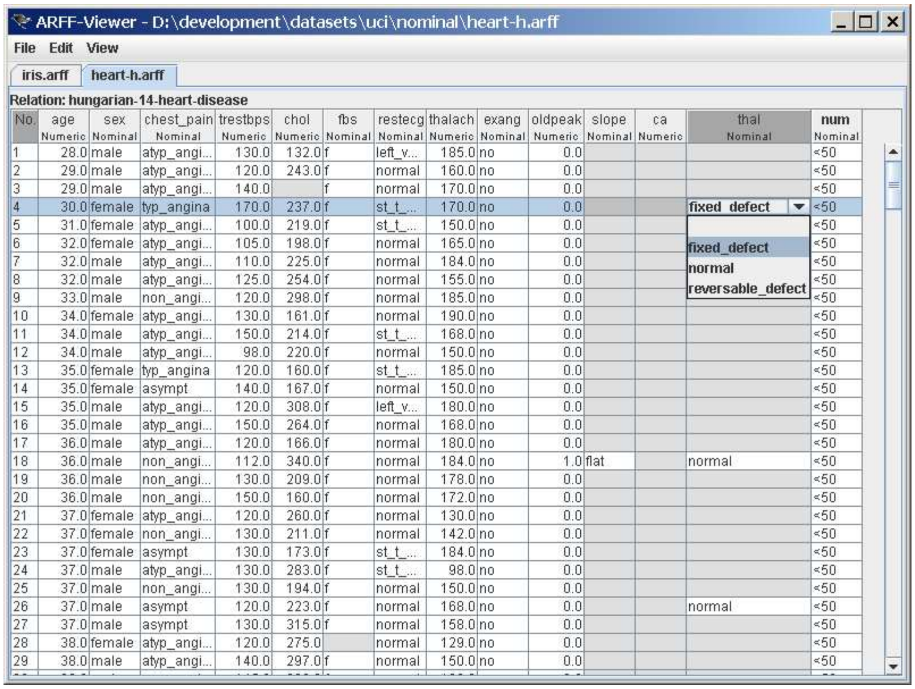

For convenience, it is possible to sort the view based on a column (the underlying data is NOT changed; via Edit/Sort data one can sort the data permanently). This enables one to look for specific values, e.g., missing values. To better distinguish missing values from empty cells, the background of cells with missing values is colored grey.

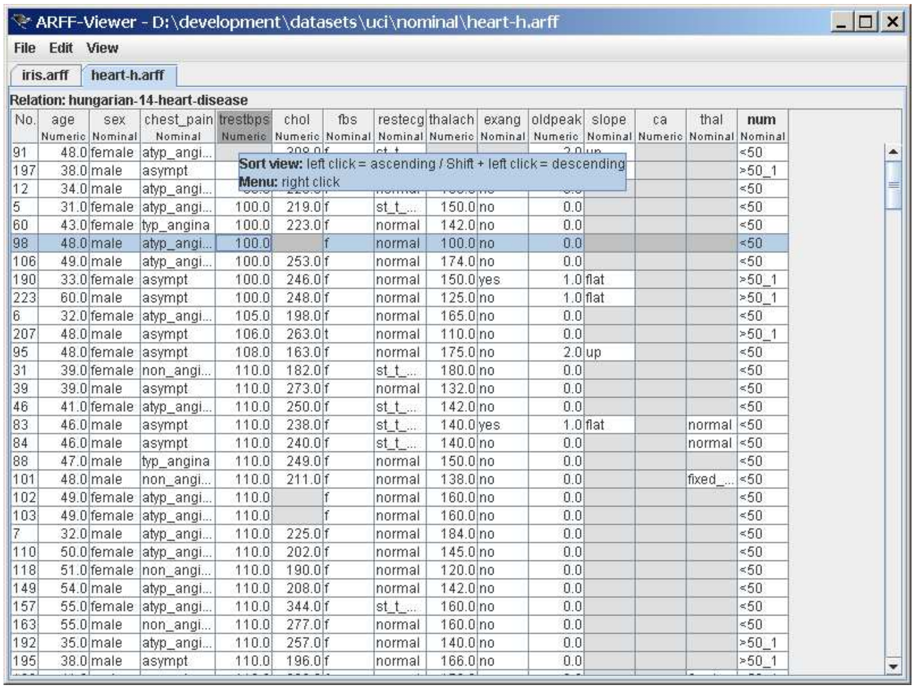

Chapter 8

Bayesian Network Classifiers

8.1 Introduction

Let $U = \{x_1, \ldots, x_n\}$, $n \geq 1$ be a set of variables. A *Bayesian network* B over a set of variables U is a *network structure* B_S, which is a directed acyclic graph (DAG) over U and a set of probability tables $B_P = \{p(u|pa(u))|u \in U\}$ where $pa(u)$ is the set of parents of u in B_S. A Bayesian network represents a probability distributions $P(U) = \prod_{u \in U} p(u|pa(u))$.

Below, a Bayesian network is shown for the variables in the iris data set. Note that the links between the nodes class, petallength and petalwidth do not form a *directed* cycle, so the graph is a proper DAG.

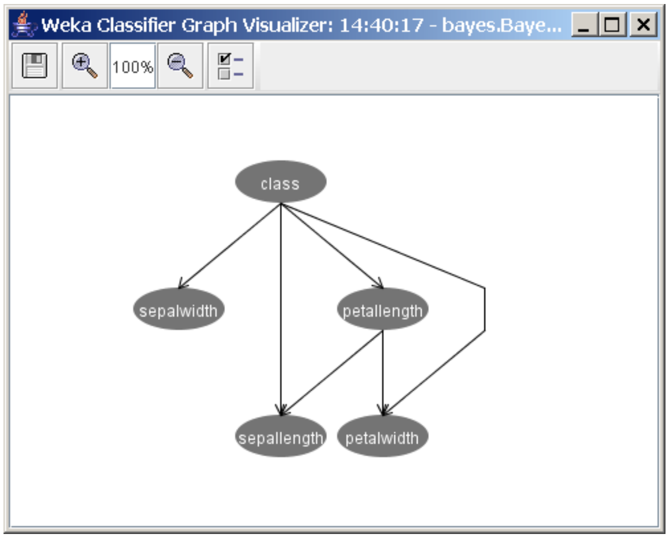

This picture just shows the network structure of the Bayes net, but for each of the nodes a probability distribution for the node given its parents are specified as well. For example, in the Bayes net above there is a conditional distribution

for petallength given the value of class. Since class has no parents, there is an unconditional distribution for sepalwidth.

Basic assumptions

The classification task consist of classifying a variable $y = x_0$ called the *class variable* given a set of variables $\mathbf{x} = x_1 \ldots x_n$, called *attribute variables*. A classifier $h : \mathbf{x} \to y$ is a function that maps an instance of $\mathbf{x}$ to a value of y. The classifier is learned from a dataset D consisting of samples over $(\mathbf{x}, y)$. The learning task consists of finding an appropriate Bayesian network given a data set D over U.

All Bayes network algorithms implemented in Weka assume the following for the data set:

- all variables are discrete finite variables. If you have a data set with continuous variables, you can use the following filter to discretize them: `weka.filters.unsupervised.attribute.Discretize`
- no instances have missing values. If there are missing values in the data set, values are filled in using the following filter: `weka.filters.unsupervised.attribute.ReplaceMissingValues`

The first step performed by `buildClassifier` is checking if the data set fulfills those assumptions. If those assumptions are not met, the data set is automatically filtered and a warning is written to STDERR.[1]

Inference algorithm

To use a Bayesian network as a classifier, one simply calculates $argmax_y P(y|\mathbf{x})$ using the distribution $P(U)$ represented by the Bayesian network. Now note that

$$\begin{aligned} P(y|\mathbf{x}) &= P(U)/P(\mathbf{x}) \\ &\propto P(U) \\ &= \prod_{u \in U} p(u|pa(u)) \end{aligned} \quad (8.1)$$

And since all variables in $\mathbf{x}$ are known, we do not need complicated inference algorithms, but just calculate (8.1) for all class values.

Learning algorithms

The dual nature of a Bayesian network makes learning a Bayesian network as a two stage process a natural division: first learn a network structure, then learn the probability tables.

There are various approaches to structure learning and in Weka, the following areas are distinguished:

[1] If there are missing values in the test data, but not in the training data, the values are filled in in the test data with a ReplaceMissingValues filter based on the training data.

- *local score metrics*: Learning a network structure B_S can be considered an optimization problem where a quality measure of a network structure given the training data $Q(B_S|D)$ needs to be maximized. The quality measure can be based on a Bayesian approach, minimum description length, information and other criteria. Those metrics have the practical property that the score of the whole network can be decomposed as the sum (or product) of the score of the individual nodes. This allows for local scoring and thus local search methods.

- *conditional independence tests*: These methods mainly stem from the goal of uncovering causal structure. The assumption is that there is a network structure that exactly represents the independencies in the distribution that generated the data. Then it follows that if a (conditional) independency can be identified in the data between two variables that there is no arrow between those two variables. Once locations of edges are identified, the direction of the edges is assigned such that conditional independencies in the data are properly represented.

- *global score metrics*: A natural way to measure how well a Bayesian network performs on a given data set is to predict its future performance by estimating expected utilities, such as classification accuracy. Cross-validation provides an out of sample evaluation method to facilitate this by repeatedly splitting the data in training and validation sets. A Bayesian network structure can be evaluated by estimating the network's parameters from the training set and the resulting Bayesian network's performance determined against the validation set. The average performance of the Bayesian network over the validation sets provides a metric for the quality of the network.

  Cross-validation differs from local scoring metrics in that the quality of a network structure often cannot be decomposed in the scores of the individual nodes. So, the whole network needs to be considered in order to determine the score.

- *fixed structure*: Finally, there are a few methods so that a structure can be fixed, for example, by reading it from an XML BIF file[2].

For each of these areas, different search algorithms are implemented in Weka, such as hill climbing, simulated annealing and tabu search.

Once a good network structure is identified, the conditional probability tables for each of the variables can be estimated.

You can select a Bayes net classifier by clicking the classifier 'Choose' button in the Weka explorer, experimenter or knowledge flow and find `BayesNet` under the `weka.classifiers.bayes` package (see below).

---

[2]See `http://www-2.cs.cmu.edu/~fgcozman/Research/InterchangeFormat/` for details on XML BIF.

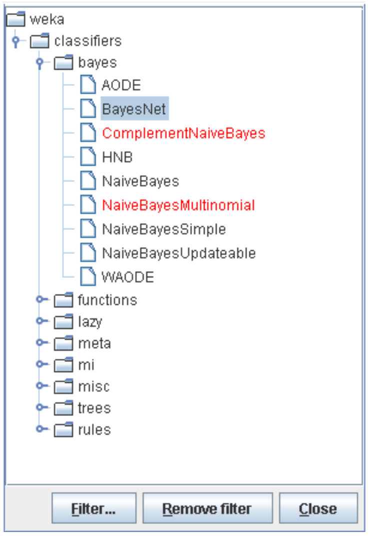

The Bayes net classifier has the following options:

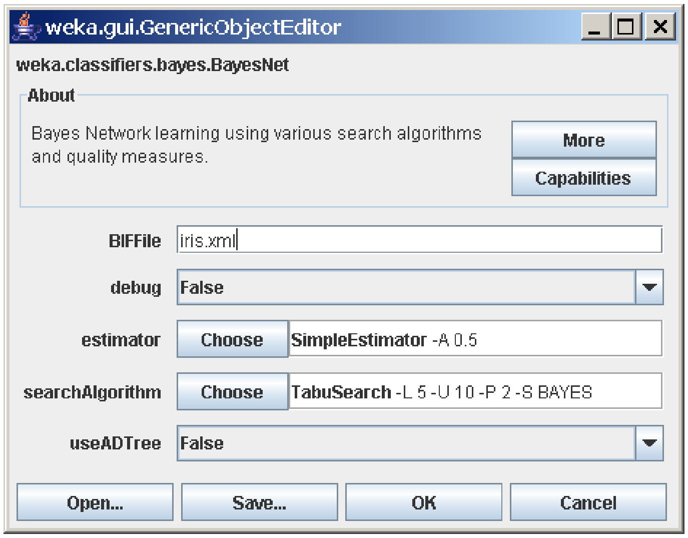

The `BIFFile` option can be used to specify a Bayes network stored in file in BIF format. When the `toString()` method is called after learning the Bayes network, extra statistics (like extra and missing arcs) are printed comparing the network learned with the one on file.

The `searchAlgorithm` option can be used to select a structure learning algorithm and specify its options.

The `estimator` option can be used to select the method for estimating the conditional probability distributions (Section 8.6).

When setting the `useADTree` option to `true`, counts are calculated using the ADTree algorithm of Moore [24]. Since I have not noticed a lot of improvement for small data sets, it is set off by default. Note that this ADTree algorithm is different from the ADTree classifier algorithm from `weka.classifiers.tree.ADTree`.

The `debug` option has no effect.

8.2 Local score based structure learning

Distinguish score metrics (Section 2.1) and search algorithms (Section 2.2). A local score based structure learning can be selected by choosing one in the `weka.classifiers.bayes.net.search.local` package.

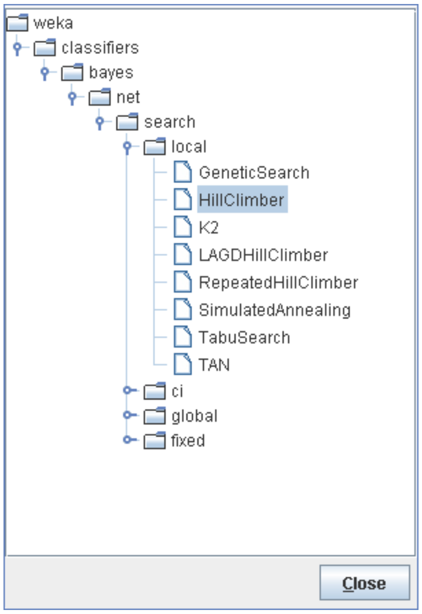

Local score based algorithms have the following options in common:
`initAsNaiveBayes` if set **true** (default), the initial network structure used for starting the traversal of the search space is a naive Bayes network structure. That is, a structure with arrows from the class variable to each of the attribute variables.
If set **false**, an empty network structure will be used (i.e., no arrows at all).
`markovBlanketClassifier` (**false** by default) if set **true**, at the end of the traversal of the search space, a heuristic is used to ensure each of the attributes are in the Markov blanket of the classifier node. If a node is already in the Markov blanket (i.e., is a parent, child of sibling of the classifier node) nothing happens, otherwise an arrow is added.
If set to **false** no such arrows are added.
`scoreType` determines the score metric used (see Section 2.1 for details). Currently, K2, BDe, AIC, Entropy and MDL are implemented.
`maxNrOfParents` is an upper bound on the number of parents of each of the nodes in the network structure learned.

8.2.1 Local score metrics

We use the following conventions to identify counts in the database D and a network structure B_S. Let r_i $(1 \leq i \leq n)$ be the cardinality of x_i. We use q_i to denote the cardinality of the parent set of x_i in B_S, that is, the number of different values to which the parents of x_i can be instantiated. So, q_i can be calculated as the product of cardinalities of nodes in $pa(x_i)$, $q_i = \prod_{x_j \in pa(x_i)} r_j$.

Note $pa(x_i) = \emptyset$ implies $q_i = 1$. We use N_{ij} $(1 \le i \le n,\ 1 \le j \le q_i)$ to denote the number of records in D for which $pa(x_i)$ takes its jth value.We use N_{ijk} $(1 \le i \le n,\ 1 \le j \le q_i,\ 1 \le k \le r_i)$ to denote the number of records in D for which $pa(x_i)$ takes its jth value and for which x_i takes its kth value. So, $N_{ij} = \sum_{k=1}^{r_i} N_{ijk}$. We use N to denote the number of records in D.

Let the *entropy metric* $H(B_S, D)$ of a network structure and database be defined as

$$H(B_S, D) = -N \sum_{i=1}^{n} \sum_{j=1}^{q_i} \sum_{k=1}^{r_i} \frac{N_{ijk}}{N} \log \frac{N_{ijk}}{N_{ij}} \tag{8.2}$$

and the *number of parameters* K as

$$K = \sum_{i=1}^{n} (r_i - 1) \cdot q_i \tag{8.3}$$

AIC metric The AIC metric $Q_{AIC}(B_S, D)$ of a Bayesian network structure B_S for a database D is

$$Q_{AIC}(B_S, D) = H(B_S, D) + K \tag{8.4}$$

A term $P(B_S)$ can be added [15] representing prior information over network structures, but will be ignored for simplicity in the Weka implementation.

MDL metric The minimum description length metric $Q_{MDL}(B_S, D)$ of a Bayesian network structure B_S for a database D is is defined as

$$Q_{MDL}(B_S, D) = H(B_S, D) + \frac{K}{2} \log N \tag{8.5}$$

Bayesian metric The Bayesian metric of a Bayesian network structure B_D for a database D is

$$Q_{Bayes}(B_S, D) = P(B_S) \prod_{i=0}^{n} \prod_{j=1}^{q_i} \frac{\Gamma(N'_{ij})}{\Gamma(N'_{ij} + N_{ij})} \prod_{k=1}^{r_i} \frac{\Gamma(N'_{ijk} + N_{ijk})}{\Gamma(N'_{ijk})}$$

where $P(B_S)$ is the prior on the network structure (taken to be constant hence ignored in the Weka implementation) and $\Gamma(.)$ the gamma-function. N'_{ij} and N'_{ijk} represent choices of priors on counts restricted by $N'_{ij} = \sum_{k=1}^{r_i} N'_{ijk}$. With $N'_{ijk} = 1$ (and thus $N'_{ij} = r_i$), we obtain the K2 metric [19]

$$Q_{K2}(B_S, D) = P(B_S) \prod_{i=0}^{n} \prod_{j=1}^{q_i} \frac{(r_i - 1)!}{(r_i - 1 + N_{ij})!} \prod_{k=1}^{r_i} N_{ijk}!$$

With $N'_{ijk} = 1/r_i \cdot q_i$ (and thus $N'_{ij} = 1/q_i$), we obtain the **BDe metric** [22].

8.2.2 Search algorithms

The following search algorithms are implemented for local score metrics;

- *K2* [19]: hill climbing add arcs with a fixed ordering of variables.
  Specific option: `randomOrder` if **true** a random ordering of the nodes is made at the beginning of the search. If **false** (default) the ordering in the data set is used. The only exception in both cases is that in case the initial network is a naive Bayes network (`initAsNaiveBayes` set **true**) the class variable is made first in the ordering.

- *Hill Climbing* [16]: hill climbing adding and deleting arcs with no fixed ordering of variables.
  `useArcReversal` if **true**, also arc reversals are consider when determining the next step to make.

- *Repeated Hill Climber* starts with a randomly generated network and then applies hill climber to reach a local optimum. The best network found is returned.
  `useArcReversal` option as for Hill Climber.

- *LAGD Hill Climbing* does hill climbing with look ahead on a limited set of best scoring steps, implemented by Manuel Neubach. The number of look ahead steps and number of steps considered for look ahead are configurable.

- *TAN* [17, 21]: *T*ree *A*ugmented *N*aive Bayes where the tree is formed by calculating the maximum weight spanning tree using Chow and Liu algorithm [18].
  No specific options.

- *Simulated annealing* [15]: using adding and deleting arrows.
  The algorithm randomly generates a candidate network B'_S close to the current network B_S. It accepts the network if it is better than the current, i.e., $Q(B'_S, D) > Q(B_S, D)$. Otherwise, it accepts the candidate with probability
  $$e^{t_i \cdot (Q(B'_S,D) - Q(B_S,D))}$$
  where t_i is the temperature at iteration i. The temperature starts at t_0 and is slowly decreases with each iteration.

  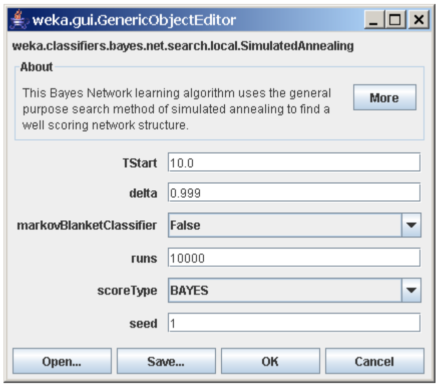

  Specific options:
  `TStart` start temperature t_0.
  `delta` is the factor δ used to update the temperature, so $t_{i+1} = t_i \cdot \delta$.
  `runs` number of iterations used to traverse the search space.
  `seed` is the initialization value for the random number generator.

- *Tabu search* [15]: using adding and deleting arrows.
  Tabu search performs hill climbing until it hits a local optimum. Then it

steps to the least worse candidate in the neighborhood. However, it does not consider points in the neighborhood it just visited in the last tl steps. These steps are stored in a so called tabu-list.

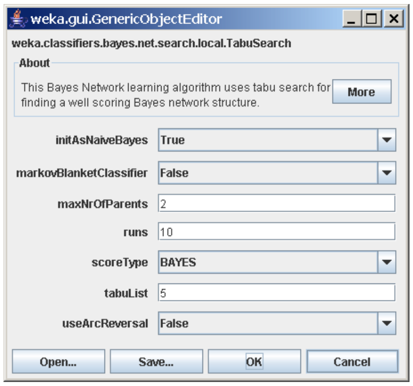

Specific options:
`runs` is the number of iterations used to traverse the search space.
`tabuList` is the length tl of the tabu list.

- *Genetic search*: applies a simple implementation of a genetic search algorithm to network structure learning. A Bayes net structure is represented by a array of $n \cdot n$ (n = number of nodes) bits where bit $i \cdot n + j$ represents whether there is an arrow from node $j \rightarrow i$.

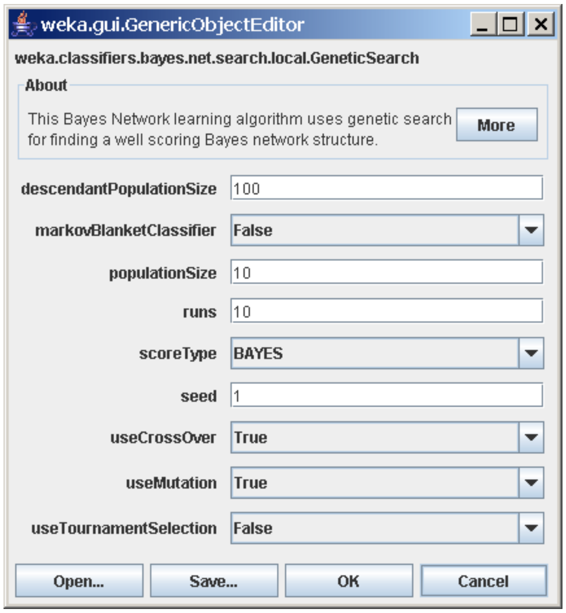

Specific options:
`populationSize` is the size of the population selected in each generation.
`descendantPopulationSize` is the number of offspring generated in each

generation.
`runs` is the number of generation to generate.
`seed` is the initialization value for the random number generator.
`useMutation` flag to indicate whether mutation should be used. Mutation is applied by randomly adding or deleting a single arc.
`useCrossOver` flag to indicate whether cross-over should be used. Cross-over is applied by randomly picking an index k in the bit representation and selecting the first k bits from one and the remainder from another network structure in the population. At least one of `useMutation` and `useCrossOver` should be set to **true**.
`useTournamentSelection` when **false**, the best performing networks are selected from the descendant population to form the population of the next generation. When **true**, tournament selection is used. Tournament selection randomly chooses two individuals from the descendant population and selects the one that performs best.

8.3 Conditional independence test based structure learning

Conditional independence tests in Weka are slightly different from the standard tests described in the literature. To test whether variables x and y are conditionally independent given a set of variables Z, a network structure with arrows $\forall_{z \in Z} z \rightarrow y$ is compared with one with arrows $\{x \rightarrow y\} \cup \forall_{z \in Z} z \rightarrow y$. A test is performed by using any of the score metrics described in Section 2.1.

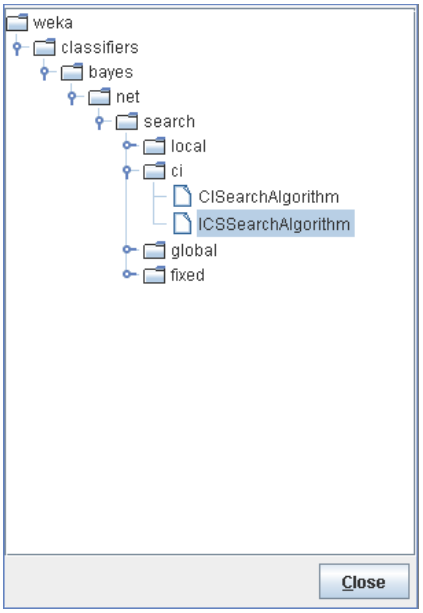

At the moment, only the ICS [25]and CI algorithm are implemented.

The ICS algorithm makes two steps, first find a skeleton (the undirected graph with edges iff there is an arrow in network structure) and second direct

all the edges in the skeleton to get a DAG.

Starting with a complete undirected graph, we try to find conditional independencies $\langle x, y|Z\rangle$ in the data. For each pair of nodes x, y, we consider sets Z starting with cardinality 0, then 1 up to a user defined maximum. Furthermore, the set Z is a subset of nodes that are neighbors of both x and y. If an independency is identified, the edge between x and y is removed from the skeleton.

The first step in directing arrows is to check for every configuration $x--z--y$ where x and y not connected in the skeleton whether z is in the set Z of variables that justified removing the link between x and y (cached in the first step). If z is not in Z, we can assign direction $x \rightarrow z \leftarrow y$.

Finally, a set of graphical rules is applied [25] to direct the remaining arrows.

```
Rule 1: i->j--k & i-/-k => j->k
Rule 2: i->j->k & i--k => i->k
Rule 3  m
            /|\
           i | k  => m->j
     i->j<-k  \|/
             j

Rule 4  m
            / \
           i---k  => i->m & k->m
       i->j   \ /
             j
Rule 5: if no edges are directed then take a random one (first we can find)
```

The ICS algorithm comes with the following options.

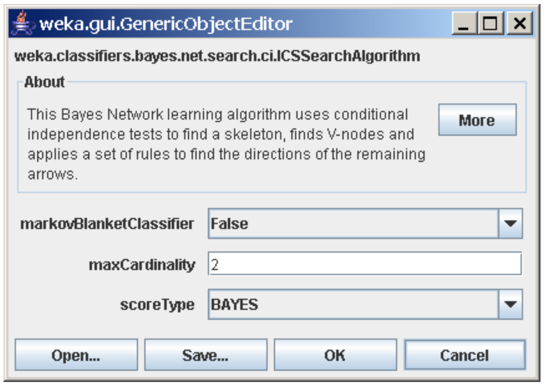

Since the ICS algorithm is focused on recovering causal structure, instead of finding the optimal classifier, the Markov blanket correction can be made afterwards.

Specific options:
The `maxCardinality` option determines the largest subset of Z to be considered in conditional independence tests $\langle x, y|Z\rangle$.
The `scoreType` option is used to select the scoring metric.

8.4 Global score metric based structure learning

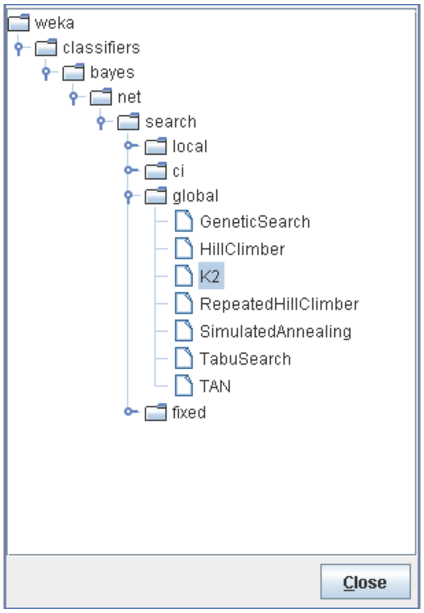

Common options for cross-validation based algorithms are: `initAsNaiveBayes`, `markovBlanketClassifier` and `maxNrOfParents` (see Section 8.2 for description).

Further, for each of the cross-validation based algorithms the `CVType` can be chosen out of the following:

- *Leave one out cross-validation (loo-cv)* selects $m = N$ training sets simply by taking the data set D and removing the ith record for training set D_i^t. The validation set consist of just the ith single record. Loo-cv does not always produce accurate performance estimates.

- *K-fold cross-validation (k-fold cv)* splits the data D in m approximately equal parts $D_1, \ldots, D_m$. Training set D_i^t is obtained by removing part D_i from D. Typical values for m are 5, 10 and 20. With $m = N$, k-fold cross-validation becomes loo-cv.

- *Cumulative cross-validation (cumulative cv)* starts with an empty data set and adds instances item by item from D. After each time an item is added the next item to be added is classified using the then current state of the Bayes network.

Finally, the `useProb` flag indicates whether the accuracy of the classifier should be estimated using the zero-one loss (if set to **false**) or using the estimated probability of the class.

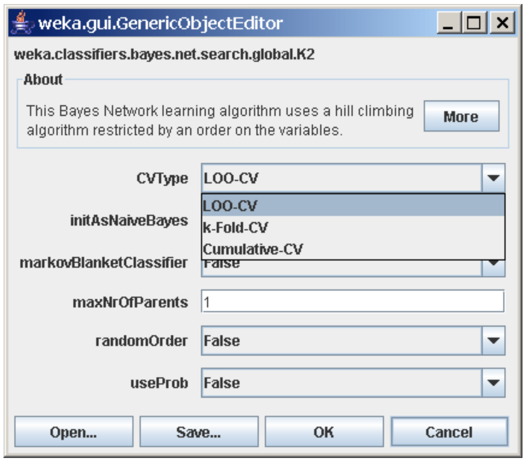

The following search algorithms are implemented: K2, HillClimbing, RepeatedHillClimber, TAN, Tabu Search, Simulated Annealing and Genetic Search. See Section 8.2 for a description of the specific options for those algorithms.

8.5 Fixed structure 'learning'

The structure learning step can be skipped by selecting a fixed network structure. There are two methods of getting a fixed structure: just make it a naive Bayes network, or reading it from a file in XML BIF format.

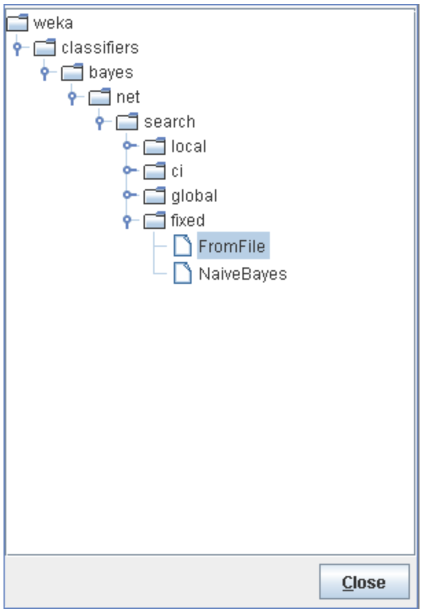

8.6 Distribution learning

Once the network structure is learned, you can choose how to learn the probability tables selecting a class in the `weka.classifiers.bayes.net.estimate`

package.

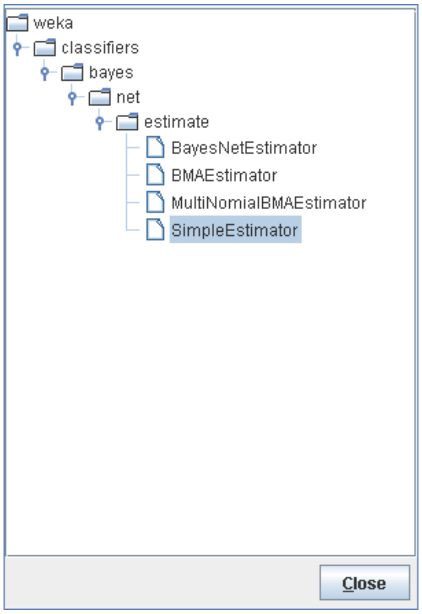

The `SimpleEstimator` class produces direct estimates of the conditional probabilities, that is,

$$P(x_i = k|pa(x_i) = j) = \frac{N_{ijk} + N'_{ijk}}{N_{ij} + N'_{ij}}$$

where N'_{ijk} is the alpha parameter that can be set and is 0.5 by default. With $alpha = 0$, we get maximum likelihood estimates.

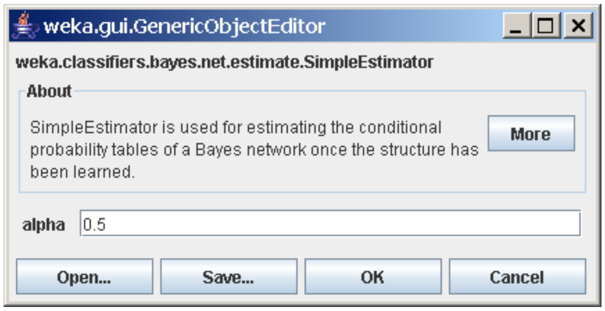

With the `BMAEstimator`, we get estimates for the conditional probability tables based on Bayes model averaging of all network structures that are substructures of the network structure learned [15]. This is achieved by estimating the conditional probability table of a node x_i given its parents $pa(x_i)$ as a weighted average of all conditional probability tables of x_i given subsets of $pa(x_i)$. The weight of a distribution $P(x_i|S)$ with $S \subseteq pa(x_i)$ used is proportional to the contribution of network structure $\forall_{y \in S} y \to x_i$ to either the BDe metric or K2 metric depending on the setting of the `useK2Prior` option (**false** and **true** respectively).

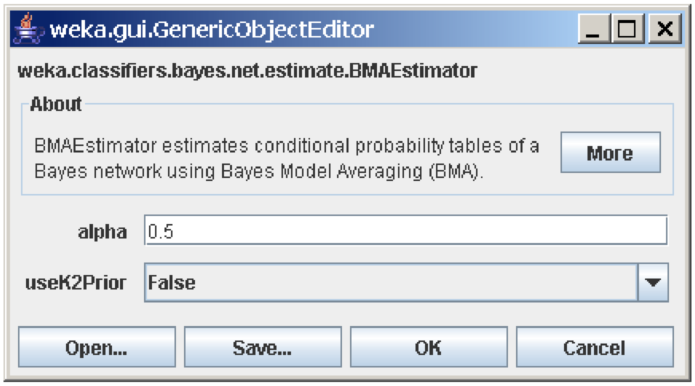

8.7 Running from the command line

These are the command line options of `BayesNet`.

```
General options:

-t <name of training file>
        Sets training file.
-T <name of test file>
        Sets test file. If missing, a cross-validation will be performed on the
        training data.
-c <class index>
        Sets index of class attribute (default: last).
-x <number of folds>
        Sets number of folds for cross-validation (default: 10).
-no-cv
        Do not perform any cross validation.
-split-percentage <percentage>
        Sets the percentage for the train/test set split, e.g., 66.
-preserve-order
        Preserves the order in the percentage split.
-s <random number seed>
        Sets random number seed for cross-validation or percentage split
        (default: 1).
-m <name of file with cost matrix>
        Sets file with cost matrix.
-l <name of input file>
        Sets model input file. In case the filename ends with '.xml',
        the options are loaded from the XML file.
-d <name of output file>
        Sets model output file. In case the filename ends with '.xml',
        only the options are saved to the XML file, not the model.
-v
        Outputs no statistics for training data.
-o
        Outputs statistics only, not the classifier.
-i
        Outputs detailed information-retrieval statistics for each class.
-k
```

```
        Outputs information-theoretic statistics.
-p <attribute range>
        Only outputs predictions for test instances (or the train
        instances if no test instances provided), along with attributes
        (0 for none).
-distribution
        Outputs the distribution instead of only the prediction
        in conjunction with the '-p' option (only nominal classes).
-r
        Only outputs cumulative margin distribution.
-g
        Only outputs the graph representation of the classifier.
-xml filename | xml-string
        Retrieves the options from the XML-data instead of the command line.

Options specific to weka.classifiers.bayes.BayesNet:

-D
        Do not use ADTree data structure

-B <BIF file>
        BIF file to compare with

-Q weka.classifiers.bayes.net.search.SearchAlgorithm
        Search algorithm

-E weka.classifiers.bayes.net.estimate.SimpleEstimator
        Estimator algorithm
```

The search algorithm option -Q and estimator option -E options are mandatory.

Note that it is important that the -E options should be used after the -Q option. Extra options can be passed to the search algorithm and the estimator after the class name specified following '--'.
For example:

```
java weka.classifiers.bayes.BayesNet -t iris.arff -D \
  -Q weka.classifiers.bayes.net.search.local.K2 -- -P 2 -S ENTROPY \
  -E weka.classifiers.bayes.net.estimate.SimpleEstimator -- -A 1.0
```

Overview of options for search algorithms

- `weka.classifiers.bayes.net.search.local.GeneticSearch`

```
-L <integer>
        Population size
-A <integer>
        Descendant population size
-U <integer>
        Number of runs
-M
        Use mutation.
```

```
        (default true)
-C
        Use cross-over.
        (default true)
-O
        Use tournament selection (true) or maximum subpopulatin (false).
        (default false)
-R <seed>
        Random number seed
-mbc
        Applies a Markov Blanket correction to the network structure,
        after a network structure is learned. This ensures that all
        nodes in the network are part of the Markov blanket of the
        classifier node.
-S [BAYES|MDL|ENTROPY|AIC|CROSS_CLASSIC|CROSS_BAYES]
        Score type (BAYES, BDeu, MDL, ENTROPY and AIC)
```

- weka.classifiers.bayes.net.search.local.HillClimber

```
-P <nr of parents>
        Maximum number of parents
-R
        Use arc reversal operation.
        (default false)
-N
        Initial structure is empty (instead of Naive Bayes)
-mbc
        Applies a Markov Blanket correction to the network structure,
        after a network structure is learned. This ensures that all
        nodes in the network are part of the Markov blanket of the
        classifier node.
-S [BAYES|MDL|ENTROPY|AIC|CROSS_CLASSIC|CROSS_BAYES]
        Score type (BAYES, BDeu, MDL, ENTROPY and AIC)
```

- weka.classifiers.bayes.net.search.local.K2

```
-N
        Initial structure is empty (instead of Naive Bayes)
-P <nr of parents>
        Maximum number of parents
-R
        Random order.
        (default false)
-mbc
        Applies a Markov Blanket correction to the network structure,
        after a network structure is learned. This ensures that all
        nodes in the network are part of the Markov blanket of the
        classifier node.
```

```
-S [BAYES|MDL|ENTROPY|AIC|CROSS_CLASSIC|CROSS_BAYES]
        Score type (BAYES, BDeu, MDL, ENTROPY and AIC)
```

- weka.classifiers.bayes.net.search.local.LAGDHillClimber

```
-L <nr of look ahead steps>
        Look Ahead Depth
-G <nr of good operations>
        Nr of Good Operations
-P <nr of parents>
        Maximum number of parents
-R
        Use arc reversal operation.
        (default false)
-N
        Initial structure is empty (instead of Naive Bayes)
-mbc
        Applies a Markov Blanket correction to the network structure,
        after a network structure is learned. This ensures that all
        nodes in the network are part of the Markov blanket of the
        classifier node.
-S [BAYES|MDL|ENTROPY|AIC|CROSS_CLASSIC|CROSS_BAYES]
        Score type (BAYES, BDeu, MDL, ENTROPY and AIC)
```

- weka.classifiers.bayes.net.search.local.RepeatedHillClimber

```
-U <integer>
        Number of runs
-A <seed>
        Random number seed
-P <nr of parents>
        Maximum number of parents
-R
        Use arc reversal operation.
        (default false)
-N
        Initial structure is empty (instead of Naive Bayes)
-mbc
        Applies a Markov Blanket correction to the network structure,
        after a network structure is learned. This ensures that all
        nodes in the network are part of the Markov blanket of the
        classifier node.
-S [BAYES|MDL|ENTROPY|AIC|CROSS_CLASSIC|CROSS_BAYES]
        Score type (BAYES, BDeu, MDL, ENTROPY and AIC)
```

- weka.classifiers.bayes.net.search.local.SimulatedAnnealing

```
-A <float>
        Start temperature
-U <integer>
        Number of runs
-D <float>
        Delta temperature
-R <seed>
        Random number seed
-mbc
        Applies a Markov Blanket correction to the network structure,
        after a network structure is learned. This ensures that all
        nodes in the network are part of the Markov blanket of the
        classifier node.
-S [BAYES|MDL|ENTROPY|AIC|CROSS_CLASSIC|CROSS_BAYES]
        Score type (BAYES, BDeu, MDL, ENTROPY and AIC)
```

- weka.classifiers.bayes.net.search.local.TabuSearch

```
-L <integer>
        Tabu list length
-U <integer>
        Number of runs
-P <nr of parents>
        Maximum number of parents
-R
        Use arc reversal operation.
        (default false)
-P <nr of parents>
        Maximum number of parents
-R
        Use arc reversal operation.
        (default false)
-N
        Initial structure is empty (instead of Naive Bayes)
-mbc
        Applies a Markov Blanket correction to the network structure,
        after a network structure is learned. This ensures that all
        nodes in the network are part of the Markov blanket of the
        classifier node.
-S [BAYES|MDL|ENTROPY|AIC|CROSS_CLASSIC|CROSS_BAYES]
        Score type (BAYES, BDeu, MDL, ENTROPY and AIC)
```

- weka.classifiers.bayes.net.search.local.TAN

```
-mbc
        Applies a Markov Blanket correction to the network structure,
        after a network structure is learned. This ensures that all
        nodes in the network are part of the Markov blanket of the
```

```
        classifier node.
-S [BAYES|MDL|ENTROPY|AIC|CROSS_CLASSIC|CROSS_BAYES]
        Score type (BAYES, BDeu, MDL, ENTROPY and AIC)
```

- weka.classifiers.bayes.net.search.ci.CISearchAlgorithm

```
-mbc
        Applies a Markov Blanket correction to the network structure,
        after a network structure is learned. This ensures that all
        nodes in the network are part of the Markov blanket of the
        classifier node.
-S [BAYES|MDL|ENTROPY|AIC|CROSS_CLASSIC|CROSS_BAYES]
        Score type (BAYES, BDeu, MDL, ENTROPY and AIC)
```

- weka.classifiers.bayes.net.search.ci.ICSSearchAlgorithm

```
-cardinality <num>
        When determining whether an edge exists a search is performed
        for a set Z that separates the nodes. MaxCardinality determines
        the maximum size of the set Z. This greatly influences the
        length of the search. (default 2)
-mbc
        Applies a Markov Blanket correction to the network structure,
        after a network structure is learned. This ensures that all
        nodes in the network are part of the Markov blanket of the
        classifier node.
-S [BAYES|MDL|ENTROPY|AIC|CROSS_CLASSIC|CROSS_BAYES]
        Score type (BAYES, BDeu, MDL, ENTROPY and AIC)
```

- weka.classifiers.bayes.net.search.global.GeneticSearch

```
-L <integer>
        Population size
-A <integer>
        Descendant population size
-U <integer>
        Number of runs
-M
        Use mutation.
        (default true)
-C
        Use cross-over.
        (default true)
-O
        Use tournament selection (true) or maximum subpopulatin (false).
        (default false)
-R <seed>
```

```
        Random number seed
-mbc
        Applies a Markov Blanket correction to the network structure,
        after a network structure is learned. This ensures that all
        nodes in the network are part of the Markov blanket of the
        classifier node.
-S [LOO-CV|k-Fold-CV|Cumulative-CV]
        Score type (LOO-CV,k-Fold-CV,Cumulative-CV)
-Q
        Use probabilistic or 0/1 scoring.
        (default probabilistic scoring)
```

- weka.classifiers.bayes.net.search.global.HillClimber

```
-P <nr of parents>
        Maximum number of parents
-R
        Use arc reversal operation.
        (default false)
-N
        Initial structure is empty (instead of Naive Bayes)
-mbc
        Applies a Markov Blanket correction to the network structure,
        after a network structure is learned. This ensures that all
        nodes in the network are part of the Markov blanket of the
        classifier node.
-S [LOO-CV|k-Fold-CV|Cumulative-CV]
        Score type (LOO-CV,k-Fold-CV,Cumulative-CV)
-Q
        Use probabilistic or 0/1 scoring.
        (default probabilistic scoring)
```

- weka.classifiers.bayes.net.search.global.K2

```
-N
        Initial structure is empty (instead of Naive Bayes)
-P <nr of parents>
        Maximum number of parents
-R
        Random order.
        (default false)
-mbc
        Applies a Markov Blanket correction to the network structure,
        after a network structure is learned. This ensures that all
        nodes in the network are part of the Markov blanket of the
        classifier node.
-S [LOO-CV|k-Fold-CV|Cumulative-CV]
        Score type (LOO-CV,k-Fold-CV,Cumulative-CV)
```

```
-Q
        Use probabilistic or 0/1 scoring.
        (default probabilistic scoring)
```

- weka.classifiers.bayes.net.search.global.RepeatedHillClimber

```
-U <integer>
        Number of runs
-A <seed>
        Random number seed
-P <nr of parents>
        Maximum number of parents
-R
        Use arc reversal operation.
        (default false)
-N
        Initial structure is empty (instead of Naive Bayes)
-mbc
        Applies a Markov Blanket correction to the network structure,
        after a network structure is learned. This ensures that all
        nodes in the network are part of the Markov blanket of the
        classifier node.
-S [LOO-CV|k-Fold-CV|Cumulative-CV]
        Score type (LOO-CV,k-Fold-CV,Cumulative-CV)
-Q
        Use probabilistic or 0/1 scoring.
        (default probabilistic scoring)
```

- weka.classifiers.bayes.net.search.global.SimulatedAnnealing

```
-A <float>
        Start temperature
-U <integer>
        Number of runs
-D <float>
        Delta temperature
-R <seed>
        Random number seed
-mbc
        Applies a Markov Blanket correction to the network structure,
        after a network structure is learned. This ensures that all
        nodes in the network are part of the Markov blanket of the
        classifier node.
-S [LOO-CV|k-Fold-CV|Cumulative-CV]
        Score type (LOO-CV,k-Fold-CV,Cumulative-CV)
-Q
        Use probabilistic or 0/1 scoring.
        (default probabilistic scoring)
```

- weka.classifiers.bayes.net.search.global.TabuSearch

```
-L <integer>
        Tabu list length
-U <integer>
        Number of runs
-P <nr of parents>
        Maximum number of parents
-R
        Use arc reversal operation.
        (default false)
-P <nr of parents>
        Maximum number of parents
-R
        Use arc reversal operation.
        (default false)
-N
        Initial structure is empty (instead of Naive Bayes)
-mbc
        Applies a Markov Blanket correction to the network structure,
        after a network structure is learned. This ensures that all
        nodes in the network are part of the Markov blanket of the
        classifier node.
-S [LOO-CV|k-Fold-CV|Cumulative-CV]
        Score type (LOO-CV,k-Fold-CV,Cumulative-CV)
-Q
        Use probabilistic or 0/1 scoring.
        (default probabilistic scoring)
```

- weka.classifiers.bayes.net.search.global.TAN

```
-mbc
        Applies a Markov Blanket correction to the network structure,
        after a network structure is learned. This ensures that all
        nodes in the network are part of the Markov blanket of the
        classifier node.
-S [LOO-CV|k-Fold-CV|Cumulative-CV]
        Score type (LOO-CV,k-Fold-CV,Cumulative-CV)
-Q
        Use probabilistic or 0/1 scoring.
        (default probabilistic scoring)
```

- weka.classifiers.bayes.net.search.fixed.FromFile

```
-B <BIF File>
        Name of file containing network structure in BIF format
```

- weka.classifiers.bayes.net.search.fixed.NaiveBayes

```
No options.
```

Overview of options for estimators

- `weka.classifiers.bayes.net.estimate.BayesNetEstimator`

```
-A <alpha>
        Initial count (alpha)
```

- `weka.classifiers.bayes.net.estimate.BMAEstimator`

```
-k2
        Whether to use K2 prior.
-A <alpha>
        Initial count (alpha)
```

- `weka.classifiers.bayes.net.estimate.MultiNomialBMAEstimator`

```
-k2
        Whether to use K2 prior.
-A <alpha>
        Initial count (alpha)
```

- `weka.classifiers.bayes.net.estimate.SimpleEstimator`

```
-A <alpha>
        Initial count (alpha)
```

Generating random networks and artificial data sets

You can generate random Bayes nets and data sets using
`weka.classifiers.bayes.net.BayesNetGenerator`
The options are:

```
-B
        Generate network (instead of instances)
-N <integer>
        Nr of nodes
-A <integer>
        Nr of arcs
-M <integer>
        Nr of instances
-C <integer>
        Cardinality of the variables
-S <integer>
        Seed for random number generator
-F <file>
        The BIF file to obtain the structure from.
```

The network structure is generated by first generating a tree so that we can ensure that we have a connected graph. If any more arrows are specified they are randomly added.

8.8 Inspecting Bayesian networks

You can inspect some of the properties of Bayesian networks that you learned in the Explorer in text format and also in graphical format.

Bayesian networks in text

Below, you find output typical for a 10 fold cross-validation run in the Weka Explorer with comments where the output is specific for Bayesian nets.

```
=== Run information ===

Scheme:       weka.classifiers.bayes.BayesNet -D -B iris.xml -Q weka.classifiers.bayes.n
```

Options for `BayesNet` include the class names for the structure learner and for the distribution estimator.

```
Relation:     iris-weka.filters.unsupervised.attribute.Discretize-B2-M-1.0-Rfirst-last
Instances:    150
Attributes:   5
              sepallength
              sepalwidth
              petallength
              petalwidth
              class
Test mode:    10-fold cross-validation

=== Classifier model (full training set) ===

Bayes Network Classifier
not using ADTree
```

Indication whether the ADTree algorithm [24] for calculating counts in the data set was used.

```
#attributes=5 #classindex=4
```

This line lists the number of attribute and the number of the class variable for which the classifier was trained.

```
Network structure (nodes followed by parents)
sepallength(2): class
sepalwidth(2): class
petallength(2): class sepallength
petalwidth(2): class petallength
class(3):
```

This list specifies the network structure. Each of the variables is followed by a list of parents, so the *petallength* variable has parents *sepallength* and *class*, while *class* has no parents. The number in braces is the cardinality of the variable. It shows that in the iris dataset there are three class variables. All other variables are made binary by running it through a discretization filter.

```
LogScore Bayes: -374.9942769685747
LogScore BDeu: -351.85811477631626
LogScore MDL: -416.86897021246466
LogScore ENTROPY: -366.76261727150217
LogScore AIC: -386.76261727150217
```

These lines list the logarithmic score of the network structure for various methods of scoring.

If a BIF file was specified, the following two lines will be produced (if no such file was specified, no information is printed).

```
Missing: 0 Extra: 2 Reversed: 0
Divergence: -0.0719759699700729
```

In this case the network that was learned was compared with a file `iris.xml` which contained the naive Bayes network structure. The number after "Missing" is the number of arcs that was in the network in file that is not recovered by the structure learner. Note that a reversed arc is not counted as missing. The number after "Extra" is the number of arcs in the learned network that are not in the network on file. The number of reversed arcs is listed as well.

Finally, the divergence between the network distribution on file and the one learned is reported. This number is calculated by enumerating all possible instantiations of all variables, so it may take some time to calculate the divergence for large networks.

The remainder of the output is standard output for all classifiers.

```
Time taken to build model: 0.01 seconds

=== Stratified cross-validation ===
=== Summary ===

Correctly Classified Instances         116               77.3333 %
Incorrectly Classified Instances        34               22.6667 %
```

etc...

Bayesian networks in GUI

To show the graphical structure, right click the appropriate `BayesNet` in result list of the Explorer. A menu pops up, in which you select "Visualize graph".

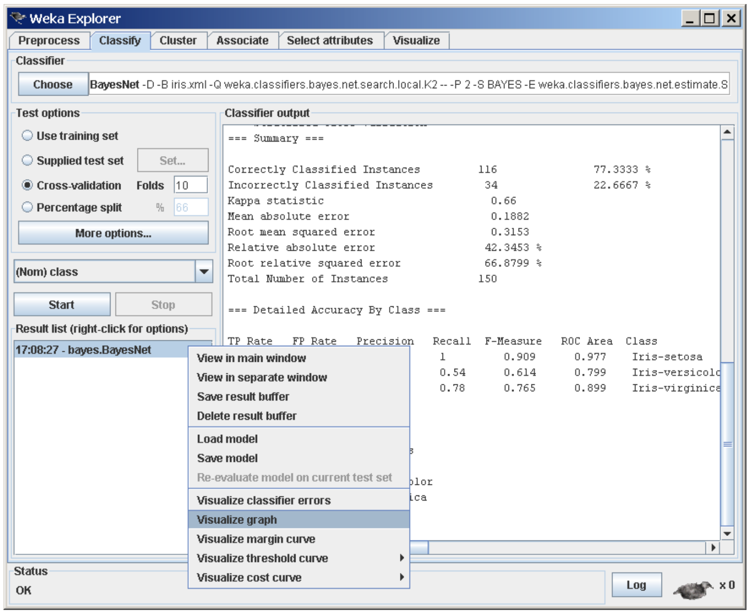

The Bayes network is automatically layed out and drawn thanks to a graph drawing algorithm implemented by Ashraf Kibriya.

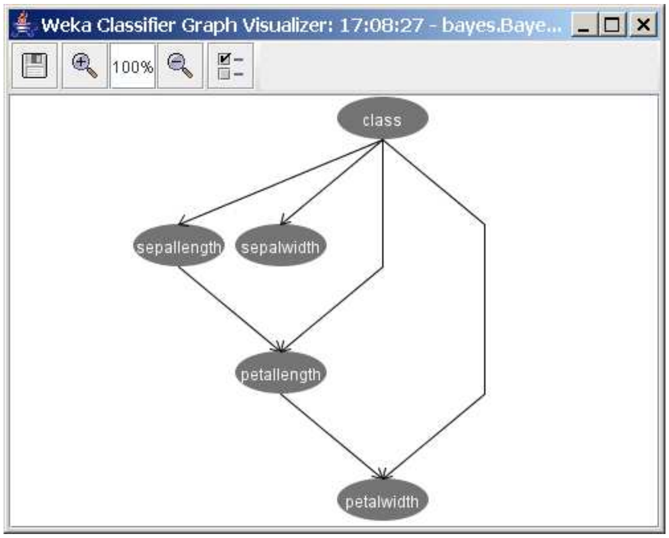

When you hover the mouse over a node, the node lights up and all its children are highlighted as well, so that it is easy to identify the relation between nodes in crowded graphs.

Saving Bayes nets You can save the Bayes network to file in the graph visualizer. You have the choice to save as XML BIF format or as dot format. Select the floppy button and a file save dialog pops up that allows you to select the file name and file format.

Zoom The graph visualizer has two buttons to zoom in and out. Also, the exact zoom desired can be entered in the zoom percentage entry. Hit enter to redraw at the desired zoom level.

Graph drawing options Hit the 'extra controls' button to show extra options that control the graph layout settings.

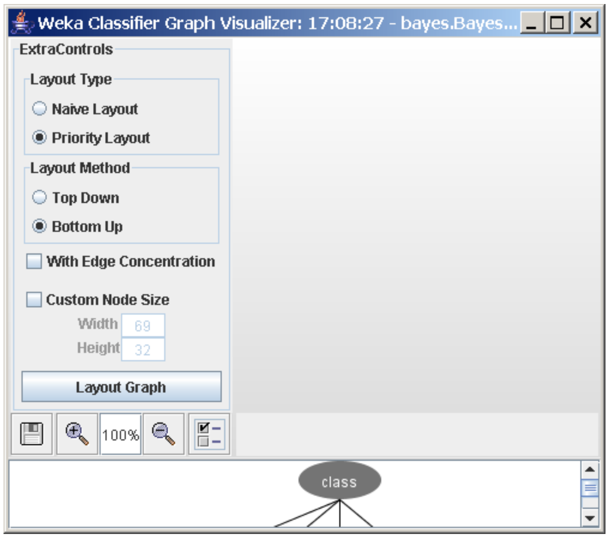

The `Layout Type` determines the algorithm applied to place the nodes.

The `Layout Method` determines in which direction nodes are considered.

The `Edge Concentration` toggle allows edges to be partially merged.

The `Custom Node Size` can be used to override the automatically determined node size.

When you click a node in the Bayesian net, a window with the probability table of the node clicked pops up. The left side shows the parent attributes and lists the values of the parents, the right side shows the probability of the node clicked conditioned on the values of the parents listed on the left.

Probability Distribution Table For petallength

class	sepallength	'(-inf-3.95]'	'(3.95-inf)'
Iris-setosa	'(-inf-6.1]'	0.99	0.01
Iris-setosa	'(6.1-inf)'	0.5	0.5
Iris-versicolor	'(-inf-6.1]'	0.329	0.671
Iris-versicolor	'(6.1-inf)'	0.029	0.971
Iris-virginica	'(-inf-6.1]'	0.042	0.958
Iris-virginica	'(6.1-inf)'	0.012	0.988

So, the graph visualizer allows you to inspect both network structure and probability tables.

8.9 Bayes Network GUI

The Bayesian network editor is a stand alone application with the following features

• Edit Bayesian network completely by hand, with unlimited undo/redo stack, cut/copy/paste and layout support.

• Learn Bayesian network from data using learning algorithms in Weka.

• Edit structure by hand and learn conditional probability tables (CPTs) using

learning algorithms in Weka.
• Generate dataset from Bayesian network.
• Inference (using junction tree method) of evidence through the network, interactively changing values of nodes.
• Viewing cliques in junction tree.
• Accelerator key support for most common operations.

The Bayes network GUI is started as
java weka.classifiers.bayes.net.GUI ¡bif file¿
The following window pops up when an XML BIF file is specified (if none is specified an empty graph is shown).

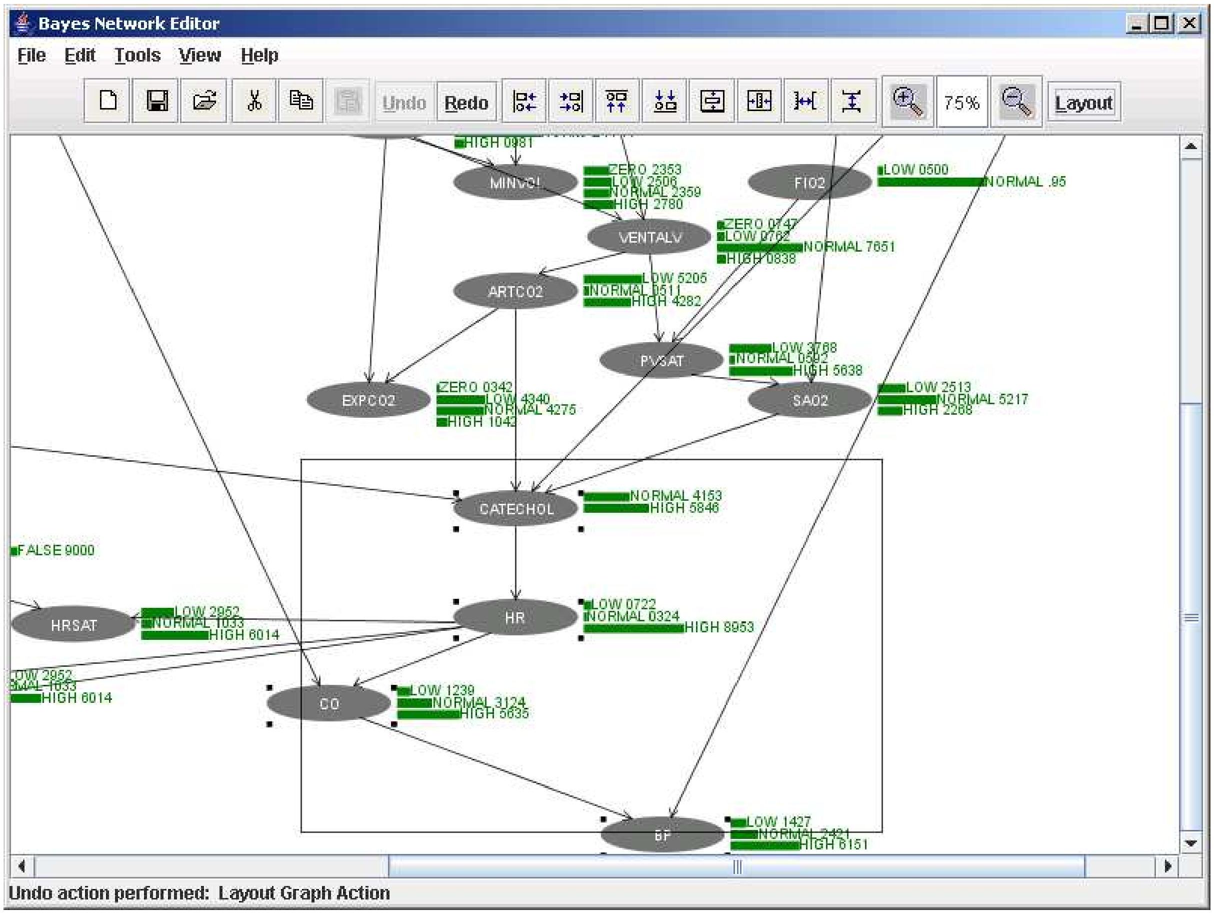

Moving a node

Click a node with the left mouse button and drag the node to the desired position.

Selecting groups of nodes

Drag the left mouse button in the graph panel. A rectangle is shown and all nodes intersecting with the rectangle are selected when the mouse is released. Selected nodes are made visible with four little black squares at the corners (see screenshot above).

The selection can be extended by keeping the shift key pressed while selecting another set of nodes.

The selection can be toggled by keeping the ctrl key pressed. All nodes in the selection selected in the rectangle are de-selected, while the ones not in the selection but intersecting with the rectangle are added to the selection.

Groups of nodes can be moved by keeping the left mouse pressed on one of the selected nodes and dragging the group to the desired position.

File menu

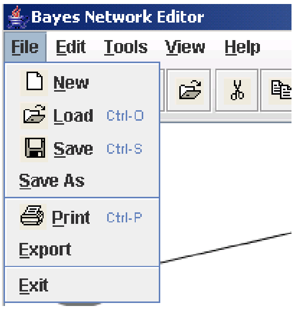

The New, Save, Save As, and Exit menu provide functionality as expected. The file format used is XML BIF [20].

There are two file formats supported for opening
• **.xml** for XML BIF files. The Bayesian network is reconstructed from the information in the file. Node width information is not stored so the nodes are shown with the default width. This can be changed by laying out the graph (menu Tools/Layout).
• **.arff** Weka data files. When an arff file is selected, a new empty Bayesian network is created with nodes for each of the attributes in the arff file. Continuous variables are discretized using the `weka.filters.supervised.attribute.Discretize` filter (see note at end of this section for more details). The network structure can be specified and the CPTs learned using the Tools/Learn CPT menu.

The Print menu works (sometimes) as expected.

The Export menu allows for writing the graph panel to image (currently supported are bmp, jpg, png and eps formats). This can also be activated using the Alt-Shift-Left Click action in the graph panel.

Edit menu

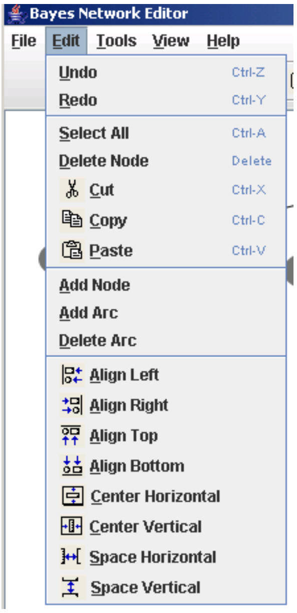

Unlimited undo/redo support. Most edit operations on the Bayesian network are undoable. A notable exception is learning of network and CPTs.

Cut/copy/paste support. When a set of nodes is selected these can be placed on a clipboard (internal, so no interaction with other applications yet) and a paste action will add the nodes. Nodes are renamed by adding "Copy of" before the name and adding numbers if necessary to ensure uniqueness of name. Only the arrows to parents are copied, not these of the children.

The Add Node menu brings up a dialog (see below) that allows to specify the name of the new node and the cardinality of the new node. Node values are assigned the names 'Value1', 'Value2' etc. These values can be renamed (right click the node in the graph panel and select Rename Value). Another option is to copy/paste a node with values that are already properly named and rename the node.

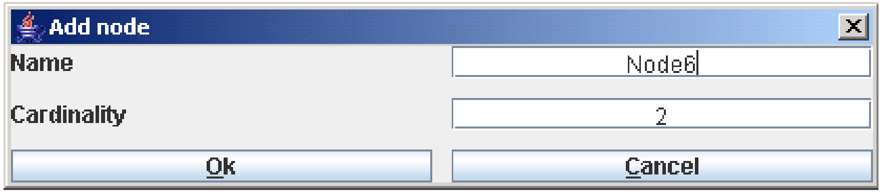

The Add Arc menu brings up a dialog to choose a child node first;

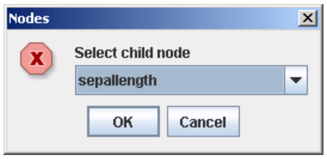

Then a dialog is shown to select a parent. Descendants of the child node, parents of the child node and the node itself are not listed since these cannot be selected as child node since they would introduce cycles or already have an arc in the network.

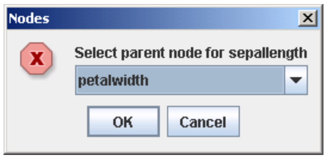

The Delete Arc menu brings up a dialog with a list of all arcs that can be deleted.

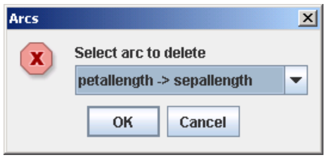

The list of eight items at the bottom are active only when a group of at least two nodes are selected.
• Align Left/Right/Top/Bottom moves the nodes in the selection such that all nodes align to the utmost left, right, top or bottom node in the selection respectively.
• Center Horizontal/Vertical moves nodes in the selection halfway between left and right most (or top and bottom most respectively).
• Space Horizontal/Vertical spaces out nodes in the selection evenly between left and right most (or top and bottom most respectively). The order in which the nodes are selected impacts the place the node is moved to.

Tools menu

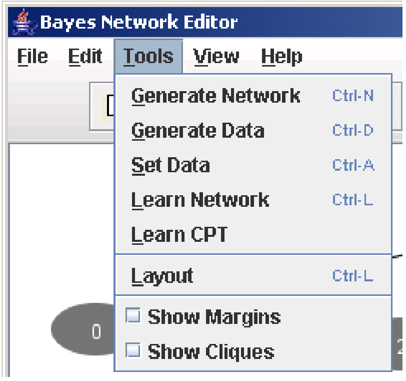

The Generate Network menu allows generation of a complete random Bayesian network. It brings up a dialog to specify the number of nodes, number of arcs, cardinality and a random seed to generate a network.

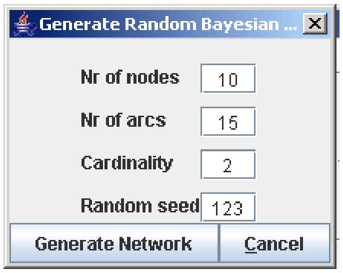

The Generate Data menu allows for generating a data set from the Bayesian network in the editor. A dialog is shown to specify the number of instances to be generated, a random seed and the file to save the data set into. The file format is arff. When no file is selected (field left blank) no file is written and only the internal data set is set.

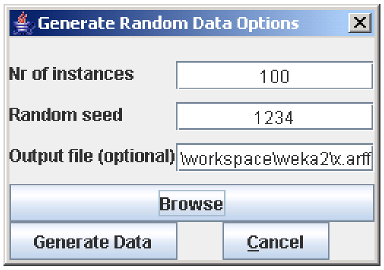

The Set Data menu sets the current data set. From this data set a new Bayesian network can be learned, or the CPTs of a network can be estimated. A file choose menu pops up to select the arff file containing the data.

The Learn Network and Learn CPT menus are only active when a data set is specified either through

- Tools/Set Data menu, or
- Tools/Generate Data menu, or
- File/Open menu when an arff file is selected.

The Learn Network action learns the whole Bayesian network from the data set. The learning algorithms can be selected from the set available in Weka by selecting the Options button in the dialog below. Learning a network clears the undo stack.

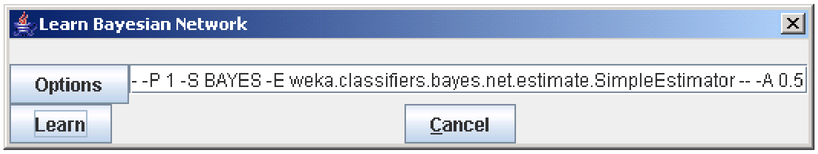

The Learn CPT menu does not change the structure of the Bayesian network, only the probability tables. Learning the CPTs clears the undo stack.

The Layout menu runs a graph layout algorithm on the network and tries to make the graph a bit more readable. When the menu item is selected, the node size can be specified or left to calculate by the algorithm based on the size of the labels by deselecting the custom node size check box.

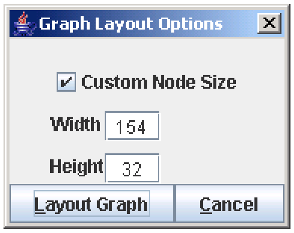

The Show Margins menu item makes marginal distributions visible. These are calculated using the junction tree algorithm [23]. Marginal probabilities for nodes are shown in green next to the node. The value of a node can be set (right click node, set evidence, select a value) and the color is changed to red to indicate evidence is set for the node. Rounding errors may occur in the marginal probabilities.

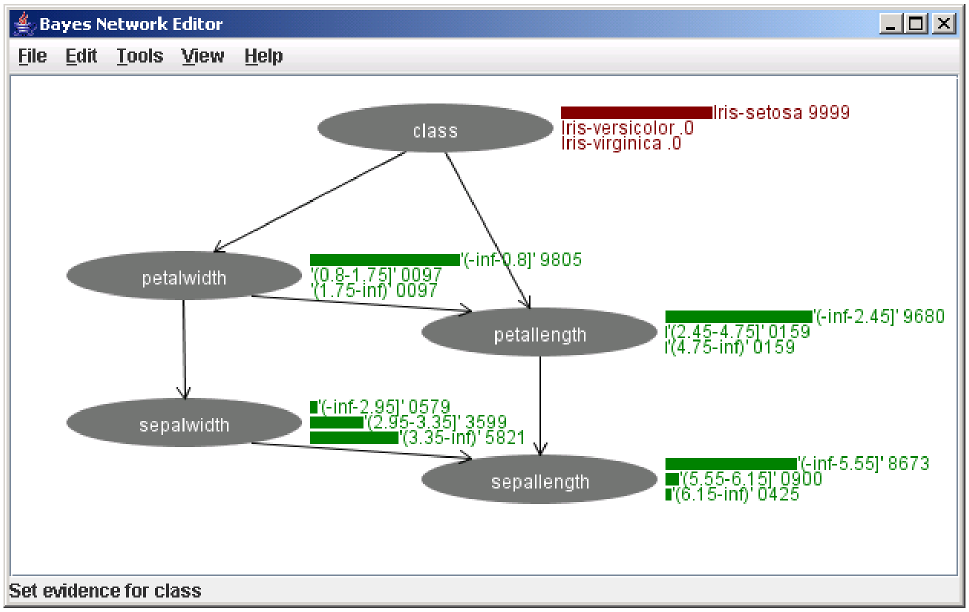

The Show Cliques menu item makes the cliques visible that are used by the junction tree algorithm. Cliques are visualized using colored undirected edges. Both margins and cliques can be shown at the same time, but that makes for rather crowded graphs.

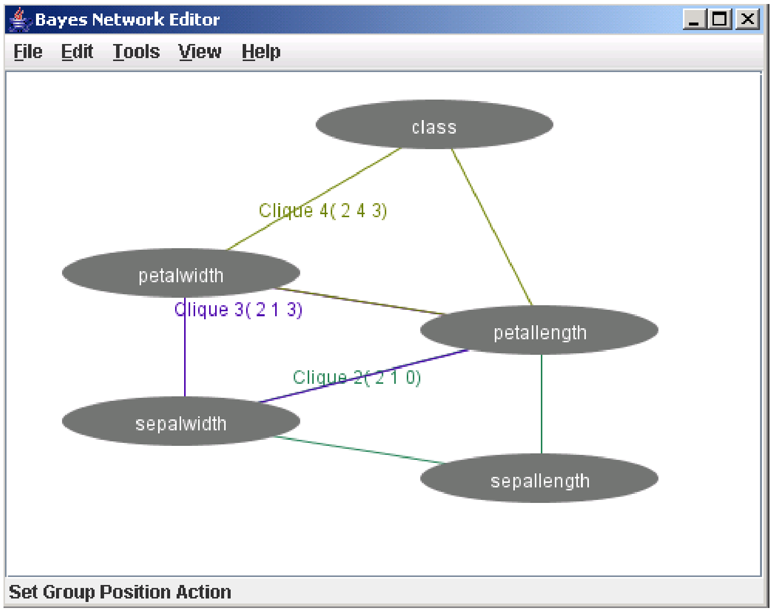

View menu

The view menu allows for zooming in and out of the graph panel. Also, it allows for hiding or showing the status and toolbars.

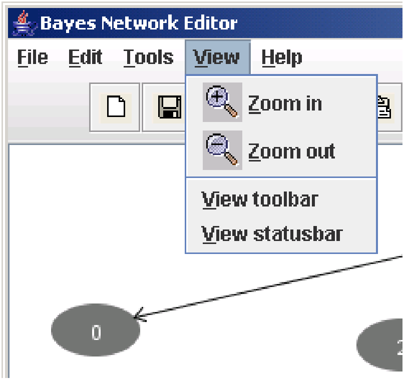

Help menu

The help menu points to this document.

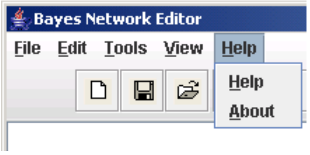

Toolbar

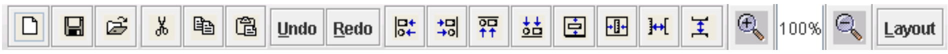

The toolbar allows a shortcut to many functions. Just hover the mouse over the toolbar buttons and a tooltiptext pops up that tells which function is activated. The toolbar can be shown or hidden with the View/View Toolbar menu.

Statusbar

At the bottom of the screen the statusbar shows messages. This can be helpful when an undo/redo action is performed that does not have any visible effects, such as edit actions on a CPT. The statusbar can be shown or hidden with the View/View Statusbar menu.

Click right mouse button

Clicking the right mouse button in the graph panel outside a node brings up the following popup menu. It allows to add a node at the location that was clicked, or add select a parent to add to all nodes in the selection. If no node is selected, or no node can be added as parent, this function is disabled.

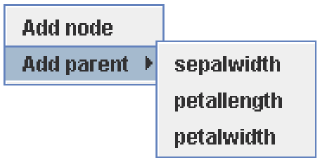

Clicking the right mouse button on a node brings up a popup menu.

The popup menu shows list of values that can be set as evidence to selected node. This is only visible when margins are shown (menu Tools/Show margins). By selecting 'Clear', the value of the node is removed and the margins calculated based on CPTs again.

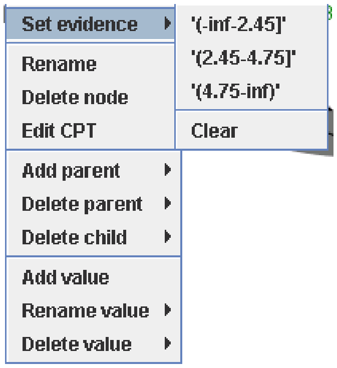

A node can be renamed by right click and select Rename in the popup menu. The following dialog appears that allows entering a new node name.

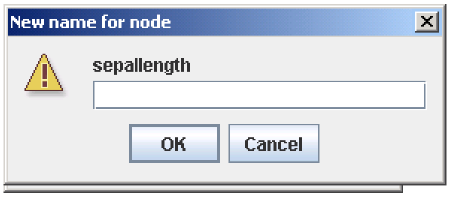

The CPT of a node can be edited manually by selecting a node, right click/Edit CPT. A dialog is shown with a table representing the CPT. When a value is edited, the values of the remainder of the table are update in order to ensure that the probabilities add up to 1. It attempts to adjust the last column first, then goes backward from there.

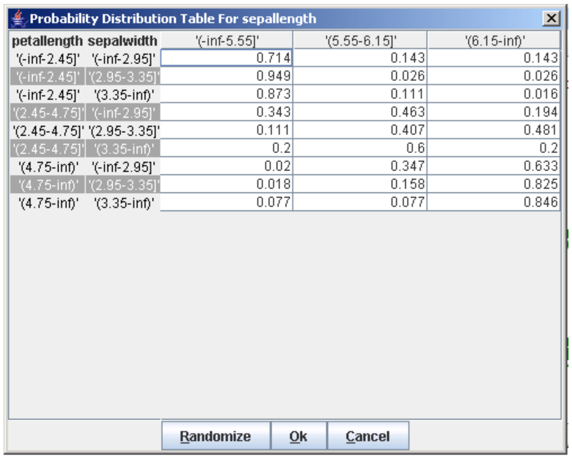

The whole table can be filled with randomly generated distributions by selecting the Randomize button.

The popup menu shows list of parents that can be added to selected node. CPT for the node is updated by making copies for each value of the new parent.

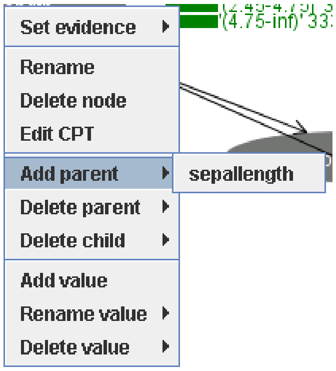

The popup menu shows list of parents that can be deleted from selected node. CPT of the node keeps only the one conditioned on the first value of the parent node.

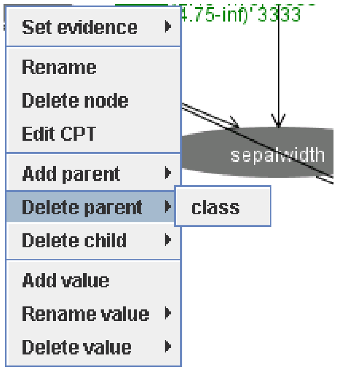

The popup menu shows list of children that can be deleted from selected node. CPT of the child node keeps only the one conditioned on the first value of the parent node.

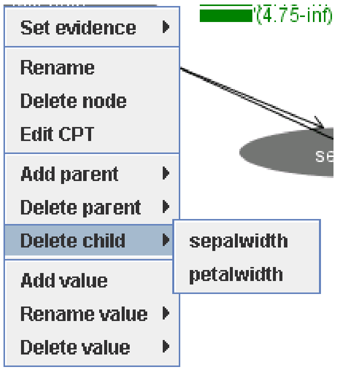

Selecting the Add Value from the popup menu brings up this dialog, in which the name of the new value for the node can be specified. The distribution for the node assign zero probability to the value. Child node CPTs are updated by copying distributions conditioned on the new value.

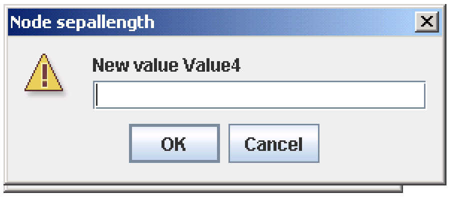

The popup menu shows list of values that can be renamed for selected node.

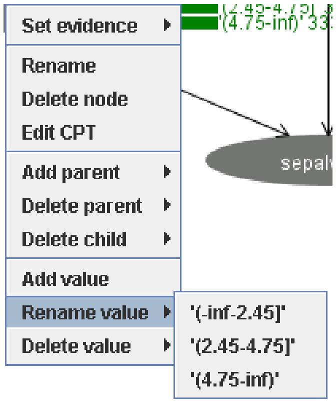

Selecting a value brings up the following dialog in which a new name can be specified.

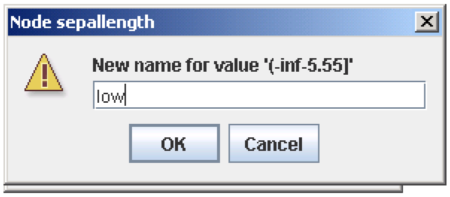

The popup menu shows list of values that can be deleted from selected node. This is only active when there are more then two values for the node (single valued nodes do not make much sense). By selecting the value the CPT of the node is updated in order to ensure that the CPT adds up to unity. The CPTs of children are updated by dropping the distributions conditioned on the value.

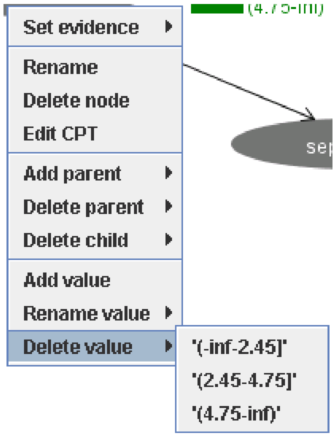

A note on CPT learning

Continuous variables are discretized by the Bayes network class. The discretization algorithm chooses its values based on the information in the data set.

However, these values are not stored anywhere. So, reading an arff file with continuous variables using the File/Open menu allows one to specify a network, then learn the CPTs from it since the discretization bounds are still known. However, opening an arff file, specifying a structure, then closing the application, reopening and trying to learn the network from another file containing continuous variables may not give the desired result since a the discretization algorithm is re-applied and new boundaries may have been found. Unexpected behavior may be the result.

Learning from a dataset that contains more attributes than there are nodes in the network is ok. The extra attributes are just ignored.

Learning from a dataset with differently ordered attributes is ok. Attributes are matched to nodes based on name. However, attribute values are matched with node values based on the order of the values.

The attributes in the dataset should have the same number of values as the corresponding nodes in the network (see above for continuous variables).

8.10 Bayesian nets in the experimenter

Bayesian networks generate extra measures that can be examined in the experimenter. The experimenter can then be used to calculate mean and variance for those measures.

The following metrics are generated:

- measureExtraArcs: extra arcs compared to reference network. The network must be provided as BIFFile to the BayesNet class. If no such network is provided, this value is zero.
- measureMissingArcs: missing arcs compared to reference network or zero if not provided.
- measureReversedArcs: reversed arcs compared to reference network or zero if not provided.
- measureDivergence: divergence of network learned compared to reference network or zero if not provided.
- measureBayesScore: log of the K2 score of the network structure.
- measureBDeuScore: log of the BDeu score of the network structure.
- measureMDLScore: log of the MDL score.
- measureAICScore: log of the AIC score.
- measureEntropyScore:log of the entropy.

8.11 Adding your own Bayesian network learners

You can add your own structure learners and estimators.

Adding a new structure learner

Here is the quick guide for adding a structure learner:

1. Create a class that derives from `weka.classifiers.bayes.net.search.SearchAlgorithm`. If your searcher is score based, conditional independence based or cross-validation based, you probably want to derive from `ScoreSearchAlgorithm`, `CISearchAlgorithm` or `CVSearchAlgorithm` instead of deriving from `SearchAlgorithm` directly. Let's say it is called `weka.classifiers.bayes.net.search.local.MySearcher` derived from `ScoreSearchAlgorithm`.

2. Implement the method `public void buildStructure(BayesNet bayesNet, Instances instances)`. Essentially, you are responsible for setting the parent sets in `bayesNet`. You can access the parentsets using `bayesNet.getParentSet(iAttribute)` where `iAttribute` is the number of the node/variable.

   To add a parent `iParent` to node `iAttribute`, use `bayesNet.getParentSet(iAttribute).AddParent(iParent, instances)` where `instances` need to be passed for the parent set to derive properties of the attribute.

   Alternatively, implement `public void search(BayesNet bayesNet, Instances instances)`. The implementation of `buildStructure` in the base class. This method is called by the `SearchAlgorithm` will call search after initializing parent sets and if the `initAsNaiveBase` flag is set, it will start with a naive Bayes network structure. After calling `search` in your custom class, it will add arrows if the `markovBlanketClassifier` flag is set to ensure all attributes are in the Markov blanket of the class node.

3. If the structure learner has options that are not default options, you want to implement `public Enumeration listOptions()`, `public void setOptions(String[] options)`, `public String[] getOptions()` and the get and set methods for the properties you want to be able to set.

   NB 1. do not use the -E option since that is reserved for the `BayesNet` class to distinguish the extra options for the `SearchAlgorithm` class and the `Estimator` class. If the -E option is used, it will not be passed to your `SearchAlgorithm` (and probably causes problems in the `BayesNet` class).

   NB 2. make sure to process options of the parent class if any in the get/setOpions methods.

Adding a new estimator

This is the quick guide for adding a new estimator:

1. Create a class that derives from `weka.classifiers.bayes.net.estimate.BayesNetEstimator`. Let's say it is called `weka.classifiers.bayes.net.estimate.MyEstimator`.

2. Implement the methods
   `public void initCPTs(BayesNet bayesNet)`

public void estimateCPTs(BayesNet bayesNet)
public void updateClassifier(BayesNet bayesNet, Instance instance), and
public double[] distributionForInstance(BayesNet bayesNet, Instance instance).

3. If the structure learner has options that are not default options, you want to implement `public Enumeration listOptions()`, `public void setOptions(String[] options)`, `public String[] getOptions()` and the get and set methods for the properties you want to be able to set.

   NB do not use the -E option since that is reserved for the BayesNet class to distinguish the extra options for the SearchAlgorithm class and the Estimator class. If the -E option is used and no extra arguments are passed to the SearchAlgorithm, the extra options to your Estimator will be passed to the SearchAlgorithm instead. In short, do not use the -E option.

8.12 FAQ

How do I use a data set with continuous variables with the BayesNet classes?

Use the class `weka.filters.unsupervised.attribute.Discretize` to discretize them. From the command line, you can use
`java weka.filters.unsupervised.attribute.Discretize -B 3 -i infile.arff -o outfile.arff`
where the -B option determines the cardinality of the discretized variables.

How do I use a data set with missing values with the BayesNet classes?

You would have to delete the entries with missing values or fill in dummy values.

How do I create a random Bayes net structure?

Running from the command line
`java weka.classifiers.bayes.net.BayesNetGenerator -B -N 10 -A 9 -C 2`
will print a Bayes net with 10 nodes, 9 arcs and binary variables in XML BIF format to standard output.

How do I create an artificial data set using a random Bayes nets?

Running
`java weka.classifiers.bayes.net.BayesNetGenerator -N 15 -A 20 -C 3 -M 300`
will generate a data set in arff format with 300 instance from a random network with 15 ternary variables and 20 arrows.

How do I create an artificial data set using a Bayes nets I have on file?

Running
`java weka.classifiers.bayes.net.BayesNetGenerator -F alarm.xml -M 1000`
will generate a data set with 1000 instances from the network stored in the file `alarm.xml`.

How do I save a Bayes net in BIF format?

- **GUI:** In the Explorer
  - learn the network structure,
  - right click the relevant run in the result list,
  - choose "Visualize graph" in the pop up menu,
  - click the floppy button in the Graph Visualizer window.
  - a file "save as" dialog pops up that allows you to select the file name to save to.
- **Java:** Create a `BayesNet` and call `BayesNet.toXMLBIF03()` which returns the Bayes network in BIF format as a String.
- **Command line:** use the `-g` option and redirect the output on stdout into a file.

How do I compare a network I learned with one in BIF format?

Specify the -B <bif-file> option to BayesNet. Calling `toString()` will produce a summary of extra, missing and reversed arrows. Also the divergence between the network learned and the one on file is reported.

How do I use the network I learned for general inference?

There is no general purpose inference in Weka, but you can export the network as XML BIF file (see above) and import it in other packages, for example JavaBayes available under GPL from `http://www.cs.cmu.edu/~javabayes`.

8.13 Future development

If you would like to add to the current Bayes network facilities in Weka, you might consider one of the following possibilities.

- Implement more search algorithms, in particular,
  - general purpose search algorithms (such as an improved implementation of genetic search).
  - structure search based on equivalent model classes.
  - implement those algorithms both for local and global metric based search algorithms.

  - implement more conditional independence based search algorithms.

- Implement score metrics that can handle sparse instances in order to allow for processing large datasets.

- Implement traditional conditional independence tests for conditional independence based structure learning algorithms.

- Currently, all search algorithms assume that all variables are discrete. Search algorithms that can handle continuous variables would be interesting.

- A limitation of the current classes is that they assume that there are no missing values. This limitation can be undone by implementing score metrics that can handle missing values. The classes used for estimating the conditional probabilities need to be updated as well.

- Only leave-one-out, k-fold and cumulative cross-validation are implemented. These implementations can be made more efficient and other cross-validation methods can be implemented, such as Monte Carlo cross-validation and bootstrap cross validation.

- Implement methods that can handle incremental extensions of the data set for updating network structures.

And for the more ambitious people, there are the following challenges.

- A GUI for manipulating Bayesian network to allow user intervention for adding and deleting arcs and updating the probability tables.

- General purpose inference algorithms built into the GUI to allow user defined queries.

- Allow learning of other graphical models, such as chain graphs, undirected graphs and variants of causal graphs.

- Allow learning of networks with latent variables.

- Allow learning of dynamic Bayesian networks so that time series data can be handled.

Part III

Data

Chapter 9

ARFF

An ARFF (= *Attribute-Relation File Format*) file is an ASCII text file that describes a list of instances sharing a set of attributes.

9.1 Overview

ARFF files have two distinct sections. The first section is the **Header** information, which is followed the **Data** information.

The **Header** of the ARFF file contains the name of the relation, a list of the attributes (the columns in the data), and their types. An example header on the standard IRIS dataset looks like this:

```
% 1. Title: Iris Plants Database
%
% 2. Sources:
%      (a) Creator: R.A. Fisher
%      (b) Donor: Michael Marshall (MARSHALL%PLU@io.arc.nasa.gov)
%      (c) Date: July, 1988
%
@RELATION iris

@ATTRIBUTE sepallength  NUMERIC
@ATTRIBUTE sepalwidth   NUMERIC
@ATTRIBUTE petallength  NUMERIC
@ATTRIBUTE petalwidth   NUMERIC
@ATTRIBUTE class        {Iris-setosa,Iris-versicolor,Iris-virginica}
```

The **Data** of the ARFF file looks like the following:

```
@DATA
5.1,3.5,1.4,0.2,Iris-setosa
4.9,3.0,1.4,0.2,Iris-setosa
4.7,3.2,1.3,0.2,Iris-setosa
4.6,3.1,1.5,0.2,Iris-setosa
5.0,3.6,1.4,0.2,Iris-setosa
5.4,3.9,1.7,0.4,Iris-setosa
```

```
4.6,3.4,1.4,0.3,Iris-setosa
5.0,3.4,1.5,0.2,Iris-setosa
4.4,2.9,1.4,0.2,Iris-setosa
4.9,3.1,1.5,0.1,Iris-setosa
```

Lines that begin with a `%` are comments. The `@RELATION`, `@ATTRIBUTE` and `@DATA` declarations are case insensitive.

9.2 Examples

Several well-known machine learning datasets are distributed with Weka in the `$WEKAHOME/data` directory as ARFF files.

9.2.1 The ARFF Header Section

The ARFF Header section of the file contains the relation declaration and attribute declarations.

The @relation Declaration

The relation name is defined as the first line in the ARFF file. The format is:

```
@relation <relation-name>
```

where <*relation-name*> is a string. The string must be quoted if the name includes spaces.

The @attribute Declarations

Attribute declarations take the form of an ordered sequence of `@attribute` statements. Each attribute in the data set has its own `@attribute` statement which uniquely defines the name of that attribute and it's data type. The order the attributes are declared indicates the column position in the data section of the file. For example, if an attribute is the third one declared then Weka expects that all that attributes values will be found in the third comma delimited column.

The format for the `@attribute` statement is:

```
@attribute <attribute-name> <datatype>
```

where the <*attribute-name*> must start with an alphabetic character. If spaces are to be included in the name then the entire name must be quoted.

The <*datatype*> can be any of the four types supported by Weka:

- *numeric*
- *integer* is treated as *numeric*
- *real* is treated as *numeric*
- <nominal-specification>
- *string*

- *date* [<date-format>]
- *relational* for multi-instance data (for future use)

where <*nominal-specification*> and <*date-format*> are defined below. The keywords **numeric**, **real**, **integer**, **string** and **date** are case insensitive.

Numeric attributes

Numeric attributes can be real or integer numbers.

Nominal attributes

Nominal values are defined by providing an <nominal-specification> listing the possible values: `<nominal-name1>, <nominal-name2>, <nominal-name3>, ...`

For example, the class value of the Iris dataset can be defined as follows:

```
@ATTRIBUTE class {Iris-setosa,Iris-versicolor,Iris-virginica}
```

Values that contain spaces must be quoted.

String attributes

String attributes allow us to create attributes containing arbitrary textual values. This is very useful in text-mining applications, as we can create datasets with string attributes, then write Weka Filters to manipulate strings (like StringToWordVectorFilter). String attributes are declared as follows:

```
@ATTRIBUTE LCC string
```

Date attributes

Date attribute declarations take the form:

```
@attribute <name> date [<date-format>]
```

where <*name*> is the name for the attribute and <*date-format*> is an optional string specifying how date values should be parsed and printed (this is the same format used by `SimpleDateFormat`). The default format string accepts the ISO-8601 combined date and time format: `yyyy-MM-dd'T'HH:mm:ss`.

Dates must be specified in the data section as the corresponding string representations of the date/time (see example below).

Relational attributes

Relational attribute declarations take the form:

```
@attribute <name> relational
  <further attribute definitions>
@end <name>
```

For the multi-instance dataset MUSK1 the definition would look like this ("..." denotes an omission):

```
@attribute molecule_name {MUSK-jf78,...,NON-MUSK-199}
@attribute bag relational
  @attribute f1 numeric
  ...
  @attribute f166 numeric
@end bag
@attribute class {0,1}
...
```

9.2.2 The ARFF Data Section

The ARFF Data section of the file contains the data declaration line and the actual instance lines.

The @data Declaration

The `@data` declaration is a single line denoting the start of the data segment in the file. The format is:

```
@data
```

The instance data

Each instance is represented on a single line, with carriage returns denoting the end of the instance. A percent sign (`%`) introduces a comment, which continues to the end of the line.

Attribute values for each instance are delimited by commas. They must appear in the order that they were declared in the header section (i.e. the data corresponding to the nth `@attribute` declaration is always the nth field of the attribute).

Missing values are represented by a single question mark, as in:

```
@data
4.4,?,1.5,?,Iris-setosa
```

Values of string and nominal attributes are case sensitive, and any that contain space or the comment-delimiter character `%` must be quoted. (The code suggests that double-quotes are acceptable and that a backslash will escape individual characters.) An example follows:

```
@relation LCCvsLCSH

@attribute LCC string
@attribute LCSH string

@data
AG5,   'Encyclopedias and dictionaries.;Twentieth century.'
AS262, 'Science -- Soviet Union -- History.'
AE5,   'Encyclopedias and dictionaries.'
AS281, 'Astronomy, Assyro-Babylonian.;Moon -- Phases.'
AS281, 'Astronomy, Assyro-Babylonian.;Moon -- Tables.'
```

Dates must be specified in the data section using the string representation specified in the attribute declaration. For example:

```
@RELATION Timestamps

@ATTRIBUTE timestamp DATE "yyyy-MM-dd HH:mm:ss"

@DATA
"2001-04-03 12:12:12"
"2001-05-03 12:59:55"
```

Relational data must be enclosed within double quotes ”. For example an instance of the MUSK1 dataset (”...” denotes an omission):

```
MUSK-188,"42,...,30",1
```

9.3 Sparse ARFF files

Sparse ARFF files are very similar to ARFF files, but data with value 0 are not be explicitly represented.

Sparse ARFF files have the same header (i.e `@relation` and `@attribute` tags) but the data section is different. Instead of representing each value in order, like this:

```
@data
0, X, 0, Y, "class A"
0, 0, W, 0, "class B"
```

the non-zero attributes are explicitly identified by attribute number and their value stated, like this:

```
@data
{1 X, 3 Y, 4 "class A"}
{2 W, 4 "class B"}
```

Each instance is surrounded by curly braces, and the format for each entry is: <index> <space> <value> where index is the attribute index (starting from 0).

Note that the omitted values in a sparse instance are **0**, they are not **missing** values! If a value is unknown, you must explicitly represent it with a question mark (?).

Warning: There is a known problem saving `SparseInstance` objects from datasets that have string attributes. In Weka, string and nominal data values are stored as numbers; these numbers act as indexes into an array of possible attribute values (this is very efficient). However, the first string value is assigned index 0: this means that, internally, this value is stored as a 0. When a `SparseInstance` is written, string instances with internal value 0 are not output, so their string value is lost (and when the arff file is read again, the default value 0 is the index of a different string value, so the attribute value appears to change). To get around this problem, add a dummy string value at index 0 that is never used whenever you declare string attributes that are likely to be used in `SparseInstance` objects and saved as Sparse ARFF files.

9.4 Instance weights in ARFF files

A weight can be associated with an instance in a standard ARFF file by appending it to the end of the line for that instance and enclosing the value in curly braces. E.g:

```
@data
0, X, 0, Y, "class A", {5}
```

For a sparse instance, this example would look like:

```
@data
{1 X, 3 Y, 4 "class A"}, {5}
```

Note that any instance without a weight value specified is assumed to have a weight of 1 for backwards compatibility.

Chapter 10

XRFF

The XRFF (*Xml attribute Relation File Format*) is a representing the data in a format that can store comments, attribute and instance weights.

10.1 File extensions

The following file extensions are recognized as XRFF files:

- `.xrff`
  the default extension of XRFF files

- `.xrff.gz`
  the extension for gzip compressed XRFF files (see *Compression* section for more details)

10.2 Comparison

10.2.1 ARFF

In the following a snippet of the UCI dataset *iris* in ARFF format:

```
@relation iris

@attribute sepallength numeric
@attribute sepalwidth numeric
@attribute petallength numeric
@attribute petalwidth numeric
@attribute class {Iris-setosa,Iris-versicolor,Iris-virginica}

@data
5.1,3.5,1.4,0.2,Iris-setosa
4.9,3,1.4,0.2,Iris-setosa
...
```

10.2.2 XRFF

And the same dataset represented as XRFF file:

```
<?xml version="1.0" encoding="utf-8"?>

<!DOCTYPE dataset
[
   <!ELEMENT dataset (header,body)>
   <!ATTLIST dataset name CDATA #REQUIRED>
   <!ATTLIST dataset version CDATA "3.5.4">

   <!ELEMENT header (notes?,attributes)>
   <!ELEMENT body (instances)>
   <!ELEMENT notes ANY>

   <!ELEMENT attributes (attribute+)>
   <!ELEMENT attribute (labels?,metadata?,attributes?)>
   <!ATTLIST attribute name CDATA #REQUIRED>
   <!ATTLIST attribute type (numeric|date|nominal|string|relational) #REQUIRED>
   <!ATTLIST attribute format CDATA #IMPLIED>
   <!ATTLIST attribute class (yes|no) "no">
   <!ELEMENT labels (label*)>
   <!ELEMENT label ANY>
   <!ELEMENT metadata (property*)>
   <!ELEMENT property ANY>
   <!ATTLIST property name CDATA #REQUIRED>

   <!ELEMENT instances (instance*)>
   <!ELEMENT instance (value*)>
   <!ATTLIST instance type (normal|sparse) "normal">
   <!ATTLIST instance weight CDATA #IMPLIED>
   <!ELEMENT value (#PCDATA|instances)*>
   <!ATTLIST value index CDATA #IMPLIED>
   <!ATTLIST value missing (yes|no) "no">
]
>

<dataset name="iris" version="3.5.3">
   <header>
      <attributes>
         <attribute name="sepallength" type="numeric"/>
         <attribute name="sepalwidth" type="numeric"/>
         <attribute name="petallength" type="numeric"/>
         <attribute name="petalwidth" type="numeric"/>
         <attribute class="yes" name="class" type="nominal">
            <labels>
               <label>Iris-setosa</label>
               <label>Iris-versicolor</label>
               <label>Iris-virginica</label>
            </labels>
```

```
      </attribute>
    </attributes>
  </header>

  <body>
    <instances>
      <instance>
        <value>5.1</value>
        <value>3.5</value>
        <value>1.4</value>
        <value>0.2</value>
        <value>Iris-setosa</value>
      </instance>
      <instance>
        <value>4.9</value>
        <value>3</value>
        <value>1.4</value>
        <value>0.2</value>
        <value>Iris-setosa</value>
      </instance>
      ...
    </instances>
  </body>
</dataset>
```

10.3 Sparse format

The XRFF format also supports a sparse data representation. Even though the iris dataset does not contain sparse data, the above example will be used here to illustrate the sparse format:

```
...
<instances>
  <instance type="sparse">
    <value index="1">5.1</value>
    <value index="2">3.5</value>
    <value index="3">1.4</value>
    <value index="4">0.2</value>
    <value index="5">Iris-setosa</value>
  </instance>
  <instance type="sparse">
    <value index="1">4.9</value>
    <value index="2">3</value>
    <value index="3">1.4</value>
    <value index="4">0.2</value>
    <value index="5">Iris-setosa</value>
  </instance>
  ...
</instances>
...
```

In contrast to the *normal* data format, each sparse instance tag contains a type attribute with the value sparse:

```
<instance type="sparse">
```

And each value tag needs to specify the *index* attribute, which contains the 1-based index of this value.

```
<value index="1">5.1</value>
```

10.4 Compression

Since the XML representation takes up considerably more space than the rather compact ARFF format, one can also compress the data via **gzip**. Weka automatically recognizes a file being gzip compressed, if the file's extension is `.xrff.gz` instead of `.xrff`.

The Weka Explorer, Experimenter and command-line allow one to load/save compressed and uncompressed XRFF files (this applies also to ARFF files).

10.5 Useful features

In addition to all the features of the ARFF format, the XRFF format contains the following additional features:

- class attribute specification
- attribute weights

10.5.1 Class attribute specification

Via the `class="yes"` attribute in the attribute specification in the header, one can define which attribute should act as class attribute. A feature that can be used on the command line as well as in the Experimenter, which now can also load other data formats, and removing the limitation of the class attribute always having to be the last one.

Snippet from the *iris* dataset:

```
<attribute class="yes" name="class" type="nominal">
```

10.5.2 Attribute weights

Attribute weights are stored in an attributes meta-data tag (in the *header* section). Here is an example of the *petalwidth* attribute with a weight of 0.9:

```
<attribute name="petalwidth" type="numeric">
    <metadata>
       <property name="weight">0.9</property>
    </metadata>
</attribute>
```

10.5.3 Instance weights

Instance weights are defined via the *weight* attribute in each instance tag. By default, the weight is 1. Here is an example:

```
<instance weight="0.75">
   <value>5.1</value>
   <value>3.5</value>
   <value>1.4</value>
   <value>0.2</value>
   <value>Iris-setosa</value>
</instance>
```

Chapter 11

Converters

11.1 Introduction

Weka offers conversion utilities for several formats, in order to allow import from different sorts of datasources. These utilities, called converters, are all located in the following package:

```
weka.core.converters
```

For a certain kind of converter you will find two classes

- one for **loading** (classname ends with *Loader*) and
- one for **saving** (classname ends with *Saver*).

Weka contains converters for the following data sources:

- **ARFF** files (ArffLoader, ArffSaver)
- **C4.5** files (C45Loader, C45Saver)
- **CSV** files (CSVLoader, CSVSaver)
- files containing **serialized instances** (SerializedInstancesLoader, SerializedInstancesSaver)
- JDBC **databases** (DatabaseLoader, DatabaseSaver)
- **libsvm** files (LibSVMLoader, LibSVMSaver)
- **XRFF** files (XRFFLoader, XRFFSaver)
- **text directories** for text mining (TextDirectoryLoader)

11.2 Usage

11.2.1 File converters

File converters can be used as follows:

- **Loader**
  They take one argument, which is the file that should be converted, and print the result to stdout. You can also redirect the output into a file:

  ```
  java <classname> <input-file> > <output-file>
  ```

  Here's an example for loading the CSV file *iris.csv* and saving it as *iris.arff*:

  ```
  java weka.core.converters.CSVLoader iris.csv > iris.arff
  ```

- **Saver**
  For a Saver you specify the ARFF input file via *-i* and the output file in the specific format with *-o*:

  ```
  java <classname> -i <input> -o <output>
  ```

  Here's an example for saving an ARFF file to CSV:

  ```
  java weka.core.converters.CSVSaver -i iris.arff -o iris.csv
  ```

A few notes:

- Using the *ArffSaver* from commandline doesn't make much sense, since this Saver takes an ARFF file as input **and** output. The *ArffSaver* is normally used from Java for saving an object of `weka.core.Instances` to a file.
- The *C45Loader* either takes the *.names*-file or the *.data*-file as input, it automatically looks for the other one.
- For the *C45Saver* one specifies as output file a filename without any extension, since two output files will be generated; *.names* and *.data* are automatically appended.

11.2.2 Database converters

The database converters are a bit more complex, since they also rely on additional configuration files, besides the parameters on the commandline. The setup for the database connection is stored in the following props file:

```
DatabaseUtils.props
```

The default file can be found here:

```
weka/experiment/DatabaseUtils.props
```

- **Loader**
  You have to specify at least a SQL query with the -*Q* option (there are additional options for incremental loading)

  ```
  java weka.core.converters.DatabaseLoader -Q "select * from employee"
  ```

- **Saver**
  The Saver takes an ARFF file as input like any other Saver, but then also the table where to save the data to via -*T*:

  ```
  java weka.core.converters.DatabaseSaver -i iris.arff -T iris
  ```

Chapter 12

Stemmers

12.1 Introduction

Weka now supports stemming algorithms. The stemming algorithms are located in the following package:

```
weka.core.stemmers
```

Currently, the Lovins Stemmer (+ iterated version) and support for the Snowball stemmers are included.

12.2 Snowball stemmers

Weka contains a wrapper class for the Snowball (homepage: `http://snowball.tartarus.org/`) stemmers (containing the Porter stemmer and several other stemmers for different languages). The relevant class is `weka.core.stemmers.Snowball`.

The Snowball classes are not included, they only have to be present in the classpath. The reason for this is, that the Weka team doesn't have to watch out for new versions of the stemmers and update them.

There are two ways of getting hold of the Snowball stemmers:

1. You can add the following pre-compiled jar archive to your classpath and you're set (based on source code from 2005-10-19, compiled 2005-10-22).
   `http://www.cs.waikato.ac.nz/~ml/weka/stemmers/snowball.jar`

2. You can compile the stemmers yourself with the newest sources. Just download the following ZIP file, unpack it and follow the instructions in the README file (the zip contains an ANT (`http://ant.apache.org/`) build script for generating the jar archive).
   `http://www.cs.waikato.ac.nz/~ml/weka/stemmers/snowball.zip`
   Note: the patch target is specific to the source code from 2005-10-19.

12.3 Using stemmers

The stemmers can either used

- from commandline
- within the StringToWordVector (package `weka.filters.unsupervised.attribute`)

12.3.1 Commandline

All stemmers support the following options:

- *-h*
  for displaying a brief help
- *-i <input-file>*
  The file to process
- *-o <output-file>*
  The file to output the processed data to (default *stdout*)
- *-l*
  Uses lowercase strings, i.e., the input is automatically converted to lower case

12.3.2 StringToWordVector

Just use the GenericObjectEditor to choose the right stemmer and the desired options (if the stemmer offers additional options).

12.4 Adding new stemmers

You can easily add new stemmers, if you follow these guidelines (for use in the GenericObjectEditor):

- they should be located in the `weka.core.stemmers` package (if not, then the `GenericObjectEditor.props/GenericPropertiesCreator.props` file need to be updated) and
- they must implement the interface `weka.core.stemmers.Stemmer`.

Chapter 13

Databases

13.1 Configuration files

Thanks to JDBC it is easy to connect to Databases that provide a JDBC driver. Responsible for the setup is the following properties file, located in the weka.experiment package:

```
DatabaseUtils.props
```

You can get this properties file from the weka.jar or weka-src.jar jar-archive, both part of a normal Weka release. If you open up one of those files, you'll find the properties file in the sub-folder weka/experiment.

Weka comes with example files for a wide range of databases:

- `DatabaseUtils.props.hsql` - HSQLDB (>=3.4.1)
- `DatabaseUtils.props.msaccess` - MS Access (>3.4.14, >3.5.8, >3.6.0) see the *Windows databases* chapter for more information.
- `DatabaseUtils.props.mssqlserver` - MS SQL Server 2000 (>=3.4.9, >=3.5.4)
- `DatabaseUtils.props.mssqlserver2005` - MS SQL Server 2005 (>=3.4.11, >=3.5.6)
- `DatabaseUtils.props.mysql` - MySQL (>=3.4.9, >=3.5.4)
- `DatabaseUtils.props.odbc` - ODBC access via Sun's ODBC/JDBC bridge, e.g., for MS Sql Server (>=3.4.9, >=3.5.4)
  see the *Windows databases* chapter for more information.
- `DatabaseUtils.props.oracle` - Oracle 10g (>=3.4.9, >=3.5.4)
- `DatabaseUtils.props.postgresql` - PostgreSQL 7.4 (>=3.4.9, >=3.5.4)
- `DatabaseUtils.props.sqlite3` - sqlite 3.x (>3.4.12, >3.5.7)

The easiest way is just to place the extracted properties file into your HOME directory. For more information on how property files are processed, check out the following URL:

`http://weka.wikispaces.com/Properties+File`

Note: Weka *only* looks for the `DatabaseUtils.props` file. If you take one of the example files listed above, you need to rename it first.

13.2 Setup

Under normal circumstances you only have to edit the following two properties:

- `jdbcDriver`
- `jdbcURL`

Driver

`jdbcDriver` is the classname of the JDBC driver, necessary to connect to your database, e.g.:

- HSQLDB
  `org.hsqldb.jdbcDriver`
- MS SQL Server 2000 (Desktop Edition)
  `com.microsoft.jdbc.sqlserver.SQLServerDriver`
- MS SQL Server 2005
  `com.microsoft.sqlserver.jdbc.SQLServerDriver`
- MySQL
  `org.gjt.mm.mysql.Driver` (or `com.mysql.jdbc.Driver`)
- ODBC - part of Sun's JDKs/JREs, no external driver necessary
  `sun.jdbc.odbc.JdbcOdbcDriver`
- Oracle
  `oracle.jdbc.driver.OracleDriver`
- PostgreSQL
  `org.postgresql.Driver`
- sqlite 3.x
  `org.sqlite.JDBC`

URL

`jdbcURL` specifies the JDBC URL pointing to your database (can be still changed in the Experimenter/Explorer), e.g. for the database MyDatabase on the server `server.my.domain`:

- HSQLDB
  `jdbc:hsqldb:hsql://server.my.domain/MyDatabase`

- MS SQL Server 2000 (Desktop Edition)
  `jdbc:microsoft:sqlserver://v:1433`
  (Note: if you add `;databasename=db-name` you can connect to a different database than the default one, e.g., MyDatabase)

- MS SQL Server 2005
  `jdbc:sqlserver://server.my.domain:1433`

- MySQL
  `jdbc:mysql://server.my.domain:3306/MyDatabase`

- ODBC
  `jdbc:odbc:DSN_name` (replace *DSN_name* with the DSN that you want to use)

- Oracle (thin driver)
  `jdbc:oracle:thin:@server.my.domain:1526:orcl`
  (Note: `@machineName:port:SID`)
  for the *Express Edition* you can use
  `jdbc:oracle:thin:@server.my.domain:1521:XE`

- PostgreSQL
  `jdbc:postgresql://server.my.domain:5432/MyDatabase`
  You can also specify user and password directly in the URL:
  `jdbc:postgresql://server.my.domain:5432/MyDatabase?user=<...>&password=<...>`
  where you have to replace the <...> with the correct values

- sqlite 3.x
  `jdbc:sqlite:/path/to/database.db`
  (you can access only local files)

13.3 Missing Datatypes

Sometimes (e.g. with MySQL) it can happen that a column type cannot be interpreted. In that case it is necessary to map the name of the column type to the Java type it should be interpreted as. E.g. the MySQL type TEXT is returned as BLOB from the JDBC driver and has to be mapped to String (0 represents String - the mappings can be found in the comments of the properties file):

Java type	Java method	Identifier	Weka attribute type
String	getString()	0	nominal
boolean	getBoolean()	1	nominal
double	getDouble()	2	numeric
byte	getByte()	3	numeric
short	getByte()	4	numeric
int	getInteger()	5	numeric
long	getLong()	6	numeric
float	getFloat()	7	numeric
date	getDate()	8	date
text	getString()	9	string
time	getTime()	10	date

In the props file one lists now the type names that the database returns and what Java type it represents (via the identifier), e.g.:

```
CHAR=0
VARCHAR=0
```

`CHAR` and `VARCHAR` are both String types, hence they are interpreted as String (identifier 0)
Note: in case database types have blanks, one needs to replace those blanks with an underscore, e.g., DOUBLE PRECISION must be listed like this:

```
DOUBLE_PRECISION=2
```

13.4 Stored Procedures

Let's say you're tired of typing the same query over and over again. A good way to shorten that, is to create a stored procedure.

PostgreSQL 7.4.x

The following example creates a procedure called emplyoee_name that returns the names of all the employees in table employee. Even though it doesn't make much sense to create a stored procedure for this query, nonetheless, it shows how to create and call stored procedures in PostgreSQL.

- Create

```
CREATE OR REPLACE FUNCTION public.employee_name()
  RETURNS SETOF text AS 'select name from employee'
  LANGUAGE 'sql' VOLATILE;
```

- SQL statement to call procedure

```
SELECT * FROM employee_name()
```

- Retrieve data via `InstanceQuery`

```
java weka.experiment.InstanceQuery
     -Q "SELECT * FROM employee_name()"
     -U <user> -P <password>
```

13.5 Troubleshooting

- In case you're experiencing problems connecting to your database, check out the WEKA Mailing List (see Weka homepage for more information). It is possible that somebody else encountered the same problem as you and you'll find a post containing the solution to your problem.
- Specific *MS SQL Server 2000* Troubleshooting
  - Error Establishing Socket with JDBC Driver
    Add TCP/IP to the list of protocols as stated in the following article:
    `http://support.microsoft.com/default.aspx?scid=kb;en-us;313178`
  - Login failed for user 'sa'. Reason: Not associated with a trusted SQL Server connection.
    For changing the authentication to mixed mode see the following article:
    `http://support.microsoft.com/kb/319930/en-us`
- *MS SQL Server 2005*: TCP/IP is not enabled for SQL Server, or the server or port number specified is incorrect.Verify that SQL Server is listening with TCP/IP on the specified server and port. This might be reported with an exception similar to: "The login has failed. The TCP/IP connection to the host has failed." This indicates one of the following:
  - SQL Server is installed but TCP/IP has not been installed as a network protocol for SQL Server by using the SQL Server Network Utility for SQL Server 2000, or the SQL Server Configuration Manager for SQL Server 2005
  - TCP/IP is installed as a SQL Server protocol, but it is not listening on the port specified in the JDBC connection URL. The default port is 1433.
  - The port that is used by the server has not been opened in the firewall
- The **Added driver: ...** output on the commandline does not mean that the actual class was found, but only that Weka will *attempt* to load the class later on in order to establish a database connection.
- The error message No suitable driver can be caused by the following:
  - The JDBC driver you are attempting to load is not in the CLASSPATH (Note: using `-jar` in the java commandline **overwrites** the CLASSPATH environment variable!). Open the SimpleCLI, run the command `java weka.core.SystemInfo` and check whether the property `java.class.path` lists your database jar. If not correct your CLASSPATH or the Java call you start Weka with.
  - The JDBC driver class is misspelled in the `jdbcDriver` property or you have multiple entries of `jdbcDriver` (properties files need *unique keys*!)
  - The jdbcURL property has a spelling error and tries to use a non-existing protocol or you listed it multiple times, which doesn't work either (remember, properties files need unique keys!)

Chapter 14

Windows databases

A common query we get from our users is how to open a Windows database in the Weka Explorer. This page is intended as a guide to help you achieve this. It is a complicated process and we cannot guarantee that it will work for you. The process described makes use of the JDBC-ODBC bridge that is part of Sun's JRE/JDK 1.3 (and higher).

The following instructions are for Windows 2000. Under other Windows versions there may be slight differences.

Step 1: Create a User DSN

1. Go to the **Control Panel**
2. Choose **Adminstrative Tools**
3. Choose **Data Sources (ODBC)**
4. At the **User DSN** tab, choose **Add...**
5. Choose database
    - Microsoft Access
        (a) Note: Make sure your database is not open in another application before following the steps below.
        (b) Choose the **Microsoft Access** driver and click **Finish**
        (c) Give the source a name by typing it into the **Data Source Name** field
        (d) In the **Database** section, choose **Select...**
        (e) Browse to find your database file, select it and click **OK**
        (f) Click **OK** to finalize your DSN
    - Microsoft SQL Server 2000 (Desktop Engine)
        (a) Choose the **SQL Server** driver and click **Finish**
        (b) Give the source a name by typing it into the **Name** field
        (c) Add a description for this source in the **Description** field
        (d) Select the server you're connecting to from the **Server** combobox

(e) For the verification of the authenticity of the login ID choose **With SQL Server...**

(f) Check **Connect to SQL Server to obtain default settings...** and supply the user ID and password with which you installed the Desktop Engine

(g) Just click on **Next** until it changes into **Finish** and click this, too

(h) For testing purposes, click on **Test Data Source...** - the result should be *TESTS COMPLETED SUCCESSFULLY!*

(i) Click on **OK**

- MySQL

  (a) Choose the **MySQL ODBC** driver and click **Finish**

  (b) Give the source a name by typing it into the **Data Source Name** field

  (c) Add a description for this source in the **Description** field

  (d) Specify the server you're connecting to in **Server**

  (e) Fill in the user to use for connecting to the database in the **User** field, the same for the password

  (f) Choose the database for this DSN from the **Database** combobox

  (g) Click on **OK**

6. Your DSN should now be listed in the **User Data Sources** list

Step 2: Set up the DatabaseUtils.props file

You will need to configure a file called `DatabaseUtils.props`. This file already exists under the path `weka/experiment/` in the `weka.jar` file (which is just a ZIP file) that is part of the Weka download. In this directory you will also find a sample file for ODBC connectivity, called `DatabaseUtils.props.odbc`, and one specifically for MS Access, called `DatabaseUtils.props.msaccess`, also using ODBC. You should use one of the sample files as basis for your setup, since they already contain default values specific to ODBC access.

This file needs to be recognized when the Explorer starts. You can achieve this by making sure it is in the working directory or the home directory (if you are unsure what the terms *working directory* and *home directory* mean, see the *Notes* section). The easiest is probably the second alternative, as the setup will apply to all the Weka instances on your machine.

Just make sure that the file contains the following lines at least:

```
jdbcDriver=sun.jdbc.odbc.JdbcOdbcDriver
jdbcURL=jdbc:odbc:dbname
```

where *dbname* is the name you gave the user DSN. (This can also be changed once the Explorer is running.)

Step 3: Open the database

1. Start up the Weka Explorer.
2. Choose **Open DB...**
3. The **URL** should read "jdbc:odbc:*dbname*" where *dbname* is the name you gave the user DSN.
4. Click **Connect**
5. Enter a **Query**, e.g., "`select * from` *`tablename`*" where *tablename* is the name of the database table you want to read. Or you could put a more complicated SQL query here instead.
6. Click **Execute**
7. When you're satisfied with the returned data, click **OK** to load the data into the Preprocess panel.

Notes

- **Working directory**
  The directory a process is started from. When you start Weka from the Windows Start Menu, then this directory would be Weka's installation directory (the java process is started from that directory).
- **Home directory**
  The directory that contains all the user's data. The exact location depends on the operating system and the version of the operating system. It is stored in the following environment variable:
  - Unix/Linux
    `$HOME`
  - Windows
    `%USERPROFILE%`
  - Cygwin
    `$USERPROFILE`

  You should be able output the value in a command prompt/terminal with the echo command. E.g., for Windows this would be:

  ```
  echo %USERPROFILE%
  ```

Part IV

Appendix

Chapter 15

Research

15.1 Citing Weka

If you want to refer to Weka in a publication, please cite following SIGKDD Explorations[1] paper. The full citation is:

> Mark Hall, Eibe Frank, Geoffrey Holmes, Bernhard Pfahringer, Peter Reutemann, Ian H. Witten (2009); *The WEKA Data Mining Software: An Update*; SIGKDD Explorations, Volume 11, Issue 1.

15.2 Paper references

Due to the introduction of the `weka.core.TechnicalInformationHandler` interface it is now easy to extract all the paper references via `weka.core.ClassDiscovery` and `weka.core.TechnicalInformation`.

The script listed at the end, extracts all the paper references from Weka based on a given jar file and dumps it to stdout. One can either generate simple plain text output (option `-p`) or BibTeX compliant one (option `-b`).

Typical use (after an `ant exejar`) for BibTeX:

```
get_wekatechinfo.sh -d ../ -w ../dist/weka.jar -b > ../tech.txt
```

(command is issued from the same directory the Weka `build.xml` is located in)

[1] `http://www.kdd.org/explorations/issues/11-1-2009-07/p2V11n1.pdf`

Bash shell script get_wekatechinfo.sh

```
#!/bin/bash
#
# This script prints the information stored in TechnicalInformationHandlers
# to stdout.
#
# FracPete, $Revision: 4582 $

# the usage of this script
function usage()
{
   echo
   echo "${0##*/} -d <dir> [-w <jar>] [-p|-b] [-h]"
   echo
   echo "Prints the information stored in TechnicalInformationHandlers to stdout."
   echo
   echo " -h   this help"
   echo " -d   <dir>"
   echo "      the directory to look for packages, must be the one just above"
   echo "      the 'weka' package, default: $DIR"
   echo " -w   <jar>"
   echo "      the weka jar to use, if not in CLASSPATH"
   echo " -p   prints the information in plaintext format"
   echo " -b   prints the information in BibTeX format"
   echo
}

# generates a filename out of the classname TMP and returns it in TMP
# uses the directory in DIR
function class_to_filename()
{
  TMP=$DIR"/"`echo $TMP | sed s/"\."/"\/"/g`".java"
}

# variables
DIR="."
PLAINTEXT="no"
BIBTEX="no"
WEKA=""
TECHINFOHANDLER="weka.core.TechnicalInformationHandler"
TECHINFO="weka.core.TechnicalInformation"
CLASSDISCOVERY="weka.core.ClassDiscovery"

# interprete parameters
while getopts ":hpbw:d:" flag
do
   case $flag in
      p) PLAINTEXT="yes"
         ;;
      b) BIBTEX="yes"
         ;;
      d) DIR=$OPTARG
         ;;
      w) WEKA=$OPTARG
         ;;
      h) usage
         exit 0
         ;;
      *) usage
         exit 1
         ;;
   esac
done

# either plaintext or bibtex
if [ "$PLAINTEXT" = "$BIBTEX" ]
then
   echo
   echo "ERROR: either -p or -b has to be given!"
   echo
   usage
   exit 2
fi
```

```
# do we have everything?
if [ "$DIR" = "" ] || [ ! -d "$DIR" ]
then
   echo
   echo "ERROR: no directory or non-existing one provided!"
   echo
   usage
   exit 3
fi

# generate Java call
if [ "$WEKA" = "" ]
then
  JAVA="java"
else
  JAVA="java -classpath $WEKA"
fi
if [ "$PLAINTEXT" = "yes" ]
then
  CMD="$JAVA $TECHINFO -plaintext"
elif [ "$BIBTEX" = "yes" ]
then
  CMD="$JAVA $TECHINFO -bibtex"
fi

# find packages
TMP=`find $DIR -mindepth 1 -type d | grep -v CVS | sed s/".*weka"/"weka"/g | sed s/"\/"/./g`
PACKAGES=`echo $TMP | sed s/" "/,/g`

# get technicalinformationhandlers
TECHINFOHANDLERS=`$JAVA weka.core.ClassDiscovery $TECHINFOHANDLER $PACKAGES | grep "\. weka" | sed s/".*weka"/weka/g`

# output information
echo
for i in $TECHINFOHANDLERS
do
  TMP=$i;class_to_filename

  # exclude internal classes
  if [ ! -f $TMP ]
  then
    continue
  fi

  $CMD -W $i
  echo
done
```

Chapter 16

Using the API

Using the graphical tools, like the Explorer, or just the command-line is in most cases sufficient for the normal user. But WEKA's clearly defined API ("application programming interface") makes it very easy to "embed" it in another projects. This chapter covers the basics of how to achieve the following common tasks from source code:

- Setting options
- Creating datasets in memory
- Loading and saving data
- Filtering
- Classifying
- Clustering
- Selecting attributes
- Visualization
- Serialization

Even though most of the code examples are for the Linux platform, using forward slashes in the paths and file names, they do work on the MS Windows platform as well. To make the examples work under MS Windows, one only needs to adapt the paths, changing the forward slashes to backslashes and adding a drive letter where necessary.

Note
WEKA is released under the GNU General Public License version 2[1] (GPLv2), i.e., that derived code or code that uses WEKA needs to be released under the GPLv2 as well. If one is just using WEKA for a personal project that does not get released publicly then one is not affected. But as soon as one makes the project publicly available (e.g., for download), then one needs to make the source code available under the GLPv2 as well, alongside the binaries.

[1] http://www.gnu.org/licenses/gpl-2.0.html

16.1 Option handling

Configuring an object, e.g., a classifier, can either be done using the appropriate `get`/`set`-methods for the property that one wishes to change, like the Explorer does. Or, if the class implements the `weka.core.OptionHandler` interface, one can just use the object's ability to parse command-line options via the `setOptions(String[])` method (the counterpart of this method is `getOptions()`, which returns a `String[]` array). The difference between the two approaches is, that the `setOptions(String[])` method cannot be used to set the options incrementally. Default values are used for all options that haven't been explicitly specified in the options array.

The most basic approach is to assemble the `String` array by hand. The following example creates an array with a single option ("`-R`") that takes an argument ("`1`") and initializes the `Remove` filter with this option:

```
import weka.filters.unsupervised.attribute.Remove;
...
String[] options = new String[2];
options[0] = "-R";
options[1] = "1";
Remove rm = new Remove();
rm.setOptions(options);
```

Since the `setOptions(String[])` method expects a fully parsed and correctly split up array (which is done by the console/command prompt), some common pitfalls with this approach are:

- Combination of option and argument – Using "`-R 1`" as an element of the `String` array will fail, prompting WEKA to output an error message stating that the option "R 1" is unknown.
- Trailing blanks – Using "`-R `" will fail as well, since no trailing blanks are removed and therefore option "R " will not be recognized.

The easiest way to avoid these problems, is to provide a `String` array that has been generated automatically from a single command-line string using the `splitOptions(String)` method of the `weka.core.Utils` class. Here is an example:

```
import weka.core.Utils;
...
String[] options = Utils.splitOptions("-R 1");
```

As this method ignores whitespaces, using "` -R 1`" or "`-R 1 `" will return the same result as "`-R 1`".

Complicated command-lines with lots of nested options, e.g., options for the support-vector machine classifier *SMO* (package `weka.classifiers.functions`) including a kernel setup, are a bit tricky, since Java requires one to escape double quotes and backslashes inside a `String`. The Wiki[2] article "Use Weka in your Java code" references the Java class `OptionsToCode`, which turns any command-line into appropriate Java source code. This example class is also available from the *Weka Examples* collection[3]: `weka.core.OptionsToCode`.

Instead of using the `Remove` filter's `setOptions(String[])` method, the following code snippet uses the actual `set`-method for this property:

```
import weka.filters.unsupervised.attribute.Remove;
...
Remove rm = new Remove();
rm.setAttributeIndices("1");
```

In order to find out, which option belongs to which property, i.e., `get/set`-method, it is best to have a look at the `setOptions(String[])` and `getOptions()` methods. In case these methods use the member variables directly, one just has to look for the methods making this particular member variable accessible to the outside.

Using the `set`-methods, one will most likely come across ones that require a `weka.core.SelectedTag` as parameter. An example for this, is the `setEvaluation` method of the meta-classifier `GridSearch` (located in package `weka.classifiers.meta`). The `SelectedTag` class is used in the GUI for displaying drop-down lists, enabling the user to chose from a predefined list of values. `GridSearch` allows the user to chose the statistical measure to base the evaluation on (accuracy, correlation coefficient, etc.).

A `SelectedTag` gets constructed using the array of all possible `weka.core.Tag` elements that can be chosen and the integer or string ID of the `Tag`. For instance, `GridSearch`'s `setOptions(String[])` method uses the supplied string ID to set the evaluation type (e.g., "`ACC`" for accuracy), or, if the evaluation option is missing, the default integer ID `EVALUATION_ACC`. In both cases, the array `TAGS_EVALUATION` is used, which defines all possible options:

```
import weka.core.SelectedTag;
...
String tmpStr = Utils.getOption('E', options);
if (tmpStr.length() != 0)
  setEvaluation(new SelectedTag(tmpStr, TAGS_EVALUATION));
else
  setEvaluation(new SelectedTag(EVALUATION_CC, TAGS_EVALUATION));
```

16.2 Loading data

Before any filter, classifier or clusterer can be applied, data needs to be present. WEKA enables one to load data from files (in various file formats) and also from databases. In the latter case, it is assumed in that the database connection is set up and working. See chapter 13 for more details on how to configure WEKA correctly and also more information on JDBC (Java Database Connectivity) URLs.

Example classes, making use of the functionality covered in this section, can be found in the `wekaexamples.core.converters` package of the *Weka Examples* collection[3].

The following classes are used to store data in memory:

- `weka.core.Instances` – holds a complete dataset. This data structure is row-based; single rows can be accessed via the `instance(int)` method using a 0-based index. Information about the columns can be accessed via the `attribute(int)` method. This method returns `weka.core.Attribute` objects (see below).
- `weka.core.Instance` – encapsulates a single row. It is basically a wrapper around an array of double primitives. Since this class contains no information about the type of the columns, it always needs access to a `weka.core.Instances` object (see methods `dataset` and `setDataset`). The class `weka.core.SparseInstance` is used in case of sparse data.
- `weka.core.Attribute` – holds the type information about a single column in the dataset. It stores the type of the attribute, as well as the labels for *nominal* attributes, the possible values for *string* attributes or the datasets for *relational* attributes (these are just `weka.core.Instances` objects again).

16.2.1 Loading data from files

When loading data from files, one can either let WEKA choose the appropriate loader (the available loaders can be found in the `weka.core.converters` package) based on the file's extension or one can use the correct loader directly. The latter case is necessary if the files do not have the correct extension.

The `DataSource` class (inner class of the `weka.core.converters.ConverterUtils` class) can be used to read data from files that have the appropriate file extension. Here are some examples:

```
import weka.core.converters.ConverterUtils.DataSource;
import weka.core.Instances;
...
Instances data1 = DataSource.read("/some/where/dataset.arff");
Instances data2 = DataSource.read("/some/where/dataset.csv");
Instances data3 = DataSource.read("/some/where/dataset.xrff");
```

In case the file does have a different file extension than is normally associated with the loader, one has to use a loader directly. The following example loads a CSV ("comma-separated values") file:

```
import weka.core.converters.CSVLoader;
```

```
import weka.core.Instances;
import java.io.File;
...
CSVLoader loader = new CSVLoader();
loader.setSource(new File("/some/where/some.data"));
Instances data = loader.getDataSet();
```

NB: Not all file formats allow to store information about the class attribute (e.g., ARFF stores no information about class attribute, but XRFF does). If a class attribute is required further down the road, e.g., when using a classifier, it can be set with the `setClassIndex(int)` method:

```
// uses the first attribute as class attribute
if (data.classIndex() == -1)
   data.setClassIndex(0);
...
// uses the last attribute as class attribute
if (data.classIndex() == -1)
   data.setClassIndex(data.numAttributes() - 1);
```

16.2.2 Loading data from databases

For loading data from databases, one of the following two classes can be used:

- `weka.experiment.InstanceQuery`
- `weka.core.converters.DatabaseLoader`

The differences between them are, that the `InstanceQuery` class allows one to retrieve sparse data and the `DatabaseLoader` can retrieve the data incrementally.

Here is an example of using the `InstanceQuery` class:

```
import weka.core.Instances;
import weka.experiment.InstanceQuery;
...
InstanceQuery query = new InstanceQuery();
query.setDatabaseURL("jdbc_url");
query.setUsername("the_user");
query.setPassword("the_password");
query.setQuery("select * from whatsoever");
// if your data is sparse, then you can say so, too:
// query.setSparseData(true);
Instances data = query.retrieveInstances();
```

And an example using the `DatabaseLoader` class in "batch retrieval":

```
import weka.core.Instances;
import weka.core.converters.DatabaseLoader;
...
DatabaseLoader loader = new DatabaseLoader();
loader.setSource("jdbc_url", "the_user", "the_password");
loader.setQuery("select * from whatsoever");
Instances data = loader.getDataSet();
```

The DatabaseLoader is used in "incremental mode" as follows:

```
import weka.core.Instance;
import weka.core.Instances;
import weka.core.converters.DatabaseLoader;
...
DatabaseLoader loader = new DatabaseLoader();
loader.setSource("jdbc_url", "the_user", "the_password");
loader.setQuery("select * from whatsoever");
Instances structure = loader.getStructure();
Instances data = new Instances(structure);
Instance inst;
while ((inst = loader.getNextInstance(structure)) != null)
   data.add(inst);
```

Notes:

- Not all database systems allow incremental retrieval.
- Not all queries have a unique key to retrieve rows incrementally. In that case, one can supply the necessary columns with the `setKeys(String)` method (comma-separated list of columns).
- If the data cannot be retrieved in an incremental fashion, it is first fully loaded into memory and then provided row-by-row ("pseudo-incremental").

16.3 Creating datasets in memory

Loading datasets from disk or database are not the only ways of obtaining data in WEKA: datasets can be created in memory or *on-the-fly*. Generating a dataset memory structure (i.e., a `weka.core.Instances` object) is a two-stage process:

1. Defining the format of the data by setting up the attributes.
2. Adding the actual data, row by row.

The class `wekaexamples.core.CreateInstances` of the *Weka Examples* collection[3] generates an `Instances` object containing all attribute types WEKA can handle at the moment.

16.3.1 Defining the format

There are currently five different types of attributes available in WEKA:

- **numeric** – continuous variables
- **date** – date variables
- **nominal** – predefined labels
- **string** – textual data
- **relational** – contains other relations, e.g., the bags in case of multi-instance data

For all of the different attribute types, WEKA uses the same class, `weka.core.Attribute`, but with different constructors. In the following, these different constructors are explained.

- **numeric** – The easiest attribute type to create, as it requires only the name of the attribute:

  ```
  Attribute numeric = new Attribute("name_of_attr");
  ```

- `date` – Date attributes are handled internally as numeric attributes, but in order to parse and present the date value correctly, the format of the date needs to be specified. The *date* and *time patterns* are explained in detail in the Javadoc of the `java.text.SimpleDateFormat` class. In the following, an example of how to create a date attribute using a date format of 4-digit year, 2-digit month and a 2-digit day, separated by hyphens:

  ```
  Attribute date = new Attribute("name_of_attr", "yyyy-MM-dd");
  ```

- **nominal** – Since nominal attributes contain predefined labels, one needs to supply these, stored in form of a `weka.core.FastVector` object:

  ```
  FastVector labels = new FastVector();
  labels.addElement("label_a");
  labels.addElement("label_b");
  labels.addElement("label_c");
  labels.addElement("label_d");
  Attribute nominal = new Attribute("name_of_attr", labels);
  ```

- **string** – In contrast to nominal attributes, this type does not store a predefined list of labels. Normally used to store textual data, i.e., content of documents for text categorization. The same constructor as for the nominal attribute is used, but a `null` value is provided instead of an instance of `FastVector`:

  ```
  Attribute string = new Attribute("name_of_attr", (FastVector) null);
  ```

- relational – This attribute just takes another weka.core.Instances object for defining the relational structure in the constructor. The following code snippet generates a relational attribute that contains a relation with two attributes, a numeric and a nominal attribute:

```
FastVector atts = new FastVector();
atts.addElement(new Attribute("rel.num"));
FastVector values = new FastVector();
values.addElement("val_A");
values.addElement("val_B");
values.addElement("val_C");
atts.addElement(new Attribute("rel.nom", values));
Instances rel_struct = new Instances("rel", atts, 0);
Attribute relational = new Attribute("name_of_attr", rel_struct);
```

A weka.core.Instances object is then created by supplying a FastVector object containing all the attribute objects. The following example creates a dataset with two numeric attributes and a nominal class attribute with two labels "no" and "yes":

```
Attribute num1 = new Attribute("num1");
Attribute num2 = new Attribute("num2");
FastVector labels = new FastVector();
labels.addElement("no");
labels.addElement("yes");
Attribute cls = new Attribute("class", labels);
FastVector attributes = new FastVector();
attributes.addElement(num1);
attributes.addElement(num2);
attributes.addElement(cls);
Instances dataset = new Instances("Test-dataset", attributes, 0);
```

The final argument in the Instances constructor above tells WEKA how much memory to reserve for upcoming weka.core.Instance objects. If one knows how many rows will be added to the dataset, then it should be specified as it saves costly operations for expanding the internal storage. It doesn't matter, if one aims to high with the amount of rows to be added, it is always possible to *trim* the dataset again, using the compactify() method.

16.3.2 Adding data

After the structure of the dataset has been defined, one can add the actual data to it, row by row. There are basically two constructors of the weka.core.Instance class that one can use for this purpose:

- Instance(double weight, double[] attValues) – generates an Instance object with the specified weight and the given double values. WEKA's internal format is using doubles for all attribute types. For nominal, string and relational attributes this is just an index of the stored values.
- Instance(int numAttributes) – generates a new Instance object with weight 1.0 and all missing values.

The second constructor may be easier to use, but setting values via the Instance class' methods is a bit costly, especially if one is adding a lot of rows. Therefore, the following code examples cover the first constructor. For simplicity, an Instances object "data" based on the code snippets for the different attribute introduced used above is used, as it contains all possible attribute types.

For each instance, the first step is to create a new double array to hold the attribute values. It is important not to reuse this array, but always create a new one, since WEKA only references it and does not create a copy of it, when instantiating the `Instance` object. Reusing means changing the previously generated `Instance` object:

```
double[] values = new double[data.numAttributes()];
```

After that, the double array is filled with the actual values:

- **numeric** – just sets the numeric value:

  ```
  values[0] = 1.23;
  ```

- **date** – turns the date string into a double value:

  ```
  values[1] = data.attribute(1).parseDate("2001-11-09");
  ```

- **nominal** – determines the index of the label:

  ```
  values[2] = data.attribute(2).indexOf("label_b");
  ```

- **string** – determines the index of the string, using the `addStringValue` method (internally, a hashtable holds all the string values):

  ```
  values[3] = data.attribute(3).addStringValue("This is a string");
  ```

- **relational** – first, a new `Instances` object based on the attribute's relational definition has to be created, before the index of it can be determined, using the `addRelation` method:

  ```
  Instances dataRel = new Instances(data.attribute(4).relation(),0);
  valuesRel = new double[dataRel.numAttributes()];
  valuesRel[0] = 2.34;
  valuesRel[1] = dataRel.attribute(1).indexOf("val_C");
  dataRel.add(new Instance(1.0, valuesRel));
  values[4] = data.attribute(4).addRelation(dataRel);
  ```

Finally, an `Instance` object is generated with the initialized double array and added to the dataset:

```
Instance inst = new Instance(1.0, values);
data.add(inst);
```

16.4 Randomizing data

Since learning algorithms can be prone to the order the data arrives in, randomizing (also called "shuffling") the data is a common approach to alleviate this problem. Especially repeated randomizations, e.g., as during cross-validation, help to generate more realistic statistics.

WEKA offers two possibilities for randomizing a dataset:

- Using the `randomize(Random)` method of the `weka.core.Instances` object containing the data itself. This method requires an instance of the `java.util.Random` class. How to correctly instantiate such an object is explained below.
- Using the `Randomize` filter (package `weka.filters.unsupervised.instance`). For more information on how to use filters, see section 16.5.

A very important aspect of Machine Learning experiments is, that experiments have to be repeatable. Subsequent runs of the same experiment setup have to yield the exact same results. It may seem weird, but randomization is still possible in this scenario. Random number generates never return a completely random sequence of numbers anyway, only a pseudo-random one. In order to achieve repeatable pseudo-random sequences, *seeded* generators are used. Using the same *seed value* will always result in the same sequence then.

The default constructor of the `java.util.Random` random number generator class should never be used, as such created objects will generate most likely different sequences. The constructor `Random(long)`, using a specified seed value, is the recommended one to use.

In order to get a more dataset-dependent randomization of the data, the `getRandomNumberGenerator(int)` method of the `weka.core.Instances` class can be used. This method returns a `java.util.Random` object that was seeded with the sum of the supplied seed and the hashcode of the string representation of a randomly chosen `weka.core.Instance` of the `Instances` object (using a random number generator seeded with the seed supplied to this method).

16.5 Filtering

In WEKA, filters are used to preprocess the data. They can be found below package `weka.filters`. Each filter falls into one of the following two categories:

- *supervised* – The filter requires a class attribute to be set.
- *unsupervised* – A class attribute is not required to be present.

And into one of the two sub-categories:

- *attribute-based* – Columns are processed, e.g., added or removed.
- *instance-based* – Rows are processed, e.g., added or deleted.

These categories should make it clear, what the difference between the two `Discretize` filters in WEKA is. The *supervised* one takes the class attribute and its distribution over the dataset into account, in order to determine the optimal number and size of bins, whereas the *unsupervised* one relies on a user-specified number of bins.

Apart from this classification, filters are either *stream-* or *batch-based*. *Stream* filters can process the data straight away and make it immediately available for collection again. *Batch* filters, on the other hand, need a batch of data to setup their internal data structures. The `Add` filter (this filter can be found in the `weka.filters.unsupervised.attribute` package) is an example of a stream filter. Adding a new attribute with only missing values does not require any sophisticated setup. However, the `ReplaceMissingValues` filter (same package as the `Add` filter) needs a batch of data in order to determine the means and modes for each of the attributes. Otherwise, the filter will not be able to replace the missing values with meaningful values. But as soon as a batch filter has been initialized with the first batch of data, it can also process data on a row-by-row basis, just like a stream filter.

Instance-based filters are a bit special in the way they handle data. As mentioned earlier, *all* filters can process data on a row-by-row basis after the first batch of data has been passed through. Of course, if a filter adds or removes rows from a batch of data, this no longer works when working in single-row processing mode. This makes sense, if one thinks of a scenario involving the `FilteredClassifier` meta-classifier: after the training phase (= first batch of data), the classifier will get evaluated against a test set, one instance at a time. If the filter now removes the only instance or adds instances, it can no longer be evaluated correctly, as the evaluation expects to get only a single result back. This is the reason why *instance-based* filters only pass through any subsequent batch of data without processing it. The `Resample` filters, for instance, act like this.

One can find example classes for filtering in the `wekaexamples.filters` package of the *Weka Examples* collection[3].

The following example uses the `Remove` filter (the filter is located in package `weka.filters.unsupervised.attribute`) to remove the first attribute from a dataset. For setting the options, the `setOptions(String[])` method is used.

```
import weka.core.Instances;
import weka.filters.Filter;
import weka.filters.unsupervised.attribute.Remove;
...
String[] options = new String[2];
options[0] = "-R";                    // "range"
options[1] = "1";                     // first attribute
Remove remove = new Remove();         // new instance of filter
remove.setOptions(options);           // set options
remove.setInputFormat(data);          // inform filter about dataset
                                      // **AFTER** setting options
Instances newData = Filter.useFilter(data, remove); // apply filter
```

A common trap to fall into is setting options **after** the `setInputFormat(Instances)` has been called. Since this method is (normally) used to determine the output format of the data, **all** the options have to be set **before** calling it. Otherwise, all options set afterwards will be ignored.

16.5.1 Batch filtering

Batch filtering is necessary if two or more datasets need to be processed according to the same filter initialization. If batch filtering is not used, for instance when generating a training and a test set using the `StringToWordVector` filter (package `weka.filters.unsupervised.attribute`), then these two filter runs are completely independent and will create two most likely incompatible datasets. Running the `StringToWordVector` on two different datasets, this will result in two different word dictionaries and therefore different attributes being generated.

The following code example shows how to standardize, i.e., transforming all numeric attributes to have zero mean and unit variance, a training and a test set with the `Standardize` filter (package `weka.filters.unsupervised.attribute`):

```
Instances train = ...   // from somewhere
Instances test = ...    // from somewhere
Standardize filter = new Standardize();
// initializing the filter once with training set
filter.setInputFormat(train);
// configures the Filter based on train instances and returns
// filtered instances
Instances newTrain = Filter.useFilter(train, filter);
// create new test set
Instances newTest = Filter.useFilter(test, filter);
```

16.5.2 Filtering on-the-fly

Even though using the API gives one full control over the data and makes it easier to juggle several datasets at the same time, filtering data **on-the-fly** makes life even easier. This handy feature is available through meta schemes in WEKA, like `FilteredClassifier` (package `weka.classifiers.meta`), `FilteredClusterer` (package `weka.clusterers`), `FilteredAssociator` (package `weka.associations`) and `FilteredAttributeEval`/`FilteredSubsetEval` (in `weka.attributeSelection`). Instead of filtering the data *beforehand*, one just sets up a meta-scheme and lets the meta-scheme do the filtering for one.

The following example uses the `FilteredClassifier` in conjunction with the `Remove` filter to remove the first attribute (which happens to be an ID attribute) from the dataset and `J48` (J48 is WEKA's implementation of C4.5; package `weka.classifiers.trees`) as base-classifier. First the classifier is built with a training set and then evaluated with a separate test set. The actual and predicted class values are printed in the console. For more information on classification, see chapter 16.6.

```
import weka.classifiers.meta.FilteredClassifier;
import weka.classifiers.trees.J48;
import weka.core.Instances;
import weka.filters.unsupervised.attribute.Remove;
...
Instances train = ...         // from somewhere
Instances test = ...          // from somewhere
// filter
Remove rm = new Remove();
rm.setAttributeIndices("1");  // remove 1st attribute
// classifier
J48 j48 = new J48();
j48.setUnpruned(true);        // using an unpruned J48
// meta-classifier
FilteredClassifier fc = new FilteredClassifier();
fc.setFilter(rm);
fc.setClassifier(j48);
// train and output model
fc.buildClassifier(train);
System.out.println(fc);
for (int i = 0; i < test.numInstances(); i++) {
  double pred = fc.classifyInstance(test.instance(i));
  double actual = test.instance(i).classValue();
  System.out.print("ID: "
    + test.instance(i).value(0));
  System.out.print(", actual: "
    + test.classAttribute().value((int) actual));
  System.out.println(", predicted: "
    + test.classAttribute().value((int) pred));
}
```

16.6 Classification

Classification and regression algorithms in WEKA are called "classifiers" and are located below the `weka.classifiers` package. This section covers the following topics:

- *Building a classifier* – batch and incremental learning.
- *Evaluating a classifier* – various evaluation techniques and how to obtain the generated statistics.
- *Classifying instances* – obtaining classifications for unknown data.

The *Weka Examples* collection[3] contains example classes covering classification in the `wekaexamples.classifiers` package.

16.6.1 Building a classifier

By design, all classifiers in WEKA are *batch-trainable*, i.e., they get trained on the whole dataset at once. This is fine, if the training data fits into memory. But there are also algorithms available that can update their internal model *on-the-go*. These classifiers are called *incremental*. The following two sections cover the batch and the incremental classifiers.

Batch classifiers

A batch classifier is really simple to build:

- *set options* – either using the `setOptions(String[])` method or the actual set-methods.
- *train it* – calling the `buildClassifier(Instances)` method with the training set. By definition, the `buildClassifier(Instances)` method resets the internal model completely, in order to ensure that subsequent calls of this method with the same data result in the same model ("repeatable experiments").

The following code snippet builds an unpruned J48 on a dataset:

```
import weka.core.Instances;
import weka.classifiers.trees.J48;
...
Instances data = ...              // from somewhere
String[] options = new String[1];
options[0] = "-U";                // unpruned tree
J48 tree = new J48();             // new instance of tree
tree.setOptions(options);         // set the options
tree.buildClassifier(data);       // build classifier
```

Incremental classifiers

All incremental classifiers in WEKA implement the interface `UpdateableClassifier` (located in package `weka.classifiers`). Bringing up the Javadoc for this particular interface tells one what classifiers implement this interface. These classifiers can be used to process large amounts of data with a small memory-footprint, as the training data does not have to fit in memory. ARFF files, for instance, can be read incrementally (see chapter 16.2).

Training an incremental classifier happens in *two* stages:

1. *initialize* the model by calling the `buildClassifier(Instances)` method. One can either use a `weka.core.Instances` object with no actual data or one with an initial set of data.
2. *update* the model row-by-row, by calling the `updateClassifier(Instance)` method.

The following example shows how to load an ARFF file incrementally using the `ArffLoader` class and train the `NaiveBayesUpdateable` classifier with one row at a time:

```
import weka.core.converters.ArffLoader;
import weka.classifiers.bayes.NaiveBayesUpdateable;
import java.io.File;
...
// load data
ArffLoader loader = new ArffLoader();
loader.setFile(new File("/some/where/data.arff"));
Instances structure = loader.getStructure();
structure.setClassIndex(structure.numAttributes() - 1);

// train NaiveBayes
NaiveBayesUpdateable nb = new NaiveBayesUpdateable();
nb.buildClassifier(structure);
Instance current;
while ((current = loader.getNextInstance(structure)) != null)
  nb.updateClassifier(current);
```

16.6.2 Evaluating a classifier

Building a classifier is only one part of the equation, evaluating how *well* it performs is another important part. WEKA supports two types of evaluation:

- *Cross-validation* – If one only has a single dataset and wants to get a reasonable realistic evaluation. Setting the number of folds equal to the number of rows in the dataset will give one leave-one-out cross-validation (LOOCV).
- *Dedicated test set* – The test set is solely used to evaluate the built classifier. It is important to have a test set that incorporates the same (or similar) concepts as the training set, otherwise one will always end up with poor performance.

The evaluation step, including collection of statistics, is performed by the `Evaluation` class (package `weka.classifiers`).

Cross-validation

The `crossValidateModel` method of the `Evaluation` class is used to perform cross-validation with an **untrained** classifier and a single dataset. Supplying an untrained classifier ensures that no information leaks into the actual evaluation. Even though it is an implementation requirement, that the `buildClassifier` method resets the classifier, it cannot be guaranteed that this is indeed the case ("leaky" implementation). Using an untrained classifier avoids unwanted side-effects, as for each train/test set pair, a copy of the originally supplied classifier is used.

Before cross-validation is performed, the data gets randomized using the supplied random number generator (`java.util.Random`). It is recommended that this number generator is "seeded" with a specified seed value. Otherwise, subsequent runs of cross-validation on the same dataset will not yield the same results, due to different randomization of the data (see section 16.4 for more information on randomization).

The code snippet below performs 10-fold cross-validation with a J48 decision tree algorithm on a dataset `newData`, with random number generator that is seeded with "1". The summary of the collected statistics is output to `stdout`.

```
import weka.classifiers.Evaluation;
import weka.classifiers.trees.J48;
import weka.core.Instances;
import java.util.Random;
...
Instances newData = ... // from somewhere
Evaluation eval = new Evaluation(newData);
J48 tree = new J48();
eval.crossValidateModel(tree, newData, 10, new Random(1));
System.out.println(eval.toSummaryString("\nResults\n\n", false));
```

The `Evaluation` object in this example is initialized with the dataset used in the evaluation process. This is done in order to inform the evaluation about the type of data that is being evaluated, ensuring that all internal data structures are setup correctly.

Train/test set

Using a dedicated test set to evaluate a classifier is just as easy as cross-validation. But instead of providing an untrained classifier, a trained classifier has to be provided now. Once again, the `weka.classifiers.Evaluation` class is used to perform the evaluation, this time using the `evaluateModel` method.

The code snippet below trains a J48 with default options on a training set and evaluates it on a test set before outputting the summary of the collected statistics:

```
import weka.core.Instances;
import weka.classifiers.Evaluation;
import weka.classifiers.trees.J48;
...
Instances train = ...   // from somewhere
Instances test = ...    // from somewhere
// train classifier
Classifier cls = new J48();
cls.buildClassifier(train);
// evaluate classifier and print some statistics
Evaluation eval = new Evaluation(train);
eval.evaluateModel(cls, test);
System.out.println(eval.toSummaryString("\nResults\n\n", false));
```

Statistics

In the previous sections, the `toSummaryString` of the `Evaluation` class was already used in the code examples. But there are other summary methods for nominal class attributes available as well:

- `toMatrixString` – outputs the confusion matrix.
- `toClassDetailsString` – outputs TP/FP rates, precision, recall, F-measure, AUC (per class).
- `toCumulativeMarginDistributionString` – outputs the cumulative margins distribution.

If one does not want to use these summary methods, it is possible to access the individual statistical measures directly. Below, a few common measures are listed:

- nominal class attribute
  - `correct()` – The number of correctly classified instances. The incorrectly classified ones are available through `incorrect()`.
  - `pctCorrect()` – The percentage of correctly classified instances (accuracy). `pctIncorrect()` returns the number of misclassified ones.
  - `areaUnderROC(int)` – The AUC for the specified class label index (0-based index).
- numeric class attribute
  - `correlationCoefficient()` – The correlation coefficient.
- general
  - `meanAbsoluteError()` – The mean absolute error.
  - `rootMeanSquaredError()` – The root mean squared error.
  - `numInstances()` – The number of instances with a class value.
  - `unclassified()` - The number of unclassified instances.
  - `pctUnclassified()` - The percentage of unclassified instances.

For a complete overview, see the Javadoc page of the `Evaluation` class. By looking up the source code of the summary methods mentioned above, one can easily determine what methods are used for which particular output.

16.6.3 Classifying instances

After a classifier setup has been evaluated and proven to be useful, a built classifier can be used to make predictions and label previously unlabeled data. Section 16.5.2 already provided a glimpse of how to use a classifier's `classifyInstance` method. This section here elaborates a bit more on this.

The following example uses a trained classifier `tree` to label all the instances in an unlabeled dataset that gets loaded from disk. After all the instances have been labeled, the newly labeled dataset gets written back to disk to a new file.

```
// load unlabeled data and set class attribute
Instances unlabeled = DataSource.read("/some/where/unlabeled.arff");
unlabeled.setClassIndex(unlabeled.numAttributes() - 1);
// create copy
Instances labeled = new Instances(unlabeled);
// label instances
for (int i = 0; i < unlabeled.numInstances(); i++) {
  double clsLabel = tree.classifyInstance(unlabeled.instance(i));
  labeled.instance(i).setClassValue(clsLabel);
}
// save newly labeled data
DataSink.write("/some/where/labeled.arff", labeled);
```

The above example works for classification and regression problems alike, as long as the classifier can handle numeric classes, of course. Why is that? The `classifyInstance(Instance)` method returns for numeric classes the regression value and for nominal classes the 0-based index in the list of available class labels.

If one is interested in the class distribution instead, then one can use the `distributionForInstance(Instance)` method (this array sums up to 1). Of course, using this method makes only sense for classification problems. The code snippet below outputs the class distribution, the actual and predicted label side-by-side in the console:

```
// load data
Instances train = DataSource.read(args[0]);
train.setClassIndex(train.numAttributes() - 1);
Instances test = DataSource.read(args[1]);
test.setClassIndex(test.numAttributes() - 1);
// train classifier
J48 cls = new J48();
cls.buildClassifier(train);
// output predictions
System.out.println("# - actual - predicted - distribution");
for (int i = 0; i < test.numInstances(); i++) {
  double pred = cls.classifyInstance(test.instance(i));
  double[] dist = cls.distributionForInstance(test.instance(i));
  System.out.print((i+1) + " - ");
  System.out.print(test.instance(i).toString(test.classIndex()) + " - ");
  System.out.print(test.classAttribute().value((int) pred) + " - ");
  System.out.println(Utils.arrayToString(dist));
}
```

16.7 Clustering

Clustering is an unsupervised Machine Learning technique of finding patterns in the data, i.e., these algorithms work without class attributes. Classifiers, on the other hand, are supervised and need a class attribute. This section, similar to the one about classifiers, covers the following topics:

- *Building a clusterer* – batch and incremental learning.
- *Evaluating a clusterer* – how to evaluate a built clusterer.
- *Clustering instances* – determining what clusters unknown instances belong to.

Fully functional example classes are located in the `wekaexamples.clusterers` package of the *Weka Examples* collection[3].

16.7.1 Building a clusterer

Clusterers, just like classifiers, are by design batch-trainable as well. They all can be built on data that is completely stored in memory. But a small subset of the cluster algorithms can also update the internal representation incrementally. The following two sections cover both types of clusterers.

Batch clusterers

Building a batch clusterer, just like a classifier, happens in two stages:

- *set options* – either calling the `setOptions(String[])` method or the appropriate `set`-methods of the properties.
- *build the model* with training data – calling the `buildClusterer(Instances)` method. By definition, subsequent calls of this method must result in the same model ("repeatable experiments"). In other words, calling this method must completely reset the model.

Below is an example of building the `EM` clusterer with a maximum of 100 iterations. The options are set using the `setOptions(String[])` method:

```
import weka.clusterers.EM;
import weka.core.Instances;
...
Instances data = ... // from somewhere
String[] options = new String[2];
options[0] = "-I";                 // max. iterations
options[1] = "100";
EM clusterer = new EM();   // new instance of clusterer
clusterer.setOptions(options);     // set the options
clusterer.buildClusterer(data);    // build the clusterer
```

Incremental clusterers

Incremental clusterers in WEKA implement the interface `UpdateableClusterer` (package `weka.clusterers`). Training an incremental clusterer happens in three stages, similar to incremental classifiers:

1. *initialize* the model by calling the `buildClusterer(Instances)` method. Once again, one can either use an empty `weka.core.Instances` object or one with an initial set of data.
2. *update* the model row-by-row by calling the the `updateClusterer(Instance)` method.
3. *finish* the training by calling `updateFinished()` method. In case cluster algorithms need to perform computational expensive post-processing or clean up operations.

An `ArffLoader` is used in the following example to build the `Cobweb` clusterer incrementally:

```
import weka.clusterers.Cobweb;
import weka.core.Instance;
import weka.core.Instances;
import weka.core.converters.ArffLoader;
...
// load data
ArffLoader loader = new ArffLoader();
loader.setFile(new File("/some/where/data.arff"));
Instances structure = loader.getStructure();
// train Cobweb
Cobweb cw = new Cobweb();
cw.buildClusterer(structure);
Instance current;
while ((current = loader.getNextInstance(structure)) != null)
  cw.updateClusterer(current);
cw.updateFinished();
```

16.7.2 Evaluating a clusterer

Evaluation of clusterers is not as comprehensive as the evaluation of classifiers. Since clustering is unsupervised, it is also a lot harder determining how *good* a model is. The class used for evaluating cluster algorithms, is `ClusterEvaluation` (package `weka.clusterers`).

In order to generate the same output as the Explorer or the command-line, one can use the `evaluateClusterer` method, as shown below:

```
import weka.clusterers.EM;
import weka.clusterers.ClusterEvaluation;
...
String[] options = new String[2];
options[0] = "-t";
options[1] = "/some/where/somefile.arff";
System.out.println(ClusterEvaluation.evaluateClusterer(new EM(), options));
```

Or, if the dataset is already present in memory, one can use the following approach:

```
import weka.clusterers.ClusterEvaluation;
import weka.clusterers.EM;
import weka.core.Instances;
...
Instances data = ... // from somewhere
EM cl = new EM();
cl.buildClusterer(data);
ClusterEvaluation eval = new ClusterEvaluation();
eval.setClusterer(cl);
eval.evaluateClusterer(new Instances(data));
System.out.println(eval.clusterResultsToString());
```

Density based clusterers, i.e., algorithms that implement the interface named `DensityBasedClusterer` (package `weka.clusterers`) can be cross-validated and the log-likelyhood obtained. Using the `MakeDensityBasedClusterer` meta-clusterer, any non-density based clusterer can be turned into such. Here is an example of cross-validating a density based clusterer and obtaining the log-likelyhood:

```
import weka.clusterers.ClusterEvaluation;
import weka.clusterers.DensityBasedClusterer;
import weka.core.Instances;
import java.util.Random;
...
Instances data = ...                            // from somewhere
DensityBasedClusterer clusterer = new ...       // the clusterer to evaluate
double logLikelyhood =
ClusterEvaluation.crossValidateModel(           // cross-validate
    clusterer, data, 10,                        // with 10 folds
    new Random(1));                             // and random number generator
                                                // with seed 1
```

Classes to clusters

Datasets for supervised algorithms, like classifiers, can be used to evaluate a clusterer as well. This evaluation is called *classes-to-clusters*, as the clusters are mapped back onto the classes.

This type of evaluation is performed as follows:

1. *create a copy* of the dataset containing the class attribute and remove the class attribute, using the `Remove` filter (this filter is located in package `weka.filters.unsupervised.attribute`).
2. *build the clusterer* with this new data.
3. *evaluate the clusterer* now with the **original** data.

And here are the steps translated into code, using `EM` as the clusterer being evaluated:

1. create a copy of data without class attribute

```
Instances data = ... // from somewhere
Remove filter = new Remove();
filter.setAttributeIndices("" + (data.classIndex() + 1));
filter.setInputFormat(data);
Instances dataClusterer = Filter.useFilter(data, filter);
```

2. build the clusterer

```
EM clusterer = new EM();
// set further options for EM, if necessary...
clusterer.buildClusterer(dataClusterer);
```

3. evaluate the clusterer

```
ClusterEvaluation eval = new ClusterEvaluation();
eval.setClusterer(clusterer);
eval.evaluateClusterer(data);
// print results
System.out.println(eval.clusterResultsToString());
```

16.7.3 Clustering instances

Clustering of instances is very similar to classifying unknown instances when using classifiers. The following methods are involved:

- `clusterInstance(Instance)` – determines the cluster the `Instance` would belong to.
- `distributionForInstance(Instance)` – predicts the cluster membership for this `Instance`. The sum of this array adds up to 1.

The code fragment outlined below trains an `EM` clusterer on one dataset and outputs for a second dataset the predicted clusters and cluster memberships of the individual instances:

```
import weka.clusterers.EM;
import weka.core.Instances;
...
Instances dataset1 = ... // from somewhere
Instances dataset2 = ... // from somewhere
// build clusterer
EM clusterer = new EM();
clusterer.buildClusterer(dataset1);
// output predictions
System.out.println("# - cluster - distribution");
for (int i = 0; i < dataset2.numInstances(); i++) {
  int cluster = clusterer.clusterInstance(dataset2.instance(i));
  double[] dist = clusterer.distributionForInstance(dataset2.instance(i));
  System.out.print((i+1));
  System.out.print(" - ");
  System.out.print(cluster);
  System.out.print(" - ");
  System.out.print(Utils.arrayToString(dist));
  System.out.println();
}
```

16.8 Selecting attributes

Preparing one's data properly is a very important step for getting the best results. Reducing the number of attributes can not only help speeding up runtime with algorithms (some algorithms' runtime are quadratic in regards to number of attributes), but also help avoid "burying" the algorithm in a mass of attributes, when only a few are essential for building a good model.

There are three different types of evaluators in WEKA at the moment:

- *single attribute evaluators* – perform evaluations on single attributes. These classes implement the `weka.attributeSelection.AttributeEvaluator` interface. The `Ranker` search algorithm is usually used in conjunction with these algorithms.
- *attribute subset evaluators* – work on subsets of all the attributes in the dataset. The `weka.attributeSelection.SubsetEvaluator` interface is implemented by these evaluators.
- *attribute set evaluators* – evaluate sets of attributes. Not to be confused with the *subset evaluators*, as these classes are derived from the `weka.attributeSelection.AttributeSetEvaluator` superclass.

Most of the attribute selection schemes currently implemented are supervised, i.e., they require a dataset with a class attribute. Unsupervised evaluation algorithms are derived from one of the following superclasses:

- `weka.attributeSelection.UnsupervisedAttributeEvaluator`
  e.g., `LatentSemanticAnalysis`, `PrincipalComponents`
- `weka.attributeSelection.UnsupervisedSubsetEvaluator`
  none at the moment

Attribute selection offers *filtering on-the-fly*, like classifiers and clusterers, as well:

- `weka.attributeSelection.FilteredAttributeEval` – filter for evaluators that evaluate attributes individually.
- `weka.attributeSelection.FilteredSubsetEval` – for filtering evaluators that evaluate subsets of attributes.

So much about the differences among the various attribute selection algorithms and back to how to actually perform attribute selection. WEKA offers three different approaches:

- *Using a meta-classifier* – for performing attribute selection on-the-fly (similar to FilteredClassifier's filtering on-the-fly).
- *Using a filter* - for preprocessing the data.
- *Low-level API usage* - instead of using the meta-schemes (classifier or filter), one can use the attribute selection API directly as well.

The following sections cover each of the topics, accompanied with a code example. For clarity, the same evaluator and search algorithm is used in all of these examples.

Feel free to check out the example classes of the *Weka Examples* collection[3], located in the `wekaexamples.attributeSelection` package.

16.8.1 Using the meta-classifier

The meta-classifier `AttributeSelectedClassifier` (this classifier is located in package `weka.classifiers.meta`), is similar to the `FilteredClassifier`. But instead of taking a base-classifier and a filter as parameters to perform the filtering, the `AttributeSelectedClassifier` uses a *search* algorithm (derived from `weka.attributeSelection.ASEvaluation`), an *evaluator* (superclass is `weka.attributeSelection.ASSearch`) to perform the attribute selection and a base-classifier to train on the reduced data.

This example here uses `J48` as base-classifier, `CfsSubsetEval` as evaluator and a backwards operating `GreedyStepwise` as search method:

```
import weka.attributeSelection.CfsSubsetEval;
import weka.attributeSelection.GreedyStepwise;
import weka.classifiers.Evaluation;
import weka.classifiers.meta.AttributeSelectedClassifier;
import weka.classifiers.trees.J48;
import weka.core.Instances;
...
Instances data = ... // from somewhere
// setup meta-classifier
AttributeSelectedClassifier classifier = new AttributeSelectedClassifier();
CfsSubsetEval eval = new CfsSubsetEval();
GreedyStepwise search = new GreedyStepwise();
search.setSearchBackwards(true);
J48 base = new J48();
classifier.setClassifier(base);
classifier.setEvaluator(eval);
classifier.setSearch(search);
// cross-validate classifier
Evaluation evaluation = new Evaluation(data);
evaluation.crossValidateModel(classifier, data, 10, new Random(1));
System.out.println(evaluation.toSummaryString());
```

16.8.2 Using the filter

In case the data only needs to be reduced in dimensionality, but not used for training a classifier, then the filter approach is the right one. The `AttributeSelection` filter (package `weka.filters.supervised.attribute`) takes an evaluator and a search algorithm as parameter.

The code snippet below uses once again `CfsSubsetEval` as evaluator and a backwards operating `GreedyStepwise` as search algorithm. It just outputs the reduced data to `stdout` after the filtering step:

```
import weka.attributeSelection.CfsSubsetEval;
import weka.attributeSelection.GreedyStepwise;
import weka.core.Instances;
import weka.filters.Filter;
import weka.filters.supervised.attribute.AttributeSelection;
...
Instances data = ... // from somewhere
// setup filter
AttributeSelection filter = new AttributeSelection();
CfsSubsetEval eval = new CfsSubsetEval();
GreedyStepwise search = new GreedyStepwise();
search.setSearchBackwards(true);
filter.setEvaluator(eval);
filter.setSearch(search);
filter.setInputFormat(data);
// filter data
Instances newData = Filter.useFilter(data, filter);
System.out.println(newData);
```

16.8.3 Using the API directly

Using the meta-classifier or the filter approach makes attribute selection fairly easy. But it might not satify everybody's needs. For instance, if one wants to obtain the ordering of the attributes (using `Ranker`) or retrieve the indices of the selected attributes instead of the reduced data.

Just like the other examples, the one shown here uses the `CfsSubsetEval` evaluator and the `GreedyStepwise` search algorithm (in backwards mode). But instead of outputting the reduced data, only the selected indices are printed in the console:

```
import weka.attributeSelection.AttributeSelection;
import weka.attributeSelection.CfsSubsetEval;
import weka.attributeSelection.GreedyStepwise;
import weka.core.Instances;
...
Instances data = ... // from somewhere
// setup attribute selection
AttributeSelection attsel = new AttributeSelection();
CfsSubsetEval eval = new CfsSubsetEval();
GreedyStepwise search = new GreedyStepwise();
search.setSearchBackwards(true);
attsel.setEvaluator(eval);
attsel.setSearch(search);
// perform attribute selection
attsel.SelectAttributes(data);
int[] indices = attsel.selectedAttributes();
System.out.println(
     "selected attribute indices (starting with 0):\n"
     + Utils.arrayToString(indices));
```

16.9 Saving data

Saving `weka.core.Instances` objects is as easy as reading the data in the first place, though the process of storing the data again is far less common than of reading the data into memory. The following two sections cover how to save the data in files and in databases.

Just like with loading the data in chapter 16.2, examples classes for saving data can be found in the `wekaexamples.core.converters` package of the *Weka Examples* collection[3];

16.9.1 Saving data to files

Once again, one can either let WEKA choose the appropriate converter for saving the data or use an explicit converter (all savers are located in the `weka.core.converters` package). The latter approach is necessary, if the file name under which the data will be stored does not have an extension that WEKA recognizes.

Use the `DataSink` class (inner class of `weka.core.converters.ConverterUtils`), if the extensions are not a problem. Here are a few examples:

```
import weka.core.Instances;
import weka.core.converters.ConverterUtils.DataSink;
...
// data structure to save
Instances data = ...
// save as ARFF
DataSink.write("/some/where/data.arff", data);
// save as CSV
DataSink.write("/some/where/data.csv", data);
```

And here is an example of using the `CSVSaver` converter explicitly:

```
import weka.core.Instances;
import weka.core.converters.CSVSaver;
import java.io.File;
...
// data structure to save
Instances data = ...
// save as CSV
CSVSaver saver = new CSVSaver();
saver.setInstances(data);
saver.setFile(new File("/some/where/data.csv"));
saver.writeBatch();
```

16.9.2 Saving data to databases

Apart from the KnowledgeFlow, saving to databases is not very obvious in WEKA, unless one knows about the `DatabaseSaver` converter. Just like the `DatabaseLoader`, the saver counterpart can store the data either in batch mode or incrementally as well.

The first example shows how to save the data in batch mode, which is the easier way of doing it:

```
import weka.core.Instances;
import weka.core.converters.DatabaseSaver;
...
// data structure to save
Instances data = ...
// store data in database
DatabaseSaver saver = new DatabaseSaver();
saver.setDestination("jdbc_url", "the_user", "the_password");
// we explicitly specify the table name here:
saver.setTableName("whatsoever2");
saver.setRelationForTableName(false);
// or we could just update the name of the dataset:
// saver.setRelationForTableName(true);
// data.setRelationName("whatsoever2");
saver.setInstances(data);
saver.writeBatch();
```

Saving the data incrementally, requires a bit more work, as one has to specify that writing the data is done incrementally (using the `setRetrieval` method), as well as notifying the saver when all the data has been saved:

```
import weka.core.Instances;
import weka.core.converters.DatabaseSaver;
...
// data structure to save
Instances data = ...
// store data in database
DatabaseSaver saver = new DatabaseSaver();
saver.setDestination("jdbc_url", "the_user", "the_password");
// we explicitly specify the table name here:
saver.setTableName("whatsoever2");
saver.setRelationForTableName(false);
// or we could just update the name of the dataset:
// saver.setRelationForTableName(true);
// data.setRelationName("whatsoever2");
saver.setRetrieval(DatabaseSaver.INCREMENTAL);
saver.setStructure(data);
count = 0;
for (int i = 0; i < data.numInstances(); i++) {
  saver.writeIncremental(data.instance(i));
}
// notify saver that we're finished
saver.writeIncremental(null);
```

16.10 Visualization

The concepts covered in this chapter are also available through the example classes of the *Weka Examples* collection[3]. See the following packages:

- `wekaexamples.gui.graphvisualizer`
- `wekaexamples.gui.treevisualizer`
- `wekaexamples.gui.visualize`

16.10.1 ROC curves

WEKA can generate "Receiver operating characteristic" (ROC) curves, based on the collected predictions during an evaluation of a classifier. In order to display a ROC curve, one needs to perform the following steps:

1. Generate the plotable data based on the `Evaluation`'s collected predictions, using the `ThresholdCurve` class (package `weka.classifiers.evaluation`).
2. Put the plotable data into a plot container, an instance of the `PlotData2D` class (package `weka.gui.visualize`).
3. Add the plot container to a visualization panel for displaying the data, an instance of the `ThresholdVisualizePanel` class (package `weka.gui.visualize`).
4. Add the visualization panel to a `JFrame` (package `javax.swing`) and display it.

And now, the four steps translated into actual code:

1. Generate the plotable data

   ```
   Evaluation eval = ... // from somewhere
   ThresholdCurve tc = new ThresholdCurve();
   int classIndex = 0;  // ROC for the 1st class label
   Instances curve = tc.getCurve(eval.predictions(), classIndex);
   ```

2. Put the plotable into a plot container

   ```
   PlotData2D plotdata = new PlotData2D(curve);
   plotdata.setPlotName(curve.relationName());
   plotdata.addInstanceNumberAttribute();
   ```

3. Add the plot container to a visualization panel

   ```
   ThresholdVisualizePanel tvp = new ThresholdVisualizePanel();
   tvp.setROCString("(Area under ROC = " +
     Utils.doubleToString(ThresholdCurve.getROCArea(curve),4)+")");
   tvp.setName(curve.relationName());
   tvp.addPlot(plotdata);
   ```

4. Add the visualization panel to a `JFrame`

   ```
   final JFrame jf = new JFrame("WEKA ROC: " + tvp.getName());
   jf.setSize(500,400);
   jf.getContentPane().setLayout(new BorderLayout());
   jf.getContentPane().add(tvp, BorderLayout.CENTER);
   jf.setDefaultCloseOperation(JFrame.DISPOSE_ON_CLOSE);
   jf.setVisible(true);
   ```

16.10.2 Graphs

Classes implementing the `weka.core.Drawable` interface can generate graphs of their internal models which can be displayed. There are two different types of graphs available at the moment, which are explained in the subsequent sections:

- Tree – decision trees.
- BayesNet – bayesian net graph structures.

16.10.2.1 Tree

It is quite easy to display the internal tree structure of classifiers like J48 or M5P (package `weka.classifiers.trees`). The following example builds a J48 classifier on a dataset and displays the generated tree visually using the `TreeVisualizer` class (package `weka.gui.treevisualizer`). This visualization class can be used to view trees (or digraphs) in GraphViz's DOT language[26].

```
import weka.classifiers.trees.J48;
import weka.core.Instances;
import weka.gui.treevisualizer.PlaceNode2;
import weka.gui.treevisualizer.TreeVisualizer;
import java.awt.BorderLayout;
import javax.swing.JFrame;
...
Instances data = ... // from somewhere
// train classifier
J48 cls = new J48();
cls.buildClassifier(data);
// display tree
TreeVisualizer tv = new TreeVisualizer(
    null, cls.graph(), new PlaceNode2());
JFrame jf = new JFrame("Weka Classifier Tree Visualizer: J48");
jf.setDefaultCloseOperation(JFrame.DISPOSE_ON_CLOSE);
jf.setSize(800, 600);
jf.getContentPane().setLayout(new BorderLayout());
jf.getContentPane().add(tv, BorderLayout.CENTER);
jf.setVisible(true);
// adjust tree
tv.fitToScreen();
```

16.10.2.2 BayesNet

The graphs that the BayesNet classifier (package `weka.classifiers.bayes`) generates can be displayed using the `GraphVisualizer` class (located in package `weka.gui.graphvisualizer`). The `GraphVisualizer` can display graphs that are either in GraphViz's DOT language[26] or in XML BIF[20] format. For displaying DOT format, one needs to use the method `readDOT`, and for the BIF format the method `readBIF`.

The following code snippet trains a `BayesNet` classifier on some data and then displays the graph generated from this data in a frame:

```
import weka.classifiers.bayes.BayesNet;
import weka.core.Instances;
import weka.gui.graphvisualizer.GraphVisualizer;
import java.awt.BorderLayout;
import javax.swing.JFrame;
...
Instances data = ... // from somewhere
// train classifier
BayesNet cls = new BayesNet();
cls.buildClassifier(data);
// display graph
GraphVisualizer gv = new GraphVisualizer();
gv.readBIF(cls.graph());
JFrame jf = new JFrame("BayesNet graph");
jf.setDefaultCloseOperation(JFrame.DISPOSE_ON_CLOSE);
jf.setSize(800, 600);
jf.getContentPane().setLayout(new BorderLayout());
jf.getContentPane().add(gv, BorderLayout.CENTER);
jf.setVisible(true);
// layout graph
gv.layoutGraph();
```

16.11 Serialization

Serialization[2] is the process of saving an object in a persistent form, e.g., on the harddisk as a bytestream. *Deserialization* is the process in the opposite direction, creating an object from a persistently saved data structure. In Java, an object can be serialized if it imports the `java.io.Serializable` interface. Members of an object that are not supposed to be serialized, need to be declared with the keyword `transient`.

In the following are some Java code snippets for serializing and deserializing a `J48` classifier. Of course, serialization is not limited to classifiers. Most schemes in WEKA, like clusterers and filters, are also serializable.

Serializing a classifier

The `weka.core.SerializationHelper` class makes it easy to serialize an object. For saving, one can use one of the `write` methods:

```
import weka.classifiers.Classifier;
import weka.classifiers.trees.J48;
import weka.core.converters.ConverterUtils.DataSource;
import weka.core.SerializationHelper;
...
// load data
Instances inst = DataSource.read("/some/where/data.arff");
inst.setClassIndex(inst.numAttributes() - 1);
// train J48
Classifier cls = new J48();
cls.buildClassifier(inst);
// serialize model
SerializationHelper.write("/some/where/j48.model", cls);
```

Deserializing a classifier

Deserializing an object can be achieved by using one of the `read` methods:

```
import weka.classifiers.Classifier;
import weka.core.SerializationHelper;
...
// deserialize model
Classifier cls = (Classifier) SerializationHelper.read(
    "/some/where/j48.model");
```

[2] http://en.wikipedia.org/wiki/Serialization

Deserializing a classifier saved from the Explorer

The Explorer does not only save the built classifier in the model file, but also the header information of the dataset the classifier was built with. By storing the dataset information as well, one can easily check whether a serialized classifier can be applied on the current dataset. The `readAll` methods returns an array with all objects that are contained in the model file.

```
import weka.classifiers.Classifier;
import weka.core.Instances;
import weka.core.SerializationHelper;
...
// the current data to use with classifier
Instances current = ... // from somewhere
// deserialize model
Object o[] = SerializationHelper.readAll("/some/where/j48.model");
Classifier cls = (Classifier) o[0];
Instances data = (Instances) o[1];
// is the data compatible?
if (!data.equalHeaders(current))
   throw new Exception("Incompatible data!");
```

Serializing a classifier for the Explorer

If one wants to serialize the dataset header information alongside the classifier, just like the Explorer does, then one can use one of the `writeAll` methods:

```
import weka.classifiers.Classifier;
import weka.classifiers.trees.J48;
import weka.core.converters.ConverterUtils.DataSource;
import weka.core.SerializationHelper;
...
// load data
Instances inst = DataSource.read("/some/where/data.arff");
inst.setClassIndex(inst.numAttributes() - 1);
// train J48
Classifier cls = new J48();
cls.buildClassifier(inst);
// serialize classifier and header information
Instances header = new Instances(inst, 0);
SerializationHelper.writeAll(
    "/some/where/j48.model", new Object[]{cls, header});
```

Chapter 17

Extending WEKA

For most users, the existing WEKA framework will be sufficient to perform the task at hand, offering a wide range of filters, classifiers, clusterers, etc. Researchers, on the other hand, might want to add new algorithms and compare them against existing ones. The framework with its existing algorithms is not set in stone, but basically one big plugin framework. With WEKA's automatic discovery of classes on the classpath, adding new classifiers, filters, etc. to the existing framework is very easy.

Though algorithms like clusterers, associators, data generators and attribute selection are not covered in this chapter, their implemention is very similar to the one of implementing a classifier. You basically choose a superclass to derive your new algorithm from and then implement additional interfaces, if necessary. Just check out the other algorithms that are already implemented.

The section covering the GenericObjectEditor (see chapter 18.4) shows you how to tell WEKA where to find your class(es) and therefore making it/them available in the GUI (Explorer/Experimenter) via the GenericObjectEditor.

17.1 Writing a new Classifier

17.1.1 Choosing the base class

The ancestor of all classifiers in WEKA is `weka.classifiers.Classifier`, an abstract class. Your new classifier must be derived from this class at least to be visible through the GenericObjectEditor. But in order to make implementations of new classifiers even easier, WEKA comes already with a range of other abstract classes derived from `weka.classifiers.Classifier`. In the following you will find an overview that will help you decide what base class to use for your classifier. For better readability, the `weka.classifiers` prefix was dropped from the class names:

- simple classifier
  - `Classifier` – not randomizable
  - `RandomizableClassifier` – randomizable
- meta classifier
  - single base classifier
    - `SingleClassifierEnhancer` – not randomizable, not iterated
    - `RandomizableSingleClassifierEnhancer` – randomizable, not iterated
    - `IteratedSingleClassifierEnhancer` – not randomizable, iterated
    - `RandomizableIteratedSingleClassifierEnhancer` – randomizable, iterated
  - multiple base classifiers
    - `MultipleClassifiersCombiner` – not randomizable
    - `RandomizableMultipleClassifiersCombiner` – randomizable

If you are still unsure about what superclass to choose, then check out the Javadoc of those superclasses. In the Javadoc you will find all the classifiers that are derived from it, which should give you a better idea whether this particular superclass is suited for your needs.

17.1.2 Additional interfaces

The abstract classes listed above basically just implement various combinations of the following two interfaces:

- `weka.core.Randomizable` – to allow (seeded) randomization taking place
- `weka.classifiers.IterativeClassifier` – to make the classifier an iterated one

But these interfaces are not the only ones that can be implemented by a classifier. Here is a list for further interfaces:

- `weka.core.AdditionalMeasureProducer` – the classifier returns additional information, e.g., `J48` returns the tree size with this method.
- `weka.core.WeightedInstancesHandler` – denotes that the classifier can make use of weighted `Instance` objects (the default weight of an `Instance` is 1.0).
- `weka.core.TechnicalInformationHandler` – for returning paper references and publications this classifier is based on.
- `weka.classifiers.Sourcable` – classifiers implementing this interface can return Java code of a built model, which can be used elsewhere.
- `weka.classifiers.UpdateableClassifier` – for classifiers that can be trained incrementally, i.e., row by row like `NaiveBayesUpdateable`.

17.1.3 Packages

A few comments about the different sub-packages in the `weka.classifiers` package:

- `bayes` – contains bayesian classifiers, e.g., `NaiveBayes`
- `evaluation` – classes related to evaluation, e.g., confusion matrix, threshold curve (= ROC)
- `functions` – e.g., Support Vector Machines, regression algorithms, neural nets
- `lazy` – "learning" is performed at prediction time, e.g., k-nearest neighbor (k-NN)
- `meta` – meta-classifiers that use a base one or more classifiers as input, e.g., boosting, bagging or stacking
- `mi` – classifiers that handle multi-instance data
- `misc` – various classifiers that don't fit in any another category
- `rules` – rule-based classifiers, e.g., ZeroR
- `trees` – tree classifiers, like decision trees with `J48` a very common one

17.1.4 Implementation

In the following you will find information on what methods need to be implemented and other coding guidelines for methods, option handling and documentation of the source code.

17.1.4.1 Methods

This section explains what methods need to be implemented in general and more specialized ones in case of meta-classifiers (either with single or multiple base-classifiers).

General
Here is an overview of methods that your new classifier needs to implement in order to integrate nicely into the WEKA framework:

globalInfo()
returns a short description that is displayed in the GUI, like the Explorer or Experimenter. How long this description will be is really up to you, but it should be sufficient to understand the classifier's underlying algorithm. If the classifier implements the `weka.core.TechnicalInformationHandler` interface then you could refer to the publication(s) by extending the returned string by `getTechnicalInformation().toString()`.

listOptions()
returns a `java.util.Enumeration` of `weka.core.Option` objects. This enumeration is used to display the help on the command-line, hence it needs to return the `Option` objects of the superclass as well.

setOptions(String[])
parses the options that the classifier would receive from a command-line invocation. A parameter and argument are always two elements in the string array. A common mistake is to use a single cell in the string array for both of them, e.g., `"-S 1"` instead of `"-S","1"`. You can use the methods `getOption` and `getFlag` of the `weka.core.Utils` class to retrieve the values of an option or to ascertain whether a flag is present. But note that these calls **remove** the option and, if applicable, the argument from the string array ("destructive"). The last call in the `setOptions` methods should always be the `super.setOptions(String[])` one, in order to pass on any other arguments still present in the array to the superclass. The following code snippet just parses the only option "alpha" that an imaginary classifier defines:

```
import weka.core.Utils;
...
public void setOptions(String[] options) throws Exception {
  String tmpStr = Utils.getOption("alpha", options);
  if (tmpStr.length() == 0) {
    setAlpha(0.75);
  }
  else {
    setAlpha(Double.parseDouble(tmpStr));
  }
  super.setOptions(options);
}
```

getOptions()
returns a string array of command-line options that resemble the current classifier setup. Supplying this array to the `setOptions(String[])` method must result in the same configuration. This method will get called in the GUI when copying a classifier setup to the clipboard. Since handling of arrays is a bit cumbersome in Java (due to fixed length), using an instance of `java.util.Vector` is a lot easier for creating the array that needs to be returned. The following code snippet just adds the only option "alpha" that the classifier defines to the array that is being returned, including the options of the superclass:

```
import java.util.Arrays;
import java.util.Vector;
...
public String[] getOptions() {
  Vector<String> result = new Vector<String>();
  result.add("-alpha");
  result.add("" + getAlpha());
  result.addAll(Arrays.asList(super.getOptions())); // superclass
  return result.toArray(new String[result.size()]);
}
```

Note, that the `getOptions()` method requires you to add the preceding dash for an option, opposed to the `getOption`/`getFlag` calls in the `setOptions` method.

getCapabilities()
returns meta-information on what type of data the classifier can handle, in regards to attributes and class attributes. See section "Capabilities" on page 242 for more information.

buildClassifier(Instances)
builds the model from scratch with the provided dataset. Each subsequent call of this method **must** result in the same model being built. The `buildClassifier` method also tests whether the supplied data can be handled at all by the classifier, utilizing the capabilities returned by the `getCapabilities()` method:

```
public void buildClassifier(Instances data) throws Exception {
  // test data against capabilities
  getCapabilities().testWithFail(data);
  // remove instances with missing class value,
  // but don't modify original data
  data = new Instances(data);
  data.deleteWithMissingClass();
  // actual model generation
  ...
}
```

toString()
is used for outputting the built model. This is not required, but it is useful for the user to see properties of the model. Decision trees normally ouput the tree, support vector machines the support vectors and rule-based classifiers the generated rules.

distributionForInstance(Instance)
returns the class probabilities array of the prediction for the given `weka.core.Instance` object. If your classifier handles *nominal* class attributes, then you need to override this method.

classifyInstance(Instance)
returns the classification or regression for the given `weka.core.Instance` object. In case of a *nominal* class attribute, this method returns the index of the class label that got predicted. You do not need to override this method in this case as the `weka.classifiers.Classifier` superclass already determines the class label index based on the probabilities array that the `distributionForInstance(Instance)` method returns (it returns the index in the array with the highest probability; in case of ties the first one). For *numeric* class attributes, you need to override this method, as it has to return the regression value predicted by the model.

main(String[])
executes the classifier from command-line. If your new algorithm is called `FunkyClassifier`, then use the following code as your `main` method:

```
/**
 * Main method for executing this classifier.
 *
 * @param args the options, use "-h" to display options
 */
public static void main(String[] args) {
  runClassifier(new FunkyClassifier(), args);
}
```

Note: the static method `runClassifier` (defined in the abstract superclass `weka.classifiers.Classifier`) handles all the appropriate calls and catches and processes any exceptions as well.

Meta-classifiers

Meta-classifiers define a range of other methods that you might want to override. Normally, this should not be the case. But if your classifier requires the base-classifier(s) to be of a certain type, you can override the specific set-method and add additional checks.

SingleClassifierEnhancer
The following methods are used for handling the single base-classifier of this meta-classifier.

defaultClassifierString()
returns the class name of the classifier that is used as the default one for this meta-classifier.

setClassifier(Classifier)
sets the classifier object. Override this method if you require further checks, like that the classifiers needs to be of a certain class. This is necessary, if you still want to allow the user to parametrize the base-classifier, but not choose another classifier with the GenericObjectEditor. Be aware that this method does not create a copy of the provided classifier.

getClassifier()
returns the currently set classifier object. Note, this method returns the internal object and not a copy.

MultipleClassifiersCombiner
This meta-classifier handles its multiple base-classifiers with the following methods:

setClassifiers(Classifier[])
sets the array of classifiers to use as base-classifiers. If you require the base-classifiers to implement a certain interface or be of a certain class, then override this method and add the necessary checks. Note, this method does not create a copy of the array, but just uses this reference internally.

getClassifiers()
returns the array of classifiers that is in use. Careful, this method returns the internal array and not a copy of it.

getClassifier(int)
returns the classifier from the internal classifier array specified by the given index. Once again, this method does not return a copy of the classifier, but the actual object used by this classifier.

17.1.4.2 Guidelines

WEKA's code base requires you to follow a few rules. The following sections can be used as guidelines in writing your code.

Parameters
There are two different ways of setting/obtaining parameters of an algorithm. Both of them are unfortunately completely independent, which makes option handling so prone to errors. Here are the two:

1. command-line options, using the setOptions/getOptions methods
2. using the properties through the GenericObjectEditor in the GUI

Each command-line option must have a corresponding GUI property and vice versa. In case of GUI properties, the get- and set-method for a property must comply with Java Beans style in order to show up in the GUI. You need to supply three methods for each property:

- public void set*<PropertyName>*(*<Type>*) – checks whether the supplied value is valid and only then updates the corresponding member variable. In any other case it should ignore the value and output a warning in the console or throw an IllegalArgumentException.
- public *<Type>* get*<PropertyName>*() – performs any necessary conversions of the internal value and returns it.
- public String *<propertyName>*TipText() – returns the help text that is available through the GUI. Should be the same as on the command-line. Note: everything after the first period "." gets truncated from the tool tip that pops up in the GUI when hovering with the mouse cursor over the field in the GenericObjectEditor.

With a property called "alpha" of type "double", we get the following method signatures:

- public void setAlpha(double)
- public double getAlpha()
- public String alphaTipText()

These get- and set-methods should be used in the getOptions and setOptions methods as well, to impose the same checks when getting/setting parameters.

Randomization
In order to get repeatable experiments, one is not allowed to use unseeded random number generators like Math.random(). Instead, one has to instantiate a java.util.Random object in the buildClassifier(Instances) method with a specific seed value. The seed value can be user supplied, of course, which all the Randomizable... abstract classifiers already implement.

Capabilities

By default, the `weka.classifiers.Classifier` superclass returns an object that denotes that the classifier can handle **any** type of data. This is useful for rapid prototyping of new algorithms, but also very dangerous. If you do not specifically define what type of data can be handled by your classifier, you can end up with meaningless models or errors. This can happen if you devise a new classifier which is supposed to handle only numeric attributes. By using the `value(int/Attribute)` method of a `weka.core.Instance` to obtain the numeric value of an attribute, you also obtain the internal format of nominal, string and relational attributes. Of course, treating these attribute types as numeric ones does not make any sense. Hence it is highly recommended (and required for contributions) to override this method in your own classifier.

There are three different types of capabilities that you can define:

1. *attribute related* – e.g., nominal, numeric, date, missing values, ...
2. *class attribute related* – e.g., no-class, nominal, numeric, missing class values, ...
3. *miscellaneous* – e.g., only multi-instance data, minimum number of instances in the training data

There are some special cases:

- *incremental classifiers* – need to set the minimum number of instances in the training data to 0, since the default is 1:
  `setMinimumNumberInstances(0)`
- *multi-instance classifiers* – in order to signal that the special multi-instance format (*bag-id, bag-data, class*) is used, they need to enable the following capability:
  `enable(Capability.ONLY_MULTIINSTANCE)`
  These classifiers also need to implement the interface specific to multi-instance, `weka.core.MultiInstanceCapabilitiesHandler`, which returns the capabilities for the *bag-data*.
- *cluster algorithms* – since clusterers are unsupervised algorithms, they cannot process data with the class attribute set. The capability that denotes that an algorithm can handle data without a class attribute is `Capability.NO_CLASS`

And a note on enabling/disabling *nominal attributes* or *nominal class attributes*. These operations automatically enable/disable the *binary*, *unary* and *empty nominal* capabilities as well. The following sections list a few examples of how to configure the capabilities.

Simple classifier

A classifier that handles only numeric classes and numeric and nominal attributes, but no missing values at all, would configure the `Capabilities` object like this:

```
public Capabilities getCapabilities() {
  Capabilities result = new Capabilities(this);
  // attributes
  result.enable(Capability.NOMINAL_ATTRIBUTES);
  result.enable(Capability.NUMERIC_ATTRIBUTES);
  // class
  result.enable(Capability.NUMERIC_CLASS);
  return result;
}
```

Another classifier, that only handles binary classes and only nominal attributes and missing values, would implement the `getCapabilities()` method as follows:

```
public Capabilities getCapabilities() {
  Capabilities result = new Capabilities(this);
  // attributes
  result.enable(Capability.NOMINAL_ATTRIBUTES);
  result.enable(Capability.MISSING_VALUES);
  // class
  result.enable(Capability.BINARY_CLASS);
  result.disable(Capability.UNNARY_CLASS);
  result.enable(Capability.MISSING_CLASS_VALUES);
  return result;
}
```

Meta-classifier

Meta-classifiers, by default, just return the capabilities of their base classifiers - in case of descendants of the `weka.classifier.MultipleClassifiersCombiner`, an **AND** over all the `Capabilities` of the base classifiers is returned.

Due to this behavior, the capabilities depend – normally – only on the currently configured base classifier(s). To *soften* filtering for certain behavior, meta-classifiers also define so-called *Dependencies* on a per-Capability basis. These dependencies tell the filter that even though a certain capability is not supported right now, it is possible that it will be supported with a different base classifier. By default, all capabilities are initialized as *Dependencies*.

`weka.classifiers.meta.LogitBoost`, e.g., is restricted to nominal classes. For that reason it disables the *Dependencies* for the class:

```
result.disableAllClasses();               // disable all class types
result.disableAllClassDependencies();     // no dependencies!
result.enable(Capability.NOMINAL_CLASS); // only nominal classes allowed
```

Javadoc

In order to keep code-quality high and maintenance low, source code needs to be well documented. This includes the following Javadoc requirements:

- **class**
  - description of the classifier
  - listing of command-line parameters
  - publication(s), if applicable
  - `@author` and `@version` tag
- **methods** (all, not just public)
  - each *parameter* is documented
  - *return* value, if applicable, is documented
  - *exception(s)* are documented
  - the `setOptions(String[])` method also lists the command-line parameters

Most of the *class*-related and the `setOptions` Javadoc is already available through the source code:

- description of the classifier – `globalInfo()`
- listing of command-line parameters – `listOptions()`
- publication(s), if applicable – `getTechnicalInformation()`

In order to avoid manual syncing between Javadoc and source code, WEKA comes with some tools for updating the Javadoc automatically. The following tools take a concrete class and update its source code (the source code directory needs to be supplied as well, of course):

- `weka.core.AllJavadoc` – executes all Javadoc-producing classes (this is the tool, you would normally use)
- `weka.core.GlobalInfoJavadoc` – updates the *globalinfo* tags
- `weka.core.OptionHandlerJavadoc` – updates the *option* tags
- `weka.core.TechnicalInformationHandlerJavadoc` – updates the *technical* tags (plain text and BibTeX)

These tools look for specific comment tags in the source code and replace everything in between the start and end tag with the documentation obtained from the actual class.

- description of the classifier

  ```
  <!-- globalinfo-start -->
  will be automatically replaced
  <!-- globalinfo-end -->
  ```

- listing of command-line parameters

  ```
  <!-- options-start -->
  will be automatically replaced
  <!-- options-end -->
  ```

- publication(s), if applicable

  ```
  <!-- technical-bibtex-start -->
  will be automatically replaced
  <!-- technical-bibtex-end -->
  ```

  for a shortened, plain-text version use the following:

  ```
  <!-- technical-plaintext-start -->
  will be automatically replaced
  <!-- technical-plaintext-end -->
  ```

Here is a template of a Javadoc class block for an imaginary classifier that also implements the `weka.core.TechnicalInformationHandler` interface:

```
/**
 <!-- globalinfo-start -->
 <!-- globalinfo-end -->
 *
 <!-- technical-bibtex-start -->
 <!-- technical-bibtex-end -->
 *
 <!-- options-start -->
 <!-- options-end -->
 *
 * @author John Doe (john dot doe at no dot where dot com)
 * @version $Revision: 6192 $
 */
```

The template for any classifier's `setOptions(String[])` method is as follows:

```
  /**
   * Parses a given list of options.
   *
   <!-- options-start -->
   <!-- options-end -->
   *
   * @param options the list of options as an array of strings
   * @throws Exception if an option is not supported
   */
```

Running the `weka.core.AllJavadoc` tool over this code will output code with the comments filled out accordingly.

Revisions

Classifiers implement the `weka.core.RevisionHandler` interface. This provides the functionality of obtaining the Subversion revision from within Java. Classifiers that are not part of the official WEKA distribution do not have to implement the method `getRevision()` as the `weka.classifiers.Classifier` class already implements this method. Contributions, on the other hand, need to implement it as follows, in order to obtain the revision of this particular source file:

```
  /**
   * Returns the revision string.
   *
   * @return        the revision
   */
  public String getRevision() {
    return RevisionUtils.extract("$Revision: 6192 $");
  }
```

Note, a commit into Subversion will replace the revision number above with the actual revision number.

Testing
WEKA provides already a test framework to ensure correct basic functionality of a classifier. It is essential for the classifier to pass these tests.

Option handling
You can check the option handling of your classifier with the following tool from command-line:

```
weka.core.CheckOptionHandler -W classname [-- additional parameters]
```

All tests need to return *yes*.

GenericObjectEditor
The `CheckGOE` class checks whether all the properties available in the GUI have a tooltip accompanying them and whether the `globalInfo()` method is declared:

```
weka.core.CheckGOE -W classname [-- additional parameters]
```

All tests, once again, need to return *yes*.

Source code
Classifiers that implement the `weka.classifiers.Sourcable` interface can output Java code of the built model. In order to check the generated code, one should not only compile the code, but also test it with the following test class:

```
weka.classifiers.CheckSource
```

This class takes the original WEKA classifier, the generated code and the dataset used for generating the model (and an optional class index) as parameters. It builds the WEKA classifier on the dataset and compares the output, the one from the WEKA classifier and the one from the generated source code, whether they are the same.

Here is an example call for `weka.filters.trees.J48` and the generated class `weka.filters.WEKAWrapper` (it wraps the actual generated code in a pseudo-classifier):

```
java weka.classifiers.CheckSource \
   -W weka.classifiers.trees.J48 \
   -S weka.classifiers.WEKAWrapper \
   -t data.arff
```

It needs to return *Tests OK!*.

Unit tests
In order to make sure that your classifier applies to the WEKA criteria, you should add your classifier to the junit unit test framework, i.e., by creating a Test class. The superclass for classifier unit tests is `weka.classifiers.AbstractClassifierTest`.

17.2 Writing a new Filter

The "work horses" of preprocessing in WEKA are *filters*. They perform many tasks, from resampling data, to deleting and standardizing attributes. In the following are two different approaches covered that explain in detail how to implement a new filter:

- **default** – this is how filters had to be implemented in the past.
- **simple** – since there are mainly two types of filters, batch or stream, additional abstract classes were introduced to speed up the implementation process.

17.2.1 Default approach

The *default* approach is the most flexible, but also the most complicated one for writing a new filter. This approach has to be used, if the filter cannot be written using the *simple* approach described further below.

17.2.1.1 Implementation

The following methods are of importance for the implementation of a filter and explained in detail further down. It is also a good idea studying the Javadoc of these methods as declared in the `weka.filters.Filter` class:

- `getCapabilities()`
- `setInputFormat(Instances)`
- `getInputFormat()`
- `setOutputFormat(Instances)`
- `getOutputFormat()`
- `input(Instance)`
- `bufferInput(Instance)`
- `push(Instance)`
- `output()`
- `batchFinished()`
- `flushInput()`
- `getRevision()`

But only the following ones normally need to be modified:

- `getCapabilities()`
- `setInputFormat(Instances)`
- `input(Instance)`
- `batchFinished()`
- `getRevision()`

For more information on "Capabilities" see section 17.2.3. Please note, that the `weka.filters.Filter` superclass does not implement the `weka.core.OptionHandler` interface. See section "Option handling" on page 249.

setInputFormat(Instances)
With this call, the user tells the filter what structure, i.e., attributes, the input data has. This method also tests, whether the filter can actually process this data, according to the capabilities specified in the `getCapabilities()` method.

If the output format of the filter, i.e., the new `Instances` header, can be determined based alone on this information, then the method should set the output format via `setOutputFormat(Instances)` and return `true`, otherwise it has to return `false`.

getInputFormat()
This method returns an `Instances` object containing all currently buffered `Instance` objects from the input queue.

setOutputFormat(Instances)
`setOutputFormat(Instances)` defines the new `Instances` header for the output data. For filters that work on a row-basis, there should not be any changes between the input and output format. But filters that work on attributes, e.g., removing, adding, modifying, will affect this format. This method must be called with the appropriate `Instances` object as parameter, since all `Instance` objects being processed will rely on the output format (they use it as dataset that they belong to).

getOutputFormat()
This method returns the currently set `Instances` object that defines the output format. In case `setOutputFormat(Instances)` has not been called yet, this method will return `null`.

input(Instance)
returns `true` if the given `Instance` can be processed straight away and can be collected immediately via the `output()` method (after adding it to the output queue via `push(Instance)`, of course). This is also the case if the first batch of data has been processed and the `Instance` belongs to the second batch. Via `isFirstBatchDone()` one can query whether this `Instance` is still part of the first batch or of the second.

If the `Instance` cannot be processed immediately, e.g., the filter needs to collect all the data first before doing some calculations, then it needs to be buffered with `bufferInput(Instance)` until `batchFinished()` is called. In this case, the method needs to return `false`.

bufferInput(Instance)
In case an `Instance` cannot be processed immediately, one can use this method to buffer them in the input queue. All buffered `Instance` objects are available via the `getInputFormat()` method.

push(Instance)
adds the given `Instance` to the output queue.

output()
Returns the next `Instance` object from the output queue and removes it from there. In case there is no `Instance` available this method returns `null`.

batchFinished()
signals the end of a dataset being pushed through the filter. In case of a filter that could not process the data of the first batch immediately, this is the place to determine what the output format will be (and set if via `setOutputFormat(Instances)`) and finally process the input data. The currently available data can be retrieved with the `getInputFormat()` method. After processing the data, one needs to call `flushInput()` to remove all the pending input data.

flushInput()
`flushInput()` removes all buffered `Instance` objects from the input queue. This method must be called after all the `Instance` objects have been processed in the `batchFinished()` method.

Option handling

If the filter should be able to handle command-line options, then the interface `weka.core.OptionHandler` needs to be implemented. In addition to that, the following code should be added at the end of the `setOptions(String[])` method:

```
if (getInputFormat() != null) {
    setInputFormat(getInputFormat());
}
```

This will inform the filter about changes in the options and therefore reset it.

17.2.1.2 Examples

The following examples, covering batch and stream filters, illustrate the filter framework and how to use it.

Unseeded random number generators like `Math.random()` should **never** be used since they will produce different results in each run and repeatable experiments are essential in machine learning.

BatchFilter

This simple batch filter adds a new attribute called *blah* at the end of the dataset. The rows of this attribute contain only the row's index in the data. Since the batch-filter does not have to see all the data before creating the output format, the `setInputFormat(Instances)` sets the output format and returns `true` (indicating that the output format can be queried immediately). The `batchFinished()` method performs the processing of all the data.

```
import weka.core.*;
import weka.core.Capabilities.*;

public class BatchFilter extends Filter {

  public String globalInfo() {
    return   "A batch filter that adds an additional attribute 'blah' at the end "
           + "containing the index of the processed instance. The output format "
           + "can be collected immediately.";
  }

  public Capabilities getCapabilities() {
    Capabilities result = super.getCapabilities();
    result.enableAllAttributes();
    result.enableAllClasses();
    result.enable(Capability.NO_CLASS);  // filter doesn't need class to be set
    return result;
  }

  public boolean setInputFormat(Instances instanceInfo) throws Exception {
    super.setInputFormat(instanceInfo);
    Instances outFormat = new Instances(instanceInfo, 0);
    outFormat.insertAttributeAt(new Attribute("blah"),
  outFormat.numAttributes());
    setOutputFormat(outFormat);
    return true;  // output format is immediately available
  }

  public boolean batchFinished() throws Exception {
    if (getInputFormat() = null)
      throw new NullPointerException("No input instance format defined");
    Instances inst = getInputFormat();
    Instances outFormat = getOutputFormat();
    for (int i = 0; i < inst.numInstances(); i++) {
      double[] newValues = new double[outFormat.numAttributes()];
      double[] oldValues = inst.instance(i).toDoubleArray();
      System.arraycopy(oldValues, 0, newValues, 0, oldValues.length);
      newValues[newValues.length - 1] = i;
      push(new Instance(1.0, newValues));
    }
    flushInput();
    m_NewBatch = true;
    m_FirstBatchDone = true;
    return (numPendingOutput() != 0);
  }

  public static void main(String[] args) {
    runFilter(new BatchFilter(), args);
  }
}
```

BatchFilter2

In contrast to the first batch filter, this one here cannot determine the output format immediately (the number of instances in the first batch is part of the attribute name now). This is done in the `batchFinished()` method.

```
import weka.core.*;
import weka.core.Capabilities.*;

public class BatchFilter2 extends Filter {

  public String globalInfo() {
    return   "A batch filter that adds an additional attribute 'blah' at the end "
           + "containing the index of the processed instance. The output format "
           + "cannot be collected immediately.";
  }

  public Capabilities getCapabilities() {
    Capabilities result = super.getCapabilities();
    result.enableAllAttributes();
    result.enableAllClasses();
    result.enable(Capability.NO_CLASS);  // filter doesn't need class to be set
    return result;
  }

  public boolean batchFinished() throws Exception {
    if (getInputFormat() = null)
      throw new NullPointerException("No input instance format defined");
    // output format still needs to be set (depends on first batch of data)
    if (!isFirstBatchDone()) {
      Instances outFormat = new Instances(getInputFormat(), 0);
      outFormat.insertAttributeAt(new Attribute(
          "blah-" + getInputFormat().numInstances()), outFormat.numAttributes());
      setOutputFormat(outFormat);
    }
    Instances inst = getInputFormat();
    Instances outFormat = getOutputFormat();
    for (int i = 0; i < inst.numInstances(); i++) {
      double[] newValues = new double[outFormat.numAttributes()];
      double[] oldValues = inst.instance(i).toDoubleArray();
      System.arraycopy(oldValues, 0, newValues, 0, oldValues.length);
      newValues[newValues.length - 1] = i;
      push(new Instance(1.0, newValues));
    }
    flushInput();
    m_NewBatch = true;
    m_FirstBatchDone = true;
    return (numPendingOutput() != 0);
  }

  public static void main(String[] args) {
    runFilter(new BatchFilter2(), args);
  }
}
```

BatchFilter3

As soon as this batch filter's first batch is done, it can process `Instance` objects immediately in the `input(Instance)` method. It adds a new attribute which contains just a random number, but the random number generator being used is seeded with the number of instances from the first batch.

```
import weka.core.*;
import weka.core.Capabilities.*;
import java.util.Random;

public class BatchFilter3 extends Filter {

  protected int m_Seed;
  protected Random m_Random;

  public String globalInfo() {
    return   "A batch filter that adds an attribute 'blah' at the end "
           + "containing a random number. The output format cannot be collected "
           + "immediately.";
  }

  public Capabilities getCapabilities() {
    Capabilities result = super.getCapabilities();
    result.enableAllAttributes();
    result.enableAllClasses();
    result.enable(Capability.NO_CLASS);  // filter doesn't need class to be set
    return result;
  }

  public boolean input(Instance instance) throws Exception {
    if (getInputFormat() = null)
      throw new NullPointerException("No input instance format defined");
    if (isNewBatch()) {
      resetQueue();
      m_NewBatch = false;
    }
    if (isFirstBatchDone())
      convertInstance(instance);
    else
      bufferInput(instance);
    return isFirstBatchDone();
  }

  public boolean batchFinished() throws Exception {
    if (getInputFormat() = null)
      throw new NullPointerException("No input instance format defined");
    // output format still needs to be set (random number generator is seeded
    // with number of instances of first batch)
    if (!isFirstBatchDone()) {
      m_Seed = getInputFormat().numInstances();
      Instances outFormat = new Instances(getInputFormat(), 0);
      outFormat.insertAttributeAt(new Attribute(
          "blah-" + getInputFormat().numInstances()), outFormat.numAttributes());
      setOutputFormat(outFormat);
    }
    Instances inst = getInputFormat();
    for (int i = 0; i < inst.numInstances(); i++) {
      convertInstance(inst.instance(i));
    }
    flushInput();
    m_NewBatch = true;
    m_FirstBatchDone = true;
    m_Random = null;
    return (numPendingOutput() != 0);
  }

  protected void convertInstance(Instance instance) {
    if (m_Random = null)
      m_Random = new Random(m_Seed);
    double[] newValues = new double[instance.numAttributes() + 1];
    double[] oldValues = instance.toDoubleArray();
    newValues[newValues.length - 1] = m_Random.nextInt();
    System.arraycopy(oldValues, 0, newValues, 0, oldValues.length);
    push(new Instance(1.0, newValues));
  }

  public static void main(String[] args) {
    runFilter(new BatchFilter3(), args);
  }
}
```

StreamFilter

This stream filter adds a random number (the seed value is hard-coded) at the end of each `Instance` of the input data. Since this does not rely on having access to the full data of the first batch, the output format is accessible immediately after using `setInputFormat(Instances)`. All the `Instance` objects are immediately processed in `input(Instance)` via the `convertInstance(Instance)` method, which pushes them immediately to the output queue.

```
import weka.core.*;
import weka.core.Capabilities.*;
import java.util.Random;

public class StreamFilter extends Filter {

  protected Random m_Random;

  public String globalInfo() {
    return   "A stream filter that adds an attribute 'blah' at the end "
           + "containing a random number. The output format can be collected "
           + "immediately.";
  }

  public Capabilities getCapabilities() {
    Capabilities result = super.getCapabilities();
    result.enableAllAttributes();
    result.enableAllClasses();
    result.enable(Capability.NO_CLASS);  // filter doesn't need class to be set
    return result;
  }

  public boolean setInputFormat(Instances instanceInfo) throws Exception {
    super.setInputFormat(instanceInfo);
    Instances outFormat = new Instances(instanceInfo, 0);
    outFormat.insertAttributeAt(new Attribute("blah"),
  outFormat.numAttributes());
    setOutputFormat(outFormat);
    m_Random = new Random(1);
    return true;  // output format is immediately available
  }

  public boolean input(Instance instance) throws Exception {
    if (getInputFormat() = null)
      throw new NullPointerException("No input instance format defined");
    if (isNewBatch()) {
      resetQueue();
      m_NewBatch = false;
    }
    convertInstance(instance);
    return true;  // can be immediately collected via output()
  }

  protected void convertInstance(Instance instance) {
    double[] newValues = new double[instance.numAttributes() + 1];
    double[] oldValues = instance.toDoubleArray();
    newValues[newValues.length - 1] = m_Random.nextInt();
    System.arraycopy(oldValues, 0, newValues, 0, oldValues.length);
    push(new Instance(1.0, newValues));
  }

  public static void main(String[] args) {
    runFilter(new StreamFilter(), args);
  }
}
```

17.2.2 Simple approach

The base filters and interfaces are all located in the following package:

```
weka.filters
```

One can basically divide filters roughly into two different kinds of filters:

- **batch filters** – they need to see the whole dataset before they can start processing it, which they do in one go
- **stream filters** – they can start producing output right away and the data just passes through while being modified

You can subclass one of the following abstract filters, depending on the kind of classifier you want to implement:

- `weka.filters.SimpleBatchFilter`
- `weka.filters.SimpleStreamFilter`

These filters simplify the rather general and complex framework introduced by the abstract superclass `weka.filters.Filter`. One only needs to implement a couple of abstract methods that will process the actual data and override, if necessary, a few existing methods for option handling.

17.2.2.1 SimpleBatchFilter

Only the following abstract methods need to be implemented:

- `globalInfo()` – returns a short description of what the filter does; will be displayed in the GUI
- `determineOutputFormat(Instances)` – generates the new format, based on the input data
- `process(Instances)` – processes the whole dataset in one go
- `getRevision()` – returns the Subversion revision information, see section “Revisions” on page 258

If more options are necessary, then the following methods need to be overridden:

- `listOptions()` – returns an enumeration of the available options; these are printed if one calls the filter with the -h option
- `setOptions(String[])` – parses the given option array, that were passed from command-line
- `getOptions()` – returns an array of options, resembling the current setup of the filter

See section “Methods” on page 237 and section “Parameters” on page 241 for more information.

In the following an example implementation that adds an additional attribute at the end, containing the index of the processed instance:

```
import weka.core.*;
import weka.core.Capabilities.*;
import weka.filters.*;

public class SimpleBatch extends SimpleBatchFilter {

  public String globalInfo() {
    return   "A simple batch filter that adds an additional attribute 'blah' at the end "
           + "containing the index of the processed instance.";
  }

  public Capabilities getCapabilities() {
    Capabilities result = super.getCapabilities();
    result.enableAllAttributes();
    result.enableAllClasses();
    result.enable(Capability.NO_CLASS);  //// filter doesn't need class to be set//
    return result;
  }

  protected Instances determineOutputFormat(Instances inputFormat) {
    Instances result = new Instances(inputFormat, 0);
    result.insertAttributeAt(new Attribute("blah"), result.numAttributes());
    return result;
  }

  protected Instances process(Instances inst) {
    Instances result = new Instances(determineOutputFormat(inst), 0);
    for (int i = 0; i < inst.numInstances(); i++) {
      double[] values = new double[result.numAttributes()];
      for (int n = 0; n < inst.numAttributes(); n++)
        values[n] = inst.instance(i).value(n);
      values[values.length - 1] = i;
      result.add(new Instance(1, values));
    }
    return result;
  }

  public static void main(String[] args) {
    runFilter(new SimpleBatch(), args);
  }
}
```

17.2.2.2 SimpleStreamFilter

Only the following abstract methods need to be implemented for a stream filter:

- `globalInfo()` – returns a short description of what the filter does; will be displayed in the GUI
- `determineOutputFormat(Instances)` – generates the new format, based on the input data
- `process(Instance)` – processes a single instance and turns it from the old format into the new one
- `getRevision()` – returns the Subversion revision information, see section "Revisions" on page 258

If more options are necessary, then the following methods need to be overridden:

- `listOptions()` – returns an enumeration of the available options; these are printed if one calls the filter with the -h option
- `setOptions(String[])` – parses the given option array, that were passed from command-line
- `getOptions()` – returns an array of options, resembling the current setup of the filter

See also section 17.1.4.1, covering "Methods" for classifiers.

In the following an example implementation of a stream filter that adds an extra attribute at the end, which is filled with random numbers. The `reset()` method is only used in this example, since the random number generator needs to be re-initialized in order to obtain repeatable results.

```
import weka.core.*;
import weka.core.Capabilities.*;
import weka.filters.*;
import java.util.Random;

public class SimpleStream extends SimpleStreamFilter {

  protected Random m_Random;

  public String globalInfo() {
    return   "A simple stream filter that adds an attribute 'blah' at the end "
           + "containing a random number.";
  }

  public Capabilities getCapabilities() {
    Capabilities result = super.getCapabilities();
    result.enableAllAttributes();
    result.enableAllClasses();
    result.enable(Capability.NO_CLASS);  //// filter doesn't need class to be set//
    return result;
  }

  protected void reset() {
    super.reset();
    m_Random = new Random(1);
  }

  protected Instances determineOutputFormat(Instances inputFormat) {
    Instances result = new Instances(inputFormat, 0);
    result.insertAttributeAt(new Attribute("blah"), result.numAttributes());
    return result;
  }

  protected Instance process(Instance inst) {
    double[] values = new double[inst.numAttributes() + 1];
    for (int n = 0; n < inst.numAttributes(); n++)
      values[n] = inst.value(n);
    values[values.length - 1] = m_Random.nextInt();
    Instance result = new Instance(1, values);
    return result;
  }

  public static void main(String[] args) {
    runFilter(new SimpleStream(), args);
  }
}
```

A real-world implementation of a stream filter is the `MultiFilter` class (package `weka.filters`), which passes the data through all the filters it contains. Depending on whether all the used filters are streamable or not, it acts either as *stream* filter or as *batch* filter.

17.2.2.3 Internals

Some useful methods of the filter classes:

- `isNewBatch()` – returns `true` if an instance of the filter was just instantiated or a new batch was started via the `batchFinished()` method.
- `isFirstBatchDone()` – returns `true` as soon as the first batch was finished via the `batchFinished()` method. Useful for supervised filters, which should not be altered after being trained with the first batch of instances.

17.2.3 Capabilities

Filters implement the `weka.core.CapabilitiesHandler` interface like the classifiers. This method returns what kind of data the filter is able to process. Needs to be adapted for each individual filter, since the default implementation allows the processing of all kinds of attributes and classes. Otherwise correct functioning of the filter cannot be guaranteed. See section "Capabilities" on page 242 for more information.

17.2.4 Packages

A few comments about the different filter sub-packages:

- **supervised** – contains supervised filters, i.e., filters that take class distributions into account. Must implement the `weka.filters.SupervisedFilter` interface.
  - **attribute** – filters that work column-wise.
  - **instance** – filters that work row-wise.
- **unsupervised** – contains unsupervised filters, i.e., they work without taking any class distributions into account. The filter must implement the `weka.filters.UnsupervisedFilter` interface.
  - **attribute** – filters that work column-wise.
  - **instance** – filters that work row-wise.

Javadoc

The Javadoc generation works the same as with classifiers. See section "Javadoc" on page 244 for more information.

17.2.5 Revisions

Filters, like classifiers, implement the `weka.core.RevisionHandler` interface. This provides the functionality of obtaining the Subversion revision from within Java. Filters that are not part of the official WEKA distribution do not have to implement the method `getRevision()` as the `weka.filters.Filter` class already implements this method. Contributions, on the other hand, need to implement it, in order to obtain the revision of this particular source file. See section "Revisions" on page 245.

17.2.6 Testing

WEKA provides already a test framework to ensure correct basic functionality of a filter. It is essential for the filter to pass these tests.

17.2.6.1 Option handling

You can check the option handling of your filter with the following tool from command-line:

```
weka.core.CheckOptionHandler -W classname [-- additional parameters]
```

All tests need to return *yes*.

17.2.6.2 GenericObjectEditor

The `CheckGOE` class checks whether all the properties available in the GUI have a tooltip accompanying them and whether the `globalInfo()` method is declared:

```
weka.core.CheckGOE -W classname [-- additional parameters]
```

All tests, once again, need to return *yes*.

17.2.6.3 Source code

Filters that implement the `weka.filters.Sourcable` interface can output Java code of their internal representation. In order to check the generated code, one should not only compile the code, but also test it with the following test class:

```
weka.filters.CheckSource
```

This class takes the original WEKA filter, the generated code and the dataset used for generating the source code (and an optional class index) as parameters. It builds the WEKA filter on the dataset and compares the output, the one from the WEKA filter and the one from the generated source code, whether they are the same.

Here is an example call for `weka.filters.unsupervised.attribute.ReplaceMissingValues` and the generated class `weka.filters.WEKAWrapper` (it wraps the actual generated code in a pseudo-filter):

```
java weka.filters.CheckSource \
   -W weka.filters.unsupervised.attribute.ReplaceMissingValues \
   -S weka.filters.WEKAWrapper \
   -t data.arff
```

It needs to return *Tests OK!*.

17.2.6.4 Unit tests

In order to make sure that your filter applies to the WEKA criteria, you should add your filter to the junit unit test framework, i.e., by creating a Test class. The superclass for filter unit tests is `weka.filters.AbstractFilterTest`.

17.3 Writing other algorithms

The previous sections covered how to implement classifiers and filters. In the following you will find some information on how to implement clusterers, associators and attribute selection algorithms. The various algorithms are only covered briefly, since other important components (capabilities, option handling, revisions) have already been discussed in the other chapters.

17.3.1 Clusterers

Superclasses and interfaces

All clusterers implement the interface `weka.clusterers.Clusterer`, but most algorithms will be most likely derived (directly or further up in the class hierarchy) from the abstract superclass `weka.clusterers.AbstractClusterer`.

`weka.clusterers.SingleClustererEnhancer` is used for meta-clusterers, like the `FilteredClusterer` that filters the data on-the-fly for the base-clusterer.

Here are some common interfaces that can be implemented:

- `weka.clusterers.DensityBasedClusterer` – for clusterers that can estimate the density for a given instance. `AbstractDensityBasedClusterer` already implements this interface.
- `weka.clusterers.UpdateableClusterer` – clusterers that can generate their model incrementally implement this interface, like `CobWeb`.
- `NumberOfClustersRequestable` – is for clusterers that allow to specify the number of clusters to generate, like `SimpleKMeans`.
- `weka.core.Randomizable` – for clusterers that support randomization in one way or another. `RandomizableClusterer`, `RandomizableDensityBasedClusterer` and `RandomizableSingleClustererEnhancer` all implement this interface already.

Methods

In the following a short description of methods that are common to all cluster algorithms, see also the Javadoc for the `Clusterer` interface.

buildClusterer(Instances)
Like the `buildClassifier(Instances)` method, this method completely rebuilds the model. Subsequent calls of this method with the same dataset must result in exactly the same model being built. This method also tests the training data against the capabilities of this this clusterer:

```
public void buildClusterer(Instances data) throws Exception {
  // test data against capabilities
  getCapabilities().testWithFail(data);
  // actual model generation
  ...
}
```

clusterInstance(Instance)
returns the index of the cluster the provided `Instance` belongs to.

distributionForInstance(Instance)
returns the cluster membership for this `Instance` object. The membership is a double array containing the probabilities for each cluster.

numberOfClusters()
returns the number of clusters that the model contains, after the model has been generated with the `buildClusterer(Instances)` method.

getCapabilities()
see section "Capabilities" on page 242 for more information.

toString()
should output some information on the generated model. Even though this is not required, it is rather useful for the user to get some feedback on the built model.

main(String[])
executes the clusterer from command-line. If your new algorithm is called `FunkyClusterer`, then use the following code as your `main` method:

```
/**
 * Main method for executing this clusterer.
 *
 * @param args the options, use "-h" to display options
 */
public static void main(String[] args) {
  AbstractClusterer.runClusterer(new FunkyClusterer(), args);
}
```

Testing

For some basic tests from the command-line, you can use the following test class:

```
weka.clusterers.CheckClusterer -W classname [further options]
```

For junit tests, you can subclass the `weka.clusterers.AbstractClustererTest` class and add additional tests.

17.3.2 Attribute selection

Attribute selection consists basically of two different types of classes:

- *evaluator* – determines the merit of single attributes or subsets of attributes
- *search* algorithm – the search heuristic

Each of the them will be discussed separately in the following sections.

Evaluator

The evaluator algorithm is responsible for determining merit of the current attribute selection.

Superclasses and interfaces
The ancestor for all evaluators is the `weka.attributeSelection.ASEvaluation` class.

Here are some interfaces that are commonly implemented by evaluators:

- `AttributeEvaluator` – evaluates only single attributes
- `SubsetEvaluator` – evaluates subsets of attributes
- `AttributeTransformer` – evaluators that transform the input data

Methods
In the following a brief description of the main methods of an evaluator.

buildEvaluator(Instances)
Generates the attribute evaluator. Subsequent calls of this method with the same data (and the same search algorithm) must result in the same attributes being selected. This method also checks the data against the capabilities:

```
public void buildEvaluator (Instances data) throws Exception {
  // can evaluator handle data?
  getCapabilities().testWithFail(data);
  // actual initialization of evaluator
  ...
}
```

postProcess(int[])
can be used for optional post-processing of the selected attributes, e.g., for ranking purposes.

main(String[])
executes the evaluator from command-line. If your new algorithm is called `FunkyEvaluator`, then use the following code as your `main` method:

```
/**
 * Main method for executing this evaluator.
 *
 * @param args the options, use "-h" to display options
 */
public static void main(String[] args) {
  ASEvaluation.runEvaluator(new FunkyEvaluator(), args);
}
```

Search

The search algorithm defines the heuristic of searching, e.g, exhaustive search, greedy or genetic.

Superclasses and interfaces
The ancestor for all search algorithms is the `weka.attributeSelection.ASSearch` class.

Interfaces that can be implemented, if applicable, by a search algorithm:

- `RankedOutputSearch` – for search algorithms that produce ranked lists of attributes
- `StartSetHandler` – search algorithms that can make use of a start set of attributes implement this interface

Methods
Search algorithms are rather basic classes in regards to methods that need to be implemented. Only the following method needs to be implemented:

search(ASEvaluation,Instances)
uses the provided evaluator to guide the search.

Testing

For some basic tests from the command-line, you can use the following test class:

```
weka.attributeSelection.CheckAttributeSelection
  -eval classname -search classname [further options]
```

For junit tests, you can subclass the `weka.attributeSelection.AbstractEvaluatorTest` or `weka.attributeSelection.AbstractSearchTest` class and add additional tests.

17.3.3 Associators

Superclasses and interfaces

The interface `weka.associations.Associator` is common to all associator algorithms. But most algorithms will be derived from `AbstractAssociator`, an abstract class implementing this interface. As with classifiers and clusterers, you can also implement a meta-associator, derived from `SingleAssociatorEnhancer`. An example for this is the `FilteredAssociator`, which filters the training data on-the-fly for the base-associator.

The only other interface that is used by some other association algorithms, is the `weka.clusterers.CARuleMiner` one. Associators that learn class association rules implement this interface, like `Apriori`.

Methods

The associators are very basic algorithms and only support building of the model.

buildAssociations(Instances)
Like the `buildClassifier(Instances)` method, this method completely rebuilds the model. Subsequent calls of this method with the same dataset must result in exactly the same model being built. This method also tests the training data against the capabilities:

```
public void buildAssociations(Instances data) throws Exception {
  // other necessary setups
  ...
  // test data against capabilities
  getCapabilities().testWithFail(data);
  // actual model generation
  ...
}
```

getCapabilities()
see section "Capabilities" on page 242 for more information.

toString()
should output some information on the generated model. Even though this is not required, it is rather useful for the user to get some feedback on the built model.

main(String[])
executes the associator from command-line. If your new algorithm is called `FunkyAssociator`, then use the following code as your `main` method:

```
/**
 * Main method for executing this associator.
 *
 * @param args the options, use "-h" to display options
 */
public static void main(String[] args) {
  AbstractAssociator.runAssociator(new FunkyAssociator(), args);
}
```

Testing

For some basic tests from the command-line, you can use the following test class:

```
weka.associations.CheckAssociator -W classname [further options]
```

For junit tests, you can subclass the `weka.associations.AbstractAssociatorTest` class and add additional tests.

17.4 Extending the Explorer

The plugin architecture of the Explorer allows you to add new functionality easily without having to dig into the code of the Explorer itself. In the following you will find information on how to add new tabs, like the “Classify” tab, and new visualization plugins for the “Classify” tab.

17.4.1 Adding tabs

The Explorer is a handy tool for initial exploration of your data – for proper statistical evaluation, the Experimenter should be used instead. But if the available functionality is not enough, you can always add your own custom-made tabs to the Explorer.

17.4.1.1 Requirements

Here is roughly what is required in order to add a new tab (the examples below go into more detail):

- your class must be derived from `javax.swing.JPanel`
- the interface `weka.gui.explorer.Explorer.ExplorerPanel` must be implemented by your class
- optional interfaces
  - `weka.gui.explorer.Explorer.LogHandler` – in case you want to take advantage of the logging in the Explorer
  - `weka.gui.explorer.Explorer.CapabilitiesFilterChangeListener` – in case your class needs to be notified of changes in the Capabilities, e.g., if new data is loaded into the Explorer
- adding the classname of your class to the Tabs property in the `Explorer.props` file

17.4.1.2 Examples

The following examples demonstrate the plugin architecture. Only the necessary details are discussed, as the full source code is available from the WEKA Examples [3] (package `wekaexamples.gui.explorer`).

SQL worksheet

Purpose

Displaying the SqlViewer as a tab in the Explorer instead of using it either via the *Open DB...* button or as standalone application. Uses the existing components already available in WEKA and just assembles them in a `JPanel`. Since this tab does not rely on a dataset being loaded into the Explorer, it will be used as a *standalone* one.

Useful for people who are working a lot with databases and would like to have an SQL worksheet available all the time instead of clicking on a button every time to open up a database dialog.

Implementation

- class is derived from `javax.swing.JPanel` and implements the interface `weka.gui.Explorer.ExplorerPanel` (the full source code also imports the `weka.gui.Explorer.LogHandler` interface, but that is only additional functionality):

```
public class SqlPanel
  extends JPanel
  implements ExplorerPanel {
```

- some basic members that we need to have

```
/** the parent frame */
protected Explorer m_Explorer = null;

/** sends notifications when the set of working instances gets changed*/
protected PropertyChangeSupport m_Support = new PropertyChangeSupport(this);
```

- methods we need to implement due to the used interfaces

```
/** Sets the Explorer to use as parent frame */
public void setExplorer(Explorer parent) {
  m_Explorer = parent;
}

/** returns the parent Explorer frame */
public Explorer getExplorer() {
  return m_Explorer;
}

/** Returns the title for the tab in the Explorer */
public String getTabTitle() {
  return "SQL";  // what's displayed as tab-title, e.g., Classify
}

/** Returns the tooltip for the tab in the Explorer */
public String getTabTitleToolTip() {
  return "Retrieving data from databases";  // the tooltip of the tab
}

/** ignored, since we "generate" data and not receive it */
public void setInstances(Instances inst) {
}

/** PropertyChangeListener which will be notified of value changes. */
public void addPropertyChangeListener(PropertyChangeListener l) {
  m_Support.addPropertyChangeListener(l);
}

/** Removes a PropertyChangeListener. */
public void removePropertyChangeListener(PropertyChangeListener l) {
  m_Support.removePropertyChangeListener(l);
}
```

- additional GUI elements

```
/** the actual SQL worksheet */
protected SqlViewer m_Viewer;

/** the panel for the buttons */
protected JPanel m_PanelButtons;

/** the Load button - makes the data available in the Explorer */
protected JButton m_ButtonLoad = new JButton("Load data");

/** displays the current query */
protected JLabel m_LabelQuery = new JLabel("");
```

- loading the data into the Explorer by clicking on the *Load* button will fire a *propertyChange* event:

```
  m_ButtonLoad.addActionListener(new ActionListener() {
    public void actionPerformed(ActionEvent evt){
      m_Support.firePropertyChange("", null, null);
    }
  });
```

- the *propertyChange* event will perform the actual loading of the data, hence we add an anonymous property change listener to our panel:

```
addPropertyChangeListener(new PropertyChangeListener() {
  public void propertyChange(PropertyChangeEvent e) {
    try {
      // load data
      InstanceQuery query = new InstanceQuery();
      query.setDatabaseURL(m_Viewer.getURL());
      query.setUsername(m_Viewer.getUser());
      query.setPassword(m_Viewer.getPassword());
      Instances data = query.retrieveInstances(m_Viewer.getQuery());

      // set data in preprocess panel (also notifies of capabilties changes)
      getExplorer().getPreprocessPanel().setInstances(data);
    }
    catch (Exception ex) {
      ex.printStackTrace();
    }
  }
});
```

- In order to add our `SqlPanel` to the list of tabs displayed in the Explorer, we need to modify the `Explorer.props` file (just extract it from the `weka.jar` and place it in your home directory). The `Tabs` property must look like this:

```
Tabs=weka.gui.explorer.SqlPanel,\
     weka.gui.explorer.ClassifierPanel,\
     weka.gui.explorer.ClustererPanel,\
     weka.gui.explorer.AssociationsPanel,\
     weka.gui.explorer.AttributeSelectionPanel,\
     weka.gui.explorer.VisualizePanel
```

Screenshot

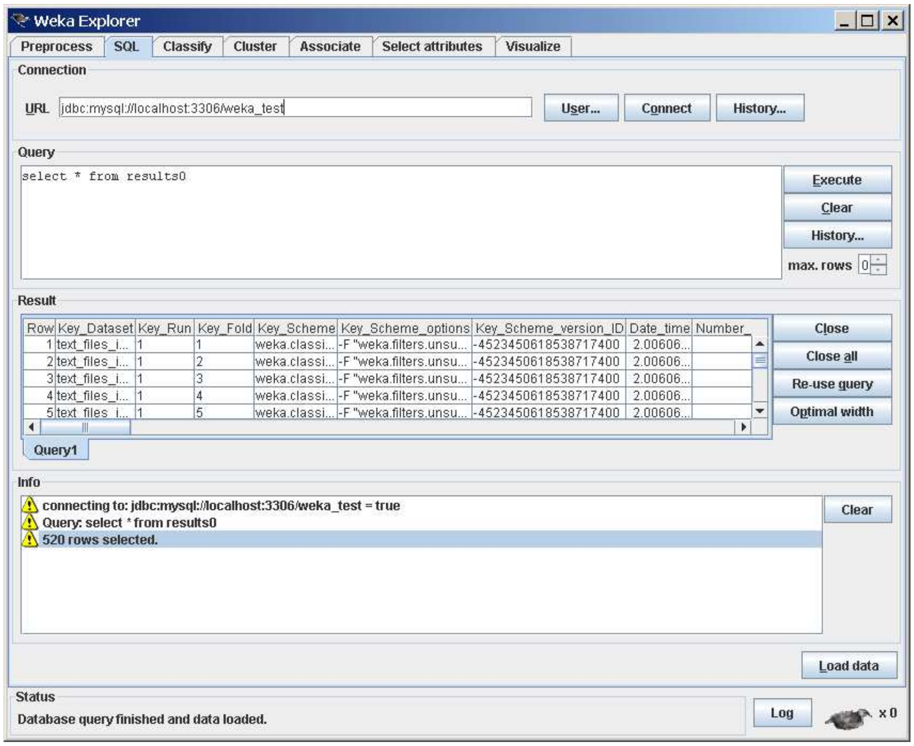
Weka Explorer
Preprocess
SQL
Classify
Cluster
Associate
Select attributes
Visualize
Connection
URL
jdbc:mysql://localhost:3306/weka_test
User...
Connect
History...
Query
select * from results0
Execute
Clear
History...
max. rows
Result
Close
Close all
Re-use query
Optimal width
Query1
Info
connecting to: jdbc:mysql://localhost:3306/weka_test = true
Query: select * from results0
520 rows selected.
Clear
Load data
Status
Database query finished and data loaded.
Log

Artificial data generation

Purpose

Instead of only having a *Generate...* button in the PreprocessPanel or using it from command-line, this example creates a new panel to be displayed as extra tab in the Explorer. This tab will be available regardless whether a dataset is already loaded or not (= standalone).

Implementation

- class is derived from `javax.swing.JPanel` and implements the interface `weka.gui.Explorer.ExplorerPanel` (the full source code also imports the `weka.gui.Explorer.LogHandler` interface, but that is only additional functionality):

```
public class GeneratorPanel
  extends JPanel
  implements ExplorerPanel {
```

- some basic members that we need to have (the same as for the `SqlPanel` class):

```
/** the parent frame */
protected Explorer m_Explorer = null;

/** sends notifications when the set of working instances gets changed*/
protected PropertyChangeSupport m_Support = new PropertyChangeSupport(this);
```

- methods we need to implement due to the used interfaces (almost identical to `SqlPanel`):

```
/** Sets the Explorer to use as parent frame */
public void setExplorer(Explorer parent) {
  m_Explorer = parent;
}
/** returns the parent Explorer frame */
public Explorer getExplorer() {
  return m_Explorer;
}
/** Returns the title for the tab in the Explorer */
public String getTabTitle() {
  return "DataGeneration";  // what's displayed as tab-title, e.g., Classify
}
/** Returns the tooltip for the tab in the Explorer */
public String getTabTitleToolTip() {
  return "Generating artificial datasets";  // the tooltip of the tab
}
/** ignored, since we "generate" data and not receive it */
public void setInstances(Instances inst) {
}
/** PropertyChangeListener which will be notified of value changes. */
public void addPropertyChangeListener(PropertyChangeListener l) {
  m_Support.addPropertyChangeListener(l);
}
/** Removes a PropertyChangeListener. */
public void removePropertyChangeListener(PropertyChangeListener l) {
  m_Support.removePropertyChangeListener(l);
}
```

- additional GUI elements:

```
/** the GOE for the generators */
protected GenericObjectEditor m_GeneratorEditor = new GenericObjectEditor();

/** the text area for the output of the generated data */
protected JTextArea m_Output = new JTextArea();

/** the Generate button */
protected JButton m_ButtonGenerate = new JButton("Generate");

/** the Use button */
protected JButton m_ButtonUse = new JButton("Use");
```

- the *Generate* button does not load the generated data directly into the Explorer, but only outputs it in the `JTextArea` (the *Use* button loads the data - see further down):

```
m_ButtonGenerate.addActionListener(new ActionListener(){
  public void actionPerformed(ActionEvent evt){
    DataGenerator generator = (DataGenerator) m_GeneratorEditor.getValue();
    String relName = generator.getRelationName();

    String cname = generator.getClass().getName().replaceAll(".*\\.", "");
    String cmd = generator.getClass().getName();
    if (generator instanceof OptionHandler)
      cmd += " "+Utils.joinOptions(((OptionHandler)generator).getOptions());

    try {
      // generate data
      StringWriter output = new StringWriter();
      generator.setOutput(new PrintWriter(output));
      DataGenerator.makeData(generator, generator.getOptions());
      m_Output.setText(output.toString());
    }
    catch (Exception ex) {
      ex.printStackTrace();
      JOptionPane.showMessageDialog(
        getExplorer(), "Error generating data:\n" + ex.getMessage(),
        "Error", JOptionPane.ERROR_MESSAGE);
    }

    generator.setRelationName(relName);
  }
});
```

- the *Use* button finally fires a *propertyChange* event that will load the data into the Explorer:

```
m_ButtonUse.addActionListener(new ActionListener(){
  public void actionPerformed(ActionEvent evt){
    m_Support.firePropertyChange("", null, null);
  }
});
```

- the *propertyChange* event will perform the actual loading of the data, hence we add an anonymous property change listener to our panel:

```
addPropertyChangeListener(new PropertyChangeListener() {
  public void propertyChange(PropertyChangeEvent e) {
    try {
      Instances data = new Instances(new StringReader(m_Output.getText()));
      // set data in preprocess panel (also notifies of capabilties changes)
      getExplorer().getPreprocessPanel().setInstances(data);
    }
    catch (Exception ex) {
      ex.printStackTrace();
      JOptionPane.showMessageDialog(
        getExplorer(), "Error generating data:\n" + ex.getMessage(),
        "Error", JOptionPane.ERROR_MESSAGE);
    }
  }
});
```

- In order to add our `GeneratorPanel` to the list of tabs displayed in the Explorer, we need to modify the `Explorer.props` file (just extract it from the `weka.jar` and place it in your home directory). The `Tabs` property must look like this:

```
Tabs=weka.gui.explorer.GeneratorPanel:standalone,\
     weka.gui.explorer.ClassifierPanel,\
     weka.gui.explorer.ClustererPanel,\
     weka.gui.explorer.AssociationsPanel,\
     weka.gui.explorer.AttributeSelectionPanel,\
     weka.gui.explorer.VisualizePanel
```

- **Note:** the standalone option is used to make the tab available without requiring the preprocess panel to load a dataset first.

Screenshot

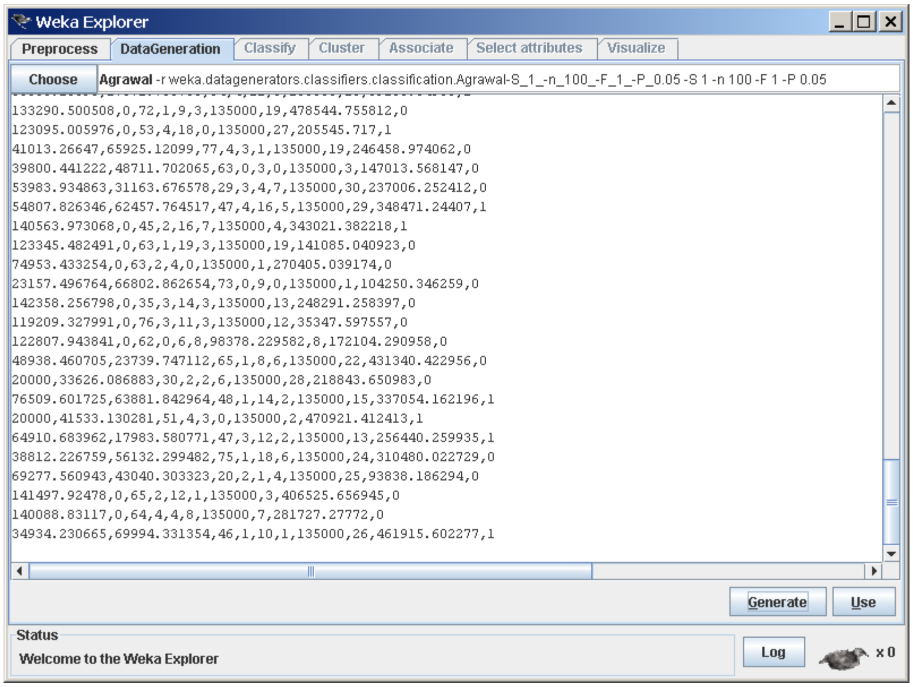

Experimenter "light"

Purpose

By default the Classify panel only performs 1 run of 10-fold cross-validation. Since most classifiers are rather sensitive to the order of the data being presented to them, those results can be too optimistic or pessimistic. Averaging the results over 10 runs with differently randomized train/test pairs returns more reliable results. And this is where this plugin comes in: it can be used to obtain statistical sound results for a specific classifier/dataset combination, without having to setup a whole experiment in the Experimenter.

Implementation

- Since this plugin is rather bulky, we omit the implementation details, but the following can be said:
  - based on the `weka.gui.explorer.ClassifierPanel`
  - the actual code doing the work follows the example in the *Using the Experiment API* wiki article [2]
- In order to add our `ExperimentPanel` to the list of tabs displayed in the Explorer, we need to modify the `Explorer.props` file (just extract it from the `weka.jar` and place it in your home directory). The `Tabs` property must look like this:

```
Tabs=weka.gui.explorer.ClassifierPanel,\
     weka.gui.explorer.ExperimentPanel,\
     weka.gui.explorer.ClustererPanel,\
     weka.gui.explorer.AssociationsPanel,\
     weka.gui.explorer.AttributeSelectionPanel,\
     weka.gui.explorer.VisualizePanel
```

Screenshot

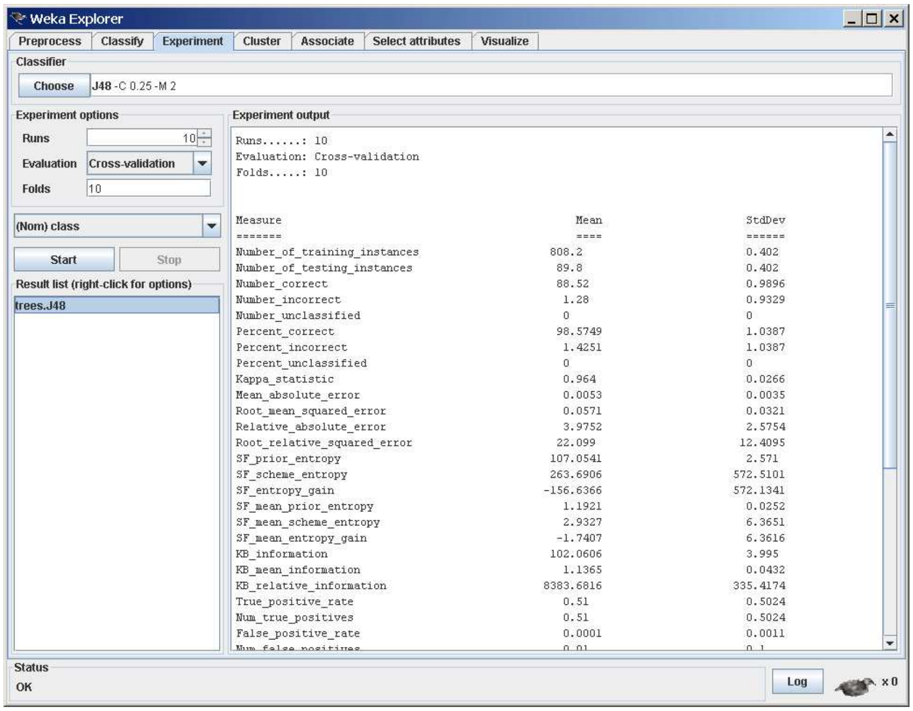

17.4.2 Adding visualization plugins

Introduction

As of WEKA version 3.5.3 you can easily add visualization plugins in the Explorer (Classify panel). This makes it easy to implement custom visualizations, if the ones WEKA offers are not sufficient. The following examples can be found in the Examples collection [3] (package `wekaexamples.gui.visualize.plugins`).

Requirements

- custom visualization class must implement the following interface

  `weka.gui.visualize.plugins.VisualizePlugin`

- the class must either reside in the following package (visualization classes are automatically discovered during run-time)

  `weka.gui.visualize.plugins`

- or you must list the package this class belongs to in the properties file `weka/gui/GenericPropertiesCreator.props` (or the equivalent in your home directory) under the key `weka.gui.visualize.plugins.VisualizePlugin`.

Implementation

The visualization interface contains the following four methods

- `getMinVersion` – This method returns the minimum version (inclusive) of WEKA that is necessary to execute the plugin, e.g., 3.5.0.
- `getMaxVersion` – This method returns the maximum version (exclusive) of WEKA that is necessary to execute the plugin, e.g., 3.6.0.
- `getDesignVersion` – Returns the actual version of WEKA this plugin was designed for, e.g., 3.5.1
- `getVisualizeMenuItem` – The `JMenuItem` that is returned via this method will be added to the plugins menu in the popup in the Explorer. The `ActionListener` for clicking the menu item will most likely open a new frame containing the visualized data.

Examples

Table with predictions

The `PredictionTable.java` example simply displays the actual class label and the one predicted by the classifier. In addition to that, it lists whether it was an incorrect prediction and the class probability for the correct class label.

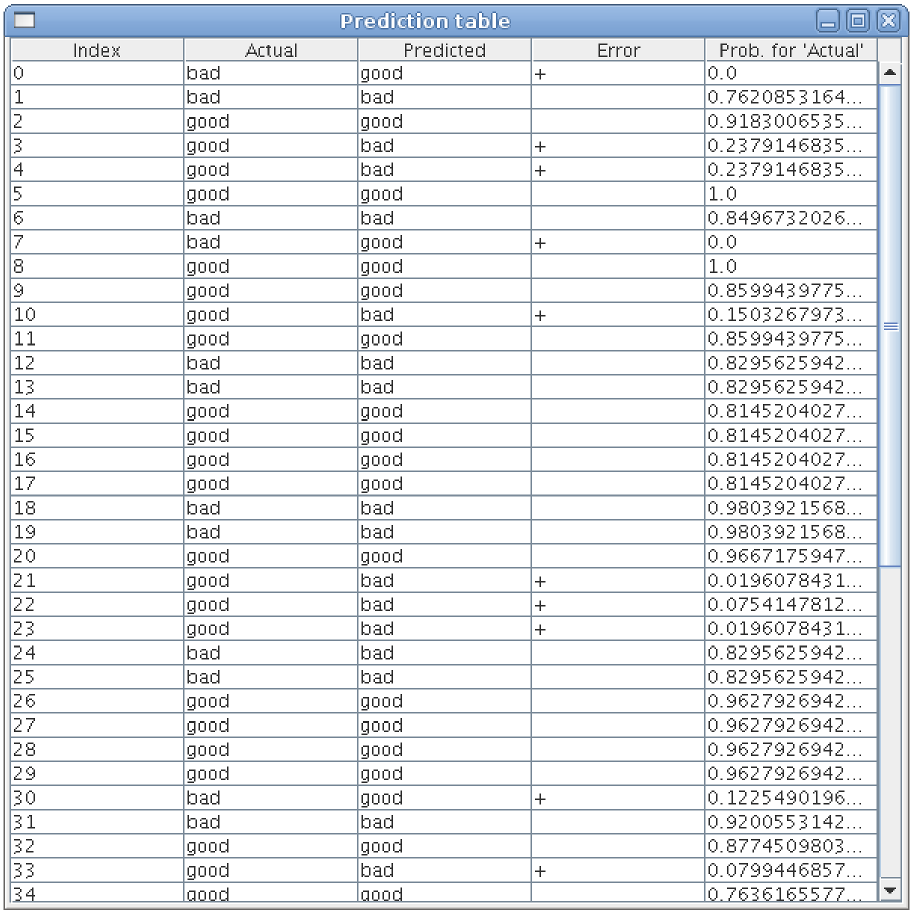

Index	Actual	Predicted	Error	Prob. for 'Actual'
0	bad	good	+	0.0
1	bad	bad		0.7620853164...
2	good	good		0.9183006535...
3	good	bad	+	0.2379146835...
4	good	bad	+	0.2379146835...
5	good	good		1.0
6	bad	bad		0.8496732026...
7	bad	good	+	0.0
8	good	good		1.0
9	good	good		0.8599439775...
10	good	bad	+	0.1503267973...
11	good	good		0.8599439775...
12	bad	bad		0.8295625942...
13	bad	bad		0.8295625942...
14	good	good		0.8145204027...
15	good	good		0.8145204027...
16	good	good		0.8145204027...
17	good	good		0.8145204027...
18	bad	bad		0.9803921568...
19	bad	bad		0.9803921568...
20	good	good		0.9667175947...
21	good	bad	+	0.0196078431...
22	good	bad	+	0.0754147812...
23	good	bad	+	0.0196078431...
24	bad	bad		0.8295625942...
25	bad	bad		0.8295625942...
26	good	good		0.9627926942...
27	good	good		0.9627926942...
28	good	good		0.9627926942...
29	good	good		0.9627926942...
30	bad	good	+	0.1225490196...
31	bad	bad		0.9200553142...
32	good	good		0.8774509803...
33	good	bad	+	0.0799446857...
34	good	good		0.7636165577...

Bar plot with probabilities

The `PredictionError.java` example uses the JMathTools library (needs the `jmathplot.jar` [27] in the CLASSPATH) to display a simple bar plot of the predictions. The correct predictions are displayed in blue, the incorrect ones in red. In both cases the class probability that the classifier returned for the correct class label is displayed on the y axis. The x axis is simply the index of the prediction starting with 0.

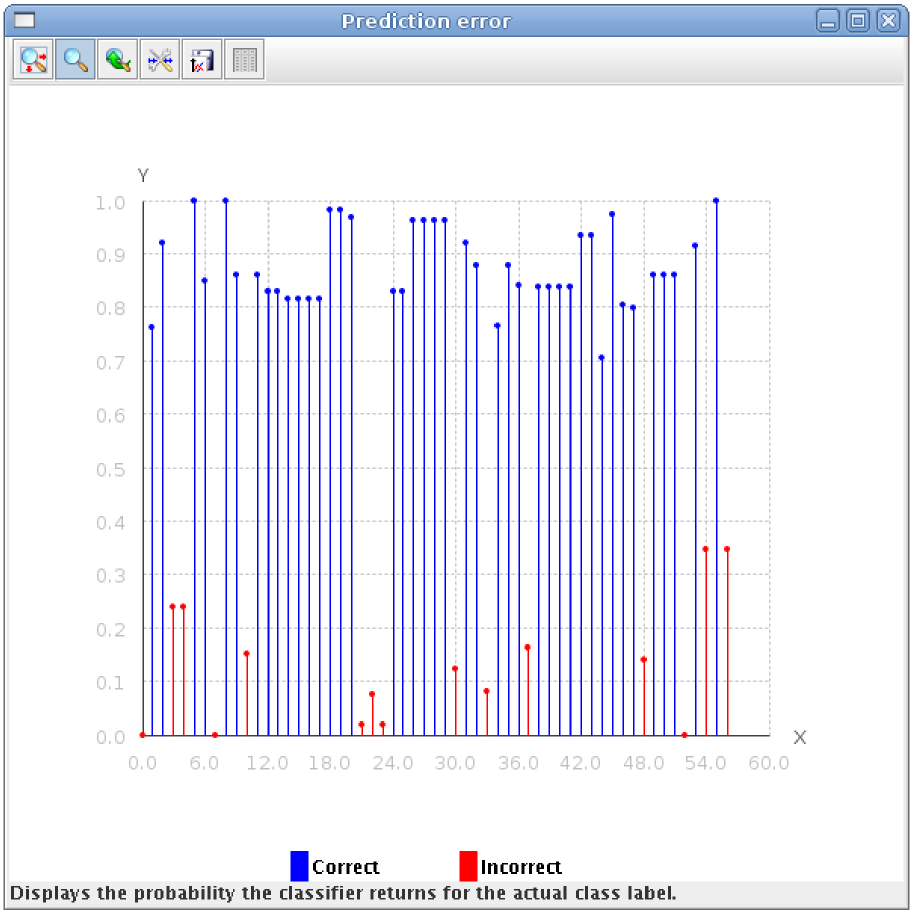

Chapter 18

Technical documentation

18.1 ANT

What is ANT? This is how the ANT homepage (`http://ant.apache.org/`) defines its tool:

Apache Ant is a Java-based build tool. In theory, it is kind of like Make, but without Make's wrinkles.

18.1.1 Basics

- the ANT build file is based on XML (`http://www.w3.org/XML/`)
- the usual name for the build file is:

  ```
  build.xml
  ```

- invocation—the usual build file needs not be specified explicitly, if it's in the current directory; if not target is specified, the default one is used

  ```
  ant [-f <build-file>] [<target>]
  ```

- displaying all the available targets of a build file

  ```
  ant [-f <build-file>] -projecthelp
  ```

18.1.2 Weka and ANT

- a build file for Weka is available from subversion
- some targets of interest
  - `clean`–Removes the build, dist and reports directories; also any class files in the source tree
  - `compile`–Compile weka and deposit class files in `${path_modifier}/build/classes`
  - `docs`–Make javadocs into `${path_modifier}/doc`
  - `exejar`–Create an executable jar file in `${path_modifier}/dist`

18.2 CLASSPATH

The CLASSPATH environment variable tells Java where to look for classes. Since Java does the search in a first-come-first-serve kind of manner, you'll have to take care where and what to put in your CLASSPATH. I, personally, never use the environment variable, since I'm working often on a project in different versions in parallel. The CLASSPATH would just mess up things, if you're not careful (or just forget to remove an entry). ANT (`http://ant.apache.org/`) offers a nice way for building (and separating source code and class files) Java projects. But still, if you're only working on totally separate projects, it might be easiest for you to use the environment variable.

18.2.1 Setting the CLASSPATH

In the following we add the `mysql-connector-java-5.1.7-bin.jar` to our `CLASSPATH` variable (this works for any other jar archive) to make it possible to access MySQL databases via JDBC.

Win32 (2k and XP)

We assume that the `mysql-connector-java-5.1.7-bin.jar` archive is located in the following directory:

```
C:\Program Files\Weka-3-7
```

In the *Control Panel* click on *System* (or right click on *My Computer* and select *Properties*) and then go to the *Avanced* tab. There you'll find a button called *Environment Variables*, click it. Depending on, whether you're the only person using this computer or it's a lab computer shared by many, you can either create a new system-wide (you're the only user) environment variable or a user dependent one (recommended for multi-user machines). Enter the following name for the variable.

```
CLASSPATH
```

and add this value

```
C:\Program Files\Weka-3-7\mysql-connector-java-5.1.7-bin.jar
```

If you want to add additional jars, you will have to separate them with the path separator, the semicolon ";" (no spaces!).

Unix/Linux

We make the assumption that the mysql jar is located in the following directory:

```
/home/johndoe/jars/
```

Open a shell and execute the following command, depending on the shell you're using:

- bash

```
export CLASSPATH=$CLASSPATH:/home/johndoe/jars/mysql-connector-java-5.1.7-bin.jar
```

- c shell

```
setenv CLASSPATH $CLASSPATH:/home/johndoe/jars/mysql-connector-java-5.1.7-bin.jar
```

Cygwin

The process is like with Unix/Linux systems, but since the host system is Win32 and therefore the Java installation also a Win32 application, you'll have to use the semicolon ; as separator for several jars.

18.2.2 RunWeka.bat

From version 3.5.4, Weka is launched differently under Win32. The simple batch file got replaced by a central launcher class (= `RunWeka.class`) in combination with an INI-file (= `RunWeka.ini`). The `RunWeka.bat` only calls this launcher class now with the appropriate parameters. With this launcher approach it is possible to define different launch scenarios, but with the advantage of having placeholders, e.g., for the max heap size, which enables one to change the memory for all setups easily.

The key of a command in the INI-file is prefixed with `cmd_`, all other keys are considered placeholders:

`cmd_blah=java ...` *command **blah***
`bloerk= ...` *placeholder **bloerk***

A placeholder is surrounded in a command with #:

```
cmd_blah=java #bloerk#
```

Note: The key *wekajar* is determined by the `-w` parameter with which the launcher class is called.

By default, the following commands are predefined:

- default
  The default Weka start, without a terminal window.
- console
  For debugging purposes. Useful as Weka gets started from a terminal window.
- explorer
  The command that's executed if one double-clicks on an ARFF or XRFF file.

In order to change the **maximum heap size** for all those commands, one only has to modify the **maxheap** placeholder.

For more information check out the comments in the INI-file.

18.2.3 java -jar

When you're using the Java interpreter with the **-jar** option, be aware of the fact that it **overwrites** your `CLASSPATH` and not **augments it**. Out of convenience, people often only use the **-jar** option to skip the declaration of the main class to start. But as soon as you need more jars, e.g., for database access, you need to use the **-classpath** option and specify the main class.

Here's once again how you start the Weka Main-GUI **with** your current `CLASSPATH` variable (and 128MB for the JVM):

- Linux
  `java -Xmx128m -classpath $CLASSPATH:weka.jar weka.gui.Main`
- Win32
  `java -Xmx128m -classpath "%CLASSPATH%;weka.jar" weka.gui.Main`

18.3 Subversion

18.3.1 General

The Weka *Subversion* repository is accessible and browseable via the following URL:

```
https://svn.scms.waikato.ac.nz/svn/weka/
```

A Subversion repository has usually the following layout:

```
root
 |
 +- trunk
 |
 +- tags
 |
 +- branches
```

Where *trunk* contains the *main trunk* of the development, *tags* snapshots in time of the repository (e.g., when a new version got released) and *branches* development branches that forked off the main trunk at some stage (e.g., legacy versions that still get bugfixed).

18.3.2 Source code

The latest version of the Weka source code can be obtained with this URL:

```
https://svn.scms.waikato.ac.nz/svn/weka/trunk/weka
```

If you want to obtain the source code of the book version, use this URL:

```
https://svn.scms.waikato.ac.nz/svn/weka/branches/book2ndEd-branch/weka
```

18.3.3 JUnit

The latest version of Weka's JUnit tests can be obtained with this URL:

```
https://svn.scms.waikato.ac.nz/svn/weka/trunk/tests
```

And if you want to obtain the JUnit tests of the book version, use this URL:

```
https://svn.scms.waikato.ac.nz/svn/weka/branches/book2ndEd-branch/tests
```

18.3.4 Specific version

Whenever a release of Weka is generated, the repository gets *tagged*

- `dev-X-Y-Z`
  the tag for a release of the developer version, e.g., *dev-3.7.0* for Weka 3.7.0
  `https://svn.scms.waikato.ac.nz/svn/weka/tags/dev-3-7-0`
- `stable-X-Y-Z`
  the tag for a release of the book version, e.g., *stable-3-4-15* for Weka 3.4.15
  `https://svn.scms.waikato.ac.nz/svn/weka/tags/stable-3-4-15`

18.3.5 Clients

Commandline

Modern Linux distributions already come with Subversion either pre-installed or easily installed via the package manager of the distribution. If that shouldn't be case, or if you are using Windows, you have to download the appropriate client from the Subversion homepage (`http://subversion.tigris.org/`).
A checkout of the current developer version of Weka looks like this:

```
svn co https://svn.scms.waikato.ac.nz/svn/weka/trunk/weka
```

SmartSVN

SmartSVN (`http://smartsvn.com/`) is a Java-based, graphical, cross-platform client for Subversion. Though it is not open-source/free software, the *foundation* version is for free.

TortoiseSVN

Under Windows, TortoiseCVS was a CVS client, neatly integrated into the Windows Explorer. TortoiseSVN (`http://tortoisesvn.tigris.org/`) is the equivalent for Subversion.

18.4 GenericObjectEditor

18.4.1 Introduction

As of version 3.4.4 it is possible for WEKA to dynamically discover classes at runtime (rather than using only those specified in the `GenericObjectEditor.props` (GOE) file). In some versions (3.5.8, 3.6.0) this facility was not enabled by default as it is a bit slower than the GOE file approach, and, furthermore, does not function in environments that do not have a CLASSPATH (e.g., application servers). Later versions (3.6.1, 3.7.0) enabled the dynamic discovery again, as WEKA can now distinguish between being a standalone Java application or being run in a non-CLASSPATH environment.

If you wish to enable or disable dynamic class discovery, the relevant file to edit is `GenericPropertiesCreator.props` (GPC). You can obtain this file either from the `weka.jar` or `weka-src.jar` archive. Open one of these files with an archive manager that can handle ZIP files (for Windows users, you can use 7-Zip (`http://7-zip.org/`) for this) and navigate to the `weka/gui` directory, where the GPC file is located. All that is required, is to change the `UseDynamic` property in this file from `false` to `true` (for enabling it) or the other way round (for disabling it). After changing the file, you just place it in your home directory. In order to find out the location of your home directory, do the following:

- Linux/Unix
  - Open a terminal
  - run the following command:
    `echo $HOME`
- Windows
  - Open a command-primpt
  - run the following command:
    `echo %USERPROFILE%`

If dynamic class discovery is too slow, e.g., due to an enormous CLASSPATH, you can generate a new `GenericObjectEditor.props` file and then turn dynamic class discovery off again. It is assumed that you already placed the GPC file in your home directory (see steps above) and that the `weka.jar` jar archive with the WEKA classes is in your CLASSPATH (otherwise you have to add it to the `java` call using the `-classpath` option).

For generating the GOE file, execute the following steps:

- generate a new `GenericObjectEditor.props` file using the following command:
  - Linux/Unix

    ```
    java weka.gui.GenericPropertiesCreator \
       $HOME/GenericPropertiesCreator.props \
       $HOME/GenericObjectEditor.props
    ```
  - Windows (command must be in one line)

    ```
    java weka.gui.GenericPropertiesCreator
       %USERPROFILE%\GenericPropertiesCreator.props
       %USERPROFILE%\GenericObjectEditor.props
    ```
- edit the `GenericPropertiesCreator.props` file in your home directory and set `UseDynamic` to `false`.

A limitation of the GOE prior to 3.4.4 was, that additional classifiers, filters, etc., had to fit into the same package structure as the already existing ones, i.e., all had to be located below `weka`. WEKA can now display multiple class hierarchies in the GUI, which makes adding new functionality quite easy as we will see later in an example (it is not restricted to classifiers only, but also works with all the other entries in the GPC file).

18.4.2 File Structure

The structure of the GOE is a key-value-pair, separated by an equals-sign. The value is a comma separated list of classes that are all derived from the superclass/superinterface *key*. The GPC is slightly different, instead of declaring all the classes/interfaces one need only to specify all the packages descendants are located in (only non-abstract ones are then listed). E.g., the `weka.classifiers.Classifier` entry in the GOE file looks like this:

```
weka.classifiers.Classifier=\
 weka.classifiers.bayes.AODE,\
 weka.classifiers.bayes.BayesNet,\
 weka.classifiers.bayes.ComplementNaiveBayes,\
 weka.classifiers.bayes.NaiveBayes,\
 weka.classifiers.bayes.NaiveBayesMultinomial,\
 weka.classifiers.bayes.NaiveBayesSimple,\
 weka.classifiers.bayes.NaiveBayesUpdateable,\
 weka.classifiers.functions.LeastMedSq,\
 weka.classifiers.functions.LinearRegression,\
 weka.classifiers.functions.Logistic,\
 ...
```

The entry producing the same output for the classifiers in the GPC looks like this (7 lines instead of over 70 for WEKA 3.4.4):

```
weka.classifiers.Classifier=\
 weka.classifiers.bayes,\
 weka.classifiers.functions,\
 weka.classifiers.lazy,\
 weka.classifiers.meta,\
 weka.classifiers.trees,\
 weka.classifiers.rules
```

18.4.3 Exclusion

It may not always be desired to list all the classes that can be found along the `CLASSPATH`. Sometimes, classes cannot be declared `abstract` but still shouldn't be listed in the GOE. For that reason one can list classes, interfaces, superclasses for certain packages to be excluded from display. This exclusion is done with the following file:

```
weka/gui/GenericPropertiesCreator.excludes
```

The format of this properties file is fairly simple:

```
<key>=<prefix>:<class>[,<prefix>:<class>]
```

Where the `<key>` corresponds to a key in the `GenericPropertiesCreator.props` file and the `<prefix>` can be one of the following:

- **S** – *Superclass*
  any class derived from this will be excluded
- **I** – *Interface*
  any class implementing this interface will be excluded
- **C** – *Class*
  exactly this class will be excluded

Here are a few examples:

```
# exclude all ResultListeners that also implement the ResultProducer interface
# (all ResultProducers do that!)
weka.experiment.ResultListener=\
  I:weka.experiment.ResultProducer

# exclude J48 and all SingleClassifierEnhancers
weka.classifiers.Classifier=\
  C:weka.classifiers.trees.J48,\
  S:weka.classifiers.SingleClassifierEnhancer
```

18.4.4 Class Discovery

Unlike the `Class.forName(String)` method that grabs the first class it can find in the `CLASSPATH`, and therefore fixes the location of the package it found the class in, the dynamic discovery examines the complete `CLASSPATH` you are starting the Java Virtual Machine (= JVM) with. This means that you can have several parallel directories with the same WEKA package structure, e.g., the standard release of WEKA in one directory (`/distribution/weka.jar`) and another one with your own classes (`/development/weka/...`), and display all of the classifiers in the GUI. In case of a name conflict, i.e., two directories contain the same class, the first one that can be found is used. In a nutshell, your `java` call of the GUIChooser can look like this:

```
java -classpath "/development:/distribution/weka.jar" weka.gui.GUIChooser
```

Note: Windows users have to replace the ":" with ";" and the forward slashes with backslashes.

18.4.5 Multiple Class Hierarchies

In case you are developing your own framework, but still want to use your classifiers within WEKA that was not possible with WEKA prior to 3.4.4. Starting with the release 3.4.4 it is possible to have multiple class hierarchies being displayed in the GUI. If you have developed a modified version of NaiveBayes, let us call it *DummyBayes* and it is located in the package `dummy.classifiers` then you will have to add this package to the classifiers list in the GPC file like this:

```
weka.classifiers.Classifier=\
 weka.classifiers.bayes,\
 weka.classifiers.functions,\
 weka.classifiers.lazy,\
 weka.classifiers.meta,\
 weka.classifiers.trees,\
 weka.classifiers.rules,\
 dummy.classifiers
```

Your java call for the GUIChooser might look like this:

```
java -classpath "weka.jar:dummy.jar" weka.gui.GUIChooser
```

Starting up the GUI you will now have another root node in the tree view of the classifiers, called *root*, and below it the weka and the *dummy* package hierarchy as you can see here:

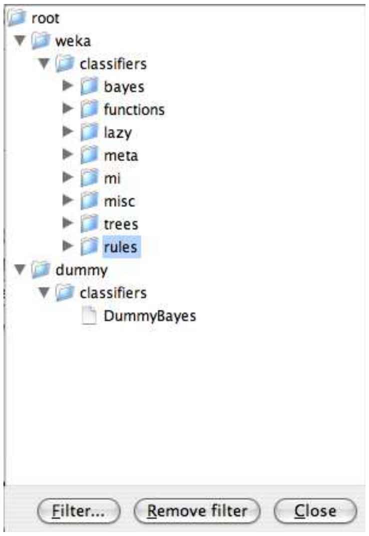

18.4.6 Capabilities

Version **3.5.3** of Weka introduced the notion of *Capabilities*. Capabilities basically list what kind of data a certain object can handle, e.g., one classifier can handle numeric classes, but another cannot. In case a class supports capabilities the additional buttons **Filter...** and **Remove filter** will be available in the GOE. The Filter... button pops up a dialog which lists all available Capabilities:

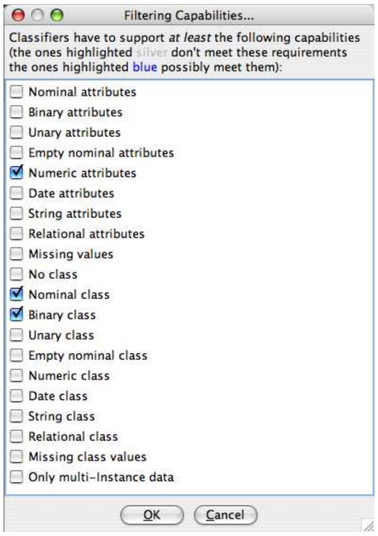

One can then choose those capabilities an object, e.g., a classifier, should have. If one is looking for classification problem, then the *Nominal* class Capability can be selected. On the other hand, if one needs a regression scheme, then the Capability *Numeric* class can be selected. This filtering mechanism makes the search for an appropriate learning scheme easier. After applying that filter, the tree with the objects will be displayed again and lists all objects that can handle all the selected Capabilities black, the ones that cannot grey and the ones that might be able to handle them blue (e.g., meta classifiers which depend on their base classifier(s)).

18.5 Properties

A properties file is a simple text file with this structure:

```
<key>=<value>
```

Comments start with the hash sign #.

To make a rather long property line more readable, one can use a backslash to continue on the next line. The Filter property, e.g., looks like this:

```
weka.filters.Filter= \
 weka.filters.supervised.attribute, \
 weka.filters.supervised.instance, \
 weka.filters.unsupervised.attribute, \
 weka.filters.unsupervised.instance
```

18.5.1 Precedence

The Weka property files (extension .props) are searched for in the following order:

- current directory
- the user's home directory (*nix `$HOME`, Windows `%USERPROFILE%`)
- the class path (normally the weka.jar file)

If Weka encounters those files it only supplements the properties, never overrides them. In other words, a property in the property file of the current directory has a higher precedence than the one in the user's home directory.

Note: Under Cywgin (`http://cygwin.com/`), the home directory is still the Windows one, since the java installation will be still one for Windows.

18.5.2 Examples

- weka/gui/LookAndFeel.props
- weka/gui/GenericPropertiesCreator.props
- weka/gui/beans/Beans.props

18.6 XML

Weka now supports XML (http://www.w3c.org/XML/) (eXtensible Markup Language) in several places.

18.6.1 Command Line

WEKA now allows Classifiers and Experiments to be started using an -xml option followed by a filename to retrieve the command line options from the XML file instead of the command line.

For such simple classifiers like e.g. J48 this looks like overkill, but as soon as one uses Meta-Classifiers or Meta-Meta-Classifiers the handling gets tricky and one spends a lot of time looking for missing quotes. With the hierarchical structure of XML files it is simple to plug in other classifiers by just exchanging tags.

The DTD for the XML options is quite simple:

```
<!DOCTYPE options
[
   <!ELEMENT options (option)*>
   <!ATTLIST options type CDATA "classifier">
   <!ATTLIST options value CDATA "">
   <!ELEMENT option (#PCDATA | options)*>
   <!ATTLIST option name CDATA #REQUIRED>
   <!ATTLIST option type (flag | single | hyphens | quotes) "single">
]
>
```

The type attribute of the option tag needs some explanations. There are currently four different types of options in WEKA:

- **flag**
  The simplest option that takes no arguments, like e.g. the -V flag for inversing an selection.

  ```
  <option name="V" type="flag"/>
  ```

- **single**
  The option takes exactly one parameter, directly following after the option, e.g., for specifying the trainings file with -t somefile.arff. Here the parameter value is just put between the opening and closing tag. Since single is the default value for the type tag we don't need to specify it explicitly.

  ```
  <option name="t">somefile.arff</option>
  ```

- **hyphens**
  Meta-Classifiers like `AdaBoostM1` take another classifier as option with the `-W` option, where the options for the base classifier follow after the `--`. And here it is where the fun starts: where to put parameters for the base classifier if the Meta-Classifier itself is a base classifier for another Meta-Classifier?

  E.g., does `-W weka.classifiers.trees.J48 -- -C 0.001` become this:

```
<option name="W" type="hyphens">
   <options type="classifier" value="weka.classifiers.trees.J48">
      <option name="C">0.001</option>
   </options>
</option>
```

  Internally, all the options enclosed by the `options` tag are pushed to the end after the `--` if one transforms the XML into a command line string.

- **quotes**
  A Meta-Classifier like `Stacking` can take several `-B` options, where each single one encloses other options in quotes (this itself can contain a Meta-Classifier!). From `-B ‘‘weka.classifiers.trees.J48’’` we then get this XML:

```
<option name="B" type="quotes">
   <options type="classifier" value="weka.classifiers.trees.J48"/>
</option>
```

  With the XML representation one doesn't have to worry anymore about the level of quotes one is using and therefore doesn't have to care about the correct escaping (i.e. `‘‘ ... \" ... \" ...’’`) since this is done automatically.

And if we now put all together we can transform this more complicated command line (`java` and the `CLASSPATH` omitted):

```
<options type="class" value="weka.classifiers.meta.Stacking">
   <option name="B" type="quotes">
      <options type="classifier" value="weka.classifiers.meta.AdaBoostM1">
         <option name="W" type="hyphens">
            <options type="classifier" value="weka.classifiers.trees.J48">
               <option name="C">0.001</option>
            </options>
         </option>
      </options>
   </option>

   <option name="B" type="quotes">
      <options type="classifier" value="weka.classifiers.meta.Bagging">
         <option name="W" type="hyphens">
            <options type="classifier" value="weka.classifiers.meta.AdaBoostM1">
               <option name="W" type="hyphens">
                  <options type="classifier" value="weka.classifiers.trees.J48"/>
               </option>
            </options>
         </option>
      </options>
   </option>

   <option name="B" type="quotes">
      <options type="classifier" value="weka.classifiers.meta.Stacking">
         <option name="B" type="quotes">
            <options type="classifier" value="weka.classifiers.trees.J48"/>
         </option>
      </options>
   </option>

   <option name="t">test/datasets/hepatitis.arff</option>
</options>
```

Note: The `type` and `value` attribute of the outermost `options` tag is not used while reading the parameters. It is merely for documentation purposes, so that one knows which class was actually started from the command line.

Responsible Class(es):

`weka.core.xml.XMLOptions`

18.6.2 Serialization of Experiments

It is now possible to serialize the Experiments from the WEKA Experimenter not only in the proprietary binary format Java offers with serialization (with this you run into problems trying to read old experiments with a newer WEKA version, due to different SerialUIDs), but also in XML. There are currently two different ways to do this:

- **built-in**
  The built-in serialization captures only the necessary informations of an experiment and doesn't serialize anything else. It's sole purpose is to save the setup of a specific experiment and can therefore not store any built models. Thanks to this limitation we'll never run into problems with mismatching SerialUIDs.

  This kind of serialization is always available and can be selected via a Filter (*.xml) in the Save/Open-Dialog of the Experimenter.

  The DTD is very simple and looks like this (for version 3.4.5):

```
<!DOCTYPE object[
   <!ELEMENT object (#PCDATA | object)*>
   <!ATTLIST object name      CDATA #REQUIRED>
   <!ATTLIST object class     CDATA #REQUIRED>
   <!ATTLIST object primitive CDATA "no">
   <!ATTLIST object array     CDATA "no">
   <!ATTLIST object null      CDATA "no">
   <!ATTLIST object version   CDATA "3.4.5">
]>
```

  Prior to versions 3.4.5 and 3.5.0 it looked like this:

```
<!DOCTYPE object
[
   <!ELEMENT object (#PCDATA | object)*>
   <!ATTLIST object name      CDATA #REQUIRED>
   <!ATTLIST object class     CDATA #REQUIRED>
   <!ATTLIST object primitive CDATA "yes">
   <!ATTLIST object array     CDATA "no">
]
>
```

  Responsible Class(es):

```
weka.experiment.xml.XMLExperiment
```

  for general Serialization:

```
weka.core.xml.XMLSerialization
weka.core.xml.XMLBasicSerialization
```

- **KOML** (`http://old.koalateam.com/xml/serialization/`)
  The Koala Object Markup Language (KOML) is published under the LGPL (`http://www.gnu.org/copyleft/lgpl.html`) and is an alternative way of serializing and derserializing Java Objects in an XML file. Like the normal serialization it serializes everything into XML via an ObjectOutputStream, including the SerialUID of each class. Even though we have the same problems with mismatching SerialUIDs it is at least possible edit the XML files by hand and replace the offending IDs with the new ones.

  In order to use KOML one only has to assure that the KOML classes are in the CLASSPATH with which the Experimenter is launched. As soon as KOML is present another Filter (`*.koml`) will show up in the Save/Open-Dialog.

  The DTD for KOML can be found at `http://old.koalateam.com/xml/koml12.dtd`

  Responsible Class(es):

  `weka.core.xml.KOML`

The experiment class can of course read those XML files if passed as input or output file (see options of `weka.experiment.Experiment` and `weka.experiment.RemoteExperiment`

18.6.3 Serialization of Classifiers

The options for models of a classifier, `-l` for the input model and `-d` for the output model, now also supports XML serialized files. Here we have to differentiate between two different formats:

- **built-in**
  The built-in serialization captures only the options of a classifier but not the built model. With the `-l` one still has to provide a training file, since we only retrieve the options from the XML file. It is possible to add more options on the command line, but it is no check performed whether they collide with the ones stored in the XML file.

  The file is expected to end with `.xml`.

- **KOML**
  Since the KOML serialization captures everything of a Java Object we can use it just like the normal Java serialization.

  The file is expected to end with `.koml`.

The **built-in** serialization can be used in the **Experimenter** for loading/saving options from algorithms that have been added to a Simple Experiment. Unfortunately it is not possible to create such a hierarchical structure like mentioned in Section 18.6.1. This is because of the loss of information caused by the `getOptions()` method of classifiers: it returns only a flat String-Array and not a tree structure.

Responsible Class(es):

```
weka.core.xml.KOML
weka.classifiers.xml.XMLClassifier
```

18.6.4 Bayesian Networks

The GraphVisualizer (`weka.gui.graphvisualizer.GraphVisualizer`) can save graphs into the Interchange Format (`http://www-2.cs.cmu.edu/~fgcozman/Research/InterchangeFormat/`) for Bayesian Networks (BIF). If started from command line with an XML filename as first parameter and not from the Explorer it can display the given file directly.

The DTD for BIF is this:

```
<!DOCTYPE BIF [
   <!ELEMENT BIF ( NETWORK )*>
         <!ATTLIST BIF VERSION CDATA #REQUIRED>
   <!ELEMENT NETWORK ( NAME, ( PROPERTY | VARIABLE | DEFINITION )* )>
   <!ELEMENT NAME (#PCDATA)>

   <!ELEMENT VARIABLE ( NAME, ( OUTCOME |  PROPERTY )* ) >
         <!ATTLIST VARIABLE TYPE (nature|decision|utility) "nature">
   <!ELEMENT OUTCOME (#PCDATA)>
   <!ELEMENT DEFINITION ( FOR | GIVEN | TABLE | PROPERTY )* >
   <!ELEMENT FOR (#PCDATA)>
   <!ELEMENT GIVEN (#PCDATA)>

   <!ELEMENT TABLE (#PCDATA)>
   <!ELEMENT PROPERTY (#PCDATA)>
]>
```

Responsible Class(es):

```
weka.classifiers.bayes.BayesNet#toXMLBIF03()
weka.classifiers.bayes.net.BIFReader
weka.gui.graphvisualizer.BIFParser
```

18.6.5 XRFF files

With Weka 3.5.4 a new, more feature-rich, XML-based data format got introduced: **XRFF**. For more information, please see Chapter 10.

Chapter 19

Other resources

19.1 Mailing list

The WEKA Mailing list can be found here:

- `http://list.scms.waikato.ac.nz/mailman/listinfo/wekalist`
  for subscribing/unsubscribing the list
- `https://list.scms.waikato.ac.nz/pipermail/wekalist/`
  (Mirrors: `http://news.gmane.org/gmane.comp.ai.weka`,
  `http://www.nabble.com/WEKA-f435.html`)
  for searching previous posted messages

Before posting, please read the Mailing List Etiquette:
`http://www.cs.waikato.ac.nz/~ml/weka/mailinglist_etiquette.html`

19.2 Troubleshooting

Here are a few of things that are useful to know when you are having trouble installing or running Weka successfullyon your machine.

NB these java commands refer to ones executed in a shell (bash, command prompt, etc.) and **NOT** to commands executed in the SimpleCLI.

19.2.1 Weka download problems

When you **download Weka**, make sure that the resulting file size is the same as on our webpage. Otherwise things won't work properly. Apparently some web browsers have trouble downloading Weka.

19.2.2 OutOfMemoryException

Most Java virtual machines only allocate a certain maximum amount of memory to run Java programs. Usually this is much less than the amount of RAM in your computer. However, you can extend the memory available for the virtual machine by setting appropriate options. With Sun's JDK, for example, you can go

```
java -Xmx100m ...
```

to set the maximum Java heap size to 100MB. For more information about these options see `http://java.sun.com/docs/hotspot/VMOptions.html`.

19.2.2.1 Windows

Book version
You have to modify the JVM invocation in the `RunWeka.bat` batch file in your installation directory.

Developer version

- up to **Weka 3.5.2**
  just like the book version.

- **Weka 3.5.3**
  You have to modify the link in the Windows Start menu, if you're starting the console-less Weka (only the link with console in its name executes the `RunWeka.bat` batch file)

- **Weka 3.5.4** and higher Due to the new launching scheme, you no longer modify the batch file, but the `RunWeka.ini` file. In that particular file, you'll have to change the **maxheap** placeholder. See section 18.2.2.

19.2.3 Mac OSX

In your Weka installation directory (`weka-3-x-y.app`) locate the `Contents` subdirectory and edit the `Info.plist` file. Near the bottom of the file you should see some text like:

```
<key>VMOptions</key>
<string>-Xmx256M</string>
```

Alter the `256M` to something higher.

19.2.4 StackOverflowError

Try increasing the stack of your virtual machine. With Sun's JDK you can use this command to increase the stacksize:

```
java -Xss512k ...
```

to set the maximum Java stack size to 512KB. If still not sufficient, slowly increase it.

19.2.5 just-in-time (JIT) compiler

For maximum enjoyment, use a virtual machine that incorporates a **just-in-time compiler**. This can speed things up quite significantly. Note also that there can be large differences in execution time between different virtual machines.

19.2.6 CSV file conversion

Either load the CSV file in the Explorer or use the CVS converter on the commandline as follows:

```
java weka.core.converters.CSVLoader filename.csv > filename.arff
```

19.2.7 ARFF file doesn't load

One way to figure out why ARFF files are failing to load is to give them to the Instances class. At the command line type the following:

```
java weka.core.Instances filename.arff
```

where you substitute 'filename' for the actual name of your file. This should return an error if there is a problem reading the file, or show some statistics if the file is ok. The error message you get should give some indication of what is wrong.

19.2.8 Spaces in labels of ARFF files

A common problem people have with ARFF files is that labels can only have spaces if they are enclosed in single quotes, i.e. a label such as:

```
some value
```

should be written either 'some value' or some_value in the file.

19.2.9 CLASSPATH problems

Having problems getting Weka to run from a DOS/UNIX command prompt? Getting `java.lang.NoClassDefFoundError` exceptions? Most likely your **CLASSPATH** environment variable is not set correctly - it needs to point to the Weka.jar file that you downloaded with Weka (or the parent of the Weka directory if you have extracted the jar). Under DOS this can be achieved with:

```
set CLASSPATH=c:\weka-3-4\weka.jar;%CLASSPATH%
```

Under UNIX/Linux something like:

```
export CLASSPATH=/home/weka/weka.jar:$CLASSPATH
```

An easy way to get avoid setting the variable this is to specify the CLASSPATH when calling Java. For example, if the jar file is located at c:\weka-3-4\weka.jar you can use:

```
java -cp c:\weka-3-4\weka.jar weka.classifiers... etc.
```

See also Section 18.2.

19.2.10 Instance ID

People often want to **tag** their **instances with identifiers**, so they can keep track of them and the predictions made on them.

19.2.10.1 Adding the ID

A new ID attribute is added real easy: one only needs to run the `AddID` filter over the dataset and it's done. Here's an example (at a DOS/Unix command prompt):

```
java weka.filters.unsupervised.attribute.AddID
  -i data_without_id.arff
  -o data_with_id.arff
```

(all on a single line).

Note: the AddID filter adds a numeric attribute, not a String attribute to the dataset. If you want to remove this ID attribute for the classifier in a `FilteredClassifier` environment again, use the `Remove` filter instead of the `RemoveType` filter (same package).

19.2.10.2 Removing the ID

If you run from the command line you can use the `-p` option to output predictions plus any other attributes you are interested in. So it is possible to have a string attribute in your data that acts as an identifier. A problem is that most classifiers don't like String attributes, but you can get around this by using the `RemoveType` (this removes *String* attributes by default).

Here's an example. Lets say you have a training file named `train.arff`, a testing file named `test.arff`, and they have an identifier String attribute as their 5th attribute. You can get the predictions from `J48` along with the identifier strings by issuing the following command (at a DOS/Unix command prompt):

```
java weka.classifiers.meta.FilteredClassifier
  -F weka.filters.unsupervised.attribute.RemoveType
  -W weka.classifiers.trees.J48
  -t train.arff -T test.arff -p 5
```

(all on a single line).

If you want, you can redirect the output to a file by adding "`> output.txt`" to the end of the line.

In the Explorer GUI you could try a similar trick of using the String attribute identifiers here as well. Choose the `FilteredClassifier`, with `RemoveType` as the filter, and whatever classifier you prefer. When you visualize the results you will need click through each instance to see the identifier listed for each.

19.2.11 Visualization

Access to **visualization** from the ClassifierPanel, ClusterPanel and Attribute-Selection panel is available from a popup menu. Click the right mouse button over an entry in the Result list to bring up the menu. You will be presented with options for viewing or saving the text output and—depending on the scheme—further options for visualizing errors, clusters, trees etc.

19.2.12 Memory consumption and Garbage collector

There is the ability to print **how much memory is available** in the Explorer and Experimenter and to run the garbage collector. Just right click over the Status area in the Explorer/Experimenter.

19.2.13 GUIChooser starts but not Experimenter or Explorer

The GUIChooser starts, but Explorer and Experimenter don't start and output an Exception like this in the terminal:

```
/usr/share/themes/Mist/gtk-2.0/gtkrc:48: Engine "mist" is unsupported, ignoring
---Registering Weka Editors---
java.lang.NullPointerException
       at weka.gui.explorer.PreprocessPanel.addPropertyChangeListener(PreprocessPanel.java:519)
       at javax.swing.plaf.synth.SynthPanelUI.installListeners(SynthPanelUI.java:49)
       at javax.swing.plaf.synth.SynthPanelUI.installUI(SynthPanelUI.java:38)
       at javax.swing.JComponent.setUI(JComponent.java:652)
       at javax.swing.JPanel.setUI(JPanel.java:131)
       ...
```

This behavior happens only under Java 1.5 and Gnome/Linux, KDE doesn't produce this error. The reason for this is, that Weka tries to look more "native" and therefore sets a platform-specific Swing theme. Unfortunately, this doesn't seem to be working correctly in Java 1.5 together with Gnome. A workaround for this is to set the cross-platform **Metal** theme.

In order to use another theme one only has to create the following properties file in ones home directory:

```
LookAndFeel.props
```

With this content:

```
Theme=javax.swing.plaf.metal.MetalLookAndFeel
```

19.2.14 KnowledgeFlow toolbars are empty

In the terminal, you will most likely see this output as well:

```
Failed to instantiate: weka.gui.beans.Loader
```

This behavior can happen under Gnome with Java 1.5, see Section 19.2.13 for a solution.

19.2.15 Links

- Java VM options (`http://java.sun.com/docs/hotspot/VMOptions.html`)

Bibliography

[1] Witten, I.H. and Frank, E. (2005) *Data Mining: Practical machine learning tools and techniques. 2nd edition* Morgan Kaufmann, San Francisco.

[2] *WekaWiki* – `http://weka.wikispaces.com/`

[3] *Weka Examples* – A collection of example classes, as part of an ANT project, included in the WEKA snapshots (available for download on the homepage) or directly from subversion `https://svn.scms.waikato.ac.nz/svn/weka/branches/stable-3-6/wekaexamples/`

[4] J. Platt (1998): Machines using Sequential Minimal Optimization. In B. Schoelkopf and C. Burges and A. Smola, editors, Advances in Kernel Methods - Support Vector Learning.

[5] Drummond, C. and Holte, R. (2000) Explicitly representing expected cost: An alternative to ROC representation. *Proceedings of the Sixth ACM SIGKDD International Conference on Knowledge Discovery and Data Mining.* Publishers, San Mateo, CA.

[6] *Extensions for Weka's main GUI* on *WekaWiki* – `http://weka.wikispaces.com/Extensions+for+Weka%27s+main+GUI`

[7] *Adding tabs in the Explorer* on *WekaWiki* – `http://weka.wikispaces.com/Adding+tabs+in+the+Explorer`

[8] *Explorer visualization plugins* on *WekaWiki* – `http://weka.wikispaces.com/Explorer+visualization+plugins`

[9] Bengio, Y. and Nadeau, C. (1999) *Inference for the Generalization Error.*

[10] Ross Quinlan (1993). *C4.5: Programs for Machine Learning*, Morgan Kaufmann Publishers, San Mateo, CA.

[11] *Subversion* – `http://weka.wikispaces.com/Subversion`

[12] *HSQLDB* – `http://hsqldb.sourceforge.net/`

[13] *MySQL* – `http://www.mysql.com/`

[14] *Plotting multiple ROC curves* on *WekaWiki* – `http://weka.wikispaces.com/Plotting+multiple+ROC+curves`

[15] R.R. Bouckaert. Bayesian Belief Networks: from Construction to Inference. Ph.D. thesis, University of Utrecht, 1995.

[16] W.L. Buntine. A guide to the literature on learning probabilistic networks from data. IEEE Transactions on Knowledge and Data Engineering, 8:195–210, 1996.

[17] J. Cheng, R. Greiner. Comparing bayesian network classifiers. Proceedings UAI, 101–107, 1999.

[18] C.K. Chow, C.N.Liu. Approximating discrete probability distributions with dependence trees. IEEE Trans. on Info. Theory, IT-14: 426–467, 1968.

[19] G. Cooper, E. Herskovits. A Bayesian method for the induction of probabilistic networks from data. Machine Learning, 9: 309–347, 1992.

[20] Cozman. See `http://www-2.cs.cmu.edu/~fgcozman/Research/InterchangeFormat/` for details on XML BIF.

[21] N. Friedman, D. Geiger, M. Goldszmidt. Bayesian Network Classifiers. Machine Learning, 29: 131–163, 1997.

[22] D. Heckerman, D. Geiger, D. M. Chickering. Learning Bayesian networks: the combination of knowledge and statistical data. Machine Learining, 20(3): 197–243, 1995.

[23] S.L. Lauritzen and D.J. Spiegelhalter. Local Computations with Probabilities on graphical structures and their applications to expert systems (with discussion). Journal of the Royal Statistical Society B. 1988, 50, 157-224

[24] Moore, A. and Lee, M.S. Cached Sufficient Statistics for Efficient Machine Learning with Large Datasets, JAIR, Volume 8, pages 67-91, 1998.

[25] Verma, T. and Pearl, J.: An algorithm for deciding if a set of observed independencies has a causal explanation. Proc. of the Eighth Conference on Uncertainty in Artificial Intelligence, 323-330, 1992.

[26] GraphViz. See `http://www.graphviz.org/doc/info/lang.html` for more information on the DOT language.

[27] JMathPlot. See `http://code.google.com/p/jmathplot/` for more information on the project.
